# The Histone Acetyltransferase GCN5 and the Associated Coactivators ADA2: From Evolution of the SAGA Complex to the Biological Roles in Plants

**DOI:** 10.3390/plants10020308

**Published:** 2021-02-05

**Authors:** Konstantinos Vlachonasios, Stylianos Poulios, Niki Mougiou

**Affiliations:** Department of Botany, School of Biology, Aristotle University of Thessaloniki, 54124 Thessaloniki, Greece; spoulios@bio.auth.gr (S.P.); nmougiou@bio.auth.gr (N.M.)

**Keywords:** *Arabidopsis thaliana*, Viridiplantae, GCN5, ADA2b, SGF29, ADA3, plant development, plant stress responses, SAGA, histone acetylation

## Abstract

Transcription of protein-encoding genes starts with forming a pre-initiation complex comprised of RNA polymerase II and several general transcription factors. To activate gene expression, transcription factors must overcome repressive chromatin structure, which is accomplished with multiprotein complexes. One such complex, SAGA, modifies the nucleosomal histones through acetylation and other histone modifications. A prototypical histone acetyltransferase (HAT) known as general control non-repressed protein 5 (GCN5), was defined biochemically as the first transcription-linked HAT with specificity for histone H3 lysine 14. In this review, we analyze the components of the putative plant SAGA complex during plant evolution, and current knowledge on the biological role of the key components of the HAT module, GCN5 and ADA2b in plants, will be summarized.

## 1. The Discovery of Histone Acetyltransferase GCN5 and the Associated SAGA Complex

Transcription of protein-encoding genes starts with forming a pre-initiation complex comprised of RNA polymerase II and several general transcription factors [1]. To activate gene expression, transcription factors must overcome the repressive chromatin structure, which is accomplished with multiprotein complexes [2]. Chromatin-modifying coactivators dynamically deposit or remove post-translational modifications (PTMs) on histones, creating or erasing docking surfaces for specific regulatory factors [2]. One class involves complexes that modify the nucleosomal histones through acetylation, phosphorylation, methylation and other modifications [3]. Histone acetyltransferases (HATs) catalyze acetylation of specific lysine residues in histone N-tails, which are involved in transcriptional regulation and other nuclear processes. HATs are parts of large multiprotein complexes, like the SAGA complex, where their activity is enhanced and their substrate specificity is altered. The whole complex is recruited to target sequences on the genome with other components involved in protein-protein interactions [4]. HATs and histone deacetylases (HDACs) can target promoters for either the activation or suppression of gene expression [2]. A prototypical HAT which acts as a transcriptional adaptor is known as GENERAL CONTROL NON-REPRESSED PROTEIN 5 (GCN5), first identified in *Saccharomyces cerevisiae* [5,6]. GCN5 was defined biochemically as the first transcription-linked HAT [7] with specificity for histone H3 lysine 14 (H3K14) [8]. However, GCN5 could also acetylate additional histone lysine residues, such as H3K9, H3K18, H3K23, H3K27, H3K36, and other histones such as H4 and H2B [9,10]. Furthermore, GCN5 was the HAT component of two distinct transcriptional adaptor complexes, SAGA (Spt-Ada-Gcn5-acetyltransferase) and ADA, capable of acetylating histones in nucleosomes [9]. These complexes are conserved in many eukaryotes [11] and have multiple distinct functions which lead to transcriptional activation. In yeast, SAGA is a large multi-subunit protein complex composed of at least 19 proteins [11,12]. These proteins are separated into four distinct modules, with two specific enzymatic activities: the HAT module that acetylates histones and contains GCN5, ADA2, ADA3, and SGF29; the deubiquitylase (DUB) module that triggers deubiquitination of histone H2B and includes UBP8, SGF11, SGF73, and SUS1; the suppressor of Ty (SPT) module that contains TRA1, ADA1, SPT3, SPT7, SPT8, and SPT20 (ADA5), and the TATA-binding protein (TBP)-associated factor (TAF) module that includes TAF5, TAF6, TAF9, TAF10, and TAF12 [4,12]. Recently, new structural studies showed that most of the proteins of SPT and TAF modules form a core module (COREm) [13,14]. The core module binds to TBP and consists of subunit TAF5, SPT20, and a histone octamer-like fold. The histone octamer-like fold comprises the heterodimers TAF6-TAF9, TAF10-SPT7, TAF12-ADA1, and two histone-fold domains in SPT3 [13,14]. SGF73 subunit is in association with DUBm and COREm. When a nucleosome binds to a SAGA complex, the HAT and the DUB modules are displaced from the COREm [14]. Several proteins in the SAGA complex have distinct protein-binding domains, writers, or readers domains (HAT, tudor, bromodomains etc.) that enable SAGA to associate with chromatin or other proteins through PTMs. Furthermore, SAGA proteins are also part of other complexes [11]. For instance, GCN5 is also part of the human ATAC complex [15]. TRA1 is also a component of the NuA4 acetyltransferase complex [16]. The proteins of the TAF module are also components of the TFIID complex [17].

## 2. The Plant SAGA Complex

**Figure 1:**
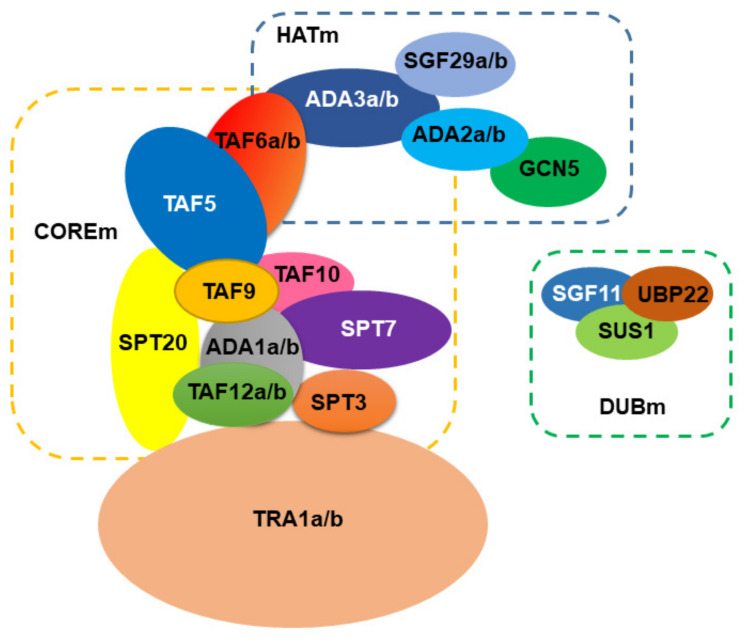
A model for the organization of SAGA complex in *Arabidopsis*.

Using the genome of *Arabidopsis thaliana* as a reference for plants, the SAGA complex is comprised of approximately 24 proteins (Figure 1). Using the current structure of the yeast SAGA complex [13,14] the HAT module (HATm) in *Arabidopsis* consists of the same proteins, GCN5, ADA2, ADA3, and SGF29. However, the *Arabidopsis* HATm contains two subunits of ADA2, ADA3, and SGF29 (designated as ADA2a, ADA2b, ADA3a, ADA3b, SGF29a, and SGF29b, respectively). Based on this structure, the *Arabidopsis* COREm consists of similar TAF proteins (TAF5, TAF6, TAF9, TAF10, and TAF12), and the adaptor proteins ADA1 and SPT20. The yeast SPT module consists of another three subunits SPT3, SPT7, and SPT8, distinct from the plant homologues. For instance, in *Arabidopsis* genome, SPT8 is not encoded; however, the presence of multiple WD40 domains in yeast Spt8 makes the evolutionary information challenging [15]. Furthermore, *Arabidopsis* SPT3 homologue is like TAF13, whereas SPT7 homologues appear to have a conserved bromodomain found in AtHAF1 (TAF1) subunit. Interestingly, several subunits from the COREm are duplicated in *Arabidopsis* including ADA1, TAF6, and TAF12. The COREm occupies a central position in the complex and is connected to the TRA1 module through TAF12-SPT20 interaction [13,14]. In *Arabidopsis*, TRA1 has two homologues (TRA1a and TRA1b). In yeast, Tra1 recruits SAGA to promoters through the interaction with transcription factors [16]. In *Arabidopsis*, TRA1a and TRA1b promote H2A.Z deposition at the whole-genome level as part of the activity of SWR1 complex [18]. Finally, the DUB module (DUBm) is partially present in *Arabidopsis* genome, consisting of the UBP22 protein that deubiquitinates histone H2B, a second enzymatic activity of the complex, and the associated proteins SGF11 and ENY2. The fourth subunit of yeast DUBm, SGF73, is absent in *Arabidopsis*, suggesting that DUBm may function as H2Bub1 deubiquitinase independent from SAGA complex [19,20].

In multi-cellular eukaryotes, SAGA (or GCN5-containing) complexes appear to have an essential role in development [21]. Likewise, in *Arabidopsis*, *gcn5* mutants have pleiotropic effects on every development aspect (Table 1) [22,23]. Furthermore, mutations in another HATm subunit ADA2b, result in pleiotropic phenotypes on every part of the whole plant life cycle; however, some are different from *gcn5* mutants [22,24]. Moreover, both *gcn5* and *ada2b* mutants are implicated in plant responses to abiotic and biotic stress [22,25,26,27]. The other components of HATm in *Arabidopsis* do not affect plant development; however, the role of ADA2a on plant development is made redundant by the ADA2b function, since the *Arabidopsis* double mutant *ada2aada2b* phenocopies the *gcn5* mutation [28]. Mutation in *SGF29a* is implicated in salt stress responses by having an auxiliary role to ADA2b [29]. These genetic interactions, together with the biochemical data showing that GCN5 acts through ADA2b and ADA2a [30,31], suggest that different versions of GCN5-containing (SAGA) complexes may exist in plants.

Several subunits of the COREm, like TAF5 and TAF6a, are required for plant viability [32,33]. In addition, both TRA1 genes are also essential for plant life cycle [33]. These functions may not be specific to SAGA complex since TAF5 and TAF6 are also present in the TFIID complex, and TRA1 is also a component of the NuA4 histone acetyltransferase complex. The other members of COREm, like the SPT20 and TAF10, are implicated in environmental stresses [34,35]. TAF12b (also known as EER4 or CHK1) is involved in ethylene and cytokinin responses [36,37]. Finally, the DUBm components in *Arabidopsis* are not crucial for proper plant development [20,38].

**Table 1:** Comparison between *Arabidopsis* and known yeast SAGA subunits.

SAGA Modules	Yeast	*Arabidopsis thaliana*	*Arabidopsis* Mutant Phenotype
HATm	GCN5	GCN5 (AT3G54610, HAG1)	Pleiotropic effects on development and responses to stress [22,23,25,26]
	ADA2	ADA2b (AT4G16420, PRZ1)	Pleiotropic effects on development and responses to stress [22,24,27]
		ADA2a (AT3G07740)	No developmental abnormalities [28]
	ADA3	ADA3a (AT2G19390)	Involved in flowering (Vlachonasios, under review)
		ADA3b (AT4G29790)	No developmental abnormalities [39]
	SGF29	SGF29a (AT3G27460)	No developmental abnormalities [29]
		SGF29b (AT5G40550)	No developmental abnormalities [29]
COREm	ADA1	ADA1a (AT2G24530)	Not available
		ADA1b (AT4G31440)	Not available
	SPT3	TAF13 (AT1G026280)	Seed development [40]
	SPT7	HAF1 (AT1G32750, HAC13, TAF1)	Light responses [41]
	SPT8	Not detected	
	SPT20	SPT20 (AT1G72390)	Late flowering [34]
	TAF5	TAF5 (AT5G25150)	Lethal [31]
	TAF6	TAF6a (AT1G04950)	Lethal [32]
		TAF6b (AT1G54360)	
	TAF9	TAF9 (AT1G54140)	Not available
	TAF10	TAF10 (AT4G31720)	Involved in osmotic stress [35]
	TAF12	TAF12a (AT3G10070)	
		TAF12b (AT1G17440, EER4, CKH1)	Involved in ethylene and cytokinin responses [36,37]
TRA1m	TRA1	TRA1a (AT2G17930)	Early flowering [33]
		TRA1b (AT4G36080)	No developmental abnormalities [33]
DUBm	SGF73	Not detected	
	SGF11	SGF11 (AT5G58575)	No developmental abnormalities [38]
	UBP8	UBP22 (AT5G10790)	No developmental abnormalities [20]
	SUS1	SUS1 (AT3G27100, ENY2)	No developmental abnormalities [38]

## 3. Origin of Plant SAGA Complexes

**Figure 2:**
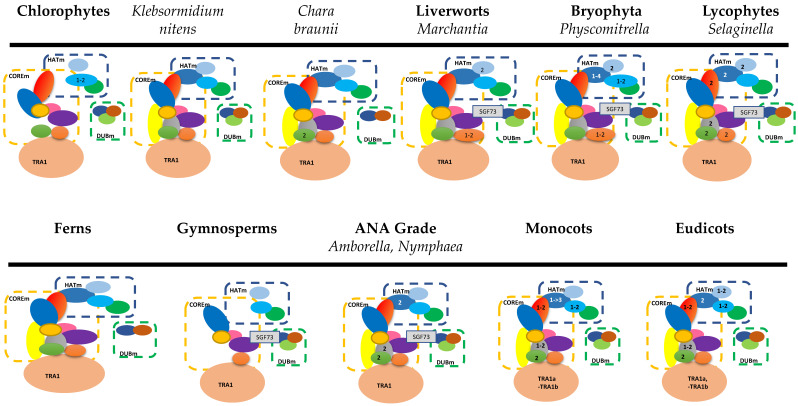
SAGA component proteins in Viridiplantae. HATm is the HAT module, COREm is the CORE module, and DUBm is the DUB module. The numbers indicate duplicated copies.

The first comparison between eukaryotic genomes and the SAGA complex revealed that yeast SAGA has dynamically diverged during eukaryotic evolution [42]. This section will cover the current knowledge of plant SAGA complex from the analysis of more than 1000 plant genomes [43]. Green plants (Viridiplantae) encompass different anatomy organisms, including the single-celled algae-like *Ostreococcus tauri*, multi-cellular green seaweeds such as the *Ulva* species, fresh-water algae with complex morphology like the *Chara* species, mosses, ferns, crop plants, and giant trees. Viridiplantae can be divided into two lineages, Chlorophyta that contains most green algae, and Streptophyta, which includes the embryophytes and the closest algae relatives, known as streptophyte and charophyte algae [44]. We used *Arabidopsis* proteins to identify homologues in plant genomes publicly available from the National Center for Biotechnology Information (NCBI). We found that the four SAGA modules are present in Chlorophyta (Figure 2; Supplementary Table S1; Supplementary Dataset 1). In approximately 20 genomes of Chlorophyta, most plant SAGA subunits are present in one copy, suggesting that early-diverging plants have the capacity to acetylate histones via GCN5; however, four subunits, including ADA3, ADA1, SPT20, and SGF73, were not detected (Figure 2). Specifically, GCN5 and SGF29 are present in one copy, whereas double copies of ADA2 are present in *Ostreococcus lucimarinus* (chromosomes 13 and 21 [45,46]), and in *Chlamydomonas eustigma*. In the other Chlorophytes, ADA2 is present in one copy (Supplementary Table S2).

Interestingly, SGF29 is only present in the genera of the monophyletic group of order Mamiellales, such as *Micromonas*, *Ostreococcus*, and *Bathycoccus* [47] and in the genus *Chloropicon* that is included in a late-diverging lineage of picoprassinophytes, named Chloropicophyceae [48]. Streptophyta comprises charophyte algae and all land plants. Proteins that constitute the GCN5-containing complexes are also present in charophytes such as *Klebsormidium nitens* and *Chara braunii* (Figure 2). The gain from the Chlorophyta SAGA-like complex is the presence of one copy of ADA3, ADA1, and SPT20 subunits. The presence of ADA1 and SPT20 could form the first complete COREm that will bind to TBP.

In contrast, the presence of ADA3 indicates the first complete HATm in plants, suggesting that the GCN5-containing complex was necessary for the early transition step from aquatic algae to land plants. The whole GCN5-containing complex is found in the early-divergent lineage Embryophytes, including liverworts and mosses. Bryophytes are characterized by the lack of vascular tissues and root systems. Still, they possess many features of land plants, including a multi-cellular diploid sporophyte, gametophytic and sporophytic shoot apical meristems with an apical cell producing three-dimensional tissues and cell fate specializations, providing morphological and physiological terrestrial adaptations [49]. In the liverwort *Marchantia polymorpha* genome [49], we detect all the SAGA module components, including the SGF73 homologue of DUBm. Horizontal gene transfer was found in the *M. polymorpha* genome since most of the genes are often homologous with fungal genes [49]. Despite the lack of ancient polyploidy in *M. polymorpha,* two paralogs of SGF29 were present (Figure 2). In the moss *Physcomitrella patens* genome [50], we observed, for the first time, the duplication of ADA2, whereas four genes encode for the ADA3 subunit (Pp3c23_22200, Pp3c20_610, Pp3c_790 and Pp3c15_5990). These suggest that in bryophytes, HATm could exist in several forms to help them cope with terrestrial environments, including enhanced tolerance to desiccation and freezing, osmotic stress, heat, and to promote the accumulation of molecules that protect plants from UV radiation. It is also evident that the *P. patens* genome contains two copies of TAF13 (Spt3) that probably arise from genome duplication. TAF13 is thought to have originated from a duplication of an ancestral SPT3 gene followed by a gene split [51]. Interestingly, the duplication of ADA2 and ADA3 is not evidenced in *Ceratodon purpureus* [52]. The duplication of the SPT3 gene is also evident in liverwort *M. polymorpha* subsp. *ruderalis* and in *Selaginella* genomes (Figure 2). In the genome of the lycophyte *Selaginella moellendorffii* that represents the earliest evolutionary branch of vascular plants [53], all the components of the plant SAGA complex were detected (Figure 2). The differences between *Selaginella* and *Physcomitrella* GCN5-containing complex are the two orthologs of SmADA1, SmTAF6, and SmTAF12, the losses of two ADA3 orthologs, and one ADA2 subunit. The *Selaginella* GCN5-containing complex differs from the *Arabidopsis* complex in the number of ADA2, TRA1, and SPT3 subunits and the absence of the SGF73 protein. In ferns, particularly in the *Adiantum capillus-veneris* genome, the components of SAGA complex exist in one copy; however, two components of the DUBm, SGF73, and SUS1 were not detected. In conifers and mainly Sitka spruce *Picea sitchensis*, several GCN5-containing complex components are detected. Still, the lack of a relevant reference genome sequence makes comparative genomics to angiosperms challenging (Figure 2).

In the first angiosperm and the most basal extant flowering plant genome of *Amborella trichopoda* [54] as well as in the nonwoody aquatic *Nymphaea* species [55], all the components of the SAGA complex were observed, some of them in duplication (e.g., ADA3, ADA1, and TAF12). Moving higher in the angiosperms, SGF73 subunit was not detected in the monocots and eudicots, suggesting that this gene was lost during plant evolution. The other components of the plant SAGA complex were observed either in one or two copies. In monocots, ADA2 is detected in one copy, or two copies in polyploidy species such as *Triticum aestivum*, *Triticum turgidum, Ananas*, *Elaeis,* and *Phoenix.* ADA3 is presented in one or two copies; however, it existed in more than three copies in the same polyploid species. The HATm in monocots also contains one copy of GCN5 and SGF29. In flowering plants (eudicots), ADA2 is represented with two orthologs, suggesting duplication of the ADA2 gene. However, in plants of the clade Campanuliids, including *Daucus carota* and members of the Asteraceae family, ADA2 is observed only in one copy, suggesting that either the duplication of ADA2 did not take place in all eudicots or the second copy was subsequently lost (Supplementary Figure S1). Furthermore, GCN5 is present with one copy, whereas ADA3 and SGF29 are detected in two copies in the majority of eudicots.

## 4. The Biological Role of GCN5 and ADA2b in Plants

In *Arabidopsis*, GCN5 and ADA2b are required for many developmental processes such as leaf development, apical dominance, root meristem activity, inflorescence, floral meristem function, and flower fertility [22,23,24,56,57,58,59]. Mutations in *gcn5* and *ada2b* genes affect the expression of many genes [22], highlighting the effect on several developmental processes and plant responses to environmental cues. GCN5 is shown to acetylate lysine 14 of histone 3 (H3K14ac) and influence H3K9 and H3K27 acetylation in the promoter and at both 5’ and 3’ UTR of its target genes [26,27,60]. During embryogenesis, GCN5 suppresses *TOPLESS* (*TPL*) embryonic activity indicating that the polarity during embryogenesis is mediated by TPL and GCN5 genetic interaction [61]. GCN5 is also required for the establishment of the epidermal cell patterning during root growth [62] and affects the expression of *PLETHORA* (*PLT*) transcription factors required for root meristem maintenance [57]. Furthermore, GCN5 is involved in the establishment of competence for de novo shoot regeneration. In this process, GCN5 triggers acetylation at the promoters of transcription factors *WUSCHEL-RELATED HOMEOBOX* 5 (*WOX*5), *SCARECROW* (*SCR*), PLT1, and PLT2 in root meristem [63], acting as an epigenetic switch that allows somatic cells to acquire regeneration potential for developing callus. Several transcription factors, e.g., the bZIP11-related basic leucine zipper (bZIP) transcription factors, interact via an amino-terminal activation domain with ADA2b adapter proteins to recruit the histone acetylation machinery to specific auxin-responsive genes [64]. GCN5 and ADA2b affect the developmental transition from the juvenile to adult phase by controlling the expression of SQUAMOSA PROMOTER BINDING PROTEIN-LIKE (SPL) factors [65]. GCN5 and ADA2b also affect several aspects of leaf cell growth and division, including in endoreduplication and trichome morphology [66,67].

GCN5 is required for the proper maintenance of the shoot apical meristem [56] as a negative regulator of the transcription factor WUSCHEL (WUS) [23,56,68] independent from the CLAVATA (CLV) pathway. Since histone acetylation is generally involved in transcriptional activation, it could be hypothesized that *WUS* is regulated indirectly, with GCN5 targeting one or more negative regulators of *WUS*. As a master regulator of the shoot apical meristem stem cell niche, *WUS* has a rather complicated regulation including epigenetic modification, chromatin remodeling, and hormone signaling [reviewed in 69–71]. Alternatively, a new mechanism has been recently proposed [27], where GCN5 can act as a suppressor of expression in certain genes by negatively controlling H3K14 acetylation levels at the 3′ ends of those genes.

Flower development is also a target of GCN5 and ADA2b function, in which the reproductive organs, stamens, and gynoecium are mainly affected [56,68]. The effect of GCN5 on gynoecium development is more pronounced when the CLV pathway is simultaneously involved. The double mutants of *GCN5* with *CLV1* or *CLV3* exhibit severe phenotypes, which include elongated gynoecia with enlarged stigma and style, reduced valves, and elongated gynophores [68]. Subsequent analysis showed that GCN5 and CLV signaling affect auxin biosynthesis, transport, and signaling [68]. The PIN1 auxin transporter is downregulated in *gcn5* and *clv1gcn5*, and *YUCCA4* (YUC4), a gene encoding an auxin biosynthesis enzyme, is overexpressed in *gcn5* and *clv1gcn5*. The acetylation of H3K14 in both genes at their promoter region is reduced in *clv1gcn5*, suggesting that histone acetylation could play a role in their regulation [68]. Whether *PIN1* and *YUC4* are direct targets of GCN5 remains to be determined. Furthermore, GCN5 and CLV pathway promote cytokinin signaling in the carpel meristem of developing gynoecia [68]. As a result, in many flowers of *clv1gcn5* double mutants, the gynoecia have large callus-like protrusions from their apex.

More interestingly, both GCN5 and CLV signaling repress *WUS* expression in the developing gynoecium. *WUS* expression is detected in the flower meristem up until floral stage 6, when it is suppressed mainly by agamous (AG) and other factors [69,70,71]. At floral stage 10, *WUS* expression can be detected in the stylar region. In *clv1gcn5* double mutants, *WUS* is ectopically overexpressed throughout the gynoecium center, severely affecting normal development. Chromatin immunoprecipitation experiments show that histone H3 acetylation is reduced in the promoter region of *WUS* in *clv1gcn5* but not in *gcn5*, suggesting that CLV1 and GCN5 synergistically affect H3 acetylation levels in WUS loci [68].

**Figure 3:**
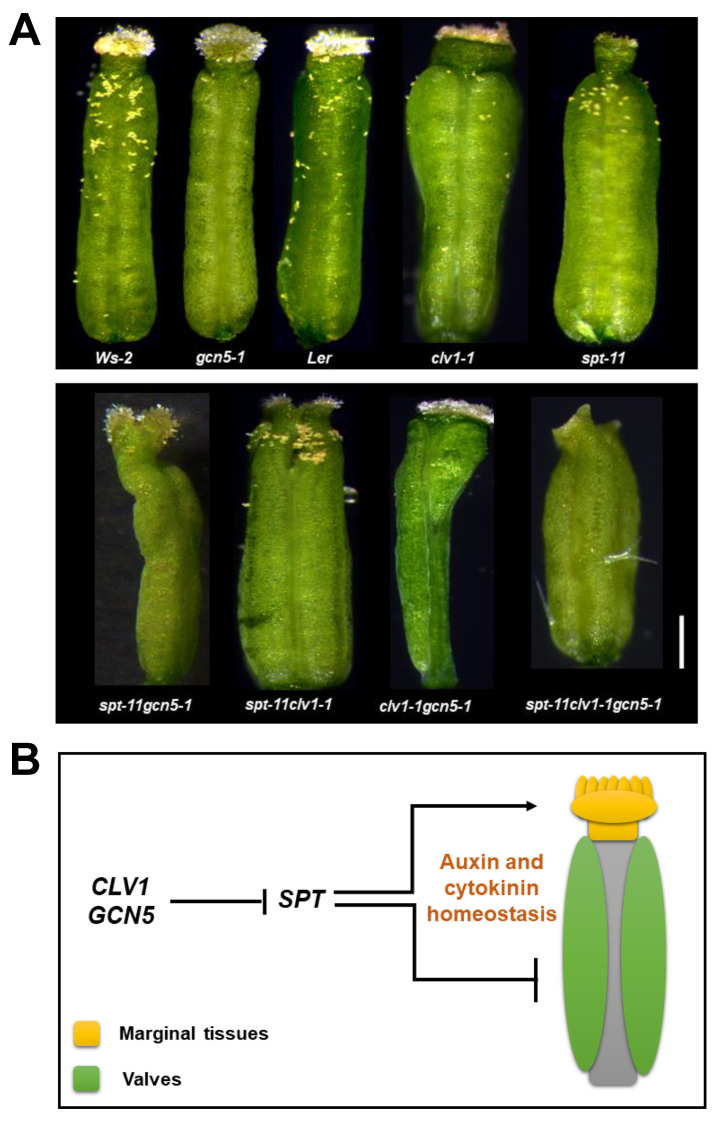
Genetic interactions between GCN5, CLV1, and SPT. (**A**) Stereoscopic images of representative gynoecia from stage 13 flowers of each genotype are shown. The white bar represents 250 μm. (**B**) A genetic model for the role of *SPT, CLV1*, and *GCN5* in gynoecium development.

Auxin-cytokinin crosstalk is important for many developmental processes in plants, including gynoecium development [72,73]. A central factor coordinating auxin and cytokinin responses in the gynoecium is the bHLH transcription factor spatula (SPT) [74]. *SPT* mutants have abnormal gynoecia: laterally expanded, unfused at the apex with reduced stigmatic and stylar tissues, and no transmitting tract [75]. We found that *spt* mutations suppress most of the abnormal phenotypes of *clv1gcn5* gynoecia, including enlarged stigma and style, reduced valves, and callus-like protrusions at the apex (Figure 3A). The genetic interaction between *spt* with *gcn5* or *clv1* alone is mostly additive. These results suggest that SPT mediates the interaction of GCN5 and CLV signaling during gynoecium development, presumably by modulating auxin and cytokinin homeostasis (Figure 3B) [74]. Further biochemical work is needed to elucidate if *SPT* is a direct target of GCN5/ADA2b and if SPT recruits GCN5/ADA2b to target genes by interacting with SAGA subunits, or the interaction of SPT, CLV1, and GCN5 involves another molecular mechanism.

GCN5 and ADA2b participate in integrating diverse internal and external signals into plant responses, including light, mineral nutrient signaling, and abiotic stress [reviewed in 19]. In light-responsive gene expression, GCN5 acts as a positive regulator; *gcn5* mutants have reduced expression of light-responsive genes like *CAB2* and *RBCS1A*, and H3 acetylation, especially for H3K14, is reduced in the promoter regions of those genes. GCN5 was found to be enriched on those genes suggesting that they are direct targets [26]. Upon iron deficiency, GCN5 is recruited to the *FRD3* locus and this recruitment is correlated with increased H3K9/14 acetylation and gene expression of *FRD3* [76]. Similarly, under phosphate starvation GCN5 is recruited to target genes WRKY6, SBT3.5, RIPK, and At4, and promotes acetylation and upregulation of their expression [77]. The first indication that GCN5 and ADA2b are involved in abiotic stress response was described in a cold acclimation process, where the transcription factor CBF1 recruits ADA2b and GCN5 to cold-regulated gene promoters [22,78,79]. ADA2b, but not GCN5, loss of function mutant was freezing tolerant [22] independent of CBF1 function. This phenotype suggests that ADA2b could have a distinct role from GCN5 in abiotic stress responses, including drought and ABA responses [25]. Furthermore, *ada2b* mutants have small cell size and affect endoreduplication independently of GCN5 [66]. ADA2b impinges on the transition between cell proliferation and differentiation and ADA2b may mediate an endoreduplication-dependent mechanism for cell morphogenesis [66]. Moreover, ADA2b is involved in DNA repair mechanisms by interacting with SMC5 protein [80]. Finally, some of the genes overexpressed in *ADA2b*, such as for the transcription factors *ERF5*, *ERF6*, *WRKY33*, *WRKY53*, *ZAT10, ZAT12*, could activate downstream genes leading to freezing tolerance [22]. GCN5 is also involved in heat-stress responses; GCN5 targets the heat-stress response genes *HSFA3* and *UVH6* and induces their expression, increasing RNA polymerase II engagement and H3K9/14 acetylation levels [81]. Interestingly, *GCN5* expression is also induced by different environmental stresses including iron deficiency [76], salt [82], heat [81], and phosphate starvation [77], suggesting that GCN5 could be a target of several stress inducible transcription factors or other chromatin related factors. The mechanisms of transcription regulation of GCN5 are still unknown.

GCN5-mediated histone acetylation is required in the integration of hormone signals during stress responses. In poplar trees, GCN5 and ADA2b are recruited by the transcription factor AREB1 to the promoter of drought and ABA-responsive genes, resulting in increased H3K9 acetylation levels [83]. GCN5 and CLV pathways act synergistically to repress ethylene signaling [84]. GCN5 also modulates histone acetylation levels in the promoter of ethylene-responsive genes [84,85,86]. Furthermore, GCN5 is also involved in biotic stress responses [27]. GCN5 is a negative regulator of salicylic acid (SA) accumulation and SA-mediated immunity. GCN5 acetylates the loci of three negative regulators of SA biosynthesis, *MYC2*, *DND2*, and *WRKY33*, and promotes their expression. In *GCN5* mutants, the reduced acetylation of *MYC2, DND2*, and *WRKY33* results in reduced expression of the genes and increased biosynthesis of SA [27]. In soybean, the cytoplasmic effector produced by the pathogen *Phytophthora sojae*, PsAvh23, suppresses H3K9ac mediated by ADA2 and GCN5 and increases plant susceptibility [87]. These results indicate that GCN5-containing complex in plants acts as a significant regulator of responses to internal and external stimuli. Determining the direct targets of SAGA modules across plants will be essential to determine the primary role of SAGA activity during plant evolution. Therefore, plant SAGA complex activity, as an indicator of diverse physiological processes mediating adaptation to the environment, remains to be deciphered in future studies. 

## Data Availability

The corresponding author K.V. is responsible for distribution of the materials generated in this study in accordance with the policy described in MDPI Research Data Policies” at https://www.mdpi.com/ethics.

## Supplementary Materials

The following are available online at https://www.mdpi.com/2223-7747/10/2/308/s1, Figure S1: ADA2 proteins in Viridiplantae, NJ-tree analysis. Table S1. List of known SAGA component proteins in Viridiplantae. Table S2. Members of the HATm in Chlorophyta. Supplementary Dataset 1: Accession number of proteins of the plant HATm.
