# Supplementary material for: The Histone Acetyltransferase GCN5 and the Associated Coactivators ADA2: From Evolution of the SAGA Complex to the Biological Roles in Plants
Source: Plants (Basel). 2021 Feb 5;10(2):308. doi: 10.3390/plants10020308 (PMC7915528; doi:10.3390/plants10020308)
Supplement: Supplementary file 1 [file plants-10-00308-s001.zip › plants-1083604-supplementary.v2-proof/2021-01-23-Supplementary Table 1&2.docx]

**Supplementary Table 1.** List of known SAGA component proteins in Viridiplantae (nd= not detected, yellow=HAT module, orange=CORE module, green=DUB module).

| SAGA components |  | Viridiplantae | | | | | | | | | | |
| --- | --- | --- | --- | --- | --- | --- | --- | --- | --- | --- | --- | --- |
|  | Chlorophytes | Streptophyta | | | | | | | | | | |
|  |  | *Klebsormidium nitens* | Streptophytina | | | | | | | | | |
|  |  |  | *Chara braunii* | Embryophyta | | | | | | | |  |
|  |  |  |  | Marchatiophyta | Bryophyta | Tracheophyta | | | | | |  |
|  |  |  |  | Liverworts | Mosses | Lycophytes | Ferns | Gymnosperms | ANA grade | Monocots | Eudicots |  |
| GCN5 | 1 | 1 | 1 | 1 | 1 | 1 | 1 | 1 | 1 | 1 | 1 |  |
| ADA2 | 1-2 | 1 | 1 | 1 | 1-2 | 1 | 1 | 1 | 1 | 1-2 | 1-2 |  |
| ADA3 | nd | 1 | 1 | 1 | 1-4 | 2 | 1 | nd | 2 | 1->3 | 2 |  |
| SGF29 | 1 | 1 | 1 | 2 | 2 | 2 | 1 | 1 | 1 | 1 | 1-2 |  |
| ADA1 | nd | 1 | 1 | 1 | 1 | 2 | 1 | nd | 2 | 1-2 | 1-2 |  |
| SPT20 | nd | 1 | 1 | 1 | 1 | 1 | 1 | nd | 1 | 1 | 1 |  |
| SPT3* | 1 | 1 | 1 | 1-2 | 1-2 | 2 | 1 | 1 | 1 | 1 | 1 |  |
| SPT7** | 1 | 1 | 1 | 1 | 1 | 1 | 1 | 1 | 1 | 1 | 1 |  |
| TAF5 | 1 | 1 | 1 | 1 | 1 | 1 | 1 | 1 | 1 | 1 | 1 |  |
| TAF6 | 1 | 1 | 1 | 1 | 1 | 2 | 1 | nd | 1 | 1-2 | 1-2 |  |
| TAF9 | 1 | 1 | 1 | 1 | 1 | 1 | 1 | 1 | 1 | 1 | 1 |  |
| TAF10 | 1 | 1 | 1 | 1 | 1 | 1 | 1 | 1 | 1 | 1 | 1 |  |
| TAF12 | 1 | 1 | 2 | 1 | 1 | 2 | 1 | nd | 2 | 2 | 2 |  |
| TRA1 | 1 | 1 | 1 | 1 | 1 | 1 | 1 | 1 | 1 | 1-2 | 1-2 |  |
| SGF73 | nd | nd | nd | 1 | 1 | 1 | nd | 1 | 1 | nd | nd |  |
| SGF11 | 1 | 1 | 1 | 1 | 1 | 1 | 1 | 1 | 1 | 1 | 1 |  |
| UBP22 | 1 | 1 | 1 | 1 | 1 | 1 | 1 | 1 | 1 | 1 | 1 |  |
| SUS1 | 1 | 1 | nd | 1 | 1 | 1 | nd | 1 | 1 | 1 | 1 |  |

**Supplementary Table 2.** Members of the HATm in Chlorophyta (nd = not detected).

| Chlorophyta | GCN5 | ADA2 | SGF29 |
| --- | --- | --- | --- |
| *Micromonas commoda* | 1 | 1 | 1 |
| *M. pussila* | 1 | 1 | 1 |
| *Ostreococcus lucimarinus* | 1 | 2 | 1 |
| *O.tauri* | 1 | 2 | 1 |
| *Bathycoccus prasinos* | 1 | 1 | 1 |
| *Chroropicon primus* | 1 | 1 | 1 |
| *Coccomyxa subellipsoidea* | 1 | 1 | nd |
| *Trebouxia sp.* | 1 | nd |  |
| *Auxenochlorella protothecoides* | 1 | 1 |  |
| *Helicosporidium sp.* | 1 | 1 |  |
| *Micractinium conductrix* | 1 | 1 |  |
| *Chlorella sorokiniana* | 1 | 1 |  |
| *C. variabilis* | 1 | 1 |  |
| *Volvox carteri f. nagariensis* | 1 | 1 |  |
| *Gonium pectorale* | 1 | 1 |  |
| *Dunaliella salina* | 1 | 1 |  |
| *Chlamydomonas eustigma* | 1 | 2 |  |
| *C. reinhardtii* | 1 | 1 |  |
| *Haematococcus lacustris* | 1 | 1 |  |
| *Tetrabaena socialis* | 1 | 1 |  |
| *Monoraphidium neglectum* | nd | 1 |  |
| *Raphidocelis subcapitata* | 1 | 1 |  |
| *Scenedesmus sp.* | 1 | 1 |  |
