# Supplementary material for: The Histone Acetyltransferase GCN5 and the Associated Coactivators ADA2: From Evolution of the SAGA Complex to the Biological Roles in Plants
Source: Plants (Basel). 2021 Feb 5;10(2):308. doi: 10.3390/plants10020308 (PMC7915528; doi:10.3390/plants10020308)
Supplement: Supplementary file 1 [file plants-10-00308-s001.zip › plants-1083604-supplementary.v2-proof/Figure S1-ada2-viridiplantae.docx]

ADA2-VIRIDIPLANTAE

>XP_011625124.1 transcriptional adapter ADA2 isoform X1 [Amborella trichopoda]

MGRSRAAVNAGEDDPNQRSKRRRVASSGENIEAGPTGTTEGKRALYHCNYCNKDISGKIR

IKCTKCPDFDLCVECFSVGAEVTPHKSNHPYRVMDNLSFPLICSDWNADEEILLLEGIEM

YGLGNWAEVAEHVGTKSKAQCIDHHTIAYINSPCYPLPDMSRVNGKNRKELLAMAKVQTE

GKKGFSAPGEILPKEESPFSPTRLKVEEAPTGRSPSNLTADGEFSESKINGVGRNSVIGA

SAGAGKKASNMLQLKDGSDGIKIENLASVEDSHMDRSLGGKKPKVIGDKGPSMTELSGYN

PKRQEFDPEYDNDAEQPLAEMEFKDNDTETDRELKLRVLRIYLSRLDERKRRKDFILERN

LLYPNPLEKELSKEDTEIYHRCKVFMRFHSQEEHDALIRGFIQERKIRQRIQELQEYRAV

GCHTLAEGERYAAEKRRKESESNIKKAKEGGHLGASGKVLQKVNRPMMKEKPESDGSPGS

TVDNQKTKGPMAMDSCVNNLSSTRAMDQWDITGLPGANLLSSSEQQLCCQNRLLPAHYLK

MKEVLTLEALKGTTVEKSDAYRFFKVDPSKVDKVYDLVTKINCVKGEEFNQCVKVEP

>XP_001420946.1 predicted protein [Ostreococcus lucimarinus CCE9901]

MASALVPKRRRVATENAMTKLSGNGESCALFNCNYCQKDISNVVRVRCAECANVDLCTEC

FAVGVEPHPHKAYHQYHVIDNMSFPLFTRDWGADEELLLLEAVEMFGLGNWTEVSEHVGT

KTRAQCHAHYFEVYVKSPCAPLPDMSKILGKGVARMTSDELKAEAEQKANENKDVEEEEK

LLESLANPNAVKTEGNVQELTGYNIKRNEFDPEYDMDAELPLAEMEFRENDTEEDVQMKL

RMIEIYNSRLQERARRKQFILERNLLNVKKQQNVEKKRSQYERDLHGTMRIFARFLTSTE

YDVLLEGLAAEHRIRTRITELKEYRRNGIHTIAEGEDYDLEKRRRETEFARLHAIEHPTS

KNIARANNLGPRRRMYLSLDLADLPGVDLLNDDEKELCRSCRLLPVQYLSMKVELMREGL

KSEKPLNRNHVRNMFKVDPLKAIRVYELLLQHGWVLEDGFVNPGEDEDSEPAPKKSASAD

EEEDEEDDEVDYETDDNDEDEDEEDDEEEDSEEDD

>XP_005647424.1 hypothetical protein COCSUDRAFT_1634, partial [Coccomyxa subellipsoidea C-169]

ALYHCNNCQKDISNTVRIKCAVCSDFDLCLECFSVGVQIHPHRNDHAYRVVDNLSFPLFH

PDWGADEELLILEGVDMFGLGNWAAVAEHVGTKGAADCQQHYTSVYINSPAFPEPTPLAS

MANVNQLQSPMQHVYRSASQRGRANGPPNEFPLMLFAQVGPNQSELTGYNAKRHEFDPEF

DQDAELLIAELEFQEEDSPEERAEKVRLVEVYNARLSGREERRAFIRDRGLLNVKRMQGA

ERRRTAYEREFHARLRPLARYQPQPDHEVFVEGLLLEARLRARLLELKEMRRAGVRTFTE

AEVYEADRKRRALEERGARGDRLHARHPLRSDADDAAILAQLAAWRARRGVALDITALPG

LDALSAKERELCASVRMLPGQYLSVKAAMLREAARRDGQHLPRSEARTMFRLEPSRALRV

YDLLAAAGWL

>XP_003082628.2 Zinc finger, ZZ-type [Ostreococcus tauri]

MASALAPKRRRVATENALTKLSGGEECALFNCAYCQKNISNVVRIRCAVCSNFELCVECF

SVGAERQQHKAYHDYHVIDNMSFPLFTRDWGADEELLLLEAVEMFGLGNWTEVSEHVGTK

TKTQCHAHYFEVYVKSPSAPLPDMSKILGKGVPRMTEEELKAELEQQANENKDKADEERA

VLESLANPNAVKTEGNVQELTGYNVKRNEFDPEYDMDAELPLAEMEFRENDTEEDVQMKL

RMIEIYNSRLQERARRKQFILERNLLNVKKQQNLEKKRSQYERDLHGTMRVFARFLSPTD

YEMLLEGLAAEHRLRSRITELKEWRRNGIHTIAEGEDYDLEKRRRETEFARLRAIEHPTS

KNIARANKFIIRDATQINEQLARIADEEKTSAIPTPRTPSTGTRRRMYLALDLTDLPGVD

LLSEDEKELCTSCRLLPVHYLAMKLELMREGLKSETPLTRNRVRTMFKIDPLKAVRVYEL

LLQHGWVLEDGFTNPDETEDAEPASKKSKRDSDADEEDMEGDENGPDDDEEKSEDDNDED

EEDEDKSEEEE

>XP_001422948.1 predicted protein [Ostreococcus lucimarinus CCE9901]

MASALVPKRRRVATENAMTKLSGNGESCALFNCNYCQKDISNVVRVRCAECANVDLCTEC

FAVGVEPHPHKAYHQYHVIDNMSFPLFTRDWGADEELLLLEAVEMFGLGNWTEVSEHVGT

KTRAQCHAHYFEVYVKSPCAPLPDMSKILGKGVARMTSDELKAEAEQKANENKDVEEEEK

LLESLANPNAVKTEGNVQELTGYNIKRNEFDPEYDMDAELPLAEMEFRENDTEEDVQMKL

RMIEIYNSRLQERARRKQFILERNLLNVKKQQNVEKKRSQYERDLHGTMRIFARFLTSTE

YDVLLEGLAAEHRIRTRITELKEYRRNGIHTIAEGEDYDLEKRRRETEFARLHAIEHPTS

KNIARANKFIVRDATQINEQLTRMNDEDKTVSVIPTPRTSSLGPRRRMYLSLDLADLPGV

DLLNDDEKELCRSCRLLPVQYLSMKVELMREGLKSEKPLNRNHVRNMFKVDPLKAIRVYE

LLLQHGWVLEDGFVNPGEDEDSEPAPKKSASADEEEDEEDDEVDYETDDNDEDEDEEDDE

EEDSEEDD

>XP_002502862.1 histone acetyltransferase complex component [Micromonas commoda]

MATENAITKVNPSEPKRAMFHCHYCSKDISAVVRIKCAVCADCTLCVECFSVGVEPHPHE

ASHAYHVIDNLSFPLFTMDWGADEEILLLEAIEIYGLGNWTEVAEHVGTKTKLQCHQHYF

DCYVNSETTPLPDMSKILGPKYTKEEPNEEPKAKKVKAEDGDADDDAAERADTLASFARP

GDERHWGNVPELTGYNVKRNEFDPEYDIEAELPLAEMEFRDTDTELDRKLKIRMLEIYNK

RLEERIRRKEFIIDRGLLNVKRQQALERKRTPQERDIHGAVRVFARFLDPNEYEIMLEGF

MAESRIRNRIAELKEYRRNGIHTLSEGEVYDAEKRHRMAEIARIKAIEYPGRGGSRANRY

LGRDGFVQAPAGDAAPKELQKLTGIAAGGGGSGALTSLGGTARKKAPLPLDLTHLPGVEL

LSKREKELCVANRLLPVHYLSIKEALMRASANGQVLKRSEVRHMFKVEPIKAVRVFELLL

QHGWVKDPNETEG

>OUS46261.1 hypothetical protein BE221DRAFT_95636, partial [Ostreococcus tauri]

MASALAPKRRRVATENALTKLSGGEECALFNCAYCQKNISNVVRIRCAVCSNFELCVECF

SVGAERQQHKAYHDYHVIDNMSFPLFTRDWGADEELLLLEAVEMFGLGNWTEVSEHVGTK

TKTQCHAHYFEVYVKSPSAPLPDMSKILGKGVPRMTEEELKAELEQQANENKDKADEERA

VLESLANPNAVKTEGNVQELTGYNVKRNEFDPEYDMDAELPLAEMEFRENDTEEDVQMKL

RMIEIYNSRLQERARRKQFILERNLLNVKKQQNLEKKRSQYERDLHGTMRVFARFLSPMD

YEMLLEGLAAEHRLRSRITELKEWRRNGIHTIAEGEDYDLEKRRRETEFARLRAIEHPTS

KNIARANKFIIRDATQINEQLARIADEEKTSAIPTPRTPSTGTRRRMYLALDLTDLPGVD

LLSEDEKELCTSCRLLPVHYLAMKLELMREGLKSETPLTRNRVRTM

>XP_007508385.1 predicted protein [Bathycoccus prasinos]

MKNGNQNQSSMTTMNTTMKNKRRRTATGTAITKAQNLSNAQVALFHCSYCQKDISSVVRM

KCASCVGVDLCVECFAVGAEPFPHKAGHPYHVIDDLSFPLLTLDWGADEELLLLEGVEIF

GLSNWTDVSEHVGTKTKSQCQQHYVEEYVKSPAAPLPDMSKVLGKGYKKLTEEDQAELRR

KQKQKSKLKEENETNGGGEENNNNNKENGTLNQDVEMTLLQQMSKPGEIRAEGNISELTG

YNVKRNEFDPEYDIEAELPLAEMEFRELDTEEDRKLKIRMIEIYNERLAERQRRKNFILE

RGLLNVKKQQMFEKKRSQYERDLHGTLRVFMRYLSQSEYDVLLEGLAAENKIRTRIGELK

EYRRNGITTLQEGENYDVEKKRRMEEFARLKSFESPHSRKKGFTPLPLAQAQPGDANKSS

NTIFPSPGGRSLKTDHHLGNTSGSKKRMYIPLDLATLPGVELLSKQEKELCVTNRMLPVQ

FLMVKQQLMKLSQERGKSNPIKRAEVRTMFKIEPIKVLRVYELLCQSGYVISGDEKKVQE

EEEEKEEDEEEEEEEDEEEEEEDEEEEEEEEEEEEEEEEEDDDDEDASVEEEESEEEEEE

ESEEEESDS

>XP_003060975.1 histone acetyltransferase complex component [Micromonas pusilla CCMP1545]

MGGPGGGPSGFGAGGFHQQPKRRRCPTENAMTKVNPSEPKRALFHCNYCQKDISNVVRIK

CAECAEMDLCAECFSVGVEPHPHKACHPYHVIDNISFPLFTMDWGADEEVLLLEAIEIYG

LGNWTEVAEHVGGAKSKMQCHDHYFETYVNSPTTPLPDMKRLLGKDYVKEEPKDAEEGKK

RGKTDPEDAERERTLASFSRPGDERHWGNAQDLTGYNVKRDEFDPEYDVEAELPLAEMEF

RDTDTELDRKLKLRMIEIYNRRLEERRKRKQFIIDRGLLNVKRQQALERKRTPQERDIHA

AVRVFARFLEPNEYEIMLEGLAAESRIRNRIAELKEYRRAGCRTIREGEQYDSDKRARIA

EHARIRAAETPAKGAAAAAAARANKYLSRDGFVAAPPGAPGGEPPKPGAGAGASSGGGGG

GRRKAPLPLDLAKLPGVELLSKREQDLCTHNRLLPVQYLAMKEAMMRASADGEPLRRADV

RYKFAVEPVKATRTYELLLSNGWITDPNGGKDAEKEQDDE

>XP_002952656.1 hypothetical protein VOLCADRAFT_62830, partial [Volvox carteri f. nagariensis]

YHCNYCQKDISHVPRIKCAECKDFDLCLECFSPHKNTHDYQVVENLSFPIYHPDWGADEE

ILLLEAIDIYGLGNWPGVSEHVGGKNPAQCRQHYFAVYIDHDMMPLPRPTPEMAQLHKKE

VCPWQSLLTRIFPPPHIPFPSARPLLPPRGGSYDATGFHPKRMEFDPEYDNDAECIVADM

EFSEYDNPADVQLKLQMLMLYNRRLDERERRRAFVLERSLLNTRAAQAVERRRNTQERDL

YARMRVFARYQSQSSHDELVEGLLLEARMRTRIAELREYRRNGIRTFVDAEVYDTEKRRQ

KAAADAAVAAANAVHGQPYSGPGRGAGKARAAAAAAAAAAAAAAAAAAGGGMMQPGYGTP

LGDDGLQLALAAKRGVPLDITCMPGVELLGSRERELCAAQRLLPCHYLALKDMLLRDCEK

NGAITKADVSGSPFLPSSLSHFSPLLSPSPFLPPSSVCVWRYSTRHIYVVG

>QDZ24601.1 transcriptional adapter 2 [Chloropicon primus]

MEEGSTGRHYRTKRRRALDGGLLTQPTRGSFSGAGKKKALYNCKYCGKDLSHTLRVRSVG

PSSGSGEEEDKGGFKGGATAGTTTDLCLECFSVGVEPWPHKRTHAYRVVEDLSVPIFDPE

WGCDEELLLLEALEIYGPGNWSEIADHVGRKDKFECRNHYFELYINSPKAPLPVVPTQEQ

LQSRVNKRKEEEAEGKSGLKVASGKQEEAEWQGGAGSDTPDQANDSAGERSVEGSPLEGK

AGKGEAQQQVDAARGEGDPNTLVFKGLSPQIRARAEGNQIEITGYNVKRDDFEPENDNDA

EVALAELEIRNEDSKEERALKLEMLRIYHVRQRERYEKRKFVLDRHFLNVRKQQLVEKRR

TREEKEIHACMRVFARFQTPDEHDELVGGICKEQRLRKRISELKEYRKMGILTLAEAEFY

EQDKRRRESERERVKQMESYLQQRAYKSSKQERANRYFNRKSSVTDQEDGNGTSVVSGPG

SSRELQGLRIEDPGKTEKIPNNLAMVTKQMEPADLTSTPYLDLLTKEEQDVCSSLRMLPV

QYLSIKTGMVSLSKSQGGLDKQDAREFFKVDVYKLNRIHDYLVSKGDITAVD

>GBF98012.1 transcriptional adapter-like [Raphidocelis subcapitata]

MSEQRSRRRTGRGGVLENGYTRAMQESKRALYHCNYCHKDIANEVRIKCADCADFDLCLD

CFAVGVEAAPHKNTHRYRVIDNLSFPLLHPDWGADEELLLLEGIDVYGFGNWAAVSEHVG

TKPAAACRDHYFAAYVDSPTFPLPSPAPEMRGADVRKLAAEARASGGRKRQRLDPQQLQA

ANAEAGGGAAPAKAAAAAAAAAAAAAAAAAAGGGDAAAAAAGAAGAGAAGSKHKRAKTAA

SGAAAAAAAAPSALAPSALGPSGKALPAGGAPGSKPGAGGWDMTGWHAKRGEFDFEYDQD

AEMAIAEMEFKEDDSPLDVAHKLKMLEIYNKRLDERERRRAFILEHGLLNVRRMQGHETR

RLPHEREQYARMRVFARYQPPGAHESLVDGLLAERRLRARIAELQAYRRAGLRTFGEVEE

AELERAAKSKPAQAAADDAAATAAGAALAAHAAAAPPTARASAHALHAWRTRRGAALDVC

GLPGTEALCARERELCAAARLLPAHYLSLKDVMMRDAEANGFITRQDARTYFRMEPSRSL

RLYDLLLSCGWLGEVPPALLAGNAAGAKAYRQILGLGDGGGGDGGGGEEEGEEEEGEEGE

EEAEGGEEASAVPTQQQQQQQHHGRRSSTANVKTEAG

>GAX82593.1 hypothetical protein CEUSTIGMA_g10019.t1 [Chlamydomonas eustigma]

MSEQTTSIGRGRRRAARESGLLKTPHFELKRALYHCNYCQKDISNVARIKCAICPDFDLC

LQCFSVGVEITPHKNYHDYRVVDNLSFPILHPEWGADEEILLLEAVDIFGLGNWVSVADH

VANKTVQQCKQHYFQTYIDTETFPYPVVAPELSLMTEEQLRLGTINAPSGSIQPSRKRLK

LDGKDQAVTPQLAESDVEQNLLLPMSAETQRIEKAEECATGTSAPVKLEDGAANTDAAAK

PSMGCKAMSHKEHAASVAASDSVAAAPAASTAASGSHSRLEGAAAIQTQGTNGTLIGHKE

GTPQLGAPVGLPWAGGKELNKSKLGFDPDGSGYHFKRHDFDPEWDNDAECVISDMEFTEL

DTEEDRKQKLRILEIYNKRLDERERRRAFLASRNLIRVKHYQSLDKRRTPQEKEMLARLR

VFARYGAAPGEHEQLVEGILLESRLRARLQELKEYRRHGIRTLADADVFETERRRSKNAD

QAAALAAKAVARSRLSIPPTPHGAAGGSTSTLSLLESVEEVTLLPTPSALAAGASKAGAA

GAPVGRSAAVALSNWRSRRGVPLDITCLPGVELLSRRERELCALSRLLPVHYLALKDLML

RDCQKHGAVSKQEARTFFRLDATRCLKLYELWTTLGWVSAGNSSNKGGAGAPHNTPLPPI

PGGALTAKRLSGSNSGLAGPSTGLHQGKSDGAAMLMSRRLHVDAPPALNLGTPGPTVGGL

VDWGFGASRPGSTQPSPSFGLDLSAFSGSADTFTAGSTFPGAPFSNLSPASQQDPSRGLM

NYAFGAQSSSSPSVLAAAAAAFIRNQRQSSGGGAVSAVGGGGAVWPTFPISMTPQQQQQM

LMLLQQQQSALQPLTQQQSNPESKEL

>PNW86501.1 hypothetical protein CHLRE_02g089150v5 [Chlamydomonas reinhardtii]

MQEQGGGRGKRRKLREGILNKNWHSQQRALYHCNNCQKDISHVPRIRCAECKDFDLCLEC

FSVGVEIKPHKNTHDYQVVENLSFPILHPDWGADEEILLLEAIDVYGLGNWLGVADHVGG

GKSAAECKRHYFQTYIDHGQMPLPVPAPEMAQVDMAQCIGRARQGYARQQYRPMQGAALL

AAGSNGPAAAAAAAAGAGGASGVLGVDGRMSSEPPGAGAASPSMDVDEPDRPADGAAAAG

AGAAGNGDVSAGEEAGPKADGAAGAGGAEAGAGGEAGGSPLANKGLKRHRHEQEAEEQAG

PKDAHGPHALVGGRSMHHEGPPAAGGKAAGGKAEAAAGPSERSAPEPSTRGEPAHAKPYN

APGTYDSTGFHAKRMEFDPEYDNDAETIVADMEFNEYDTPADVQMKVQMLQLYNRRLDER

ERRRSFLLERGLINSRAVQAPEKRRNPAERDLHARMRVFARYQPQAAHEEFVEGLVLEAR

LRSRIAELREYRRNGIRTFADADTYETEKRRQKAAADAAIAAANAVHGMPYGYGGPGRGA

GAAAAKAARAAAAAAAAGMMPPGALMSPTSLQSPPGGVGGGAFTPMGLGAPGTGGPMGMM

QAGVTDDPVAAAAAAAAAMGGNPVAAAMAAAANTAGAPSAAAAATSSDPARPLVAVPMGR

GTGTALAHWRARRGVPLDVTCLPGVELLSSRERELCAAARLLPAHYLALKDVMLRDAEKN

GAISRTEARSFFRLDPSRSLRIYDLLVAAGWVTAAPGPGGKLGAGAGGSGQLRLLAAGGE

GEGDDGAGGTEGMDLDDQGGDE

>PSC69078.1 transcriptional adapter ADA2-like isoform X1 [Micractinium conductrix]

MGAPSAAAALSRNKRRRDDAPTPGGAKAGAGKNEGLYHCDYCHKDLSSTLRIKCVVCKDF

DLCLECFSVGVQLNVTNHTSDHAYKVVQSLGFPLYHPAWRADEELLLLEGIEIYGLGNWP

KVAEHVGKSLEECRGHYLTTYIDHPGFPMPLRDASMAGLDIEQLVDEYRRSGRELVPVAQ

RLAAASPVHKKAKQGGKADGGGAAAGAAAAVAAGTVKEEVKEEPEAPVAAAGVKTEEGEE

EAGGGGGAAGKKHRSTEEAAAAGGAAPTEAHRTQGSFALRPAAPAVDVPVTPGTTGAKTP

GGGDASTAKIPAATEAQQTGYNIKRNEFEPEWDFEAETIIAELADFGPDDSPEEAAHKDR

LIQIYNRRLDERAQRRQFVLDRGLLNIKRQQAIDKRRSVAERELHGSLRVLARHLPQEQY

EALAEGIAQEQRLRARIAELQDYRAMGLRTFEQVDEVEALQEGRRRREAQQAQQQQSIRS

RIGKVAVDEGALQEALVQTMGAAHAAATLGQQHSVLPEGRGNGLTLWRNKRGVLLDITAL

PDVGPLSQRERNLCAAERYLPAQYLAIKAAILKQQELKGRVPRNDILKLPFKVDPARMQR

LHDFFVQEGWLPGGAGSGGRDGRQQQQRQSPG

>GAX86040.1 hypothetical protein CEUSTIGMA_g13455.t1 [Chlamydomonas eustigma]

MSEQTTSIGRIIDDYRVVDNLSFPILHPEWGADEEILLLEAVDIFGLGNWVSVADHVANK

TAQQCKQHYFQTYIDTETFPYPVVAPELSLMTEEQLRFGTINAPSGSIQPSRKRLKLDGK

DQAVTPQLAESDVEQNLLLPMSAETQRMEKAEECATGTSAPVKLEDGAANTDAAAKPSMG

CKAMSHKEHAASDSVAAAPAAASGSHSRLEGAAAIQTQGTNGTLIGHKEGTPQLGAPVGL

PWAGGKELNKSKLGFDPDGSGYHFKRHDFDPEWDNDAECVISDMEFTELDTEEDRKQKLR

ILEIYNKRLDERERRRAFLASRNLIRVKHYQSLDKRRTPQEKEMLARLRVFARYGAAPGE

HEQLVEGILLESRLRARLQELKEYRRHGIRTLADADVFETERRRSKNADQAAALAAKAVA

KSRLSIPPTPHGAAGGSTSTLSFLESVEEVTLLPTPSALAAGASKAGAAGAPVGRSAAVA

LSNWRSRRGVPLDITCLPGVELLSRRERELCALSRLLPVHYLALKDLMLRDCQKHGAVSK

QEARTFFRLDATRCLKLYELWTTLGWVSAGNSSNKGGAGAPHNTPLPPIPGGALTAKRLS

GSNSGLAGPSTGLHQGKSDGAAMLMSRRLHVDAPPALNLGTPGPTVGGLVDWGFGASRPG

STQPSPSFGLDLSAFSGSADTFTAGSTFPGAPFSNLSPASQQDPSRGLMNYAFGAQRSSS

PSVLAAAAAAFIRNQRQSSGGGAVSAVGGGGAVWPTFPLSMTPQQQQQMLMLLQQQQSAL

QPFTQQQSNPESKEL

>KXZ47602.1 hypothetical protein GPECTOR_34g761 [Gonium pectorale]

MALQETQGGVEIKPHRNTHDYQVVENLSFPIYHPDWGADEEVLLLEAIDQYGLGNWGGIA

EHVGGKTAAQCREHYFQVYIDHDNIPFPRPTPEMDHVNIDECIRRARQGLARSQYRGNGA

PAAPGLEVRGVSEPPGAPSPTADGAAEDGDRPADAATPAGDQAGAVEGSGAGPLGQNVAD

GEAGHGAGAASGKGLKRQRHEQQVAEEQAAAAHDGAAAPGAHVATGGRSMHHEPAQQPGG

STAGSKAPRVDSGTDKPCGSDATGFHAKRMEFDPEFDNDAECIVADMEFAETDSPQDVQL

KVQMLMLYNRRLDERERRRTLVLERGLLNTAAAQALEKKRNSQEKDLVARMRVFARYQPQ

PMHEEFVEGLLLEARLRTRIAELREYRRNGIRTFADAEVYDTEKRRQKAAADAAIAAANA

VHGQPYGGPGRGGFGAAIKGGRGAGAAGMLPQGFGPVGDDGMPPGLPPPSAAAASSSSTV

ASSAFDQSRPAVSVPMGRGAASTLAMWRAKRGVPLDITCLPGVELLSARERELCAANRLL

PAHYLALKDIMLRDCEQNGAITRQEARTFFRLDPTRSLKIYDLLVSCGWVKGSGPAKPAS

GGGERLRAITAGGELEGEEGGGGDAMDMDEQGGDE

>GAQ80026.1 histone acetyltransferase complex component [Klebsormidium nitens]

MGRSRAGQVPGKGTNLEMEDPLAALSARTKRRRGANESDSAALLHSGEARQAQYHCNYCK

KDISGCVRIKCAECPDFDLCLECFSVGAEITPHKNDHKYRVMEILDFPLLNPAWTVDEEI

LVLEGIEMFGFGNWADVADHVGTKSKGSCQEHYTSIYINSPFFPLPDMSRIAGKGRAELM

AEGKRGDVSRMSSEGPGESSQAGSLTVKLEDVKDEGISAREASVEPSIGAKSEKILLPSS

TEEERAHSHKHPSDAALASAEQGGGPGKKAAAHSHQAKPEPSAEGEQAGEDPAQSTRTLG

GKKPKLSEGEIKAGTVGAEQTGYHAKRNEFDPEYDNDAEVPLAEMEFKETDTEAERDLKI

KMLEIYNHRLDERRRRKEFILERGLLNLKKQQALDRKRTREERELYNRARVFARFTTQEE

IDELVSGLLAERRIRDRIDELKEYRAMGIRTMAEAADYESDKKKRDVEAQQRKQRESAAY

LYSSRPAQRSKRSDRAEDEPSPVGGGGKEAQKLRGNPPGTPTPTTSEAGVPSVADRVGGK

GAWKILAPLELDGHPAADLLNRLERELCEKWRLVPLHYLKMKDTLMQQSAREGLVKRSDA

VRMFKVDPIKTDAVYDLLVSKGWIEGEPSAPPRTGSAKGGSKGAASERGDAVEDGVAGSA

VHLEPDAKPMDVDGTND

>KAG0554381.1 hypothetical protein KC19_12G087000 [Ceratodon purpureus]

MGRSRGLHGSHDDDTAWFGHRSKRRRVAAGGDASEPSTTGAGETKKALYHCNYCNKDISGTIRIKCNKCPDFDLCVECFSVGVEITPHKSNHSYRVIDNLSFPLIHPEWNADEEILLLEGVEMYGLGNWGEVSEHVGTKTKTQCYDHYMTTYMNSVCSPLPDMSHVIGKSKAELLAMARSHQEGKKDGGVLRLVKQEPSISPRIKMEEGFEGRSPSSMSTGTKDIKPLLSTPGSEGDDGDGRAGGAIDSGGPNGPGLKCQKTAGGAQGAVHVKETPDSAVAGAAVEDGAQSNRTLGGKKPKPLVEDTKGGTNGTDQTGYHAKRQEFEPEYDNEAELPLADMEFKDNDHETDKELKLRMLHIYISRLDERKRRKEFILERGLLNVKRQQALDRKRTKEERELYNRCRVFMRYHSMEEHEALLNGLIAERKLRQRIEELQEYRMAGCHTLAEAEIYGSEKKKRETEANLRKARESASYLYSGKSSTHRANRYLNREKDGEAASSGSIRETPKGAVGRPLLPSGSSLLAGSVGKGSKRSGVLLDLAGCPGVDLLSHTEQDLCAQYRLLPAHYLKMKEVLMLESLKAGQVRRSDAFQMFKVDPLKTDRVYELLLSRGWIQGDGPTAPASDR

>XP_024361816.1 transcriptional adapter ADA2-like isoform X1 [Physcomitrium patens]

MGRSRGVHGTHDDDTAWFGHRSKRRRVAVGGDVPEPTATGPGEAKKALYHCNYCKKDISG

TIRIKCNKCPDFDLCVECFSVGVEITPHKSNHSYRVIDNLSFPLIHPEWNADEEILLLEG

VEMYGLGNWGEVSEHVGTKTKTQCYDHYMATYMNSICSPLPDMSHVIGKSKAELLAMARS

HQEGKKDSGVLRLVKQEPSNSPSRIKLEEGFEGRSPSSMSTGTVDIKPLLSTPGSEGDDG

DVRAGGSQAIDSGGPTVGLGSKCQKTAGGAQGVVHAKETPDSVAASSAVEDGAQSNRTLG

GKKPKPLVEDNKGGTNGTDQTGYHAKRQEFEPEYDNEAEHPLADMEFKDNDHETDRELKL

RMLHIYISRLDERKRRKDFILERGLLNIKRQQALDRKRTKEERELYNRSRVFMRYHSAEE

HEALLNGLIAERKLRQRIEELQEYRMNGCHIMADAEVYCSEKKKRETEANLRKGRESTSY

LYSGKSSTHRANRYLNREKEGEAASSGGVREVTKGRVGPHLLPSGSSLLAGGGGKGSKRS

LAPLDLAGFPGVYLLSHTEQELCAQYRLLPAHYLKMKEHLMLESMKAGQVRRSDAYQMFK

VDPTKTDRVYELLLSKGWIQGDGPTVPASDR

>XP_024363191.1 transcriptional adapter ADA2-like isoform X1 [Physcomitrium patens]

MGRSRGVHSTQDDDTAWFGHRSKRRRVAVGGDVPEPSAAGAGEAKKALYHCNYCIKDISG

TIRIKCNKCPDFDLCVECFSVGVEITPHKSNHSYRVIDNLSFPLIHPEWNADEEILLLEG

VEMYGLGNWGEASEHVGTKTKTQCFGHYMTTYMNSICSPLPDMSHVIGKSKADLLAMARS

HQEGKKDGGVLRLVKQEPPNSPSRIKMEDGFEGRSPSSMSTGTLDIKPLLSTPGSEGDDG

DGRAGGSQAIDSSGPSGGPGSKCQKTAGGTQGVVHVKESPDNTAAGAAAEDGAQSNRTLG

GKKPKPLAEDNKGGITSTDQTGYHAKRQEFEPEYDNEAEHQLADMEFKDNDHETDRELKL

RMLHIYISRLDERKRRKNFILERGLLNIKRQQVLDRKRSKEERELYNRSRVFMRYHSAEE

HEALLNGLISERKLRQRIEELQVLYHAYLYVLYTKEYRMALGQTLAETQIHGSEMKKETE

LNLRNARESTSYLYNGKFSTHRVNRYLSREKEGEAAFPGSTREVPKGRVGPHLLPIGSSL

LAGSGGKSSKRTSASFELGGFPGVDLLSHTEQDLCVQHRLIPAHYLRMKEHLMLESLKSG

QVRRSDAHQMFKVDPVKTDRVYELLLSKGWIQGDGPTASASDR

>PTQ42486.1 hypothetical protein MARPO_0029s0028 [Marchantia polymorpha]

MGRSRGPLAAATDDDGGWFGHRSKRRRLAGGEIIDPASLGDGKRPPLYHCNNCHKDISGS

IRIRCAKCTDFDLCLECLSVGVEPHPHKGNHPYQVIENLAFPLIHLDWNAEEELLLLEGV

ESYGVGNWAEVAEHVGTKNKTLCYNHYMTHYMESPFSPVPDMTHVVGKTKAELLSMAKLQ

LENKKHAPSLVDPIRTIKQEPTSSPARIKIEDVKEMTEGRSPSGLSLLGSKDGKLEGDQV

EGNSGAPMSTAKKTAAGGQVRENLDVGLTVTTDDGHSNRSIGGKKPKLLAEDNKASATNP

ESSGYVAKRHEFDPEYDNDADLLLADMEFKDNDSETDHDLKVRILRIYLARLAERRRRKD

FIIERGLINLKKQAAFDRRRTKDEKELYLRTRVFLRYHTNEEHDELLAGLCAERKIRQRI

EELQEMRAAGCRILAEGETYIVEKKKKELEGSVKKAKDNTPFLQGGKLTHRSNRYMKRET

GDGEPSPSGELRSNQKPRSGGHQSISGGNGLNTASSKDTKKSGGVSDLAGYPGMESLSFT

ERELCQQWRLLPNHYLKMKEVLLGESARKGHVNRSDAYALFKIEPIKTERVYELLVSMGW

IQGDSQPAGDRFYS

>XP_024533052.1 transcriptional adapter ADA2 [Selaginella moellendorffii]

MGRSRAASAVEDEAGWQRSKRRRYAPESLETPTPGSSEAKRAAYHCNYCNKDVSGMIRIK

CAKCADFDLCIECFSVGVEISTHKSNHPYRVIDNLSFPLIHPDWNADEEILLLEGIEMYG

LGNWAEVAEHVGTKNKTRCYEHYMTEYMKSVCSPLPDMSNVAGKTKAELLALAKAYTEGK

KNLLPYPDGSTPRTVKQEATISPSRIKVEDTKTEPLERSPTRVSSGLKAGAKKEVEDTEG

QLNESLPSNPVSAGKKSAGSQVKEAPDTANGANPTLEDGGQSTRSLGNKKPKPAQDETKP

SLATTDTTGYNAKRQEFDPEYDNDAELPLAEMEFKDIDTDADRELKLQMLHIYLARLEER

KRRKDFILERGLLNVKRQQALDRKKTKEEKELIQRSRVFLRYHSSEEHEALLAGLTAEIK

IRQRIEELQEYRSAGCHTLAEGEYYAMDKRKRSAEANLRKGRDALNSKMTNRSNRAINRD

GAEPSSSGTRDMQRYRSGANNISKLPSVGVKTTKKSSSFSPLDLAGFPGIDLLSSTEQDL

CSQLRLLPAHYLKMKEVLMLENVEKGGLRRDDACHLFKVDAAKTERVFDFLWKMGWIEAS

NDILPSL

>ADE75766.1 unknown [Picea sitchensis]

MIPLNGQKGEELHQVEIIWMVELQGVEMYGLGNWAEVADHVGTKTKSQCYDHYMMAYMNS

PCYPLPDMSHVIGKTKAELLNMAKVHGEGKKGFSAYGDPILSKPPKEEPSISPLRIKVED

VNKDSSAEGRSPSTFIAEGENSENKANNLRGNGTGKKASNAVQVKEGSNGLVAGSPALAE

DPLTNRSIGGKKPKASGEDGPPLLELSGYNPKRQEFDPEYDDDAEKPLAEMEFKENDSET

DHELKLRMLRIYLSRLNERKRRKDFILERDLLHSRPLDKILSKEEKELYQRCRVFMRFHS

QEEHNALLDGLNMERRLRQRIEELQEYRAAGCHTLAEGEQYAAEKKKREAEANQKKSKES

YQMAASAKVAQRANRTTNRERGEGDGSPGGMVDNQKIKSTAGQAPVGNNTCPAATGQKGT

KKSLIQWDIMGFPGTELLSVTERQLCTQNRLLPAHYLKMKELLMLESLKGSSVKRSDAYR

FFKVDHDKVDRVYDLLSRMGWIQGEE

>XP_011625125.1 transcriptional adapter ADA2 isoform X2 [Amborella trichopoda]

MGRSRAAVNAGEDDPNQRSKRRRVASSGENIEAGPTGTTEGKRALYHCNYCNKDISGKIR

IKCTKCPDFDLCVECFSVGAEVTPHKSNHPYRVMDNLSFPLICSDWNADEEILLLEGIEM

YGLGNWAEVAEHVGTKSKAQCIDHHTIAYINSPCYPLPDMSRVNGKNRKELLAMAKVQTE

GKKGFSAPGEILPKEESPFSPTRVEEAPTGRSPSNLTADGEFSESKINGVGRNSVIGASA

GAGKKASNMLQLKDGSDGIKIENLASVEDSHMDRSLGGKKPKVIGDKGPSMTELSGYNPK

RQEFDPEYDNDAEQPLAEMEFKDNDTETDRELKLRVLRIYLSRLDERKRRKDFILERNLL

YPNPLEKELSKEDTEIYHRCKVFMRFHSQEEHDALIRGFIQERKIRQRIQELQEYRAVGC

HTLAEGERYAAEKRRKESESNIKKAKEGGHLGASGKVLQKVNRPMMKEKPESDGSPGSTV

DNQKTKGPMAMDSCVNNLSSTRAMDQWDITGLPGANLLSSSEQQLCCQNRLLPAHYLKMK

EVLTLEALKGTTVEKSDAYRFFKVDPSKVDKVYDLVTKINCVKGEEFNQCVKVEP

>XP_031492465.1 transcriptional adapter ADA2-like isoform X2 [Nymphaea colorata]

MGRSRGVPNSGDEDPHQRSKRRRVTPSGENLENNSTGTAEGKRALYHCNYCNKDISGKIR

IKCTKCPDFDLCVECFSVGAEVTPHKSNHPYRVMDNLSFPLIYPDWNADEEILLLEGIEM

YGLGNWAEVAEHVGTKTKAQCIDHYNTAYMNSPCFPLPDMSHVIGKNRKELLAMAKGHGD

EKKGFSLLGDTTSKDSPFSSPRIKTEDSGKEAQSGRLPSSLDGDPVDSKITGAGYKDPNI

TAVGAGKKSSSMSQLKDNDGVKIEGLISSEDHSERSIGGKRPKVSGDDGPSLTELSGYNP

KRQEFDPEYDNDAEKPLGEMEFKDNDSEADRELKLRVLRIYLSRLDERKRRKDFILERNL

LYPNPLEMELSNEDKELYQHWKVFMRFHSQEEHDALIQGLILERKIRQRIQELQEYRAAG

CRTLAEGERYAAEKRKRESEANMLKAKDGNQSFAGGKVLQHVNRPGSKDKKDPDGTPGID

NNKIKSNSRNESGGADAFSTTTGHIARILDQWDITGLPGAELLSMAEQQLCCQIRLLPAQ

YLKMKEVLMLEALKGSVLKKADAYELFKVEPSKVDRVYELATKMGWVQVLAVFSHEIGFN

WTEGRLLYRRDVVLAYG

>XP_019054856.1 PREDICTED: transcriptional adapter ADA2-like isoform X2 [Nelumbo nucifera]

MGRSRGVPNSADEDPGQRSKRKRVASSGENLESATTGQGTNEGKRALYHCNYCNKDISGK

IRIKCVKCPDFDLCVECFSVGAEVTPHKSNHPYRVMDNLSFPLICPDWNADEEILLLEGI

EMYGLGNWAEVAEHVGTKNKAQCIDHYSTTYMNSPCFPLPDMSHVVGKNRKELLAMAKGH

GEGKKGFPILGEQMMKEESPFSPSRVKLEDLSKEAPAGRSPSSLTTDSGIGSSNTNPSST

GAVKKASNVTQVKESPDPIKLEDSEVDRSVGGKKPKSSGDEGPSLSELSGYNPKRQEFDP

EYDNDAEQSLAEMEFKDADTEAERELKLRVLHIYLKRLDERKRRKDFILERNLLYPNPLE

KDLSPEEKNIYHRYKVFMRFLSKEEHEELMKTVIDEHRIRKRIQDLQEARAAGCHTSADL

DRYIEHKRKREAEESARRGKEGPLVGPSGKVLQRTNRPKGEADSSPRGGVKGSLGLETGG

KDLASTTLRQSAMDDWCITGLPGSDLLSETDQRLCCELQLFPSHYLKIQEIMSVEIFSGN

ITKKSDAYRFFKVEPSKLDRIYDVLAKKLIAQP

>XP_010252741.1 PREDICTED: transcriptional adapter ADA2 isoform X6 [Nelumbo nucifera]

MGRYRGVPNSADEDPGQSRSKRKKTASSGEHLDSVTTVQGTNEGKRALYHCNYCNKDISG

KIRIKCVKCPDFDLCVECFSVGAEVTPHKSNHPYRVMDNLSFPLICPDWNADEEILLLEG

IEMYGLGNWAEVAEHVGTKSKAQCIDHYTTVYMNSPCFPLPDMSHVVGKNRKELLAMAKG

HGEGKKGFPMFGEPMIKEESSFSPSRIKMEDLSKEASTGISPSSLTADSRVDRSVGGKKP

KTSVDEGPSIIELSGYNPKRQEFDPEYDNDAEQSLAEMEFKDSDTEAERELKLRVLHIYL

KRLDERKRRKDFILERKLLYPNPLEKELSPEEKEIYQQYKVFMRFISKEEYEELIKTVIE

EHRVRKRIQDLQEARAAGSHTSADADKYIEQKRKKVAEESAHRAKEGALAGPSGKVLQKT

NRPKGEPDGSPCGGVKGSMELERGGKDLFTSISSDEWDITCFPGSELLSEPDLRLCREIK

LLPSHYLKMLEIISVEIHRSNITQKSDAYRFFKVESRKVDRVYDALVRKGLAQPHP

>XP_026448038.1 transcriptional adapter ADA2-like isoform X2 [Papaver somniferum]

MGRYRGVPNSADEDLSSRSKRKRAGSSGENLDSSSAAPGTSDGKRALYHCNYCNKDISGK

IRIKCVKCPDFDLCIECFSVGAEVTPHKSNHPYRVMDNLSFPLICPDWNADEEILLLEGI

EMYGLGNWAEIAEHVGTKSKALCIDHFTTAYMNSPCFPLPDMSHVVGKNRKELLAMAKGH

NEVKKVTATGFPTFGDFTLKEDSPFSPSRVKIEGQSPSSLTADDSGTGFSSINRASAGAV

KKASSMAQIKETADLVKVEDSHVDRSIGGKKPIPAREEGPSMTELSGYNAKRHEFEPEYD

NDAEQSLAEMEFKGTDTEAECELKLRVLRIYLNRLDERKRRKDFILERNLLNPDPLEKDL

SPEEREIYQRYKVYMRFHSKEEHEELIRTVIEEHGMKKKIQELQEARAAGCRTSAEANRY

HEQKRKREAEESAFKGKESSQLGASGKFLQRVNRPKNEPDGSSPRGGLKGSAGFESGGKG

LSTAPALGFASCLDDWDLTGLPGSDLLSDAEQRLCRESKLLPSHYLKMLEVMSKEVFSGN

ITKTADAYRLFKVDPSKIDRVYDMLVKKGIAQI

>XP_026438398.1 transcriptional adapter ADA2-like isoform X1 [Papaver somniferum]

MGRYRGVPNSADEDLSSRSKRKRAATSGENLDSSSAAPGTSDGKRALYHCNYCNKDISGK

IRIKCVKCPDFDLCIECFSVGAEVTPHKSNHPYRVMDNLSFPLICPDWNADEEILLLEGI

EMYGLGNWAEIAEHVGTKSKALCIDHFTTAYMNSPCFPLPDMSHVVGKNRKELLAMAKGH

NEVKKVAATGFPTFGDFTLKEDSPFSPSRVKIEGQSPSSLTAASDDSGTGFSSINRASAG

AVKKASSMAQIKETADPVKVEDSHVDRSIGGKKPIPAREEGPSMTELSGYNAKRHEFEPE

YDNDAEQSLAEMEFKDTDTEAECELKLRVLRIYLNRLDERKRRKDFILERNLLNADPLEK

DLSPEEREIYQRYKVYMRFHSKEEHEELIRTVIEEHGMKKKIQELQEARAAGCRTSAEAN

RYREQKRKREAEESAFKGKESSQLGASGKFLQRVNRPKSEPDGSSPRGGLKGSAGFESGG

KGLSTTPALGFASCLDDWDLTGLPGSDLLSDAEQRLCRESKLLPSHYLKMLEVMSKEVFS

GNITKTSDAYRLFKVDPSKIDGVYDMLVKKGIAQI

>OVA20155.1 zinc finger protein [Macleaya cordata]

MGRSRGPQASADEDLGQRSKRKRAASSGENSESTAAGQGTNEGKRALYHCNYCNKDISGK

IRIKCVKCPDFDLCVECFSVGAEVQPHKSNHPYRVMDNLSFPLIVPDWNADEEILLLEGI

EMYGLGNWAEVAEHVGTKSKAQCIDHYTSTYMNSPCFPLPDMSHVVGKNRKELLAMAKVQ

GDGKKGFPTLGELTLKEEPTFSPSRVKIEEAAVGRSPSMLTADADAGVGCSSSFAAATGA

VKKASNIARVKDGPDAIKVEDTQLDRSVGVKKPKSSGDEGPSMAELSGYNPKRQEFDPEY

DNDAEQSLADMEFKDTDTEAERELKLRVLHIYGKRLDERKRRKDFILERNLLYPDPLEKD

LSPEEREIYDRYKVFMRFHSKEEHDELIKTVLEEHRLRKRIQELQEARDAGCRTASEADR

YLEQKRRREAEENARRGNPSGHQVGPSGKVLQKGVTRPKGEPDISSPRGGVKGSSSFEAG

GKDQPSTAAGSASSSSLDVWDITGLPGSDLLSESEKCLCGEMKLLPSHYLKMLEVMSIEI

MKGSLTKKSDAHRLFKVDPSKVDRVYDMLVRKGIAPPPGL

>KAF5180867.1 Transcriptional adapter [Thalictrum thalictroides]

MGRSRLPTSVDDDLNQRSKRKRAASSGENLESSNAGQETTEGKRALYHCNYCNKDISGKI

RVKCVKCPDFDLCVECFSVGAEVTPHKSNHPYRVMDNLSFPLICPDWNADEEILLLEGIE

MYGMGNWAEVAEHVGTKGKDQCIEHYTEAYIDSPCFPLPDMSHVMGKNRKELLAMAKGHS

QGRKGFPTLGEVPLKEESAFSPLRIKIEESGVGRSPSSLTSDSGVGSSSTITAATGAIKK

ASDMAHIKDGTDTIKLEESHVDRSIGIKKPKASGDEGPSMTELSGYNPKRHEFDPEYDND

AEQALADMEFKETDTDAEHALKLEMLHIYSRRLDERKRRKDFVLERNLLYPDPLQKDLSP

EEREVYKHYNVFMRFHSKEDHGEFMKTVIEEHRIRRRIRDLQEARDAGCRTSAESNRYIE

QKRKREAEENERKAKESAQIGPGGKLQQKMGRIKGEPDTSFRGSIKGMALDSSVKDTSST

TAGHASANSLDDWDVTGLPGSELLSASEQRLCCEAKMLPSQYLKIAEVISVKIFEGNVTK

KSDAYGFFKVDPSKVDKVYDMLVKKGVAQP

>PIA58225.1 hypothetical protein AQUCO_00500280v1 [Aquilegia coerulea]

MGRSRLPTSVDDDLNQRSKRKRAASSGENLESSNAGQETTEGKRALYHCNYCNKDISGKI

RVKCVKCPDFDLCVECFSVGAEVTPHKSNHPYRVMDNLSFPLICPDWNADEEILLLEGIE

MYGMGNWAEVAEHVGTKGKDQCIEHYTEAYIDSPCFPLPDMSHVIGKNRKELLAMAKGYS

QGRKGFPTLGEVPLKEESAFSPLRIKIEESGVGRSPSSLTSDSGIGSSSTITAASGAVKK

ASDMAHIKDGTDTIKLEEAHVDRSIGIKKPKPSGDEGPSMTELSGYNPKRHEFDPEYDND

AEQALADMEFKDTDTDAEHALKLEMLHIYSRRLDERKRRKDFIIERNLLYPDPLLKDLSP

EEKEIYKHYNVFMRFHSKEEHGDLMKTVIEEHRIRRRIRDLQEARDAGCRTSAESNRYID

LKRKREAEEYERKAKESAQLGPGGKLQQKMSRIKGEPDTSSRGSIKGMCLESSVKDTSST

TAGHAFANSLDDWDVTGLPGSELLSTSEQRLCCEAKMLPSQYLKMAEVISVKIFEGSVTK

KSDAYGFFKVDPSKVDKVYDMLVKKGIAQP

>OVA12002.1 zinc finger protein [Macleaya cordata]

MGRYRGVPSSADEDPSSRSKRKRAASSGENLESAAAVQGTNEGKRALYHCNYCNKDISGK

IRIKCVKCSDFDLCIECFSVGAEVTLHKSNHPYRVMDNLSFPLICPDWNADEEILLLEGI

EMYGLGNWAEIAEHVGTKSKAICIDHFMTAYMNSPCFPLPDMSHVVGKNRKELLAMAKGH

GEVKKGYGLCAQINSWYDSNNACSIYLFSASDVDSGAGNSSTNKASAGAVKKASNMAQIK

DSPDPIKVEDSHMDRSIGEKKPIPSREEGPSMTELSGYNPKRHEFEPEYDNDAEQSLAEM

EFKDTETEAERELKLRVLRIYLKRLDERKRRKDFILERNLLNLNPLEKDLSAEEREIYQR

YKVFMRFHSKEEHEELIKTVIEEHRIRKRIQELQEARAAGCRTSSEADRYLEQKRKEAEE

SAHKGKECLQVGASGKFMQRVNRPKSEPDGSSPRGSLKGSSGFESGGKSSSTTPAAPTST

SSLDDWDLTGLPGTDLLSETEHRLCRESKLLPSQYLKMLEVMSKEIFNGNITKTSDAHRF

FKADTSKVDKVYDMLVKKGIA

>RZC50563.1 hypothetical protein C5167_018981 [Papaver somniferum]

MGRYRGVPNSADEDLSSRSKRKRAGSSGENLDSSSAAPGTSDGKRALYHCNYCNKDISGK

IRIKCVKCPDFDLCIECFSVGAEVTPHKSNHPYRVMDNLSFPLICPDWNADEEILLLEGI

EMYGLGNWAEIAEHVGTKSKALCIDHFTTAYMNSPCFPLPDMSHVVGKNRKELLAMAKGH

NEVKKVTATGFPTFGDFTLKEDSPFSPSRVKVFEIANSVCFFRIEGQSPSSLTAASDDSG

TGFSSINRASAGAVKKASSMAQIKETADLVKVEDSHVDRSIGGKKPIPAREEGPSMTELS

GYNAKRHEFEPEYDNDAEQSLAEMEFKGTDTEAECELKLRVLRIYLNRLDERKRRKDFIL

ERNLLNPDPLEKDLSPEEREIYQRYKVYMRFHSKEEHEELIRTVIEEHGMKKKIQELQEA

RAAGCRTSAEANRYHEQKRKREAEESAFKGKESSQLGASGKFLQRVNRPKNEPDGSSPRG

GLKGSAGFESGGKGLSTAPALGFASCLDDWDLTGLPGSDLLSDAEQRLCRESKLLPSHYL

KMLEVMSKEVFSGNITKTADAYRLFKVDPSKIDRVYDMLVKKGIAQI

>PIA28310.1 hypothetical protein AQUCO_07200158v1 [Aquilegia coerulea]

MERSRCALAATPDRTRKTTVAASSSKQTAVGSASADSKKVEGKKALYHCNYCTKDISGKI

RIKCAICPDFDLCVECFSVGAELTPHKSDHPYQVMDNLSFPLISPDWNADEEILLLEAVE

MYGLGDWEEIAEHVGTKSKAQCNEHYTSFYLNSPHFPLPDMSHVTGKNRKELPSSVEGHT

ENNKELSTLGELRLMEPLYHSGFRTGKPALSPSRFNWFGAGFRNISAAEGTVKKASGMDQ

IKHGRGFPNVKDSLVVPSIGFGGKKPELSSEEKPSLLELSGYNPKRNEFDPEYDTDAEKS

IANLEFKDTDTEAEHELKLQMLHAYNRRLDERKRRKDFVLERNLLYPDPLAKDLSPQEKV

LYDQYNVFMRFHSKEEHEELIKTVIGEHRMRKEFEERKEAQEAGSHASAEADKYFEHKRK

KIAEESSCEMKGSGKVLQRVSPLNGVPDGSALGSVKEPTTVDCRPKDSSSSNDRHAFALS

TDDWGDITGLPGFSLLSEAEKRLCYETKILPSRYLKLQEVISVEFFTGNITKKSDAHRFF

EEDPNKVDRVYDMLVRKGIALP

>PIA58229.1 hypothetical protein AQUCO_00500284v1 [Aquilegia coerulea]

MGRSRGALAATPDRQRTRKTRNSSAAAASSSQQTAADSASSDSKKVEGKKALYHCNYCTR

DISGKIHVKCAICPDFDLCMECFSVGAEVTPHKSDHPYQVTDDLSFPLLSPDWNADEEIL

LLEAIEMYGLGDWDEIAEHVGTKGKAQCIEHYTSFYLNSPNFPLPDMSHVTGKNRKGLQS

SVKGHTENSKELSTLGELRVTEQLHHSGFRTGEPALGLSRFNWFGAGFSNITAAEGTVKM

ASGMDQIKHSRGFPNVKDSVVVPSIGFGGQKPELSSEEGPSLLELSGYNPKRNEFDPEYD

IDAEKLIANLEFKDTDTEAEHELKLRMLHIYNRRLDERKRRKDFVLERNLLYPDPLAKDL

SPQEKELYGHYNVYMRFHSKEEHEELLKTVMREHRIRKRIEELQEAQENGCRTSAEADEY

FEHKRKKIAEECSRETKGRGKVLQRVSLLSGVPDGSPLGGVKEPATVECRPKDSSSSNDR

RALACSTDDWGGITGLPGFCLLSEAEKRLCCETKILPSHYLKLQEVISVEVLRGNITKKS

DAHRFFKEDPNKVDRVYDMLVRKGIALP

>KMZ71610.1 Transcriptional adapter ADA2 [Zostera marina]

MGRSRVAANPTDDDPSQRSKRRRTVSNGENLENVATGTSTTDGKKALYHCNYCNKDITGK

IRIKCTQCPDFDLCIECFSAGVEINPHKNHHPYRVMDNLSFPLITLDWFGDEEILLLEAI

EMYGLANWTEVAEHVGTKIKDQCIEHYTNVYMNSPCFPEPDMSHVLGKNRKELLAMAKGQ

GDGKQGFPVIGDVTPKEDSPFSSSKVKIEDGNRDVLLGRSSPSLNADGESGESKLGVNGC

GSSSLPLGANKKAANVMHIKDGSDRVKHEDFHGNRSIGGKKPKYIVDEGPSLTELSGYNP

KRQEFDPEYDNDAEQLLADMEFKETDSEIERNVKLRVLQIYASRLDERKRRKTFILERNL

LFPNSFEKDLSPEEKAIYDRFKVFMRFHTLEEHELLMRSLIGEYRLRKRIQELQEAQVAG

CCTRTEADAYLARQAAKKLNNNNQVGSTKVFQRGNLANKGEYDGSSPGSVGENQKSKGVG

GVESNSRDSSSCITSCLEDWDIVGLPGVSLLSEAEKQFCCANRLLPCHFLKIQQVLMKEI

ITGSVAKKADAHRLFKVDSIKVDKIYDMVSKKLDLNEEQSV

>XP_020570651.1 transcriptional adapter ADA2-like isoform X1 [Phalaenopsis equestris]

MGRSRGVSNSADDDANQRSKRRRVAPAGENLENASTGQGGADGKKALYHCNYCNKDISGK

IRIKCSKCPDFDLCVECFSVGAEVTPHRSNHPYRVMDNLSFPLICQDWNADEEILLLEGI

EMYGLGNWTEVAEHVGTKNKEQCINHYTALYMNSPSYPLPDMSRVNGKNRKELLAMAKAQ

GEGKKGISVLGDATPKEESPFSPSRFKFEELSGEVLAGRSPSGLTAGASKKVSNMGQLKD

VSDGTKIESLTPVEDHVDRSVGVKKPKYSGDEVPSLTELSGFNSKRQEFDPEYDNDAEQS

LAEMEFKETDTDTDRELKLRVLHIYLSRLEERKRRKDFILERNLLYPHPLEKELSNEDRE

LYHRYKVFMRFLSQEDHDALVSNVIEERRIRRKIQELQECRVAGCRTLAEAKMYTDQKRR

KEAEANAQKAKEPTALINNNNNKLMQKINKPISRDKIDEGSPLSMVDNHKIKVAGLDSSS

KDSPLTSGQPILRSFDDWDVTGLPGADLLSETEQIFCCQNRLFPSHYLKMQEVLVQEILK

GAVAKAADAHQLFKFDPDKIDKIYDLVAKKLGHTEDLFNA

>XP_020696921.1 transcriptional adapter ADA2 [Dendrobium catenatum]

MGRSRGVSNSADDDVNQRSKRRRVAPAGENLENASTGQGGTEGKKALYHCNYCNKDISGK

IRIKCSKCPDFDLCVECFSVGAEVTPHRSNHPYRVMDNLSFPLICQDWNADEEILLLEGI

EMYGLGNWAEVAEHVGTKNKEQCINHYTALYMNSPSYPLPDMSRVNGKNRKELLAMAKAQ

GEGKKGISVLGDVTPKEESPFSPSRIKVEDLSEVHAGRSPSGLTVGASKKVSNVGQLKEV

SDVTKVESLITAEDHVDRSVGVKKPKYSGDEAPSLTELSGFNPRRQEFDLEYDNDAEQSL

ADMEFKETDTETDRELKLRVLHIYLSRLEERRRRKDFILERNLLYPHPLEKELANEDREV

YHRYKVFMRFLSQEEHDALVSSVIEERRIRRRIQELQECRAAGCRTLAEAKMYAEQKRRK

ELEANVQKTKETSALINNNKVTQKINRPISRDKIDSDGSPLSMVDNHKIKGLTLDSSGKD

SPVTSGQANLRSFDDWDIIGLPGADLLTESEQLLCCQNRLFPSHYLKMQEVLVQEILKGA

VVKAVDAHQLFKIDPNKIDKIYDLVTKKLGQSEDLFNA

>PKA58661.1 Transcriptional adapter ADA2 [Apostasia shenzhenica]

MGRSRAVSNSADDDANQRSKRRRVASTGENLENTTTSQSGTEGKKALYHCNYCNKDISGK

IRIKCTKCPDFDLCVECFSVGAEVTPHKSNHPYRVMDNLSFPLICQDWNADEEILLLEGI

EMYGLGNWAEVAEHVGTKNKEQCINHYMALYMNSPSYPLPDMSRVNGKNRKELLAMAKAQ

GISNLGNVTPKEDSPFSPSRVKIEDLSAEALGGRSPSGTFKKVSNLGQLKGGSDETKREN

LSLPEDVNGDRTIGIKKPKYSKDEVPSMTELSGYNPKRREFDPEYDNDAEKLLAEMEFKD

TDTETDRELKLRNSCLWRDFADRLDERKRRKDFILERNLLYPHPMEKDLSDEDREVYQRF

KVFMRFLPQEEFETLVKSVLEERKMRRRIQELQECRAAGCRTLADAKMYIEQKRKELEAN

AKKTNETSPVVNNGKVLQKANRTLSREKIETDGTVESLKVRVGASIDSAGKDLSLATGQA

SLKLFEEWDIRGLPGTEFLSESEYQLCCDNRLLPNHFLKMQEALVQQILKGAVTKKDDAH

HLFKVDPNKIDKVYEIVRKKLGQNEDSANV

>XP_020271187.1 transcriptional adapter ADA2 [Asparagus officinalis]

MGRSRGVSNSTDEDGSQRSKRRRVASSGENLETSNTGPGNSEGKKALYHCNYCNKDISGK

IRIKCTKCPDFDLCVECFSVGAEVTPHKSNHPYRVMDNLSFPLIXXXXYCFLSLHGIEMY

GLGNWAEIAEHVGTKSKLQCIDHYTTAYMNSPCYPLPDMSRVNGKNRKELLAMAKVQGEG

KKGISMLGDLTPKEESPFSPSRVKIEELSEAPTGRPPSGLTSGATKKASNNGQFKVVPDG

TKVEGLTSSEDLYVDRSVGVKKPKYSGDEGPSMTELSGYNAKRQEFDPEYDNDAEKSLAE

MEFKDTDSEAERELKLRVLHIYLSRLDERKRRKDFILERNLLYPNPLEKELKNEDREVYQ

HFKVFMRFLPQEEYEALLNTVIEERKIRRRIHELQVISAWGMXXXXXXXXXTLAEVKMYT

EQKRRKELETSEHKSKESDQIVSSVKAVQKSNRPISREKCEPDGSPRNTVDNHKIRGGNG

LDPGGKDSPVLQVSANAFDEWNITGLPGTELLSETEQQLCCQSRLLPSFYLKMQEVLVQE

IFKGSVMRKADAYPLFKVDPSKVDKVYDIVTKKLGQHEEAPNV

>XP_008780988.1 transcriptional adapter ADA2-like isoform X1 [Phoenix dactylifera]

MGRSRGVPNTGDDEANQRSKRRRVASSGETLENMATGPGASEGKKALYHCNYCNKDISGK

IRIKCTKCPDFDLCVECFSVGAEVTPHKSNHPYRVMDNLSFPLICPDWNADEEILLLEGI

EMYGLGNWAEVAEHVGTKSKAQCIDHYTTAYMNSPCYPLPDMSRVNGKNRKELLAMAKMQ

GEGKKGVSVLGDVTPKEESPFSPSRVKIEDVSGEAAAGRSPSLASGASKKASSTGQLKDG

SDGTKLEDPHVDRSVGVKKPKYTGDEGPSMTELSGFNPKRQEFDPEYDNDAEQALAEMEF

KETDTETDRELKLRVLRIYLSRLDERKRRKDFILERNLLYPNPLEKELSSEDREIYNRYK

VFMRFLSPEDHEALVKSVIEERKIRRRIQELQECRAAGCRSLAEAKAYIEQKRKRELEAS

AQKAKESKEGNQVVASSKVMQKAARPMNREKGESDGSPRNTIDNHKIKGGAGLDSGSKDS

PSTTFDDWDITGLPGAELLSEHERQLCCQNRLLPSHYLKMQQKLVEEIFKGTVVKKSDAH

GLFKVDPVKVDTVYDIVIKKLGQHDESAAV

>XP_010907578.1 transcriptional adapter ADA2 [Elaeis guineensis]

MGRSRGVPNTGDDEANQRSKRRRVASSGETLENMAAGPGASEGKKALYHCNYCNKDISGK

IRIKCTKCPDFDLCVECFSVGAEVTPHKSNHPYRVMDNLSFPLICPEWNADEEILLLEGI

EMYGLGNWAEVAEHVGTKSKVQCIDHYTTAYMNSPCYPLPDMSRVNGKNRKELLAMAKMQ

GEGKKGVSVLGDVTPKEESPFSPSRVKIEDVSGEVAAGRSPSLASGASKKASNMGQLKDG

SDGTKLEDPHVDRSVGVKKPKYTGDEGPSMTVLSGFNPKRQEFDPEYDNDAEQALAEMEF

KETDTETDRELKLRVLHIYLSRLDERKRRKEFILERNLLYPNPLEKELSSEDREIYNRYK

VFMRFLSPEDHEALVKSVIEERKIRRRIQELQECRAAGCRSLAEAKAYIEQKRKRELEAS

AQKSKESKEGNQVVASSKVMQKTARPMNREKGESDGSPRNTIDNHKIKGGAGLDSGSKDS

LFTSDDWDITGLPGSELLSEHEQQFCCRNRLLPSHYLKMQEKLVEEIFRGTVVKKSDAHG

LFKVDPVKVDRVYDIVMKKLGQHDESAPV

>XP_010905667.2 transcriptional adapter ADA2 isoform X1 [Elaeis guineensis]

MGRSRGETNSTGKDSRQRSKKRRAVPVKEDAETLDAGTKERKYHCNYCNKDISGMIRIKC

AICADFDLCVECFSVGAEVSPHKSNHPYRVMDNKSFPLLCSDWNADEEILLLEGIDMYGL

GGWAEIAEHVGTKNKAQCIDHYTTAYMNSPSFPLPGMSHVHGAAKTDLLAMDNVQGISVP

QNASPKVESLFSSSGLKIGIPSSEPPAERYSSSTSVWTGEDCTNMSSTAANMKAPNMLKF

TNGSGGTKLESSTSAEGTHGDTSSGIKKTKDPGDNGLSLSELNGYNPKTQEFDPEYDDDA

EKALADMKFMPNDTEIHHELKLRMLHIYCSRLDERKRRKDFILERNLLHTIPLEKELSVE

DREVYERYKVFMRYQSPEEHDALVQSVIEEHKLRRRIQELQECRAAGCRTMAEAKAYIEQ

KKLRELEASMNKTKLDSQVSSSSKILRANELSNKEDGLSDGSPQNINEGNGGSGLELDGK

DPSAVATMQVTSKSFDVWDVTGLPGADLLSETERRLCCECKLLPCHYLKIQEVLMTEIYK

GTIVKKSDAYSFFKVDPSKINKIYDVLSKWSKRFLYYLSYLMEPPMGNLLPWNLRKAHSS

EVSGGIQSISLRHANFSAFLIRFLSILHFVKATTLVKFYFCNIMATLLLLLI

>XP_008800601.1 transcriptional adapter ADA2-like isoform X1 [Phoenix dactylifera]

MGRSRGETNSAGKDSRQRSKKRRAVPVKEDAKTLDAGTKERKYHCNYCNKDISGMIRIKC

AICADFDLCVECFSVGAEVSPHKSDHPYRVMDSKSFPLLCSDWNADEEILLLEGIDMYGL

GSWEEIAEHVATKNKTQCIDHYTTAYMNSPCFPLPDMSHVNGTAKTDLLAMDNVQGILVP

QSASPKEESLFSSSGIKIEIPSREAPAEQYSSSTAGGDCTNISSTAANTRASNMLKFKND

SGGTRVESSTSAEGTHGDTSFGVEKPKYPGDDGLSLSELSGYNPKRQEFDPEYDDDAEKA

LADMKFMPNDTEIHHELKLRMLHIYCSRLDERKRRKDFILERNLLHTIPLEKELPVEDRE

VYERYKVFMHYQSPEEHDALVQSVIEERKLRRRIQELQECRAAGCRTMAEAKAYIEQKKL

RELEASINKTKSDSQVSSSGKTLRANELSNKEDGLSDGSPQNINEVNGGSGLELDRKDPS

SVATMQVPSKSFDAWDVTGLPGADLLSETEQRLCCQCGLLPCHYLKIQEVLMSEIYKGTI

VKKSDAYSFFKVDPSKINKIYDMLSKWSKRLLYYLSCLTEPPTGNLLPMESLERTLK

>XP_009402837.1 PREDICTED: transcriptional adapter ADA2 isoform X2 [Musa acuminata subsp. malaccensis]

MGRSRAVPNSGDDDTSHRSKRRRVAASGEALGNIEAGSGTSEGKKALYHCNYCNKDISGK

IRIKCTKCADFDLCVECFSVGAEVTPHKSNHPYRVMDNLSFPLICPDWNADEEILLLEGI

EMYGLGNWTEVAEHVGTKSKAQCIDHYTTSYLNSPCYPLPDMSRVNGKNRKELLAMAKVQ

VEGKKGSSLLGDVTPKEESPFSPARVKVEDIGEGAANQSPSNLAAGASKVTSNTGKFKDN

PDGPKVEDSYLDRTIGVKKPKCSGEEGPSITESGYNPKRQEFDPEYDNDAEQALADMEFK

ENDTETERELKLRVLRIYLSRLDERKRRKDFILERNLLYPNPLEKELSSEDRELYNRFKV

FMRFLSQEEHENLVKSVIEERKIRRRIQELQECRAAGCRTLAEAKAYTEQKRKRELEVGA

QNSKENTQILSGGKVAQKANRPLNREKGDNDGSPRNTTDNHKIKGSTGFESSGKDSPSTT

TGQVSVRSFDEWDITGLPGTEFLSETEQDFCCQNRLLPSHYLKMQETLVQEIYKGNIINK

SDAHGLFKVDPVKVDKVYDIVKRKLGQQEESTIV

>THU74195.1 hypothetical protein C4D60_Mb04t30810 [Musa balbisiana]

MGRSRALPNSGDDETNQRSKRRRVASSGEALETITAGPGANEGKKALYHCNYCNKDISGK

IRIKCTKCPDFDLCVECFSVGAEVTPHKSNHPYRVMDNLSFPLICPDWNADEEILLLEGI

EMYSLGNWVEVAEHVGTKSKALCIEHYTSSYLNSPCYPLPDMSRVNGKNRKELLAMAKVQ

VEGKKGASKMASNVGQLKENSDGPKAEDSYADRTIGVKKPKCSGDEGPSITESGYNPKRQ

EFDPEYDNDAEQSLADMEFKENDSETEHELKLRVLRIYLSRLDERKRRKDFILERNLLYP

NPLEKELSNEDREIYNHFKVFMRFLSQEEHEDLVKSVIEERKIRRRIQELQECRAAGCRT

LAEAKEYTEQKRKRELQASTQNSKENTQVLSGSKLVQKANRPLNKEKGENDGSPKNTIDN

HKIKGGTGLDSGGKDSLSTATGQVSVRSFDEWDITGLPGTELLSETEQEFCCQNTLLPSH

YLKMQETLVQEIYKGNIANKSDAHGLFKVDPVKVDGVYDMVKKKLGQQEEPTVV

>XP_020092928.1 transcriptional adapter ADA2-like [Ananas comosus]

MGRSRAVPNSGDDEANQRSKRRRVASSGETLETITSGSGTNDGKKALYHCNYCNKDISGK

IRIKCSKCPDFDLCVECFSVGAEVTPHKSNHPYRVMDNLSFPLICPDWNADEEILLLEGI

EMYGLGNWAEVAEHVGTKSKAQCIDHYTTAYMNSPCYPLPDMSRVNGKNRKELLAMAKVQ

GEGKKGNSMLGDVTVKEESPFSPSRVKVEDSGGEAPAGRSPSNLSADKHMDRSVGVKKPK

YSGEEGPSVAELSGYNPKRQEFDPEYDNDAEKSLAEMEFKENDSETDRELKLRVLRIYLS

RLDERKRRKEFILERKLLFPNPLEKELSNEDKEIYHRFKVFMRFLSQEEHETLVRSVIEE

KKIRRRIQELQECRAAGCRTLAEAKTYIEQKRKKEIEANAQKTKESGQALLSTKGVQKTG

RPAKVESDGSPRNSVDPKIKGSVGLDSGGKDSPTAMGLINTKSWDDWDITGLPGAELLSE

TEQRLCCQNRLLPSHYLKMQEVLVQEILKGTVMKKSDAHGLFKVDPIKLDHIYEMVTKKL

GHHEESPIA

>KAF3334559.1 transcriptional adapter ADA2-like isoform X1 [Carex littledalei]

MGRSRGVANAGDDDTSQRSKRRRVPSGGDALENITSQGGTDGKKALYHCNYCNKDISGKI

RIKCSKCPDFDLCVECFSVGAEVTPHKSNHPYRVMDNLSFPLICADWNADEEILLLEGIE

MYGLGNWAEVAEHVGTKSKAQCIDHYMTAYLSSPCFPLPDMSRVNGKNRKELLAMAKVQG

EAKRGSSLLGETTLTDSPFSPSRVKMEDAIGDALNGRSPSNLTSGANKKASNVGHLKDSS

DGPRPEDTHMDRSVGGKKPKYSGEEGPSMAELSGYNAKRQEFDPEYDNNAEQALAEMEFK

DTDTDVERELKIRVLNIYLKRLDERKRRKDFIIERNLLYPNPLEMEFSSEDKEVYERYKV

FMRFLSPEDHEKLLRNLVEERKLRNRIQDLQICRAAGCRTISEANAYLEQKRKKELEESM

QRSKEAGASLKPQIQHKAINRPNMMKVGGAESEVGSPRSTVLDAKKGGSTESPVGSTGAA

RSGWDDWDLTGLPGTDLLSATEKRLCCDTHLLPVHYLKMQEVIVKEIFKGTVHKKSDAYG

FFKVEPAKVDLVYEMVVKKLGQIDGNPLA

>KAF0915178.1 hypothetical protein E2562_034090 [Oryza meyeriana var. granulata]

MGRSRGVPNSGDDDTNHRSKRRRVASSGDASDSLSAACGGAGEGSGKKALYHCNYCNKDI

SGKIRIKCSKCPDFDLCVECFSVGAEVTPHRSNHPYRVMDNLSFPLICPDWNADEEILLL

EGIEMYGLGNWAEVAEHVGTKSKAQCIDHYTTAYMNSPCYPLPDMSHVNGKNRKELLAMA

KVQGESKKGTSVLPGDLTPKDESPFSPPRVKVEDALGEGLAGRSPSHIAGGANKKASNVG

QFKDGSNVAKVEDGHVDRSIGVKKPRYSADEGPSLTELSGYNSKRNEFDPEYDNDAEQAL

AEMEFKETDSETDRELKLRVLRIYLSRLDERKRRKEFILERNLLFPNPLEKDLTNEDKEV

YHRYKVFMRFLSKEEHEALVRSVLEERKIRRRIQELQECRSAGCRTLAEAKIHIEQKRKK

ELEVNAQKAKESGQLLSNTKVVHKTNRPMKIESDGNLDQKKGGASLDSGGRDSPKITGHA

GTKHWDDWDIVGLPGAELLSASEKNLCCQNRLLPSHYLKMQEVLMQEIFKGSVVKKEDAH

VLFKVDPAKVDTVYDMVTKKLGTNEEVPTV

>Q75LL6.2 RecName: Full=Transcriptional adapter ADA2 [Oryza sativa Japonica Group]

MGRSRGVPNSGDDETNHRSKRRRVASSGDAPDSLSAACGGAGEGGGKKALYHCNYCNKDI

SGKIRIKCSKCPDFDLCVECFSVGAEVTPHRSNHPYRVMDNLSFPLICPDWNADEEILLL

EGIEMYGLGNWAEVAEHVGTKTKAQCIDHYTTAYMNSPCYPLPDMSHVNGKNRKELLAMA

KVQGESKKVLPGDLTPKDESPFSPPRVKVEDALGEGLAGRSPSHIAGGANKKASNVGQFK

DGANVAKVEDGHVDRSIGVKKPRYSADEGPSLTELSGYNSKRHEFDPEYDNDAEQALAEM

EFKETDSETDRELKLRVLRIYLSRLDERKRRKEFILERNLLFPNPLEKDLTNEDKEVYHR

YKVFMRFLSKEEHEALVRSVLEERKIRRRIQELQECRSAGCRTLAEAKIHIEQKRKKEHE

VNAQKAKESGQLLSNTKVVHKTNRPMKIESDGNLDQKKGGASLDSTGRDSPKTTGHAGTK

HWDDWDIVGFPGAELLSTSEKNLCCQNRLLPNHYLKMQEVLMQEIFKGSVAKKEDAHVLF

KVDPAKVDNVYDMVTKKLGTNEEAPTV

>XP_006651826.1 PREDICTED: transcriptional adapter ADA2 [Oryza brachyantha]

MGRSRGVPNSGDDDTNHRSKRRRVASSGDASDSLSAAGGGGGGGGGGEGGGKKALYHCNY

CNKDISGKIRIKCSKCPDFDLCVECFSVGAEVTPHRSNHPYRVMDNLSFPLICPDWNADE

EILLLEGIEMYGLGNWAEVAEHVGTKGKAQCIDHYTTAYMNSPCYPLPDMSHVNGKNRKE

LLAMAKVQGESKKGTSVLPGDLTPKDESPFSPPRVKVEDALGEGLAGRSPSHIASGANKK

ASNVGQFKDGANVAKVEDGHVDRSIGVKKPRYSADEGPSLTELSGYNSKRNEFDPEYDND

AEQALAEMEFKETDSETDRELKLRVLRIYLSRLDERKRRKEFILERNLLFPNPLEKDLTN

EDKEVYHRYKVFMRFLSKEEHEALVRSVLEERKIRRRIQELQECRSAGCRTLAEAKIHIE

QKRKKEHEVNAQKAKESGQLLSNTKVMHKTNRPMKIESDGNLDQKKGGASLESGGRDSPK

TTGHAGTKHWDDWDIVGLPGAELLSANEKVLCCQNKLLPCHYLKMQEVLMQEIFKGSVAK

KEDAHVLFKVDPAKVDTVYDMVTKKLGTNEEAPTV

>VAI22003.1 unnamed protein product [Triticum turgidum subsp. durum]

MGRSRGVPNSGDDDTNHRSKRRRVASTGDASDSLSAACGGAGDGKKALYHCNYCNKDLSG

KIRFKCSKCPDFDLCVECFSVGAEVTPHRSNHPYRVMDNLSFPLICPDWNADEEILLLEG

IEMYGLGNWAEVAEHVGTKSKAQCIEHYTTAYMNSPCYPLPDMSHVNGKNRKELLAMAKV

QGESKKGIPLLSGDLTPKAESPFSPSRIKKKASTVGHFKDSANLSKVEDGHMDRSIGVKK

PRYSADEGPSLTELSGYNAKRHEFDPEYDNDAEQALAEMEFKETDSETDRELKLRVLRIY

LSRLDERKRRKEFILERNLLYPNPLEKDLTNEDKEVYHRYKVFMRFLSKEEHEALVRSVI

EERKIRRRIQELQECRSAGCRTLAEAKIHIEQKRRKEYEANALKAKESGQLISNSKSGHK

TNRPMKLETDGSLDLKKGSGILDAGGRDSPKTTGPTSAKQWDDWDIVGLPGAELLSASEK

LLCCQNRLLPSHYLRMQEVLMQEMFKGNVVKKEDAHVLFKVDPAKVDTVYDMVTKKLGNN

EEAPMV

>QKE45383.1 alteration/deficiency in activation 2-B [Triticum aestivum]

MGRSRGVPNSGDDDTNHRSKRRRVASTGDASDSLSAACGGAGDGKKALYHCNYCNKDLSG

KIRFKCSKCPDFDLCVECFSVGAEVTPHRSNHPYRVMDNLSFPLICPDWNADEEILLLEG

IEMYGLGNWAEVAEHVGTKSKAQCIEHYTTAYMNSPCYPLPDMSHVNGKNRKELLAMAKV

QGESKKGIPLLSGDLTPKAESPFSPSRIKMEDALGEGPASRSPSHIPGGANKKASTVGHF

KDSANLSKVEDGHMDRSIGVKKPRYSADEGPSLTELSGYNAKRHEFDPEYDNDAEQALAE

MEFKETDSETDRELKLRVLRIYLSRLDERKRRKEFILERNLLYPNPLEKDLTNEDKEVYH

RYKVFMRFLSKEEHEALVRSVIEERKIRRRIQELQECRSAGCRTLAEAKIHIEQKRRKEY

EANALKAKESGQLISNSKSGHKTNRPMKVETDGSLDLKKGSAILDAGGRDSPKSTGPTSA

KQWDDWDIVGLPGAELLSSSEKLLCCQNRLLPSHYLRMQEVLMQEMFKGNVVKKEDAHVL

FKVDPAKVDTVYDMVMKKLGNNEEAPMV

>XP_020149065.1 transcriptional adapter ADA2 [Aegilops tauschii subsp. tauschii]

MGRSRGVPNSGDDDTNHRSKRRRVASTGDASDSLSAACGGAGDGKKALYHCNYCNKDLSG

KIRFKCSKCPDFDLCVECFSVGAEVTPHRSNHPYRVMDNLSFPLICPDWNADEEILLLEG

IEMYGLGNWAEVAEHVGTKSKAQCIEHYTTAYMNSPCYPLPDMSHVNGKNRKELLAMAKV

QGESKKGIPLLSGDLTPKAESPFSPSRIKMEDALGEGPASRSPSHIPGGANKKASTVGHF

KDSANLSKVEDGHMDRSIGVKKPRYSADEGPSLTELSGYNAKRHEFDPEYDNDAEQALAE

MEFKETDSETDRELKLRVLRIYLSRLDERKRRKEFILERNLLYPNPLEKDLTNEDKEVYH

RYKVFMRFLSKEEHEALVRSVIEERKIRRRIQELQECRSAGCRTLAEAKIHIEQKRRKEY

EANALKAKESGQLISNSKSGHKTNRPMKLETDGSLDLKKGSAILDAGGRDSPKTTGPTSA

KQWDDWDIVGLPGAELLSSSEKLLCCQNRLLPSHYLRMQEVLMQEMFKGNVVKKEDAHVL

FKVDPAKVDTVYDMVMKKLGNNEEAPMV

>EMS62919.1 Transcriptional adapter ADA2 [Triticum urartu]

MGRSRGVPNSGDDDTNHRSKRRRVASTGDASDSLSAACGGAGDGKKALYHCNYCNKDLSG

KIRFKCSKCPDFDLCVECFSVGAEVTPHRSNHPYRVMDNLSFPLICPDWNADEEILLLEG

IEMYGLGNWAEVAEHVGTKSKAQCIEHYTTAYMNSPCYPLPDMSHVNGKNRKELLAMAKV

QGESKKGIPLLSGDLTPKAESPFSPSRIKMEDALGEGPASRSPSHIPGGANKKASTVGHF

KDSANLSKVEDGHMDRSIGVKKPRYSADEGPSLTELSGYNAKRHEFDPEYDNDAEQALAE

MEFKETDSETDRELKLRVLRIYLSRLDERKRRKEFILERNLLYPNPLEKDLTNEDKEVYH

RYKVFMRFLSKEEHEALVRSVIEERKIRRRIQELQECRSAGCRTLAEAKIHIEQKRRKEY

EANALKAKESGQLISNSKSGHKTNRPMKLETDGSLDLKKGSGILDAGGRDSPKTTGPTSA

KQWDDWDIVGLPGAELLSASEKLLCCQNRLLPSHYLRMQEVLMQEMFKGNVVKKEDAHVL

FKVDPAKVDTVYDMVTKKLGNNEEAPMV

>BAJ97449.1 predicted protein [Hordeum vulgare subsp. vulgare]

MGRSRGVPNSGDDDTNHRSKRRRVASTGDASDSLSAACGGAGDGKKALYHCNYCNKDLSG

KIRFKCSKCPDFDLCVECFSVGAEVQPHRSNHPYRVMDNLSFPLICPDWNADEEILLLEG

IEMYGLGNWAEVAEHVGTKSKAQCIEHYTTAYMNSPCYPLPDMSHVNGKNRKELLAMAKV

QGESKKGIPLLSGDLTPKAESPFSPSRVKMEDALGEGPASRSPSHIPGGANKKASTAGHF

KDNSNLSKVEDGHMDRSIGVKKPRYSADEGPSLTELSGYNAKRHEFDPEYDNDAEQALAE

MEFKETDTETDRELKLRVLRIYLSRLDERKRRKEFILERNLLYPNPLEKDLTNEDKEVYH

RYKVFMRFLSKEEHEALVRSVIEERKIRRRIQELQECRSAGCRTLAEAKIHIEQKRRKEY

EANALKAKESGQLISNSKSGHKTNRPMKVGTDGSLDLKKGSAILDAGGRDSPKSTGPTSA

KQWDDWDIVGLPGAELLSVSEKLLCCQNRLLPSHYLRMQEVLMQEMFKGNIVKKEDAHVL

FKVDPAKVDTVYDMVTKKLGNNEEAPMV

>XP_003559501.1 transcriptional adapter ADA2 [Brachypodium distachyon]

MGRSRGVPNPGDDDTNHRSKRRRVASSGDATDSLSAACGGAGDGKKALYHCNYCNKDLSG

KIRFKCSKCPDFDLCVECFSVGAEVQPHRSNHPYRVMDNLSFPLICPDWNADEEILLLEG

IEMYGLGNWAEVAEHVGTKGKAQCIEHYTTAYMNSPCYPLPDMSHVNGKNRKELLAMAKV

QGESKKGISVLPGDSTPKDESPFSPSRVKVEDAPGEGPAGRSPSHIAGGANKKASTAGHF

KDSANLAKMEDGHMDRSIGVKKPRYSADEGPSLTELSGYNAKRHEFDPEYDNDAEQALAE

MEFKETDSETDRELKLRVLRIYLSRLDERKRRKEFILERNLLYPNPLEKDLTNEDKEVYH

RYKVFMRFLSKEEHEALVRSVIEERKIRRRIQELQECRSAGCRTLAEAKIHIEQKRRKEY

EANAHKAKESGQLIPTTKVVHKTNRPMKLESDGNLDPKKSNATVDSGGRDSPKATGHTVA

KQWDDWDIVGLPGAELLSASEKLLCCQNRLLPSHYLRMQEVLMQEMFKGSVVKREDAHVL

FKVDPAKVDTVYDMVMKKLGSNEEAPTV

>KAE8817073.1 Transcriptional adapter ADA2 [Hordeum vulgare]

MGRSRGVPNSGDDDTNHRSKRRRVASTGDASDSLSAACGGAGDGKKALYHCNYCNKDLSG

KIRFKCSKCPDFDLCVECFSVGAEVQPHRSNHPYRVMDNLSFPLICPDWNADEEILLLEG

IEMYGLGNWAEVAEHVGTKSKAQCIEHYTTAYMNSPCYPLPDMSHVNGKNRKELLAMAKV

QGESKKGIPLLSGDLTPKAESPFSPSRVKMEDALGEGPASRSPSHIPGGANKKASTAGHF

KDNSNLSKVEDGHMDRSIGVKKPRYSADEGPSLTELSGYNAKRHEFDPEYDNDAEQALAE

MEFKETDTETDRELKLRVLRIYLSRLDERKRRKEFILERNLLYPNPLEKDLTNEDKEVYH

RYKVFMRFLSKEEHEALVRSVIEERKIRRRIQELQECRSAGCRTLAEAKIHIEQKRRKEY

EANALKAKESGQLISNSKSGHKTNRPMKVETDGSLDLKKGSAILDAGGRDSPKSTGPTSA

KQWDDWDIVGLPGAELLSVSEKLLCCQNRLLPSHYLRMQEVLMQEMFKGNIVKKEDAHVL

FKVDPAKVDTVYDMVTKKLGNNEEAPMV

>NP_001105146.2 histone acetyltransferase complex component102 [Zea mays]

MGRSRGVQNSGDDDTVHRSKRRRVASGGDATDSVSAGIGGAGEGGGKKALYHCNYCNKDI

SGKIRIKCSKCPDFDLCVECFSVGAEVTPHRSNHPYKVMDNLSFPLICPDWNADEEILLL

EGIEMYGLGNWLEVAEHVGTKSKLQCIDHYTTAYMNSPCYPLPDMSHVNGKNRKELLAMA

KVQGESKKGTSLLPGELTPKAESPFSPSRVKVEDALGEGLAGRSPSHIAVGANKKASNVG

HIKDGSNVSKVEDGHVDRSVGVKKPRYSADEGPSLTELSGYNAKRHEFDPEYDNDAEQAL

AEMEFKETDSETDRELKLRVLRIYLSRLDERKRRKEFILERNLLFPNPLEKDLTNEDREV

YHRYKVFMRFLSKEEHEALVRSVIEERKIRRRIQELQECRSAGCRTLAEAKIHIEQKRKK

EYELNAQKAKESNHLIANTKLVQKMNRPMKIESDGNLDPKKGGVGLDSPKTTGLTSVKQW

DDWDIVGLPGAELLSASEKLLCCQNRLLPSHYLRMQEVLMQEIFKGSVLKKEDAHVLFKV

DPTKVDSVYDMVTKKLGNHVELPTV

>RLN42363.1 transcriptional adapter ADA2 [Panicum miliaceum]

MGRSRGVPNSGDDDTGHRSKRRRVSSGGDATDTISAAMGGAGEGGGKKALYHCNYCNKDI

SGKIRIKCSKCPDFDLCVECFSVGAEVTPHRSNHPYRVMDNLSFPLICPDWNADEEILLL

EGIEMYGLGNWLEVAEHVGTKSKLQCIDHYTTAYMNSPCYPLPDMSHVNGKNRKELLAMA

KVQGESKKGTSLLPGELTPKAESPFSPSRIKLEEALGDGPAGRSPSHMAGGANKKASNAG

QIKDGANVLKVEDGHVDRSVGVKKPRYSADEGPSLTELSGYNSKRHEFDPEYDNDAEQAL

AEMEFKETDSETDRELKLRVLRIYLSRLDERKRRKEFILERNLLFPNPLEKDLTNEDKEV

YHRYKVFMRFLSKEEHEALVRSVIEERKIRRRIQELQECRSAGCRTLAEAKIHIEQKRKR

EYELNAQKSKESGQLIPNNKSVQKMNRPMKIESDGNLDPKKGGTGLDSPKTTGVTSVKQW

DDWDIVGLPGAELLSANEKLLCCQNRLLPSHYLRMQEVLMQEIFKGNVLKKEDAHVLFKV

DPTKVDTVYDMVTKKLGNHEEAPTV

>PUZ37009.1 hypothetical protein GQ55_9G083900 [Panicum hallii var. hallii]

MGRSRGVPNSGDDDTGHRSKRRRVSSSGDATDTISAAMGGAGEGGGKKALYHCNYCNKDI

SGKIRIKCSKCPDFDLCVECFSVGAEVTPHRSNHPYRVMDNLSFPLICPDWNADEEILLL

EGIEMYGLGNWLEVAEHVGTKSKLQCIDHYTTAYMNSPCYPLPDMSHVNGKNRKELLAMA

KVQGESKKGTSLLPGELTPKAESPFSPSRVKLEEALGDGPAGRSPSHLAGGANKKASNAG

QIKDGANVSKVEDGHLDRSVGVKKPRYSADEGPSLTELSGYNSKRHEFDPEYDNDAEQAL

AEMEFKETDSETDRELKLRVLRIYLSRLDERKRRKEFILERNLLFPNPLEKDLTNEDKEV

YHRYKVFMRFLSKEEHEALVRSVIEERKIRRRIQELQECRSAGCRTLAEAKIHIEQKRKK

EYELNAQKSKESGQLIPNNKSVQKMNRPMKIESDGNLDPKKGGTGLDSPKTAGLTSVKQW

DDWDIVGLPGAELLSASEKLLCCQNRLLPSHYLRMQEVLMQEIFKGNVLKKEDAHVLFKV

DPTKVDTVYDMVTKKLGNHEEAPTV

>XP_002463870.1 transcriptional adapter ADA2 [Sorghum bicolor]

MGRSRGVPNSGDDDTGHRSKRRRVSGSGGDATDSISAAIGGAGEGGGKKALYHCNYCNKD

ISGKIRIKCSKCPDFDLCVECFSVGAEVTPHRSNHPYRVMDNLSFPLICPDWNADEEILL

LEGIEMYGLGNWLEVAEHVGTKSKLQCIDHYTTAYMNSPCYPLPDMSHVNGKNRKELLAM

AKVQGESKKGTSLLSGELTPKAESPFSPSRVKVEDALGEGPAGRSPSHIAVGANKKASNV

GQIKDGANVSKIEDGHVDRSVGVKKPRYSADEGPSLTELSGYNAKRHEFDPEYDNDAEQA

LAEMEFKETDSETDRELKLRVLRIYLSRLEERKRRKEFILERNLLFPNPLEKDLTNEDRE

VYHRYKVFMRFLSKEEHEALIRSVIEERKIRRRIQELQECRSAGCRTLAEAKIHIEQKRK

KEYELNAQKAKESSLIANNKSVQKMNRSMKIESDGNLDPKKGGAGLDSPKTTGLTSVKQW

DDWDIVGLPGAELLSASEKLLCCQNRLLPSHYLRMQEVLMQEIFKGSVLKKEDAHVLFKV

DPTKVDSVYDMVTKKLGNHEEAPTV

>XP_025796153.1 transcriptional adapter ADA2 [Panicum hallii]

MGRSRGVPNSGDDDTGHRSKRRRVSSSGDATDTISAAMGGAGEGGGKKALYHCNYCNKDI

SGKIRIKCSKCPDFDLCVECFSVGAEVTPHRSNHPYRVMDNLSFPLICPDWNADEEILLL

EGIEMYGLGNWLEVAEHVGTKSKLQCIDHYTTAYMNSPCYPLPDMSHVNGKNRKELLAMA

KVQGESKKGTSLLPGELTPKAESPFSPSRVKLEEALGDGPAGRSPSHMAGGANKKASNAG

QIKDGANVSKVEDGHLDRSVGVKKPRYSADEGPSLTELSGYNSKRHEFDPEYDNDAEQAL

AEMEFKETDSETDRELKLRVLRIYLSRLDERKRRKEFILERNLLFPNPLEKDLTNEDKEV

YHRYKVFMRFLSKEEHEALVRSVIEERKIRRRIQELQECRSAGCRTLAEAKIHIEQKRKK

EYELNAQKSKESGQLIPNNKSVQKMNRPMKIESDGNLDPKKGGTGLDSPKTTGLTSVKQW

DDWDIVGLPGAELLSASEKLLCCQNRLLPSHYLRMQEVLMQEIFKGNVLKKEDAHVLFKV

DPTKVDTVYDMVTKKLGNHEEAPTV

>CAB3498190.1 unnamed protein product [Digitaria exilis]

MGRSRGVSNPGDDETGHRSKRRRVSSGGDATDTISAAMGGGGEGGGKKALYHCNYCNKDI

SGKIRIKCSKCPDFDLCVECFSVGAEVTPHRSNHPYRVMDNLSFPLICPDWNADEEILLL

EGIEMYGLGNWLEVAEHVGTKSKLQCIDHYTTAYMNSPCYPLPDMSHVNGKNRKELLAMA

KVQGESKKGTSLLPGELTPKAESPFSPSRVKLEDALGDGPAGRSPSQMAGGANKKASNAG

QIKDVANLSKVEDGHVDRSVGVKKPRYSADEGPSLTELSGYNSKRHEFDPEYDNDAEQAL

AEMEFKETDSETDRELKLRVLRIYLSRLDERKRRKEFILERNLLFPNPLEKELTNEDKEV

YHRYKVFMRFLSKEEHEALVRSVIEERKIRRRIQELQECRSAGCRTLAEAKIHIEHKRKK

EYELNAQKAKESGQLIPNNKSVQKMNRPVKIESDGNLDAKKGGAGLDSPKTSGPTSVKQW

DDWDIVGLPGAELLSASEKLLCCQNRLLPSHYLRMQEVLMQEMFKGSVLKKEDAHVLFKV

DPTKVDTVYDMVSKKLGNHEEAPTV

>NP_001105664.1 uncharacterized protein LOC542677 [Zea mays]

MGRSRGVLSSGDDDTGHRSKRRRVSSGGDATDSISASIGGAGEGGGKKALYHCNYCNKDI

SGKIRIKCSKCPDFDLCVECFSVGAEVTPHRSNHPYKVMDNLSFPLICPDWNADEEILLL

EGIEMYGLGNWLEVAEHVGTKSKLQCIDHYTSAYMNSPCYPLPDMSHVNGKNRKELLAMA

KVQGESKKGTLLLPGELTPKVESQFSPSRVKVEDALGEGPAGRSPSHMAVGANKKASNVG

HIKDGATVSKVEDVHVDRSVGVKKPRYSADEGPSLTELSGYNAKRHEFDPEYDNDAEQAL

AEMEFKETDSETDRELKLRVLRIYLSRLDERKRRKEFILERNLLFPNPLEKDLTSEDREL

YHRYKVFMRFLSKEEHEALVRSVIEERKIRRRIQELQECRSAGCRTLAEAKIHIEQKRKK

EYELNAQKAKDSSQLNANNKSVQKMNRPMKIESDGNLDPKKGGAGLDSPKTTGPTSVKQW

DDWDIVGLPGAELLSASEKLLCCQNRLLPSHYLRMQEVLMQEIFKGSVLKKEDAHVLFKV

DPTKVDSVYDMVSKKLGNHEEAPTV

>XP_004981760.1 transcriptional adapter ADA2 [Setaria italica]

MGRSRGVPNSGDDDTGHRSKRRRVSSSGDATDTISAAMGGAGEGGGKKALYHCNYCNKDI

SGKIRIKCSKCPDFDLCVECFSVGAEVTPHRSNHSYRVMDNLSFPLICPDWNADEEILLL

EGIEMYGLGNWLEVAEHVGTKSKQQCIDHYTTAYMNSPCYPLPDMSHVNGKNRKELLAMA

KVQGESKKGTSLLPGELTPKAESPFSPSRVKLEEALGDGPAGRSPSHMAGGANKKASNAG

QNKDGANISKVEDGHVDRSVGVKKPRYSADEGPSLTELSGYNSKRHEFDPEYDNDAEQAL

AEMEFKETDSETDRELKLRVLRIYLSRLDERKRRKEFILERNLLFPNPLEKDLTNEDKEV

YHRYKVFMRFLSKEEHEALVRSVIEERKIRRRIQELQECRSAGCRTLAEAKIHIEQKRKK

EYELNAQKAKESGQLIPNNKSVQKMNRPVKIESDGNLDPKKGGAGLDSPKTTGLTSVKQW

DDWDIVGLPGAELLSASEKLLCCQNRLLPSHYLRMQEVLMQEIFKGSVHKKEDAHVLFKV

DPTKVDTVYDMVTKKLGNHEEAPTV

>XP_010681286.1 PREDICTED: transcriptional adapter ADA2 isoform X2 [Beta vulgaris subsp. vulgaris]

MGRSRAVFCSSDDDPAQRSKRKRAASSGENTESASAGQSLSDAKNALYHCNYCNKDISGK

VRIKCVVCPDFDLCIECFSVGAEVHPHKSNHPYRVMDNLSFPLMCPDWNTDEEILLLEGI

EMYGLGNWAEVAEHVGTKSKSECIDHYNTIYMNSPCFPLPDLSHVVGKNREELLAMAKEQ

SDLKQVLTEDSMKEESAFSAKVKIEEPHKEAPDSQSSQGVKKPTNSTAVQDGSNGIKVED

SHPDRSVGEKKPRTSADDGPPVTDLCGYNPKRGEFETEYDNDAEQLLADMEFKETDTEAE

RELKLRVLRIYSRRLDERKRRKDFVLERNLLYPDPFEKNLTPEEKEICQRYRVFMRFHSK

DEHEELLRTVIEEHRIRKRILDLQEARAAGCRTSAEAQRYISRKRKIEADENTLKLRESV

EAGAGGKVLVRTTHHYRDSVSTTTATGQRMTNSLDEWDITGFPGADLLSETEKRLCGEIR

ILPSHYLNMLQTLSMEVMKGNITKKADAHSLFKVDPSKVDRVYDMLVKKGIAQQ

>XP_010686640.1 PREDICTED: transcriptional adapter ADA2b [Beta vulgaris subsp. vulgaris]

MGRSRAHFDEDPTQRSRRKKNASSGENIESALGGQGTGDGKKALYHCNYCNKDISGRIRI

KCAMCPDFDLCIECFSVGAEVTPHKSNHTYRVMDSLSFPLISPDWNADEEILLLEGLEMY

GLGNWAEVAEHVGTKNKEQCINHYRSVYLNSPFFPLPDMSHVVGKNRNELLAMAKENSED

RKGSTLFGDHIVKTEPTFSPSRIKVEELQKGGLSGSRLLSVQNSDSQVNPIGKKPPKEEE

SSLVSLSGFSLKRREFDPEYDIDAEQLLAEMEFKDADTEDERELKLRVLRIYSKRLDERN

RRKDFILERNLLYPSPFEKDLSPEEKTICRRYDPFMRFHSKEEHEDMLQSVIAEHRTRKR

ILELEEARAAGCRTSAEADKYFELKRKREAEAIARRANEATQLGPASQMGQNTAMASEAG

KDPVSLIHGQGQATFISANDLDIGRYPGEELLSEAEKQLCHETKIPPHLYLKMQETISVK

IFSGNVTSNLDAHSLFDIEPEKIDRIYDMLIRKGIAPP

>XP_021856198.1 transcriptional adapter ADA2-like [Spinacia oleracea]

MGRSRAVPSASDDDPTLKLKRKKTASSVENTESATAGQGLSDGKKALYHCNYCNKDISGK

IRIKCVVCPDFDLCIECYSVGAEVHPHKSNHPYRVMDSLSFPLICADWNADEETLLLEGI

DMYGLGNWAEVAEHVGTKSKSECIDHYNNIYMNSPCFPLPDLSRVAGKSREELFAMAKEH

SDRKLALTEELLKEVSTFSATVKIEEPDKEALGSQSSQAVKNPTNSTSVQDGSDGIKVED

SHPDRSVGEKKPRTSADEGPPVTDLCGYNSKREEFEVEYDNDAEQLLADMEFKETDTEAE

HELKLRVLRIYNRRLDERKRRKEFVLERNLLYPDPFEKNLTHEEKELCQRYRVFMRFHSK

QEHEELLKSVVEEHRLRRKIQDLEEAKAAGCRTSAEAERFIGLKRKIEAEEKTRKLRENV

EACPGGGKVLVRNGPHNRDFVSRDTISTTTGQFMTSSLDEWDITGFPGADLLSENEKRLC

GEIRMLPSHYVNMKQTLSIEVLKGNISKKSDAHGLFKVDPSKVDRVYDMLVKKGITQQ

>XP_021766757.1 transcriptional adapter ADA2b-like [Chenopodium quinoa]

MGRSRVFHSDEDPTQRSRRKKNVSSGESIESALGGQGTGDGKKALYHCNYCNKDITGRIR

IKCAMCPDFDLCIECFSVGVEMTPHKSNHPYRVMDSLSFPLISPDWNADEEILLLEGLEM

YGLGNWAEVAEHVGTKNKEQCINHYRSVYLNSPFFPLPDMSHVVGKNRNDLLAMAKENDE

DRKGSTLFGDHIVKTEPTFSPSRVKVDELPKGGPPSRLLSVQNADSHVNPIGKKPPKEED

SSLVSLSGFSLKRHEFDPEYDIDAEQLLAEMEFKDADTEDERELKLRVLRIYSKRLDERN

RRKDFIFERNLLHPSPFEKDLSPEEKAICRRYDPFMRFHSKEEHEDMLQAVIAEHRTHKR

ILELEEARAVGCRTSAEADKYFELKRKREAEESSRRAKESAQLGPASQLGQNAAMTSETV

KDPGSLIHELDIGRYPGEELLSEAEKQLCHETKISPHQYLKMQETISVKIFSGNVTSNVD

AHCLFDIEPGKIDRIYDMLIRKGVAPP

>XP_021755971.1 transcriptional adapter ADA2-like isoform X2 [Chenopodium quinoa]

MGRSRAVVPCPSDDDPNQKSKRKKAVSSVENTESVTAGQGSSDGKKALYHCNYCNKDISG

LIRFKCAVCPDFDLCVECFSVGAEVNPHKSNHPYRVMDSLSFPLICPDWNADEESLLLEG

IEMYGLGNWAEVAEHVGTKSKKECIDHYNNIYMNSPCFPLPDLSHVAGKNREELFAMAKE

HSNPIATGPALKEELSKEESINPGTVKIEEPQKEASGSHSSQAVEKPTNVLSAQDGSIKV

EDSHLDRSVGEKKPRASADEGPPVTDLCGYNPKREEFEVEYDNDAEHLLADMEFKDTDTE

AERDLKLKVLRIYNRRLDERKRRKDFVLERNLLYPDPFEKTLTPEEKELCQRYRAFMRYH

SKKEHEELLASVVNEHRIRKRLQDLQEAKAAGCRTSAEADIYIGRQRRMEAEENARKQRE

SAEAGAGGKVLLRNNRDFVSKDSASTTTGQFKTNSLDKWDITGFPGADLLSDKEKKLCSE

IRILPSHYLNMSQTLTMEAFKGNITKKSDAHGLFKVDPSKVDRVYDMLVKKGAISQ

>XP_021865335.1 transcriptional adapter ADA2b-like [Spinacia oleracea]

MGRSRVFHSDEDPTQRSRRKKNASSGESIESALGGQGIGDGKRALYHCNYCNKDITGRIR

IKCAMCPDFDLCIECFSVGAEMTPHKSNHAYRVMDSLSFPLISPDWNADEEILLLEGLEM

YGLGNWAEVAEHVGTKNKEQCINHYRSVYLNSPFFPLPDMSHVVGKNRNELLAMAKENSE

DRKGSTLFGDHIVKAEPTFSPSRVKVDELPKGGPPSRLLSVQNADSLVNPIGKKPPKEED

SSLVSLSGFSLKRHEFDPEYDIDAEQLLAEMEFKDADTEDERELKLRVLRIYSKRLDERN

RRKDFILERSLLYPSSFEKDLSPEEKSICRRYDPFMRFHSKEDHENMLHAIIGEHRTRKR

ILELEEARAVGCRTSDEADKYFDLKRKREAEDSSRRAKENAQNTAMTSDGGKDPASLIHG

QTTFISANELDIGRCPGEELLSDAEKQLCHETKISPYLYLKIEETISVKIFSGNVTNKVD

VHSLFDIEPEKIDRVYDMLIIKGIAPP

>XP_024980296.1 transcriptional adapter ADA2-like isoform X1 [Cynara cardunculus var. scolymus]

MGRSRAVNHSAEEDPSQSRSKRKRTASNLENLEAATSGQGMSEGKKALYHCNYCNKDISG

KIRIKCACCSDFDLCVECFSVGAEVYPHKSNHSYRVMDNLSFPLFCSDWNADEEILLLEG

IEMYGLANWNEVAEHVGTKSKSRCIEHYNTIYMNSPCFPLPDMSHVMGKNREELLAMARG

HGEATKGLPTGGELSVKEESPFSARIKVEDLRKEGSAGRSSSALASDVGSVGGSGTVNAS

AGAGKRTSSIVQSDKGGDGIKIEDSHADRSFGEKKLRTSAEEGPSITELSGYNFKRQEFE

IEYDNDAEQLLADMEFKDADTDPERELKLRVLRIYSKRLDERKRRKDFILERNLLYTDPF

EQGLSPEEKEICRRYRVFMRFHTKEEHEELLKTVIEEHRIRRRIEDLQEARAAGCRTSAD

AERYIEQKRKREAEENARRVKDNVQPGPSGKFLQRANHLKGDPDVGGATGKDSSSTCGGL

GIGNVDDWDVTGHFGADLLSDAEKRLCTEIRVLPVHYLSMLEKLSVEVLNGHIAQKSDAH

RLFNVEPSKVDRVYDMLLKKGIGQP

>XP_021998231.1 transcriptional adapter ADA2-like isoform X4 [Helianthus annuus]

MGRSRAVNHSTEEDSNQSRSKRKRTTSNLENLEAATSGQGMSEGKKALYHCNYCNKDISG

KIRIKCACCSDFDLCVECFSVGAEVYPHKSNHPYRVMDNLSFPLFCSDWNADEEILLLEG

IEMYGLANWNEVAEHVGTKSKSQCIEHYNTIYMNSPCFPLPDMSHVMGKNREELLAMARG

GEVAKGHQTGGELSVKEESPFSARIKVEDIRKENSAGRPLSGAGKRTSSIIPSDKSGDGI

KIEVPDSHTDRSFGEKKLRTSVEEGPSMTELSGYNFKRQEFEVEYDNDAEQLLADMEFKE

TDTDPERELKLRVLRIYSKRLDERKRRKDFILERNLLYGDPFEQGLSPEEKEICRRYRVF

MRFHTKEEHEELLKTIIEEHRIRKRIEDLQEARAAGCRGSADAERYIEQKRKREAEEKAR

GLKDNVLPGPSGKFLQRANHLKGDVDTGGKDSSAPGGLAIASLDDWDVSGHLGAELLSEA

EKRLCSEMKVLPTHYLGMLEKLTIEVLNGHIAQKSDAHRLFNVDPNKVDRVYDMLLKKGI

GQP

>KAD6794397.1 hypothetical protein E3N88_05293 [Mikania micrantha]

MGRSRANHSAEEDSNQSRSKRKRTASNLENLEAATSGQGMSDGKKALYHCNYCNKDISGK

IRIKCACCSDFDLCVECFSVGAEVYPHKSNHPYRVMDNLSFPLFCSDWNADEEILLLEGI

EMYGLANWNEVAEHVGTKSKSHCIEHYNTIYMSSPCFPLPDMSHVMGKNREELLAMARGH

GGEVSKGHPTGGELSVKEEFPFSARIKIEDLRKESPAGRPSSALASDSGSVGGSSSVNAT

TGAGKRTSSIAQSEKVGDGIKIEDSHTDRSFGEKKLRTSTEDGPSITELSGYNFKRQEFE

VEYDNDAEQLLADMEFKEADTDPERELKLRVLRIYSKRLDERKRRKDFILERNLLYADPF

EQGLSPEEKEICRRYRVFMRFHKKEEHEELLKTVIEEHRIRRRIEDLQEARASGCRTSAD

AERYIEQKRKREAEENTRRVKDNIPPGPSGKFLQRANHLKGDVDNRGSTLADTGGKDSFS

TPGGLAIANLDDWDVTGHLGADLLSEAEKRLCSEIKVVPVHYLSMLEKLTIEVMNGHINQ

KSDAHRLFNVDPSKVDRVYDMLLKKGIGQP

>XP_017258848.1 PREDICTED: transcriptional adapter ADA2 [Daucus carota subsp. sativus]

MGRSRAIPNPADDDPSQSRSKRKRTASNVENLESAATGQGTIEGKKALYHCNYCNKDISG

KIRIKCAMCSDFDLCVECFSVGAEVYPHKSNHPYRVMDNLSFPLICPDWNADEEILLLEG

IEMYGLGNWNEVAEHVGTKSRSQCIDHYNTIYMNSLCFPLPDMSHVMGKNREELLAMARE

QGEVKKGLPVPMELTVKEESSYPARVKIEDQSRDVLGRPLSSLVPEVGTGAGSSNGKPSA

GGVKRASSIVQSKVDSDGPKIEDRSVGEKKPRISGDEGPSLTELSGYNSKRQEFEIEYDN

DAEQLLADMEFKDTDSDAEREIKLRILRIYSKRLDERKRRKDFILDRNLLYPDPFEKDLT

PEEKEICQQFRVFMRFHMKEEHAEFLKVMIEEHRIRRRIQDLKEARAAGCRTSVEAERFI

EQKRKREAEEHARKAKENSQTGPSGKFLQRANHLKGELDNSPRGGPPGSVFEDWNVNGFP

GADLLSESERRLCSEIRILPAHYLKMLETLSIEVMKGRLSQKSDAHVLFDVDPGKVDKVY

DMLMRKGIVQP

>PWA57565.1 transcriptional adapter ADA2 [Artemisia annua]

MGRSRAVNHSAEEDSNQSRSKRKRTASNLENLEAATSGQVMSEGKKALYHCNYCNKDISG

KIRIKCACCSDFDLCVECFSVGAEVYPHKSNHPYRVMDNLSFPLFCADWNADEEILLLEG

IEMYGLANWNEVAEHVGTKSKANCIEHYNTIYMNSICFPLPDMSHVMGKNREELLAMARG

HGEAAKGPPTGGELSVKEESPFSARIKVEDLRKEGSAGRLSPALASDVGSVGGSSTINAS

SGAGKRPASGVQNDKGGDGVKLEDSHADRSFGEKKLRTSVEEGPSITEISGYNFKRQEFE

VEYDNDAEQLLADMEFKDTDTDAERELKLRVLRIYSKRLDERKRRKDFILERNLLYADPF

EQGLSPEEREICRRYRVFMRFHTKEEHAELLKTVIEEHRIRRRIQDLQEARAAGCQTSAD

AERYIEQKRKREAEENARRVKDNVQPGPSGKFLQRANHLKGDLDGSKDSSSTPGGLAINN

LDEWDVNGHLGADLLSEAEKRLCTEIRVLPAHYLNMLEKLTIEVLNGHIAQKSDAHRLFN

VDPNKVDKVYDMLLKKGIGQA

>KAA8519204.1 hypothetical protein F0562_013460 [Nyssa sinensis]

MGRSRGNFQPDEDPSQRSRRKKNTSSGENLESAVAGQGTGEGKRALYHCNYCNKDITGKI

RIKCVTCPDFDLCIECFSVGAEVKPHKSNHPYRVMDNLSFPLICPDWNADDEILLLEGIE

MYGMGNWAEISEHVGTKSKEQCIEHYTNAFMNSPYFPLPDMTHVIGKNRKELLAMAKGHG

EDKKGLSSLGELSLKEESPFSPSRVKVEDSHKGGPSGRLSSSLNAGAIKKASNMAQVKGG

LDSMKVEDNLSDKSLGGTKPKSSRDEGPLVEFSGFNPKRQEFDPEYDNDAEQLLAEMEFK

DTDTEEEHELKLRVLRIYSKRLDERYRRKGFILERNLLHPNSFEEDLSPEEKELCRCYDV

FMRFHSKEDHEDLLKTVISEHRILKRIQKLKEARAAGCHSSAEADRYFEQKRKRKAEDST

GRVNEISQAGPSSQGGLNVSMPLDSVSKDSNSRNAGQANSSSFTDLDIIAFPGAELLSES

EKRLCCEMRLTPPHYLKMQETMSVAIFNGSITKKSDAYPLFKLEPSKVDRVYDMLVKKGI

AQP

>KAA8537279.1 hypothetical protein F0562_027034 [Nyssa sinensis]

MGRSRGNFQPDEDPSQRSRRKKNASSGENLESSAAGQGTGEGKGALYHCNYCNKDISGKI

RIKCVTCPDFDLCIECFSVGAEVTPHKSNHPYRVMDNLSFSLICPDWNADNEILLLEGIE

MYGMGNWAEIAEHVGTKSKEQCIEHYMSAYMNSLYFPLPDMTHVVGKNRKELLAMAKGHG

EDKKGLSSLGELSLKDESPFSPSRVKVEDSHKGGPSGRLSSNLNPDIDFYSNTNIAGIGA

VKRAANMVQVKGGPDSKKEEDRSFGGKKPKSSRDEGPSLMEFSGYNPKRHEFDPEYDNDA

EQLLAEMEFKDIDTEEEHELKLRVLHIYSKRLDERKRRKDFILERDLLQPNTFEKELSPE

EKKLCRRYDVFMRFHSKVEHEDLLKTVISEHRVLKRIQELKEAQAAGCHSSTEAGRYLEQ

KKKREAQESACRTKESAQAGPSSQGGLIASLPLDSITKDLNSRTEGQANSGSLGDLEIMA

FPGADLLSESEKRLCREMRLPPPHYLKMQETISIAIFSGNITKKADAHPMFKIEPSKVDR

VYDMLVKKGIAHQ

>XP_028076478.1 transcriptional adapter ADA2b isoform X1 [Camellia sinensis]

MGRSRPNFHADEDPSQRSRRKKNASSGENLESTAAGQGTGDGKRALYHCNYCNKDITGKI

RIKCFMCPDFDLCIECFSVGAEVTPHKSNHPYRVMDNLSFPLICPDWNADEEILLLEGIE

MYGMGNWAEIAEHVGTKSREQCIQHYNTAYMNSPYFPLPDMTHVVGKNRKELLAMAKGLG

EDKKGLSSLGELTLKEESPFSPSRIKVEDSHKNGSSGRLLSSLNAGTTAIKKASTIAQVK

NSPNPIKVEDHDNRSFGGKKPKPSKDEGPSLIELSGYNSKRQEFDPEYDNEAEQLLAEMD

FKEADTEEERELKLRVLRIYSKRLDERQRRKEFILERNLLHPNPFEKDLSPEEKELCRRY

DVFMRFHSKEEHDDLLKTVVQEHRIMKRIQELKEARTAGCRSSDEAKRYFEQKRKRDAEE

SARSAKDSTRAGPSSQAGISVPTSTESVGKDSNSRSTGQANSTSLSELDIMAFPGADLLS

ESEKRLCCELRLPPPDYHKMQEIMTIQIISGSITKKSDAYPFFNKIEPSKVDRIYDMLVK

KGFAQS

>PSR98386.1 Transcriptional adapter like [Actinidia chinensis var. chinensis]

MGRSRGNFHADEDPSQRSRRKKNASSGENLESTAAGQGTGEGKKALYHCNYCNKDLTGKI

RIKCAMCPDFDLCIECFSVGAEVTPHKSNHLYSVMDNLSFPLICPDWNADEEILLLEGIE

MYGMGNWAEICEHVGTKSKEQCIQHYNTTYMNSPYFPLPDMTHVVGKNRKELLAMAKGHG

EDKKGLSSVGELTMKEESRFSPSRIKVEDSHKNGSSGRLLSVNAGSTALKKESDTPLVKN

SPNSIKVEDPLSDRSFGGKKPKPSKDEGPTLMELSGYNPKRHEFDPEYDNDAEQLLAEME

FKEADTEEERELKLRVLRIYSKRLDERKRRKDFILERDLLHPNPFKEDLSPEEKELCCRY

DVFMRFHSKEEHDDLLKTVVQEHRIMKRIQELKEARAAGCRSSDEANRYLEQKRKRDAEE

NARNVKENIQAGASGQGSVNLPTSFDSVYRDPNSRTLANSSSLTDLDTGAFPGADLLSES

EKQLCREIRLPPPDYLKIQEIITIQIMRGCITQKSDAYSFFNKIEPGKVDRVFDMVVKKG

FAE

>KAE9452772.1 hypothetical protein C3L33_15324, partial [Rhododendron williamsianum]

MGRSRGNFHADEDPSQRSRRKKNASSGENLESTVAELDEGQGTGDGKKALYHCNYCNKDI

TGKIRIKCAVCPDFDLCIECFSVGAEVTPHKSNHPYRVMDNLSFPLICPDWNADEEILLL

EGIEMYGMGNWAEIAEHVGTKSKQQCIQHYDTTYMNSPYFPLPDMTHVVGKNRKELLAMA

KGQGEDRKGLGELTLKEESLFSPSRIKIEELHKSGSSGRLPSVNAGSDLKKASNIPQVKN

SSNSVKVEDHLPDRNSGDKKPKPSKDEGPSLMELSGFNAKRQEFDPEYDNEAEQLLAEME

FKETDTEEERELKLRVLRIYSKRLDERKRRKDFILERNLLQPSAFENDLSPEEKDICRRY

DVFMRFHSKEEHDDLLKTVVQEHRILKRIQELKEARTAGCRSREEARSYFEQKRKREAEE

NACRAKENPQAGPSSQGGVNGLKSCNSVGKDSNSRTTDANSTSAMELLSESEKQLCREIR

LPPTDYLKMQELMTIQIMSGYIAKKSDAYSFFNKVEPSKVDRVYDMLVKKGLAQA

>PSS06458.1 Transcriptional adapter like [Actinidia chinensis var. chinensis]

MGRSRALPHSAEEDPNQSRSKRKRTAANVENLEPASTGRGMNEGKKALYHCNYCNKDISG

KIRIKCVVCPDFDLCMECFSVGAEVTPHKSNHSYRVMDNLSFPLICSDWNADEEILLLEG

IEMYGLGNWNEVAEHVGTKNKTQCIDHYNVIYMNSPCFPLPDMSHVMGKNREELLAMAKE

HGEVNKGFPTLGELTVKEEPPLSARVKVEDQRKEGSVGRSSSLISEIGTGAGAGSGKTSV

SEVKKASQINDGPEDIKEEDSHADRSIGEKKPRMSGDEGPSMKELSGYNSNRQEFEIEYD

NDAEQLLADMEFKGTDSDAERELKLRVLRIYSKRLDERKRRKEFILERDLLYPDPFEKNL

SPEEREVCRHYRVFMRFHSKEEHEELLKSVIEEHRILKRIQDLQEARAAGCRTSAQAERY

IEETRKKEAEETAHKLNDSGPAGPSDKFLQRANHLKGEPDSSPWGNARDSSLTSAGQANS

SFLEGWDISGFLGADLLSETEKQLCREIKILPSHYLSMSQTMSVAMLNGNITQKSDAHGL

FKVVDPSKVDRVYDMLVKKGIGQP

>XP_028115817.1 transcriptional adapter ADA2-like isoform X2 [Camellia sinensis]

MGRSRAIPHSAEEDHNQSRSKRKRNASNVENLEPATADQEIAEGKKALYHCNYCNKDISG

MIRIKCIMCPDFDLCVECFSVGAELNPHKSNHPYRVMDNLSFPLICSDWNADEEILLLEG

IEMYGLGNWTEVAEHVGTKSKAQCIDHYNAIYMNSPCFPLPDMSHVMGKNREELLAMAKE

HGEVNKGFPTLGELTVNDELLLSARIKVEDQRKEGSTGQSSSSLTSGAGVVAGNTSTSAV

KRASQIKDDHQDIKMEDSHADRSIGEKKLRTPGEEGPSMTRLSGYNPKRREFELEYDHDA

EQLLADMEFKESDTDAERELKLRVLRIYLKRLDERKRRKDFVLERNLLYPDPFEKNLSPE

EREICRRYRVFMRFHSKEVHEELLTSIIEEHRIMKIIQDLQEARAAGCRTSAQAEKYIEE

KRKKEAEESACKSKDSAQAGPSGKFLQRAIHLKGEPDSSPRGNARGLSLSDSFGKDSSST

TPGQATSSSLLDSWDVNGFLGADLLSESEKWLCSEIRILPSHYLSMLQTMSVGILNGNIT

TKSDAHGLFKAVDPSKVDRVYDMLVKKGIGSS

>XP_027088380.1 LOW QUALITY PROTEIN: transcriptional adapter ADA2b-like [Coffea arabica]

MGRSRGNFQAEEDPSQRSRRRKNATNSEHLESANAGQGTAEGKRALYHCNYCKKDITGRT

RIKCAACSDFDLCIECFSVGAEVYPHKSNHPYRVMDVLSFPLICPDWKADEEMLLLEGIE

MYGMWNWAEVGEHVGTKTKEACIEHFRNAYLNSPYFPLPDMTHVVAKNRKELLATAKSEE

KRGFSALGELTPKDESQFSPSRVKIEDQHISGPSGRLPSAVNAGTTGKKKASNKVQVRDR

HDSMKPKGKNFDNNRSNCSKDEGPSLMELSGYNXKRQEFDPEYDNDAEHLLADMEFKETD

TEEERKLKLRVLHIYWKRLDERKRRKDFILDRNLLHQDAFEKDLSQEEKVLCRRYDVFMR

FHSKEEHEELLKATVAEHRTLRRIQELKEAQAAGCHFSNDADRYLDWKRKKETELNGCDD

GGQYAFLNGPVASDSADAYSINLDFVSFSEAELLSASEKRLCGELRLAPAQYLKMVEVMT

TQIFSGNITKKSDAYSLFQIEPTKVDRVYDMLVKKGIAGPL

>XP_027185891.1 transcriptional adapter ADA2b [Coffea eugenioides]

MGRSRGNFQAEEDPSQRSRRRKNATNSEHLESANAGQGTAEGKRALYHCNYCKKDITGRT

RIKCAACSDFDLCIECFSVGAEVYPHKSNHPYRVMDVLSFPLICPDWKADEEMLLLEGIE

MYGMWNWAEVGEHVGTKTKEACIEHFRNAYLNSPYFPLPDMTHVVAKNRKELLATAKSEE

KRGFSALGELTPKDESQFSPSRVKIEDQHISGPSGRLPSAVNAGTTGKKKASNKVQVRDR

HDSMKPKGKNFDNNRSNCSKDEGPSLMELSGYNPKRQEFDPEYDNDAEHLLADMEFKETD

TEEERKLKLRVLHIYWKRLDERKRRKDFILDRNLLHQDAFEKDLSQEEKVLCRRYDVFMR

FHSKEEHEELLKATVAEHRTLRRIQELKEAQAAGCHFSNDADRYLDWKRKKETELNGCDD

GGNSIAFLTGPVASDSADAYSINLDFVTFSEAELLSASEKRLCGELRLAPAQYLKMVEVM

TTQIFSGNITKKSDAYSLFQIEPTKVDRVYDMLVKKGIAGPL

>XP_027087392.1 transcriptional adapter ADA2b-like isoform X1 [Coffea arabica]

MGRSRGNFQAEEDPSQRSRRRKNATNSEHLESANAGQGTAEGKRALYHCNYCKKDITGRT

RIKCAVCSDFDLCIECFSVGAEVYPHKSNHPYRVMDVLSFPLICPDWKADEEMLLLEGIE

MYGMWNWAEVGEHVGTKTKEACIEHFRNAYLNSPYFPLPDMTHVAKNRKELLATAKNEEK

RGFSALGELTPKDESQFSPSRVKIEDQHKSGPSGRLPSAVNAGTTGKKKASNKVQVRDRH

DSMKPKGKSFDNNRSNCSKDEGPSLMELSGYNPKRQEFDPEYDNDAEHLLADMEFKETDT

EEERKLKLRVLHIYWKRLDERKRRKDFILDRNLLHQDAFEKDLSQEEKVLCRRYDVFMRF

HSKEEHEELLKATVAEHRTLRRIQELKEAQAAGCHFSNDADRYLDWKRKKETELNGCDDG

GNSMAFLNGPVASDSTDAYSINLDFVSFSEAELLSASEKRLCGELRLAPAQYLKMVEVMT

TQIFSGNITKKSDAYSLFQIEPTKVDRVYDMLVKKGIAGPL

>XP_027170025.1 transcriptional adapter ADA2-like [Coffea eugenioides]

MGRSRAVPHSADEDLSQSRSKRKRTASNVENSEIATAGQGKAEGKALYHCNYCSKDISGK

IRIKCAICSNFDLCVECFSVGAEVHPHKSNHPYRVMDNLSFPLICPEWNADEEILLLEGT

EMYGLGNWTEVAEHVGTKSKLQCIDHYNVIYMNSPYFPLPDMSHVMGKNREELLAMAREH

GEIKKGSSAPGEIKEEFPFSAKIKVEDQKKDGQAGHSSSSLTSDSLVDRSIGEKKPRTSG

DEGPSVTEVSGYNSKRQEFEVEYDNDAEQLLADMEFKDTDTDAERELKLRVLRIYSKRLD

ERKRRKDFILERNLLYADTVEKELSHEEKDICHRYRVFMRFHSKEDHEELLRSLVEEHRV

LKRIQDLQEARAAGCRTSAEAERYIEQKRGEAEENALRISESSQAGPCGKFLQRANHLKG

DFDSSPRGVIRGPTVLDSAGIESPTAKRRLDVINALDNWDVTGFLGADLLSESEKQLCGE

IRILPAHYLNMLQTMSMGILSGNITKKSDAHGLFNVDPSKVDKVYDMLARKGIAQT

>CDP17436.1 unnamed protein product [Coffea canephora]

MGRSRAVPHSADEDLSQSRSKRKRTASNVENSEIATAGQGKAEGKALYHCNYCSKDISGK

IRIKCAICSNFDLCVECFSVGAEVHPHKSNHPYRVMDNLSFPLICPEWNADEEILLLEGT

EMYGLGNWTEVAEHVGTKSKLQCIDHYNVIYMNSPCFPLPDMSHVMGKNREELLAMAREH

GEIKKGSSAPGEIKEEFPFSAKIKVEDQKKDVQAGHSSSSLTSDSLVDRSIGEKKPRTSG

DEGPSVTEVSGYNSKRQEFEVEYDNDAEQLLADMEFKDTDTDAERELKLRVLRIYSKRLD

ERKRRKDFILERNLLYADTVEKELSHEEKDICHRYRVFMRFHSKEDHEELLRSLVEEHRV

LKRIQDLQEARAAGCRTSAEAERYIEQKRGEAEENALRISESSQAGPCGKFLQRANHLKG

DFDSSPRGVIRGPTVLDSAGIESPTAKRRLDVINALDNWDVTGFLGADLLSESEKQLCGE

IRILPAHYLNMLQTMSMGILSGNITKKSDAHGLFNVDPSKVDKVYDMLARKGIAQT

>XP_022848257.1 transcriptional adapter ADA2b-like isoform X1 [Olea europaea var. sylvestris]

MGRSRGNFRAEEDPSQRSRRKKNASSGENIESVTVGQGTSDGKRALYHCNYCNKDITGRI

RIKCALCSDFDLCIECFSVGAEVHPHKSNHPYRVMDILSFPLICPDWNADEEMLLLEGIE

MYGMGNWAEVAEHVGTKTKEVCIEHYRNAYLNSPCFPLPDMTHVVGKNRKELLAMAKGHL

EDKKGELPVKEESPFSPSRVKIEDSYKNGPLGRLPSASNTGTTDIKNASNMVQVKDQPDS

LKLDDHLSGRSFGSKKPKSADDEGPSLLEFSGYNTKRQEFDPEYDNDAEQLLADMEFKET

DTQEERELKLRVLHIYSKRLDERKRRKDFILERNLLHPSPFEEELSQEEKDLCRRYDVFM

RFHSKEEHEELLKTVVSEHRILKRIRELKEARAAGCRSSGEADRYLEHKRRREAEDSGHR

KESSQAGPSSQESLNVPVSSDSLNTYSNTTSVGHANSSSLTDLDFVPLSGADLLSESEKQ

LCREIRLPAPHYLRMMEFMTIQMMSGNINQKSDAYSFFQIEPTKIDRVYDMLLKKGMVQQ

>XP_011086259.1 transcriptional adapter ADA2b isoform X2 [Sesamum indicum]

MGRSRGNFHAEDDPSQRSRRKKSAQNGENLESVTAGQGSTDGKRALYHCNYCNKDITGRI

RIKCAVCSDFDLCIECFSVGAEVHPHKSGHPYRVMDILSFPLICPEWNADEEMLLLEGIE

MYGMGNWAEVAEHVGTKTKEVCIEHYRNAYLNSPYFPLPDMTHVVGKNRKELLAMAKEHF

EDKKGEIQLKEESPFSPSRVKVEDSYKTGSSGRLPSTSVAGAKGNKKASNMAHAKDQGDL

KMEDHMSGRSFGGNKPKSAKVEGPSLVDSSGYNPKRQEFDPEYDNDAEQLLADMEFKETD

TEEEREIKLRVLRIYSKRLDERQRRKNFILERNLLHPSPFEKDLSPEEKQLCRRYDVFMR

LHSKEDHEELLKTVVSEYRILKRIRELKEARAAGCRSAAEADRYFEQKKRETEDGDHRKE

NSQAGPSSQESLSVPVSSDSFGTYSTTTSAGQANSSTDLDFVTISAANLLSESEKQLCRE

IRLAPQHYLKMQEDMTTQIMIGNITKKSDAHSLFQIEAAKIDKVYDMLVKKGIVQS

>XP_022877888.1 transcriptional adapter ADA2b-like isoform X1 [Olea europaea var. sylvestris]

MGRSRGNFRGEEDPTQRSRRKKNASSGENIESVTSGQGTSDGKRARYHCNYCNKDITGRI

RIKCAVCSDFDLCIECFSVGAEVHPHKSNHPYRVMDILSFPLICPDWNADEEMLLLEGIE

MYGMGNWAEVAEHVGTKTKEVCIEHYRNAYLNSPYFPLPDMTHVVGKNRKELLAMAKEHI

EDKKGELPLKEESPFSPLRVKIENSYKNDPSGRLPSGSNGGIADNKMASNMVQVKDQPDS

LKLNDHLSGRSFGSNKPKSAEDEVPSLMESSGYNPKRQEFDTEYDNDAEQLLADMEFKET

ETQEEHELKLRVLRIYSKRLDERKRRKDFILERNLFHPSPFEEELSQEEKDLCRRYDVFM

RFHSKEEHEELLRTVISEHRILKRIRELKEARVAGCRSSGEADRYLEQKRRREAEDRGHR

KENSHAGPSSQESLNVPVSSDSLNTYSNTTSVGHSTSASLTDLDFVSLSGADLLSESEKQ

LCREIRLPAPHYLRMLEFMTIQMMSGNINQKSDAYSFFQIEPTKIDRVYDMLLKKGMLQE

>KZV35581.1 hypothetical protein F511_32747 [Dorcoceras hygrometricum]

MGRSRGNFHSEEDPSQRSRRKKNASSGENIESLAAGQVAGEGKRALYHCNYCNKDITGRI

RIKCGVCSDFDLCIECFSVGAEVHPHKSGHRYRVMDILSFPLICPEWNADEEMLLLEGIE

MYGMCNWAEVAEHVGTKTKEACIEHYKNAYLNSPHFPIPDMTHVVGKNRNELLAMAKGHL

DDKKGELQLKEESTFSPSRVKVEESYKSGPSGRLPSTSLAENFTGAKVKASNMAHIKDQG

DLLKMEDHMSGRSFGGNKPKSAKIEAPSLLDSGGYNAKRQEFDPEYDNDAEQLLADMEFK

ETDTEEERELKLRVLRIYSKRLDERKRRKDFILDRNILHPSPFLRELSQEEKELCRRYDV

FMRFHSKEEHEELIKTVVSEYRIQKRIQELKEARAAGCRSSAEADRYLEQKRREAEDGAC

LKESYQAGPSSQESLSVPVSSDSFGTYSNTPSAGPANSTHTDMDVVSFSATNCLSESEKQ

LCREIRLAPHDFLKLQAEMTIQFMIGNLSNKSDAYSFFQVDRTKIDGVYDLLLKKGLVQQ

>GER56332.1 transcriptional adapter ADA2 [Striga asiatica]

MGRSRGNFHAEEDPSQRSRRKKNASSGENLESVTAGQGTGDGKGALYHCNYCNKDITGRI

RIKCAVCSDFDLCVECFSVGGEVHPHKNGHPYRVMDILSFPLICPEWNADEEMLLLEGIE

MYGMGNWAEVAEHVGTKTKDACIEHYRNAYLNSPYFPLPDMTHVVGKNRNELLAMAKGHV

EDKIGDRQMKVESPFSPSRVKYRAKVNKKGSSTGHGKDNSDPLKMEDHLSGRSFGGNKPK

AAKVDAPSSTDASGYNAKREEFDPEYDNDAEQLLADMEFKEGEPEVERQLKLRVLRIYSK

RLDERKRRKDFILERNLLQYPNQYEGLSQEEKELCRRYDVFMRFHSKEEHEELLKTVVSE

HRTLKRIRELKEARAAGCRTSAEADRYHERKRRETDRKESSQAGPSSQESLSVPLSSDSF

GTYSTMTSAGQANSSTDSDFMSISGVNLLSESEKQLCREMKLAPQQYLKIQEDMTTNFLT

GNITKKSDAYSFFQVEPNKIDRIYDMLMRKGIIISQ

>XP_011085400.1 transcriptional adapter ADA2 isoform X1 [Sesamum indicum]

MGRSRAASHAAPDDPGQSRSKRKRGAQNVENTESAPPEVPGITDGKKALYHCNYCNKDIS

GKIRIKCVVCSDFDLCVECFSVGAEVHPHKSNHPYRVMDNLAFPLICPDWNADEEMLLLE

GIEMYGFGNWNEVAEHVGTKSKSQCIDHYDKIYMKSPCFPLPDMSHVMGKNREELLAMAK

EQNETKKGATTSGEGDGKEESSFSAGVKVEDQRKDGQAGRSSSSISSEVDTVGGTRCGKI

STGGSQRISSKVPSNDGPDAIKVEEFHADRSVGEKKPRASGDEGVSMKELSGYNSKRQEF

EIEYDNDAEQLLADMEFKETDTDAERELKLRVLHIYSKRLDERKRRKDFILERNLLYPDP

FEKDLTSEEKELCNRYRVFMRFHSKEEHDELLRSIVEEQRILKRIQDLQEARAAGCRTSA

EAERYIEQKMKREIEENARRVKESSQAGPSGKYLQRMNHHKGEQDSSPRGGNNSPSVLDP

GGKDSSTNTRGLNGSDPLDNWDVSGFLGADMLSEAEKQLCGEMRILPTHYLNMLQTMSIG

ILNGNVTKKSDAHGLFNVDPVKVDKVYDMLIRKGIAQT

>XP_012841942.1 PREDICTED: transcriptional adapter ADA2b isoform X1 [Erythranthe guttata]

MGRSRGNFHGGEDPSQRSRRKKNASSGENFESSSTGQGTTDGKGALYHCNYCNKDITGRI

RMKCAVCSDFDLCVECFSVGAEVHPHKSSHSYRVMDTLSFPLITPDWNADEEMLLLEGLE

MYGMGNWAEVAEHVGTKTKEMCIEHYRNAYLNSPYFPLPDMSHVDGKNRNELLAMGKGNL

QDKIGELKLEEETPFSPSRVKVEDSYKSGSSGRLPSAASSAGAKVNKKASNMSHAKDQGE

NPKTEGRSFGGNKPKSTKAKAPSLMDAIGYNPKRQEFDVEYDNDAEQLLADMEFKENDTE

VERELKIRVLRIYSKRLDERKRRKDFIVERNLLDPSPFGELSQEEKEIVRKYDPLMRLHT

KEEHEELIKSVISAHRIKRRMVELKVARAVGCRTSAEADRYFEQRRREAEDNARRKESSQ

AGQSSQESRNIPVSSDSFGTYSTTTSAGQGNSSTDLDSASVSAANLLSESEMKLCQEMGL

VPKQYLKMQERMSILILSGKIKTKSDAYAFFRIDPLKIDKVYNILMKKGIVQP

>PIN20368.1 Histone acetyltransferase complex SAGA/ADA, subunit ADA2 [Handroanthus impetiginosus]

MGRSRAVSQAAADDSGQSRSKRKRTTQNVENTESATPEIPGITDGKKATYHCNYCKSDIS

GTIRIKCALCSDFDLCVECFSVGAEVHPHKSNHSYRVMDNLAFPLLCPDWNADEEMLLLE

GLEMYGLWNWSEVAEHVGTKSKAQCIDHYNRTYMNSPCFPLPDMSHVMGKNREELIAMAK

ECSETKKGAATSGEVDGKEESLFPARIKVENQRKEGQAGLSSSSVSSEVDTVGASSCGKM

SAGATERTSSQVPANDGLDAMKVEELHADRSVGEKKPRTAGDDGVSMKELSGYNSKRQEF

EIEYDNDAEQLLADMEFKETDTDAERELKLRVLHIYSKRLDERKRRKDFILERNLLYPDP

FEKDLTLEEKELCRHYRVFMRFHSKEEHYELLRSVVAEHRIVKRIQHLQEARAAGCRTSA

EAERYIEQKIKRETEENARRVKESSQAGPSGKYLQRVNHHKGEQDTSPWGGNNNPSILDP

GGKDSLSNTGRLMGSDIADDWDVTGFEGADMLSEAEKQLCGEIRILPTHYLNMLQTMSMG

ILNGNLTKKSDAHGLFNVDPDKVDKVYDMLIRKGIAQA

>XP_012830156.1 PREDICTED: transcriptional adapter ADA2 [Erythranthe guttata]

MGRSRAASQAAVDDPGQSRSKRKRTTQNVDNTDSATPEFPGITDGKKALYHCNYCNKDIS

GKIRIKCVVCSDFDLCIECFSVGAEVYPHKSNHPYRVMDNLAFPLICPDWNADEEMLLLE

GLEMYGLGNWNEVAEHVGTKSKLQCIDHYDKVFMKSSCFPLPDMSHVMGKSREELLAMAK

EHGETKEGATTFGEVDGKEQSPFSARIKTEDQRKEGQSGRSSSISSEVDTIGGSGCGKMS

AGATKRMSSEVPSNDAPPDRVKVEEFHSDRSIGEKKPRTSGDEGVSMKELSGYNSKRQEF

EVEYDNDAEQLLADMEFKETDTETERELKLRVLHIYLKRLDERKRRKDFILERNLLYPDP

FEKDLTSEEKELCRRYRVFMRFHSKKEHDELLKSVVEEQRILKRIQNLQEARAAGCRTSS

EAERYIEQKMKREVELEESARRVKESSQAGPSGKYLQRMNNHHNNNTSPRVGNKSPSVLD

PGGMDSSSNTKQGGLAASDILSDNWDVTGFLGADILSQAEKQLCGEMRILPAHYLNMLQT

MSMGILNGNVTKKSDAHGLFNVDPGKVDKVYDMLIRKGIAQT

>TEY66568.1 transcriptional adapter 2-alpha [Salvia splendens]

MGRSRAASQATADDPGQSRSKRKRTAQNVENIDSAPPVPGITDGKKALYHCNYCNKDISG

KTRIKCVVCSDFDLCVECFSVGAEVFPHKSSHLYRVMDNLAFPLICPDWNADEEMLLLEG

IEMYGLANWSEVAEHVGTKSRSQCIEHYNRVYMNSPCFPLPDMSHVMGKNKEELLAMAKE

NDETKKGATMSGEVDGKEASPFAARIKMEDQKKEGQTGRSSSSISSGTSHTYGTGCLIIS

VPKSSCADEAGTIAGSSGAKMATGAGKRTSEKALSKDGLNVPKVEELHLDRSIGEKKPRT

SDNEGVSMKELSGYNSKRQEFEIEYDNDAEQLLADMEFKETDTDAERELKIRILHIYSKR

LDERKRRKDFILERNLLYPDPFERDLTMEEKELCHRYRVFMRFHSKEEHDELLRSVVEEQ

RILKRIEDLQCELHGEARAAGCRTACEAERFIEQKMKREFEENGHRVKESSQAGTGGKYL

QRINHQKGDQDTSSPRGGNKSPSVLDPVGKESSSNRRGLTGFDVSDKWDVSGFIGADILS

EAEKQLCVEIRILPAHYLNMLQTMSMRILSGNLTKKADAHGLFNVDPGKVDKVYDMLIKK

GIAQV

>XP_016554171.1 PREDICTED: transcriptional adapter ADA2b isoform X1 [Capsicum annuum]

MGRSRGNFQADEDPSQRSRRKKNASSGDNLESVIPDQGTTDGKKALYHCNYCNKDLSGRT

RIKCAVCSDFDLCIECFSVGAEVHPHKSYHHYRVMDILAFPLICPDWNADEEMLLLEGIE

MYGMGNWAEVGEHVGTKSKEACVDHFKDEYLDSPYFPLPDMTHVMGKNRKELLAMAKGNF

TDKKGVSSLGEVVPKDESFSPSQVKVEDSHRKGPSGRLSAVSNAGITGIKKPSSKSQIKI

QNEPVKLEGRNFGGKKPKSLKEDGSSLMKLSGYIPKREEFDPEYDNDAEQLLADMEFKET

ETEQERELKLRVLRIYSKRLDERKRRKDFILERNLLQPSEFEKNLSPEEKDLCRRYDAIM

RFLSKEEHEELMKTVVSEHRYLKRIQELKESRAAGCHSSIEVDRYLERKRKRELEDGVPR

KESSQIGPMSQESLNMPASSDSLGTHSNRKPCSQANLSSINDSGVAALSAGELLSEPEKQ

LCREIRLSPHHYLRMQEVLTIQIYSGNITRKSDAYPLFQIEATKVDRVYDMLLKKGVSPL

>XP_031118687.1 transcriptional adapter ADA2b [Ipomoea triloba]

MGRSRGNFHAEEDPSQRSRRKKNASNGENLDYITAGQGSGEGKPASYHCNYCNKDITGRI

RIKCAVCSDFDLCIECFSVGAEVQPHKSNHPYRVMDILSFPLICPDWNADEEMLLLEGIE

MYGIGKWAEVGEHVGTKTKDACIEHYRSAYLNSPYFPLPDMTHVVGRNREELLAMSKDNG

EDKKGFFSLGELARKNESPFSPSRVKVEDSNRSGSSGRLTSASNTGTTGIKKPSKKVLAK

DQTDSVKLEDNVSALNFGSKKPKLSKDEGPSLMEISGYNQKRHEFDPEYDNDAELLLADM

EFKDADTEEERELKLRVLRIYSKRLDERKRRKDFIIGRNLLYPTEFEKTLSQEEKDLWRR

YDAFMRFHSREEHEELLKAIILEHRTVKRIKELKEARAAGCRSSAEADRYFEQKRKREVE

ENGPKKENYHAGPSIQDSLSSLGTHSNKRSSSLANLSSLTDLEFAAHSAIELLSEPEKQL

CRDIRMPPPHYLKMLELMTVHIYSGDITKKSDAYTFFQIETTKVDRVYDMLLKKGIAPL

>PHT49062.1 Transcriptional adapter ADA2 [Capsicum baccatum]

MGRSRGIFQADEDPSQRSRRKKNASSGDNLESVIPDQGTTDGKKALYHCNYCNKDISGRT

RIKCAVCSDFDLCIECFSVGAEVHPHKSYHHYRVMDILAFPLICPDWNADEEMLLLEGIE

MYGMGNWAEVGEHVGTKSKEACVDHFKDAYLDSPYFPLPDMTHVMGKNRKELLAMAKGNF

TDKKGVSSLGEVVPKDESFSPSQVKVEDSHRKGPSGRLSSVSNAGITGIKKPSSKSQIKN

QNEPVKLEVNLLLVDNSGRNFGGKKPKSLKEDGSSLMKLSGYIPKREEFDPEYDNDAEQL

LADMEFKETETEQERELKLRVLRIYSKRLDERKRRKDFILERNLLQPSEFEKNLSPEEKD

LCRRYDAIMRFLSKEEHEELMKTVVSEHRYLKRIQELKESRAAGCHSSTEVDRYLERKRK

RELEDGVPRKESSQIGPMSQESLNMPASSDSLGTHSNRKPCSQANLSSINDSGVAALSAG

ELLSEPEKQLCREIRLSPHHYLRMQEVLTIQIYSGNITRKSDAYPLFQIEATKVDRVYDM

LLKKGVSPL

>TMW86970.1 hypothetical protein EJD97_020624 [Solanum chilense]

MGRSRGNFQADEDPSQRSRRKKNASSGDNLESVTTGQGTADGKRALYHCNYCNKDISGRT

RIKCAVCSDFDLCIECFSVGAEVHPHKSHHHYRVMDILAFPLICPDWNADEEMLLLEGIE

MYGMGNWAEVGEHVGTKTKEACIDHFKDAYLKSPYFPLPDMTHVMGKNRMELLAMAKGNF

TDKKGLSSLGDVAPKDESFSPSRIKVEDTHKIGPSGRLTSVSNAGITGIKKPSSKTLIKD

QNEPVKFEDNSGRNFGGKKPKSLKDDGSSLMKLSGYIPKRQEFDPEYDNDAEQLLADMEF

KENETEEERELKLRVLRIYSKRLDERKRRKDFILERNLLQPSEFEKNLSPEEKGICRRYD

VIMRFLSKEEHEELLKAVVSEHRYLKRIQELKEAKAAGCRSSAEVDRYLERKRKKEVEEG

VSRKGSSQIGPMSQESLNIPASSESLGIHSNRKPCSQAILSSDTNAGVPAFSAGELLSEP

EKQLCQEIRLSPHHYLRMQEVLTVQIYSGNITRKSDAYPLFQIEATKVDRVYDMLLKKGV

APL

>XP_019176987.1 PREDICTED: transcriptional adapter ADA2b isoform X1 [Ipomoea nil]

MGRSRGNFHAEEDPSQRSRRKKNASNGENLDYITAGQGSGEGKPASYHCNYCNKDITGRI

RIKCAVCSDFDLCIECFSVGAEVQPHKSNHPYRVMDILSFPLICPDWNADEEMLLLEGIE

MYGIGKWAEVGEHVGTKTKDACIEHYRSAYLNSPYFPLPDMTHVVGRNREELLAMSKENG

EDKKGFVSLGELARKNELPYSPSRVKLEDSNRSGSSGRLTSASNTGTAGIKKPSKKVLTK

DQTDSVKLEDNVSALNFGSKKPKLSKDEGPSLMEISGYNPKRHEFDPEYDNDAEQLLADM

EFKDADTEEERELKLRVLRIYSKRLDERKRRKDFITERNLLYPTEFEKTLSQEEKDIWRR

YDAFMRFHSRGEHEELLKAIISEHRILKRIKELKEARAAGCRSSAEADRYFEQKRKREVE

ENGPKKENYHVGPSSLDSLSSLGTHSNKRSSSLANLSSLTDLEFAAHSAIELLSEPEKQL

CRDIRMPAPHYLKMLELMTVHIYSGDITKKSDAYTFFQIETTKVDRVYDMLLKKGIAPL

>XP_015074692.1 transcriptional adapter ADA2b [Solanum pennellii]

MGRSRGNFQADEDPSQRSRRKKNASSGDNLESVTTGQGTADGKRALYHCNYCNKDISGRT

RIKCAVCSDFDLCIECFSVGAEVHPHKSHHNYRVMDILAFPLICPDWNADEEMLLLEGIE

MYGMGNWAEVGEHVGTKTKEACIEHFKDAYLKSPYFPLPDMTHVMGKNRMELLAMAKGNF

TDKKGLSSLGDVAPKDESFSPSRIKVEDTHKIGPSGRLTSVSNAGITGIKKPSSKTLIKD

QNEPVRFEDNSGRNFGGKKPKSLKDDGSSLMKLSGYIPKRQEFDPEYDNDAEQLLADMEF

KETETEEERELKLRVLRIYSKRLDERKRRKDFILERNLLQPSEFEKNLSPEEKGICRRYD

AIMRFLSKEEHEELLKAVVSEHRYLKRIQELKEAKAAGCRSSAEVDRYLERKRKKEVEEG

VPRKGSSQIGPMSQESLNMPASSESLGIHSNRKPFSQAILSSDTDSGVPAFSAGELLSEP

EKQLCQEIRLSPHHYLRMQEVLTVQIYSGNITRKSDAYPLFQIEATKVDRVYDMLLKKGV

APL

>XP_009803649.1 PREDICTED: transcriptional adapter ADA2-like isoform X3 [Nicotiana sylvestris]

MGRSRAVHQSADDDPSQRSKRKRTVPNVENFDTAAAGQVLSDGKKALYHCNYCNKDISGR

IRIKCAVCSDFDLCVECFSVGAEVQPHKSNHLYRVMDNLSFPLICADWNADEEMLLLEGM

EMYGLANWAEVAEHVGTKSKLQCIDHYNTIYINSPCFPLPDMSHVMGKSREELLAMAKDQ

GYAAPQEVNVKEESPFSARIKMEDQREENSAGLASGAGKRTSGVLHSKENHDSIKVEGCP

ADRSVGEKKPRTAMDEGPSMTELSGYNSKREEFEIEYDNDAEQMLADMEFKETDSNAERE

LKLRVLRIYYKRLDERKRRKDFILERNLLHPDPFEKDLTPEEKDICRCYRVFMRFNSKEE

HEDFLRSIIEEHRMVKRIQDLQDARAAGCRTSAEAERYIEQKRKRETEENLLRLKENSQS

GPSGKYLQRAGHFKVEHNSSPRGVATGPEILDSCYKDLSSTTAPHGVGSALDVWDVSGFF

GAELLSEAEKQLCDEIRILPAHYLNMLQTMSMGILNGNITKKSDAHGLFNVDPNKVDKVY

EMLVKKGIAQA

>XP_004239816.1 transcriptional adapter ADA2b isoform X1 [Solanum lycopersicum]

MGRSRGNFQADEDPSQRSRRKKNASSVDNLESATTGQGTADGKRALYHCNYCNKDISGRT

RIKCAVCYDFDLCIECFSVGAEVHPHKSHHHYRVMDILAFPLICPDWNADEEMLLLEGIE

MYGMGNWAEVGEHVGTKTKEACIDHFKDAYLKSPYFPLPDMTHVMGKNRMELLAMAKGNF

TDKKGLSSLGDVAPKDESFSPSRIKVEDTHKIGPSGRLTSVSNAGITGIKKPSSKTLIKD

QNEPVKFEDNSGRNFGGKKPKSLKDDGSSLMKLSGYIPKRQEFDPEYDNDAEQLLADMEF

KETETEEERELKLRVLRIYSKRLDERKRRKVFILERNLLQPSEFEKNLSPEEKGICRCYD

AIMRFLSKEEHEELLKAVVSEHRYLKRIQELKEAKAAGCRSSAEVDRYLERKRKKEVEEG

VPRKGSSQIGPMSQESLNIPASSESLGIHSNRKPCSQAILSSDTNAGVPAFSAGELLSEP

EKQLCQEIRLSPHHYLRMQEVLTIQIYSGNITRKSDAYPLFQIEATKVDRVYDMLLKKGV

APL

>XP_019245461.1 PREDICTED: transcriptional adapter ADA2b [Nicotiana attenuata]

MGRSRGNFQADEDPSQRSRRKKNASSGENLESVTVGQGTGEGKRALYHCNYCNKDISGRI

RIKCGVCSDFDLCIECFSVGAEVQPHKSHHPYRVMDILSFPLICPDWNADEEMLLLEGLE

MYGMGNWAEVGEHVGTKTKEACVEHFKSAYLESPYFPLPDMTHVIGKNRKELLAMAKGNF

TDKKGLSSLGEVVPKDESFSPSRVKVEDSHKNGPLGRLNSISNAGTTGINKPSSKAQAKD

QNVPVKLEDNHPGRNFGGKKPKSLKDDGSSLMKLSGYNAKRQEFDPEYDNDAEQLLADME

FKETETEEERELKLRVLRIYGKRLDERKRRKDFILERNLLQPSEFEKDLSPEERDICRRY

DAIMRFLSKEEHEELLKTVVSEHRFLKRIQELKEARAAGCRSSAEVERYIERKRKREVEE

GVPRKESSQIGPTSQESCQGNLSSITDSGIAAFSAGELLSEPEKQLCREIRIPPQHYLRM

QEVLTIQIFSGNITRKSDAYPLFQIEATKVDRVYDMLLKKGVAPL

>XP_006365843.1 PREDICTED: transcriptional adapter ADA2b-like [Solanum tuberosum]

MGRSRGNFQADEDPSQRSRRKKNASSGDNLETVTTGQGTADGKRALYHCNYCNKDISGRT

RIKCAVCSDFDLCIECFSVGAEVHPHKSHHHYRVMDILAFPLICPDWNADEEMLLLEGIE

MYGMGNWAEVGEHVGTKTKEVCIDHFKAAYLESPYFPLPDMTHVMGKNRMELIAMAKGNF

ADKKGLSSLGEDVPKDESFCPSRIKVEDTHKIGPSGRLTSVSNAGITGIKKPSSKTQIKD

QNEPVKFEDNSGRNFGGKKPKSLKDDGSSLMKLSGYIPKRQEFDPEYDNDAEQLLADMEF

KETETEEERELKLRVLRIYSKRLDERKRRKDFILERNLLQPSEFEKNLSPEEKDICRRYD

AIMRFLSKEEHEELLKTVISEHRYLKRIQELKEAKAAGCRSSAEVDRYLERKRKKEVEEG

VPRKGSSQIGPVIQESLNIPASSESLGIHSNRKPCSQAILSSDTDSGVPAFSAGELLSEP

EKQLCREIRLSPHHYLRMQEVLTVQIYSGNITRKSDAYPLFQIEATNVDRVYDMLLKKGV

APL

>XP_009766401.1 PREDICTED: transcriptional adapter ADA2b [Nicotiana sylvestris]

MGRSRGNFQADEDPSQRSRRKKNASSGENLESVTVGQGTGEGKRALYHCNYCNKDISGRI

RIKCGVCSDFDLCIECFSVGAEVQPHKSHHPYRVMDILSFPLICPDWNADEEMLLLEGLE

MYGMGNWAEVGEHVGTKTKEACVEHFKSAYLESPYFPLPDMTHVIGKNRKELLAMAKGNF

TDKKGHSSLGDVVPKDESFSPSRVKVEDSHKNGPLGRLNSVSNTGTTGIKKPSSKAQAKD

QNAPVKLEDNHPGRNFGGKKPKSLKDDGSSLMKLSGYNAKRQEFDPEYDNDAEQLLADME

FKETETEEERELKLRVLHIYGKRLDERKRRKDFILERNLLQPSEFEKDLSPEERDICRRY

DAIMRFLSKEEHEELLKTVVSEHRFLKRIQELKEARAAGCRSSAEVERYIERKRKREVEE

GVPRKESSQIGPTSQESCQGNLSSITDSGVVAFSAGELLSEPEKQLCREIRLPPQHYLRM

QEVLTIQIFSGNITRKSDAYPLFQIEATKVDRVYDMLLKKGVAPL

>XP_031105327.1 transcriptional adapter ADA2-like [Ipomoea triloba]

MGRSRAVPHSADDDPSQSRSKRKRTSQNVESLETGTAGQGLSDGKKASYHCNYCNKDISG

KIRIKCAVCSDFDLCIECFSVGAEVQPHKSNHPYRVMDNLSFPLICPDWSADEEILLLEG

IEMHGLGNWSDVAEHVGTKSKQQCVDHYNAIYMNSPCFPLPDMSHVMGKNREELLAMAKE

LGELKKGYTAVGDVNVKEETPFLGRVKMEDQRKEASTGRASSCLASADVVPGGAPTGAGK

RVSMGPSKDGHDGIKLEDSHADRSVGEKKPRNSGDEGPSMTELSGYNFKRQEFEIEYDND

AEQLLADMEFKDTDTDAERELKLRVLHIYLKRLDERKRRKDFILERNLLYPDPFEKDLNP

EEKEICRGYRVFMRFHSKEEHEVLLRSVIEEHRILRRIQDLQEARAAGCRTSAEAERYVE

QKRKREVEENTRRVKESMQTAANGKYVQRANSIKEEHDSSPRGGLRGPVVLDSSGKDFSS

TPSEHFVGNAVDNWDVSGFIGADLLSESEKQLCGEIRILPTHYLNILETLSTGILSGNIT

KKSDAHGLFNVDPSKVDRVYDMLVKKGFAPA

>RAL37892.1 hypothetical protein DM860_000586 [Cuscuta australis]

MGRSRANFHAEEDPTQRSRRKKNASSGENFDYIAAGQGAVDGKPASYHCNYCNKDITGRV

RIKCAMCSDFDLCIECFSVGVEVHPHKSNHPYRVMDILSFPLLCPDWNADEEMLLLEGIE

MYGIGKWAEVCEHVGTKTKDACIEHYTNTYLNSPYFPLPDMTHVVGRNIKELLDMTKENV

EDMKGFASLGEMAQKDGFVPRVKVEDANRSGSAGHFISASNAGIKKPSNVLAKDQTDSVK

LEGDIVSVVNLGSKKPKPLKDEGTWLMNISGYNPKRHEFDPEYDNEAEQLLADMEFKEND

TEEERELKLRVLRIYSKRLDERQRRKKFIIERNLLYPSELENTLSREEKDIWRHYDAFMR

FHSKQEHEELLEAIISEHRILKKIKDLKEARAAGCRSSAEADRYIERKRKMEAEENVPKK

ETYHAGPSSQKESMRALGTHPNKRSTSLANTTPITDLECAAVHSAIELLSEPEKQLCRDI

RMPPPHYLNMLEMMTVHIFSGNITKKSDAYTFFQIETTKVDRVYDMLLKKGVVPL

>XP_016472796.1 PREDICTED: transcriptional adapter ADA2b-like isoform X2 [Nicotiana tabacum]

MGRSRGNFQADEDPSQRSRRKKNASSGENLESVTVGQGTGEGKRALYHCNYCNKDISGRI

RIKCGVCSDFDLCIECFSVGAEVQPHKSHHPYRVMDILSFPLIRPDWNADEEMLLLEGLE

MYGMGNWAEVGEHVGTKTKEACVEHFKSAYLESPYFPLPDMTHVIGKNRKELLAMAKGNF

TDKKGLSSLGEVVPKDESFSPSRVKVEDSHKNGRLGRLNSISNADKTGTTGIKKPSSKAQ

AKDQNVPVKLEDNHLGRNFGGKKPKSLKDDGSSLMKLSGYNPKRQEFDPEYDNDAEQLLA

DMEFKETETEEERELKLRVLRIYGKRLDERKRRKDFILERNLLQPSEFEKDLSPEERDIC

RHYDAIMRFLSKEEHEELLKTVVSEHRFLKRIQELKEARAAGCRSSAEVERYIERKRKRE

VEEGVPRKESSQIGPTSQEICQGNLSSITDSGIAAFSAGELLSEPEKQLCREIRLPPQHY

LRMQEVLTIQIFSGNITRKSDAYPFFQIEATKVDRVYDMLLKKGVAPL

>XP_019249384.1 PREDICTED: transcriptional adapter ADA2-like [Nicotiana attenuata]

MGRSRAVHQSADDDPSQRSKRKRTVPNVENFDTAAAGQVLSDGKKALYHCNYCNKDISGR

IRIKCAVCSDFDLCVECFSVGAEVQPHKSNHLYRVMDNLSFPLICADWNADEEMLLLEGM

EMYGLANWAEVAEHVGTKSKLQCIDHYNTIYINSPCFPLPDMSHVMGKSREELLAMAKEQ

GYAAPQEVNVKEESPFSARIKMEDQREENSAGLASVGGSASGTLTGAGKRTSGVLHSKEN

HDSIKVEGCPADRSVGEKKPRPAMDEGPSMTELSGYNSKRGEFEIEYDNDAEQMLADMEF

KETDSNAERELKLRVLHIYYKRLDERKRRKDFILERNLLQPDPFEKDLTPEEKDICRCYR

VFMRFNSKEEHEDFLRSIIEEHRMVKRIQDLQDARAAGCRTSAEAERYIEQKRKRETGEN

LLRLKENSQSGPSGKYLQRAGHFKVEHNSSPRGVATGPEILDSCYKDLSSTTAPHGVGSA

LDVWDVSGFFGAELLSEAEKQLCDEIRILPAHYLNMLQTMSMGILNGNITKKSDAHGLFN

VDPNKLDKVYEMLVKKGIAQA

>XP_019193880.1 PREDICTED: transcriptional adapter ADA2-like [Ipomoea nil]

MGRSRAVPHSADDDPSQSRSKRKRTAQNVESLETGTAGQSLSDGKKASYHCNYCNKDISG

KIRIKCAVCSDFDLCIECFSVGAEVQPHKSNHPYRVMDNLSFPLICPDWSADEEILLLEG

IEMHGLGNWSDVSEHVGTKSKQQCVDHYNAIYMNSPCFPLPDMSHVMGKNREELLAMAKE

LGELKKGYTAVGDVNVKEETPFLGRVKMEDQRKEASTGRASSCLASADVVPGGAPTGAGK

RLSSMGPSKDGHDGIKLEDSHADRSVGEKKPRTSGDEGPSITELSGYNFKRQEFEIEYDN

DAEQLLADMEFKDTDTDAERELKLRVLHIYLKRLDERKRRKDFILGRNLLYPDPFEKDLT

PEEKEICRGYRVFMRFHSKEEHEVLLRSVIEEHRILRRIQDLQEARAAGCRTSAEAERYV

EQKRKREVEENTRRVKESMQTAANGKYVQRANSIKEEHDSSPRGGVRGPVVLDSSGKDFS

STPSENFVGNAVDNWDVSGFIGADLLSESEKQLCGEIRILPTHYLNILETLSTGILGGNI

TKKSDAHGLFNVDPSKVDRVYDVLVKKGFAPA

>XP_009609286.1 transcriptional adapter ADA2b isoform X2 [Nicotiana tomentosiformis]

MGRSRGNFQADEDPSQRSRRKKNASSGENLESVTVGQGTGEGKRALYHCNYCNKDISGRI

RIKCGVCSDFDLCIECFSVGAEVQPHKSHHPYRVMDILSFPLIRPDWNADEEMLLLEGLE

MYGMGNWAEVGEHVGTKTKEACVEHFKSAYLESPYFPLPDMTHVIGKNRKELLAMAKGNF

TDKKGLSSLGEVVPKDESFSPSRVKVEDSHKNGPLGRLNSISNADKTGTTGIKKPSSKAQ

AKDQNVPVKLEDNHSGRNFGGKKPKSLKDDGSSLMKLSGYNPKRQEFDPEYDNDAEQLLA

DMEFKETETEEERELKLRVLRIYGKRLDERKRRKDFILERNLLQPSEFEKDLSPEERDIC

RHYDAIMRFLSKEEHEELLKTVVSEHRFLKRIQELKEARAAGCRSSAEVERYIERKRKRE

VEEGVPRKESSQIGPTSQEICQGNLSSITDSGIAAFSAGELLSEPEKQLCREIRLPPQHY

LRMQEVLTIQIFSGNITRKSDAYPFFQIEATKVDRVYDMLLKKGVAPL

>XP_016580486.1 PREDICTED: transcriptional adapter ADA2 isoform X2 [Capsicum annuum]

MGRSRAVHQSTDDDPTQRSKRKRAAPNVENFDTAAAGQMLTEGKKALYHCNYCNKDISGR

IRIKCAVCSDFDLCVECFSVGAEVQPHKSNHLYRVMDNLSFPLICADWNADEEMLLLEGL

EMYGLANWAEVAEHVGTKSKLQCIEHYKSIYISSPCFPLPDMSHVMGKSREELLAMAKEQ

GYAAPGEVNVKEESPFSARIKMEDQREENSAGLASGCPADRSVGEKKPRTSVDEGPSMTE

LSGYNSKREEFEIEYDNDAEQMLADMEFKETDSNAERELKLRVLRIYSKRLDERKRRKDF

ILERKLLHPDPFEKDLTPEEKDICRSYRVFMRFSTKGEHEDFLRSIIEEHRMAKRIQNLQ

DARAAGCRTSAEAERYIEQKRTQESEENIRRLKENSQNGPSGKYLQRAGHFKVEHDSSPS

GVVRGPEMLDSCCKDLSSTTAPHGVGSALDIWDVSGFLGAELLSETEKKLCDEIRILPSH

YLNMLQTMSMGILNGNITKRSDAHGLFNVDPNKVDKVYEMLVKKGLAQA

>TMW95035.1 hypothetical protein EJD97_009458 [Solanum chilense]

MGRSRAVHQSTDDDPSQRSKRKRAAPNVESFDTAATGQILTEGKKALYHCNYCNKDISGR

IRIKCVVCSDFDLCVECFSVGAEVQPHKSNHLYRVMDNLSFPLICADWNADEEMLLLEGL

EMYGLANWAEVAEHVGTKSKQQCIDHYKSTYISSPCFPLPDMSHVMGKNREELLAMAKDQ

GYAAPGGVNVKEESPFSAGIKMEDQREENSTGLASVGGSASGTLAGAGKRTSSLLHSKEN

HDSIKVEGCPADRSVGEKKPRSSVDEGPSMTELSGYNSKREEFEIEYDNDAEQMVADMEF

KETDTNAERELKLRVLRIYNKRLDERKRRKDFILERKLLHPDPFEKDLTPEEKDICRRYR

VFMRFSSKEEHEDFLRSIIEEHRIVKRIRDLQDARIAGCRTLAEAERYVEQKRARESEEN

IRRLKENTQSGPSGKYLQRAGHFKVEHDSSPRGVGRGPEMMDCCNDLSSTTAPHGVGSAV

DIWDVSGFSGAELLSEAEKKLCDEMRILPAHYLNMSQTMSMGIFNGNITKKSDAHGLFNV

DPNKIDKVYEMLVKKGLAQA

>XP_015080359.1 transcriptional adapter ADA2-like isoform X1 [Solanum pennellii]

MGRSRAVHQSTDDDPSQRSKRKRAAPNVESFDTAATGQILTEGKKALYHCNYCNKDISGR

IRIKCVVCSDFDLCVECFSVGAEVQPHKSNHLYRVMDNLSFPLICADWNADEEMLLLEGL

EMYGLANWAEVAEHVGTKSKQQCIDHYKSTYISSPCFPLPDMSHVMGKNREELLAMAKDQ

GYAAPGGVNVKEESPFSAGIKMEDQREENSTGLASVGGSASGTLAGAGKRTSSLLHSKEN

HDSIKVEGCPADRSVGEKKPRSSVDEGPSMTELSGYNSKREEFEIEYDNDAEQMVADMEF

KETDTNAERELKLRVLRIYNKRLDERKRRKDFILERKLLHPDPFEKDLTPEEKDICRRYR

VFMRFSSKEEHEDFLRSIIEEHRIVKRIRDLQDARIAGCRTLAEAERYVEQKRARESEEN

IRRLKENTQSGPSGKYLQRAGHFKVEHDSSPRGVGRGPEMMDCCNDLSSTTAPNGVGSAV

DIWDVSGFSGAELLSEAEKKLCDEMRILPAHYLNMSQTMSMGIFNGNITKKSDAHGLFNV

DPNKIDKVYEMLVKKGLAQA

>XP_004243566.1 transcriptional adapter ADA2 isoform X1 [Solanum lycopersicum]

MGRSRAVHQSTDDDPSQRSKRKRAVPNVESFDTAATGQILTEGKKALYHCNYCNKDISGR

IRIKCVVCSDFDLCVECFSVGAEVQPHKSNHLYRVMDNLSFPLICADWNADEEMLLLEGL

EMYGLANWAEVAEHVGTKSKQQCIDHYKSTYISSPCFPLPDMSHVMGKNREELLAMAKDQ

GYAAPGGVNVKEESPFSAGIKMEDQREENSTGLASVGGSASGTLAGAGKRTSSLLHSKEN

HDSIKVEGCPADRSVGEKKPRSSVDEGPSMTELSGYNSKREEFEIEYDNDAEQMVADMEF

KETDTNAERELKLRVLRIYNKRLDERKRRKDFILERKLLHPDPFEKDLTPEEKDICRRYR

VFMRFSSKEEHEDFLRSIIEEHRIVKRIRDLQDARIAGCRTLAEAERYVEQKRARESEEN

IRRLKENTQSGPSGKYLQRAGHFKVEHDSSPRGVGRGPEMMDCCNDLSSTTAPHGVGSAV

DIWDVSGFSGAELLSEAEKKLCDEMRILPAHYLNMSQTMSMGIFNGNITKKSDAHGLFNV

DPNKIDKVYEMLVKKGLAQA

>XP_006367401.1 PREDICTED: transcriptional adapter ADA2-like isoform X1 [Solanum tuberosum]

MGRSRAVHQSTDDDPSQRSKRKRAAPNVESFDTAATGQILTEGKKALYHCNYCNKDISGR

IRIKCVVCSDFDLCVECFSVGAEVQPHKSNHLYRVMDNLSFPLICADWNADEEMLLLEGL

EMYGLANWAEVAEHVGTKSKLQCIDHYKSTYISSPCFPLPDMSHVMGKNREELLAMAKDQ

GYAAPGGVNVKEESPFSAGIKMEDQREENSTGLASVGGSASGTLAGAGKRTSSLLHSKEN

HDSIKVEGCPADRSVGEKKPRSSVDEGPSMTELSGYNSKREEFEIEYDNDAEQMVADMEF

KETDSNAERELKLRVLRIYNKRLDERKRRKDFILERKLLHPDPFEKDLTPEEKDICRRYR

VFMRFSSKEEHEDFLRSIVEEHRIVKRIRDLQDARIAGCRTLAEAERYVEQKRTRESEEN

VRRLKENSQSGPSGKYLQRAGHFKVEHDSSPRGVGRGPEMMDCCNDLSSTTAPHGVGSAV

DIWDVSGFSGAELLSEAEKKLCDEMRILPAHYLNMSQTMSMGIFNGNITKKSDAHGLFNV

DPNKVDKVYEMLVKKGLAQA

>RAL41874.1 hypothetical protein DM860_009056 [Cuscuta australis]

MGRSRAVPHPADDDSSQSRSKRKRAAQSAENSETGTAGQGLNDGKKALYHCNYCNKDISG

KIRIKCAVCSDFDLCIECFSVGAEIHPHKSNHPYRVMDNLSFPLISPDWSADEEILLLEG

IEMHGLGSWNDVAEHVGTKSKEQCVDHYNAIYMNSRCFPLPDMSHVRGKNREELLAMAKE

QDEVKKGYTDVGSVTIKEETPSSGRVMMEDQQTEASIGCASSCPASGDMVPCDARTGAGK

TVSSVAHINDGCDGIKLEDSHAYRSRGEKKPRIAGDEVPTVTEISGYNFKRQEFEIEYDN

DAEQLLADMEFKDTDTDAERKLKLRVLHIFLKRLDERKRRKDFILDRNLLYPDPFEKDLT

PEEKAICRAYRVFMRFHTKEEHDVLLKSVIEEHRILRRIQDLQAARAAGCRTSAEAERYI

EQKRKREIEENSRRGKENLQIVPNGKYVQRANNLKEEHVSSPRGGVRGPVVLDSSGKDFL

STPLEHLVGNAVDIWDVSGFIGADLLSEHEKQLCSEIRLLPTHYLGIMETLSTGILSGSI

TKKSDAHRLFNVDPTKVDKIYDMLIKKGLAQV

>PHU11830.1 Transcriptional adapter ADA2a [Capsicum chinense]

MGRSRPVRQSTDDDPTQRSKRKRAAPNVENFDTAAAGQMLTEGKKALYHCNYCNKDISGR

IRIKCAVCSDFDLCVECFSVGAEVQPHKSNHLYRVMDNLSFPLICADWNADEEMLLLEGL

EMYGLANWAEVAEHVGTKSKLQCIEHYKSIYISSPCFPLPDMSHVMGKSREELLAMAKEQ

GYAAPGEVNVKEESPFSARIKMEDQREENSAGLASVGGSASGKLAGAGKRTSAVLNSKEN

HDSIKLEGCPADRSVGEKKPRTSVDEGPSMTELSGYNSKREEFEIEYDNDAEQMLADMEF

KETDSNAERELKLRVLRIYSKRLDERKRRKDFILERKLLHPDPFEKDLTPEEKDICRSYR

VFMRFSTKGEHEDFLRSIIEEHRMAKRIQNLQDARAAGCRTSAEAERYIEQKRTQESEEN

IRRLKENSQNGPSGKYLQRAGHFKVEHDSSPSGVVRGPEMLDSCCKDLSSTTAPHGVGSA

LDIWDVSGFLGAELLSETEKKLCDEIRILPSHYLNMLQTMSMGILNGNITKRSDAHGLFN

VDPNKVDKVYEMLVKKGLAQA

>XP_004139515.1 transcriptional adapter ADA2b isoform X3 [Cucumis sativus]

MGRSRGNFQSDEDPTQRSRRKKNSSSGDNLESTTPGQGTTEGKKALYHCNYCIKDITGKI

RIKCAMCPDFDLCIECFSVGAELTPHKSNHPYRVMDNLSFPLICPDWNADDEILLLEGIE

MYGFWNWAEVAEHVGTKSKEQCIEHYSSVYMNSPYFPLPDMSHVVGKNRKELLAMAKGHG

EDKKGFSMLGELNLKAESPFSPSRVKVEDTHKVDPSGRLSSSSTSEEGSFNMATATANKK

ASSANQVKDSLVKVEDSQTDRIFKGKKPNIQANKGPSLLELSGYNEKRQEFDPEYDNEAE

QLLAEMEFKDADGEDERELKMRVLRIYSKRLDERKRRKDFILQRNLLYPSSFEKELSAEE

RAICRQYDVFMRFHSKEEHEELLQTIVAEHRTLKRIQDLKEARLAGYRTPAEAEIFLDKK

RKRESEEADRRVKDGNLTGPGSQGNSIMFIPSESAGKDSNSRPAVQALSGSVNDFDMLGF

NGADFLSEAEKRLCSEIRLTPPLYLRMEEVLSVEIFNGNVTKKSDAHHLFKIDPSKIDRI

YEMLIKKGIAQS

>TYK15464.1 transcriptional adapter ADA2b isoform X1 [Cucumis melo var. makuwa]

MGRSRGNFQSDEDPTQRSRRKKNSSSGDNLESTTPGQGTTEGKKALYHCNYCIKDITGKI

RIKCAMCPDFDLCIECFSVGAELTPHKSNHPYRVMDNLSFPLICPDWNADDEILLLEGIE

MYGFWNWAEVAEHVGTKSKEQCIEHYSSVYMNSPYFPLPDMSHVVGKNRKELLAMAKGHG

EDKKGFSMLGELNLKAESPFSPSRVKVEDTHKVDPSGRLSSSSTSEEGSFNMATATANKK

ASSANQVKDSLVKVEDSQTDRIFKGKKPNIQANKGPSLLELSGYNEKRQEFDPEYDNEAE

QLLAEMEFKDADGEDERELKMRVLRIYSKRLDERKRRKDFILQRNLLYPSSFEKELSAEE

RAICRQYDVFMRFHSKEEHEELLQTIVAEHRTLKRIHDLKEARLAGYRTPAEAEIFLDKK

RKRESEEAERRVKDGNLTGPGSQGNSIMFIPSESAGKDSNSRPAVQALSGSVNDFDMLGF

NGADFLSEAEKRLCSEIRLTPPLYLRMEEVLSVEIFNGNVTKKSDAHHLFKIDPSKIDRI

YEMLIKKGIAQS

>XP_023542054.1 transcriptional adapter ADA2b-like [Cucurbita pepo subsp. pepo]

MGRSRGNFQSDEDPTQRSRRKKNSSSGDNLESTTAGQGTTEGKKALYHCNYCIKDITGKI

RIKCAMCPDFDLCIECFSVGAELTPHKSNHPYRVMDNLSFPLICPDWNADDEMLLLEGIE

MYGFWNWTEVAEHVGTKSKEQCIEHYSSVYMNSPYFPLPDMSHVVGKNRKELLAMAKGHG

EDKKGYSILGELTLKEESPFSPPRVKVEDTHKVDPSGRLSSSATSEGSFNVATTTGNKKA

SSANQVKDGLVKMEDSQTDRNFKGKKPNLPANKGPSLIELSGYNEKRQEFDPEYDNEAEQ

LLAEMEFKDADGEDERELKMRVLRIYSKRLDERKRRKDFILQRNLLYPSSFEKDLSVEEM

AICRQYDVFMRFHSKEEHKELLKTVVAEHRTLKRIQELKEARAAGCRTPTEVEIFLDKKR

KRECDLTGPGSQGNSIMLMPSESGGKDSNSRAAVQGVSGSLNDLDTLGFNGADFLSEAEK

RVCSEIRVSPPVYLRMEEVLSVEIFNGNVSNKTDAHRLFKIEATKVDRIYEMLIKKGIAQ

P

>XP_022967070.1 transcriptional adapter ADA2b-like [Cucurbita maxima]

MGRSRGNFQSDEDPTQRSRRKKNSSSGDNLESTTAGQGATEGKKALYHCNYCIKDITGKI

RIKCAMCPDFDLCIECFSVGAELTPHKSNHPYRVMDNLSFPLICPDWNADDEMLLLEGIE

MYGFWNWTEVAEHVGTKSKEQCIEHYSSVYMNSPYFPLPDMSHVVGKNRKELLAMAKGHG

EDKKGYSILGELTLKEESPFSPPRVKVEDTHKADPSGRLSSSTMSEGSFNVAATTGNKKA

SSANQVKDGLVKMEDSQTDRNFKGKKPNLPANKGPSLLELSGYNEKRQEFDPEYDNEAEQ

LLAEMEFKDADGEDERELKMRVLRIYSKRLDERKRRKDFILQRNLLYPSSFEKDLSVEER

AICRQYDVFMRFHSKEEHKELLQTVVAEHRTLKRIQELKEARAAGCRTPTEVEIFLDKKR

KRECDLTGPGSRGNSIMFMPSESGGKDSNSRAAVQGVSGSLNDLDSLGFNGADFLSEAEK

RVCSEIRVSPPVYLRMEEVLSVEIFNGNVSNKSDAHRLFKIEATKIDRIYEMLIKKGIAQ

P

>XP_022142568.1 transcriptional adapter ADA2b [Momordica charantia]

MGRSRGNFQSDEDPTQRSRRKKNSSSGDNLESTTPGQGTSEGKKALYHCNYCIKDITGKI

RIKCAMCPDFDLCIECFSVGAELTPHKSNHPYRVMDNLSFPLICPDWNADDEILLLEGIE

MYGFWNWTEVAEHVGTKSKEQCIEHYSSVYMNSPYFPLPDMSHVVGKNRKELLAMAKGHG

EDKKGFSMLGELTLKEESPFSPSRVKVEDTHKVDPSGRLSSSSTSEDGSFNMAATTANKK

ASNTNQVKDSLVKVEDIKTDRNFKGKKPNLPANKGPSLLELSGYNEKRQEFDPEYDNEAE

QLLAEMEFKDADGEDERELKMRVLRIYSKRLDERKRRKDFILQRNLLYPSHFEKELSAEE

RAICRQYDVFMRFHSKEEHEELLQTVVAEHRTLKRIQELKEARAAGCRTANEAEIFLDKK

RKRESEETDCRVKEGTLTGPGSQGNSIMFMPSESASKDPNSRPANDFDTLGFNHADLLSD

AEKRLCSEIRLTPPLYLKMEEVLSVEIFNGNVTKKSDAHHLFKIDPIKIDRIYEMLIKKG

IAQP

>XP_022925352.1 transcriptional adapter ADA2b-like [Cucurbita moschata]

MGRSRGNFQSDEDPTQRSRRKKNSSSGDNLESTTAGQGTTEGKKALYHCNYCIKDITGKI

RIKCAMCPDFDLCIECFSVGAELTPHKSNHPYRVMDNLSFPLICPDWNADDEMLLLEGIE

MYGFWNWTEVAEHVGTKSKEQCIEHYSSVYMNSPYFPLPDMSHVVGKNRKELLAMAKGHG

EDKKGYSILGELTLKEESTFSPQRVKVEDTHKVDPSGRLSSSAMSEGSFNVAATTGNKKA

SSANQVKDGLVKMEDSQTDRNFKGKKPNLPANKGPSLIELSGYNEKRQEFDPEYDNEAEQ

LLAEMEFKDADGEDERELKMRVLRIYSKRLDERKRRKDFILQRNLLYPSSFEKDLSVEER

AICRQYDVFMRFHSKEEHKELLQTVVAEHRTLKRIQELKEARAAGCRTPTEVEIFLNKKR

KRECDLTGPGSQGNSIMFMPSESGGKDSNSRAAVQGVSGSLNDLDTLGFNGADFLSEAEK

RVCSEIRVSPPVYLRMEEVLSVEIFNGNVSNKSDAHRLFKIEATKVDRIYEMLIKKGIAQ

P

>XP_022135786.1 transcriptional adapter ADA2-like isoform X1 [Momordica charantia]

MGRSRMVSRLEDDDPNQSKPKRKRPNFTEATNSAAVGQESRDGKVALYHCNYCNKDLSGR

IRMKCVACPDFDLCVECFSVGAELTPHKSNHPYRVMDNLSFPLICPDWHADEEILLLEGI

AVYGFGNWDGVAEHVGTKSKSQCLNHYSAVYMNSPCFPLPDLSHVMGKSREELLAMATVP

GEVKKEFPMIGEHNLNEGSSPSARIKCEESKKEDSAHQTSSSGTAGSISGSTFSGAVKKS

NKPQIKDETKQGESEADRSFSEKKPRVLGDIGPSVVELSGYNFKRKEFDIEYDNDAEHLL

ADMEFKDTDSEADHELKLRILRIYSKRLDERKRRKDFILDRDLLYSDPFDKHLSPEERAI

CQPYKVFMRFHSKEDHEELLKNLIEEHRIVKRIQELQEARAAGCRTVAESNRLFDQKRKE

TRESNKRIKESSQAVPSDTVSNLLKGEHDDEGNVKESPRSQGSNKEPSSATTWISSSVHD

WDISGFSGVDLLSEMERRICCEIRILPAHYLKMVDIISVEMLKGSVTKKSDVHGLFKVDP

SKVDRVYDMVVKKGIAQA

>XP_022976656.1 transcriptional adapter ADA2a-like isoform X1 [Cucurbita maxima]

MGRSRMVSRPEDEDPNQSKSKRKRPSSTEATNQATVGQESRDGKVALYHCNYCNKDLSGR

IRMKCVACPDYDLCVECFSVGAELTPHKSNHPYRVMDNLSFPLLCPDWHADEESLLLEGI

AVYGFGNWDGVAEHVGTKSKLQCLNHYNAMYMNSPCFPLPDLSHVKGKSREELLAMATVP

GEVKKEFPMVGEHNLNEGSSLSARVKCEESKKEDSAHQTSSSGTAGSISGSTFSGAVKKS

NKPQIKDEIKQGESEADRSFSEKKPRVLGDSGPSVVELSGYNFKRKEFDIEYDNDAEHLL

ADMEFKDNDSAADHELKLRILRIYSKRLDERKRRKEFILDRDLLYSDPFEKHLSPEERAI

CQPYKVFMRFHSKEDHEELLKNLIEEHRIVKRIQELQEARAAGCRTIVESNRFLDQKRKE

TRDSSKRVKENSQAVPSDVSNHLKGEHDDNPRGSVKESPRSRGSGKEPSPATPWISSSIH

DWDITGFAGADLLSEMERRLCCEIRILPAHYLKMVDIISVEMLKGSVTKKSDVHGLFKVD

PSKVDRVYEMVVKKGIAQA

>XP_023536232.1 transcriptional adapter ADA2a-like isoform X1 [Cucurbita pepo subsp. pepo]

MGRSRMVSRPEDEDPNQSKSKRKRPSSTEATNQATVGQESRDGKVALYHCNYCNKDLSGR

IRMKCVACPDYDLCVECFSVGAELTPHKSNHPYRVMDNLSFPLLCPDWHADEESLLLEGI

AVYGFGNWDGVAEHVGTKSKLQCLNHYNAMYMNSPCFPLPDLSHVKGKSREELLAMATVP

GEVKKEFPMVGEHNLNEGSSLSARVKCEESKKEDSAHQTSSSGTAGSISGSTFSGAVKKS

NKPQIKDEIKQGESEADRSFSEKKPRVLGDSGPSVVELSGYNFKRKEFDIEYDNDAEHLL

ADMEFKDTDSAADHELKLRILRIYSKRLDERKRRKEFILDRDLLYSDPFEKHLSPEERAI

CQPYKVFMRFHSKEDHEELLKNLIEEHRIVKRIQELQEARAAGCRTIVESNRFLDQKRKE

TRDSSKRVKENSRAVPSDVSNHLKGEHDDNPRGSVKESPRSQGSGKEPSPATTWISSSIH

DWDISGFAGADLLSEMERRLCCEIRILPAHYLKMVDIISVEMLKGSVTKKSDVHGLFKVD

PSKVDRVYEMVVKKGIAQA

>XP_031744982.1 transcriptional adapter ADA2a isoform X3 [Cucumis sativus]

MGRSRMVSRPEDEDSNQSKSKRKRPSSTEATNPATVGQELGDGKAALYHCNYCNKDLSGR

IRIKCVACPDFDLCVECFSVGAELRPHKSNHPYRVMDNLSFPLLCPDWHADEESLLLEGI

AVYGFGNWDGVAEHVGTKSKLQCLNHYNAIYMNSPCFPLPDLSHVMGKSREELLAMATVP

EFPMAGEHNLNEGSSLSARVKCEESKKEDSAHQTSSSGTAGSISGSTFSGAVKKSNKPQI

KKETKQGESEADRSFSEKKPRVLGDSGPSVVELSGYNFKRKEFDIEYDNDAEHLLADMEF

KDTDSEADHELKLRILRIYSKRLDERKRRKDFILDRDLLYSDPFEKHLSPEERAICQPYK

VFMRFHSKEDHEELLKNLIEEHRIVKRIQELQEARAAGCRTIVESNKFLDQKRKETRESS

KRIKESSQGVPCEVSNHLKGEYDDIPRGNVKESPRSQGSGKDPSSTTPWMSTTVHNWDIS

EFAGADLLSEMERRLCCEIRILPAHYLKMVDIISVEMLKGSVTKKSDVHGLFKVDPSKID

RVYDMVVKKGISQA

>XP_022936263.1 transcriptional adapter ADA2-like isoform X1 [Cucurbita moschata]

MGRSRMVSRPEDEDPNQSKSKRKRPSSTEATNATVGQESRDGKVALYHCNYCNKDLSGRI

RMKCVACPDYDLCVECFSVGAELTPHKSNHPYRVMDNLSFPLLCPDWHADEESLLLEGIA

VYGFGNWDGVAEHVGTKSKLQCLNHYNAMYMNSPCFPLPDLSHVKGKSREELLAMATVPG

EVKKEFPMVGEHNLNEGSSLSARVKCEESKKEDSAHQTSSSGTAGSISGSTFSGAVKKSN

KPQIKDEIKQGESEADRSFSEKKPRVLGDSGPSVVELSGYNFKRKEFDIEYDNDAEHLLA

DMEFKDTDSAADHELKLRILRIYSKRLDERKRRKEFILDRDLLYSDPFEKHLSPEERAIC

QPYKVFMRFHSKEDQEELLKNLIEEHRIVKRIQELQAARAAGCRTIVESNRFLDQKRKET

RDNSKRVKENSQAVPSDVSNHLKGEHDDNPRGSVKESPRSQGSGKEPSPATTWISSSIHD

WDITGFAGADLLSEMERRLCCEIRILPAHYLKMVDIISVEMLKGSVTKKSDVHGLFKVDP

SKVDRVYEMVVKKGIAQA

>XP_016900505.1 PREDICTED: transcriptional adapter ADA2-like isoform X2 [Cucumis melo]

MGRSRMVSRPEDEDSNQSKSKRKRPSSTEATNPATGQESGDGKAALYHCNYCNKDLSGRI

RIKCVACPDFDLCVECFSVGAELRPHKSNHPYRVMDNLSFPLLCPDWHADEESLLLEGIA

VYGFGNWDGVAEHVGTKSKLQCLNHYNAIYMNSPCFPLPDLSHVMGKSREELLAMATVPG

EVKNEFPMAGEHNLNEGSSLSARVKCEESKKEDSAHQTSSSGTAGSISGSTFSGAVKKSN

KPQIKNETKQGESEADRSFSEKKPRVLGDSGPSVVELSGYNFKRKEFDIEYDNDAEHLLA

DMEFKDTDSEADHELKLRILRIYSKRLDERKRRKDFILDRDLLYSDPFEKHLSPEEREIC

QPYKVFMRFHSKEDHEELLKNLIEEHRIVKRIQELQEARAAGCRTIVESNKFLDQKRKET

RESSKRIKESSQAVPCEVSNHVKGECDDIPRGNVKESPRSQGSGKDPSSTTSWISSSVHD

WDISEFAGADLLSEMERRLCCEIRILPAHYLKMVDIISVEMLKGSVTKKSDVHGLFKVDP

SKIDRVYDMVVKKGISQA

>XP_027362435.1 transcriptional adapter ADA2b isoform X3 [Abrus precatorius]

MGRSRGNFHHADEDPNQRSRRKKNSVSGENSESGAAGQGTSEGKRALYHCNYCNKDITGK

IRIKCAMCPDFDLCIECFSVGAEVTPHKSNHPYRVMDNLSFPLICPDWNADDEILLLEGI

EMYGLGNWTEVAEHVGTKNKESCIEHYRNVYLNSPFFPLPDMTHVVGKNRKELLAMAKGQ

GEDKKGISMGDLSLKEDSPFSPSRVKVEDSHKADSQVDRDFGGKKPTSSGNEGPSLVESS

GYNSKRQEFDPEYDNDAEQLLAEMEFKDTDTEEERELKLRVLRFYSKRLDERKRRKDFIL

ERNLLYPNSFEKDLTTEEKTICQKYDFFMRFHTKEEHEELLRTVISEHRTRKRLRELKEA

RAAGCRNVMEADRYMVQKRRREAEESARRKKESAQVGPSNQGDLSARPAGPATSSSVNEM

DVTGYYGADLLSEPEKRLCCELRLPPATYLKMQEQLSLQILAGNVSAKSDAHQLFKIDTM

KIDRVYDMLIKKGIALP

>XP_020976293.1 transcriptional adapter ADA2b isoform X1 [0278]

MGRSRGNFHHADEDPSQRSRRKKNAASGENLEPGAAGQGASEGKRALYHCNYCNKDITGK

IRIKCAMCPDFDLCIECFSVGAEVTPHKSSHPYRVMDNLSFPLICADWNADDEILLLEGI

EMYGLGNWAEVAEHVGTKNKESCIEHYRNIYLNSPFFPLPDMSHVVGKNKKELLAMAKGQ

GEDKKGISMADGLKEESTFSPSRVKVEDSHKAGSSGPNQKASNSARGKDGLGVVKLEDSQ

VDRNFGGKKPNSSGNEGPSLVESSGYNSKRQEFDPEYDNDAEQLLAEMEFKDADTDDERE

IKLRVLRIYAKRLDERKRRKDFILERNLLYPNSFEKDLTPEEKAICRRYDIFMRFHKKEE

HEELLRTVISEHRTLKRLQDLKEARAAGCRNAAEAERYLLNKRRREADESARRVKESAQG

GPSNQGVPNALMSPDSAGKDMSGRPAGPATSSSVNEMDVTGYYGADLLSEPEKRLCCELR

LPPALYLKMQEQLSLQILSGTISAKSDAHQLFKMDAIKIDRVYDMLIKKGILRCCINYQA

LVWT

>XP_015958984.1 transcriptional adapter ADA2b isoform X2 [Arachis duranensis]

MGRSRGNFHHADEDPSQRSRRKKNAASGENLEPGAAGQGASEGKRALYHCNYCNKDITGK

IRIKCAMCPDFDLCIECFSVGAEVTPHKSSHPYRVMDNLSFPLICADWNADDEILLLEGI

EMYGLGNWAEVAEHVGTKNKESCIEHYRNIYLNSPFFPLPDMSHVVGKNKKELLAMAKGQ

GEDKKGISMADGLKEESTFSPSRVKVEDSHKAGSSGPNQKASNSARGKDGLGVVKLEDSQ

VDRNFGGKKPNSSGNEGPSLVESSGYNSKRQEFDPEYDNDAEQLLAEMEFKDADTDDERE

IKLRVLRIYAKRLDERKRRKDFILERNLLYPNSFEKDLTPEEKVICRRYDIFMRFHKKEE

HEELLRTVISEHRTLKRLQDLKEARAAGCRNAAEAERYLLNKRRREADESTRRVKESAQG

GPSNQGVPNALMSPDSASKDMSGRPAGPATSSSVNEMDVTGYYGADLLSEPEKRLCCELR

LPPALYLKMQEQLSLQILGGTISAKSDAHQLFKMDAIKIDRVYDMLIKKGIASP

>XP_025694061.1 transcriptional adapter ADA2b [Arachis hypogaea]

MGRSRGNFHHADEDPSQRSRRKKNAASGENLEPGAAGQGASEGKRALYHCNYCNKDITGK

IRIKCAMCPDFDLCIECFSVGAEVTPHKSSHPYRVMDNLSFPLICADWNADDEILLLEGI

EMYGLGNWAEVAEHVGTKNKESCIEHYRNIYLNSPFFPLPDMSHVVGKNKKELLAMAKGQ

GEDKKGISMADGLKEESTFSPSRVKVEDSHKAGSSGPNQKASNSARGKDGLGVVKLEDSQ

VDRNFGGKKPNSSGNEGPSLVESSGYNSKRQEFDPEYDNDAEQLLAEMEFKDADTDDERE

IKLRVLRIYAKRLDERKRRKDFILERNLLYPNSFEKDLTPEEKVICRRYDIFMRFHKKEE

HEELLRTVISEHRTLKRLQDLKEARAAGCRNAAEAERYLLNKRRREADESTRRVKESAQG

GPSNQGVPNALMSPDSASKDMSGRPAGPATSSSVNEMDVTGYYGADLLSEPEKRLCCELR

LPPALYLKMQEQLSLQILSGTISAKSDAHQLFKMDAIKIDRVYDMLIKKGIASP

>XP_019439999.1 PREDICTED: transcriptional adapter ADA2b-like isoform X3 [Lupinus angustifolius]

MGRSRGNFHPADEDPNQRSRRKKNAASGENLESGAAGQGASEGKRALYHCNYCNKDITGK

IRIKCAMCPDFDLCIECFSVGAEMIPHKSNHPYRVMDNLSFPFICPEWNADDEILLLEGI

EMYGLGNWTEVAEHVGTKNKESCIEHYKNVYLNSPFFPLPDMSHVVKKNREELLAMAKGQ

AEDKKGISMGDLGLKEESPFSPSRVKVEDSHKGGSSSRLLSSLNSDPQVDRNFGGKKPSS

SGNEGPSLVESSGYNQKRQEFDPEYDNDAEQLLAEMEFKDTDTEEDRELKLRVLRIYGKR

LDERKRRKDFILDRNLLYPNPFERDLTPEEKAICRKCDIFMRFHSKEEHEELLRTVIFEH

RTLKRLQELKEAQAAGCRNAAEADRYLVQKRRRVAQETARRAKETAQVGPITQGVPNALM

SPDSAGKDLNTRAAGPATSSSVNEMDVTGYYGADLLSEPEKRLCCELRLPPAVYLKMQER

LSVEILSGTVSTKSDAHRLFKMDTIKIDRVYDMLIKKGIGSP

>XP_004486337.1 transcriptional adapter ADA2b isoform X2 [Cicer arietinum]

MGRSRGNFHHADDDPSQRSRRKKNAASGDNLESGAAGQGASEGKRALYHCNYCNKDITGK

IRIKCAVCPDFDLCIECFSVGAEVTPHKSNHPYRVMDNLSFPLICPDWNADDEILLLEGI

EMYGMGNWAEVAEHVGTKNKEACIEHYRNVYLSSPYFPLPDMSHVVGKNRKELLAMAKGQ

GDDKKGVSMGDLSLKEETPYSPSRVKVEDSHKTGSNSRLASNMNSESDSAPSGNTHAASA

TNQKASNVGRGKGGPAIVKMEDSPVDRDFGGKKPNSSANEGSLVEASGYNVKRQEFDPEY

DNDAEQLLAEMEFKDTDTEDEREIKLRVLRIYSKRLDERKRRKDFILERNLLYPNPFEKD

LTPEEKTICRKYDIFMRFHTKEAHEELLRTVISEHRALKRIEELKEARAAGCRTSAEADI

YLANKRRKQSEESARRARENPQVIPNNHGVPNALMSPDSASKDLSTTRPAGPATSSSVNE

MDRTGYYGTDLLSEPEKRLCSELRLPPAVYLKMQEQLSIQMIAGNVTSKSDAHQMFKMDT

MKIDRVYDMLIKKGIGSP

>XP_006586771.1 transcriptional adapter ADA2b [Glycine max]

MGRSRGNFHHADEDPNQRSRRKKNAASGENSESGAAGQGAGEGKKALYHCNYCNKDITGK

IRIKCAMCPDFDLCIECFSVGAEVTPHKSNHPYRVMDNLSFPLICPDWNADDEILLLEGI

EMYGLGNWTEVAEHVGTKNKESCIEHYRNVYLNSPFFPVPDMSHVVGKNRKELLAMAKGQ

GEDKKGISMADLSIKAESSFSPSRVKVEDSHKAGSANRLASSLNSESDGPSGNTHAANQK

ASNVGRGKGGPGIIKMEDSQLDRDFGGKKPTSSGNEGPSLVESSGYNAKRQEFDPEYDND

AEQLLAEMEFKDTDTDDERELKLRVLRFYAKRLDERKRRKDFILERNLLYPNPFEKDFTP

EEKAICRNYDLFMRFHTKEEHEELLRTVISEHRTRKRLQDLKEARAAGCRNSAEADRYLA

QKRKREAEESARRTKESAQGGPSNLGVSNALMSPDSAGKDLRGRPAGPATSSSVNEMDVT

GYYGADLLSESEKRLCCELRLPPAMYLKMQEQLSLQILAGTVTAKSDAHQLFKMDAMKID

RVYDILIKKGIGSP

>XP_020221941.1 transcriptional adapter ADA2b [Cajanus cajan]

MGRSRGNFHADEDPHQRSRRKKNAASGENLESGASGQGAGDGKKALYHCNYCNKDITGKI

RIKCAVCPDFDLCIECVSVGAEVQPHKNNHPYRVMDNLSFPLICPDWNADDEILLLEGIE

MYGLGNWAEVAEHVGTKNKETCIEHYTNVYSNSPFFPVPDMSHVVGKNRKELLAMAKGQG

EDKKGFSMGDLSLKAESSFSPSRAKVEDSHKAGSANRLNSESDSGPLGNTHGATGANQKA

SNVGRGKGGVIKMEDSQIDRDFGGKKPTSSGNEGPSLVELSGYNAKRQEFDPEYDHDAEQ

LLAEMEFKDTDTDDERELKLRVLHYFSKRLDERKRRKDFILERNLLYPNPFEKDLTPEEK

TVCRKYDLFMRFHSKEEHEELIRIVISEHRTRKRLQELKQARAAGCRNSAEADKYLLHKR

KREAEESARRTKESPQVGPSNQGVPNALMSPDSAGKDLSARPAGPATSSSVNEMDVTGYY

GADLLSEPEKRLCCELRLPPAIYLKMQEQLSLQILAGTVSAKSDAHQLFKMDAMKIDRVY

DMLIKKGIGSP

>KAE9599115.1 putative transcription factor MYB/SANT family [Lupinus albus]

MGRSRGNFHPADEDPNQRSRRKKNAASGENLESGAAGQGASEGKRALYHCNYCNKDITGK

IRIKCAMCPDFDLCIECFSVGAEVTPHKSNHPYRVMDNLSFPFICPEWNADDEILLLEGI

EMYGLGNWTEVAEHVGTKNKESCIEHYKNVYLSSPFFPLPDMSHVVKKNREELLAMAKGQ

AEDKKGISMGDLGLKEESPFSPSRVKVEDSHKGGSSSRLLSSLNSESESGSAGNQKASNS

ARVKDGPGVIKMEDPQVDRNFGGKKPSSSGNEGPSLVESSGYNQKRQEFDPEYDNDAEQL

LAEMEFKDTDTEEERELKLRVLRIYGKRLDERKRRKDFILDRNLLYPNPFEKDLTPEEKA

ICRKYDLFMRFHSKEEHEELLRTVIFEHRTLKRLQELKEAQAAGCRNAAEADRYLVQKRR

RLAEETARRAKETAQVGPSSQGIPNALMSPDSAAKDLNTRAAGPATSSSVNEIDITGYYG

ADLLSEPEKRLCCELRLPPAVYLKMQERLSVEILSGTVSTKSDAHRLFKMDTIKIDRVYD

MLVKKGIGSP

>XP_028204769.1 transcriptional adapter ADA2b-like [Glycine soja]

MGRSRGNFHHADEDPNQRSRRKKNAASGENSESGAAGQGAGEGKKALYHCNYCNKDITGK

IRIKCAMCPDFDLCIECFSVGAEVTPHKSSHPYRVMDNLSFPLICPDWNADDEILLLEGI

EMYGLGNWTEVAEHVGTKNKESCIEHYRNVYLNSPFFPVPDMSHVVGKNRKELLAMAKGQ

GEDKKGISMGDLSIKAESSFSPSRAKVEDSHKAGSSNRLASGLNSESDGPLGNTHAANQK

ASNVGRGKGGPGIIKMEDSQLDRDFGGKKPTSSGNEGPSLVESSGYNAKRQEFDPEYDND

AEQLLAEMEFKDTDTDDERELKLRVLRFYAKRLDERKRRKDFILERNLLYPNPFEKDLTP

EEKTICRKYDLFMRFHTKEEHEELLRTVISEHRTRKRLQELKEARAAGCRNSAEADRYLA

QKRRREAEESGCRTKESAQGGPSNQGVPNALMSPDSAGKDLSGRPAGPATSSSVNEMDVT

GYYGADLLSEPEKRLCCELRLPPAMYLKMQEQLSLQILAGTVTAKSDAHQLFKMDAMKID

RVYDMLIKKGIGSP

>XP_017434027.1 PREDICTED: transcriptional adapter ADA2b [Vigna angularis]

MGRSRGNFHHADEDPNQRSRRKKNAASGENSESGAAGQGAGEGKKALYHCNYCNKDITGK

IRIKCAMCPDFDLCIECFSVGAEVTPHKSNHPYRVMDNLSFPLICPDWNADDEILLLEGI

EMYGLGNWTEVAEHVGTKNKESCIEHYKNIYLSSPFFPVPDMSHVVGKNRKELLAMAKGQ

GEDKKGISMGDLSLKAESPFSPSRAKVEDSHKAGTANRLSSNLNSELDSGPSGNTHSAAG

ANQKASNVTRGKGGPGVIKMEDSQIDRDFGGKKPTSSGNEGPSLVELSGYNAKRQEFDPE

YDNDAEQLLAEMEFKDTDSEEERELKLRVLRLYSKRLDERKRRKDFILERNLLYPNPLEK

DLTAEEKAMCRKYDIFMRFHSKEDHDELLRTMLFEHRSWKKIQELKEARLAGCRNSADAE

KYISLKRKREAEENARRTRTKESAQVGPSNQGIPNALTSPDSAGKDLSARPAGPATSSSV

NEMDAIGYNGADLLSEAEKRLCCELRLSPATYLKMQEQLSLQILAGTVSAKSDAHQLFKL

DAMKVDRVYDMLIKKGIGLP

>TKY72253.1 Transcriptional adapter ADA2 [Spatholobus suberectus]

MGRSRGNFHHADEDPNQRSRRKKNAASGENSESGAAGQGAGDGKKALYHCNYCNKDITGK

IRIKCAMCPDFDLCIECFSVGAEVTPHKSNHPYRVMDNLSFPLICPDWNADDEILLLEGI

EMYGLGNWAEVAEHVGTKNKESCIEHYRNVYLNSPFFPVPDMSLVVGKNRKELLAMAKGQ

GEDKKGISMGDLSIKAESTFSPSRAKVEDSHKAGSTNRLPSSLNSESDSGPSGNIHAAAG

ANQKASNVARGKGGPGIVKMEDSQIDRDFGGKKPTSSGNEGPSLVESSGYNPKRQEFDPE

YDNDAEQLLAEMEFKDTDTEDERELKLRVLRFYSKRLDERKRRKDFILERNLLYPNPFEK

DLTPEERTICRKYDLFMRFHTKEEHEELLRTVISGHRTRKRLQELKEARAAGCRNSAEAD

RYLAHKRRREAEESARRTKESAQVGPSNQGVPNALMSPDSAGKDLSARPAGPATSSSVNE

MDLTGYYGADLLSEPEKRLCCDLRLPPAVYLKMQEQLSLQILAGTVSAKSDAHQLFKMDA

MKIDRVYDMLIKKGIGSP

>XP_027933407.1 transcriptional adapter ADA2b isoform X2 [Vigna unguiculata]

MGRSRGNFHHADEDPNQRSRRKKNAASGENSESGAAGQGAGEGKKALYHCNYCNKDITGK

IRIKCAMCPDFDLCIECFSVGAEVTPHKSNHPYRVMDNLSFPLICPDWNADDEILLLEGI

EMYGLGNWTEVAEHVGTKNKESCIEHYKNIYLSSPFFPVPDMSHVVGKNRKELLAMAKGQ

GEDKKGISMGDLSLKAESPFSPSRAKVEDSHKAGTANRLSSNLNSDSGPSGNTHSAAGAN

QKASNVARGKGGPGVIKMEDSQIDRDFGGKKPTSSGNEGPSLVELSGYNAKRQEFDTEYD

NDAEQLLAEMEFKDTDSEEERELKLRVLRLYSKRLDERKRRKDFILERNLLYPNPLEKDL

TPEEKAMCRKYDIFMRFHSKEDHDELLRTMIFEHRSWKKIQELKEARLAGCRNSADAEKY

ISLKRKREAEENARRTRTKESAQVGPSNQGIPNALTSPDSASKDLSARPAGPATSSSVNE

MDAIGYSGADLLSEAEKRLCCELRLSPATYLKMQEQLSLQILAGTVSAKSDAHQLFKLDA

IKVDRVYDMLIKKGIGLP

>XP_014516922.1 transcriptional adapter ADA2b [Vigna radiata var. radiata]

MGRSRGNFHHADEDPNQRSRRKKNAASGENSESGAAGQGAGEGKKALYHCNYCNKDITGK

IRIKCAMCPDFDLCIECFSVGAEVTPHKSNHPYRVMDNLSFPLICPDWNADDEILLLEGI

EMYGLGNWTEVAEHVGTKNKESCIEHYKNIYLSSPFFPVPDMSHVVGKNRKELLAMAKGQ

GEDKKGISMGDLSLKAESPFSPSRAKVEDSHKAGTANRLSSNLNSELDSGPSGNTHSAAG

ANQKASNVTRGKGGPGVIKMEDSQIDRDFGGKKPTSSGNEGPSLVELSGYNAKRQEFDPE

YDNDAEQLLAEMEFKDTDSEEERELKLRVLRLYSKRLDERKRRKDFILERNLLYPNPLEK

DLTPEEKAMCRKYDIFMRFHSKEDHDELLRTMLFEHRSWKKIQDLKEARLAGCRNSSDAE

KYISSKRKREAEENARRTRTKESAQVGPSNQGIPNALTSPDSAGKDLSARPAGPATSSSV

NEMDGIGYNGADLLSEAEKRLCCELRLSPATYLKMQEQLSLQILAGTVSAKSDAHQLFKL

DAMKVDRVYDMLIKKGIGLP

>QCD77231.1 transcriptional adapter 2-alpha [Vigna unguiculata]

MGRSRGNFHHADEDPNQRSRRKKNAASGENSESGAAGQGAGEGKKALYHCNYCNKDITGK

IRIKCAMCPDFDLCIECFSVGAEVTPHKSNHPYRVMDNLSFPLICPDWNADDEILLLEGI

EMYGLGNWTEVAEHVGTKNKESCIEHYKNIYLSSPFFPVPDMSHVVGKNRKELLAMAKGQ

GEDKKGISMGDLSLKAESPFSPSRAKVEDSHKAGTANRLSSNLNSESDSGPSGNTHSAAG

ANQKASNVARGKGGPGVIKMEDSQIDRDFGGKKPTSSGNEGPSLVELSGYNAKRQEFDTE

YDNDAEQLLAEMEFKDTDSEEERELKLRVLRLYSKRLDERKRRKDFIFERNLLYPNPLEK

DLTPEEKAMCRKYDIFMRFHSKEDHDELLRTMIFEHRSWKKIQELKEARLAGCRNSADAE

KYISLKRKREAEENARRTRTKESAQVGPSNQGIPNALTSPDSASKDLSARPAGPATSSSV

NEMDAIGYSGADLLSEAEKRLCCELRLSPATYLKMQEQLSLQILAGTVSAKSDAHQLFKL

DAIKVDRVYDMLIKKGIGLP

>XP_007147630.1 hypothetical protein PHAVU_006G140900g [Phaseolus vulgaris]

MGRSRGNFHHADEDPNQRSRRKKNAASGENSESGAAGQGAGEGKKALYHCNYCNKDITGK

IRIKCAMCPDFDLCIECFSVGAEVTPHKSNHPYRVMDNLSFPLICPDWNADDEILLLEGI

EMYGLGNWTEVAEHVGTKNKESCIEHYKNIYLSSPFFPVPDMSHVVGKNRKELLAMAKGQ

GEDKKGISMGDLSLKAESPFSPSRAKVEDSHKAGSTNRLSSNLNSELDSGPSGNTHSAAG

ANQKASHARGKGGPGIIKMEDSQIDRDFGGKKPTSSGNEGPSLVELSGYNAKRQEFDPEY

DNDAEQLLAEMEFKDTDSEEERELKLRVLRLYSKRLDERKRRKDFILERNLLYPNPLEKD

LTPEEKAICRKYDIFMRFHSKEDHDELLRTMLFEHRSWKKIQELKEARIAGCRNSAEADR

YISSKRKREAEENVRRTRTKESAQVGPSNQGIPNALTSPDSAGKDTSARPAGPATSSSVN

EMDVIGYSGADLLCEAEKRLCCELRLSPAMYLKMQEQLSLQILAGTVSAKSDAHKLFNKL

DAIKIDRVYDMLIKKGIGLP

>GAU38960.1 hypothetical protein TSUD_378360 [Trifolium subterraneum]

MGRSRGNFHHADEDPSQRSRRKKNAASGENSESGAAGQGASEGKRALYHCNYCNKDITGK

IRIKCAMCPDFDLCIECFSVGAEVQPHKSNHPYRVMDNLSFPLICPDWNADDEILLLEGI

EMYGMGNWAEVAEHVGTKNKEACIEHYQNVYLNSPFFPLPDLSHVVGKNKKELLAMAKGQ

GEDKKGPSIGDLSIKEESPFSPARVKTEDSHKTGSNSRLTSHLNSESDSGPSVNNHAAAS

ANQRASNKGRGKGGPGIVKTEDSPMDKDFGGNKPNPSGNNGPSLVELSGYNAKRQEFDPE

YDNDAEQLLAEMEFKDTDTEEEREIKLRVLRIYSKRLDERKRRKEFIIERNLLYPSEFEK

DLTPEERAICRKYDMFMRFHSKEEHEELLRTTIEEHRTLKRLTDLKEARAAGCRNPAEAE

RYLAQRRRKEAEESARRAREGAHVGPNNHGVPNALMSPDSTGTRPAGPANSSSVNQMDLS

GYYGADLLSEAEKRLCCELRMAPAMYLKMQEQLSVQMITGAVTSKSEAHQMFKMEDPIKI

DRVYDMLIKKGIGSP

>PNY06312.1 transcriptional adapter ADA2b-like protein [Trifolium pratense]

MGRSRGNFHHADEDPSQRSRRKKNAASGENSESGAAGQGASDGKRALYHCNYCNKDITGK

IRIKCAMCPDFDLCIECFSVGAEVQPHKSNHPYRVMDNLSFPLICPDWNADDEILLLEGI

EMYGMGNWAEVAEHVGTKNKEACIEHYQNVYLNSPFFPLPDLSHVVGKNKKELLAMAKGQ

GEDKKGPSIGDLSIKEESPFSPARVKTEDSHKTGSNSRLTSNLNSESDSGPSVNNHAAAS

ANQRASNKGRGKGGPGIVKTEDSPMDKDFGGNKPNPSRNGGPSLVELSGYNAKRQEFDPE

YDNDAEQLLAEMEFKDTDTEEEREIKLRVLRIYSKRLDERKRRKEFIIERNLLYPSEFEK

TLTPEERAICRKYDMFMRFHSKEEHEELLRVTIEEHRTLKRLTELKEAKAAGCRNPAEAE

RYLAQKRRKEAEESARRAREGAHVGPNNHGVPNAMMSPDSAGTRPAGPANSSSANQMDLS

GYYGADLLSEAEKRLCCELRMAPAMYLKMQEQLSVQMITGTVTSKSEAHQMFKMEDPIKI

DRVYDMLIKKGIGSP

>XP_028754930.1 transcriptional adapter ADA2b-like isoform X2 [Prosopis alba]

MGRSRGNFHADEDPNQRSRRKKNAASGDITEPGAAGQGTSEGKKALYHCNYCNKDITGKI

RIKCAMCPDFDLCIECFSVGAEVTPHKSNHPYRVMDNLSFPLICPDWNADDEILLLEGIE

MYGLGNWAEVAEHVGTKNKEACIEHYRNVYLNSKTFPLPDMSHVVGKNRKELLAMAKGQS

DDKKGIPMGDLGIKEESPFSPARVKVEDSHKAGKSSRLQSNLNSESGHSSGSHTAATANQ

RASNVGRGKDGPGVIKMEDAQLEKYSGGNKPSSSGNEGPSLVELSGYNPKRQEFDPEYDN

DAEQLLAEMEFKDTDTDDERELKLRILRIYSKRLDERKHRKDFILERNLLYPNPFEKDLT

PEERTICRKYDVFMRFHTKEDHEELLKVVISEHRIRRRIEELQEARAAGCRTSTEADRYL

EQKRRREAEESARRSKERAQTGPNNHGVPSAFMSPDSAGKDSSARPAGPAGSSSVNEMDV

TGYYGADLLSAPEKRLCCEHRLPPAIFLKMQEVLSVQILSGNITAKSDAHHLFNMDSSKI

DCVYDILMKKGIAMP

>XP_003594266.1 transcriptional adapter ADA2b [Medicago truncatula]

MGRSRGNFHHNDDDPSQRSRRKKNAASGDNSESGVAGQGAGEAKRALYHCNYCNKDITGK

IRIKCAKCPDFDLCIECFSVGAEVTPHKSNHNYRVMDNLNFHFICPGWHADDEILLLEGI

EMYGMGNWAEVAEHVGTKNKEACIEHYRNVYLNSPFFPLPDMSHVVGKNREEPAMAKGQG

DDKKGLPMGDLSIKEESPFSPSRVKMEDSNKSGSTGRLTSNMNSGSDSGPSVNTHAAASA

NQKASNKGRGKGGPGIVKMEDSPMDRDFGGNKPNSSRNEGPSLVEVSGYNPKRQEFDPEY

DNDAEQLLAEMEFKDTDTEEEREIKLRVLRIYSKRLDERKRRKEFILERNLLYPNPFEKD

LTPEEKTICRKYDMFMRFHTKEEHDELLRTVISEHRTLKRIQELKEARAAGCRSSVEADR

YLAHKRRKESEESACRARESAHVVPNNHGVPNALMSPDSAGTRPAGSSSVNEMDATGYYG

ADLLSEAEKRLCCELRLPPTVYLKMQEDLSVQMIAGNVSSKSDAHQMFKNMDTIKIDRVY

DMLIKKGIGSP

>XP_015938035.1 transcriptional adapter ADA2b [Arachis duranensis]

MGRCRAASRPADDDPNLRSKKKKAALNVENLDTSSPDVAAQGVTDGKGALYHCNYCNKDI

SGMIRIKCAVCQDFDLCIECFSVGAEVTPHKSNHPYMIMDNLSFPLITPDWNADEEMLLL

EGIEMYGFGNWNEVAEYVGTKSKSQCIDHYNAIYMNSPCFPLPDLSHVMGKNKEELLEMA

KGHEVKKGLFLYNLKSQDHPFNADGVKVEEYQSDRSIGEKKPKLSGKVEPSVTELSGYSF

KREEFEIEYDNDAEQVLADMEFKDIDTESEREMKLHVLHIYSRRLDERKRRKNFILERNL

LYPDPFEKSLSPEEVEICQRYKVFTRFHSKEEHMELLKNIIEEHRLIKRIQDLQEAHTAG

YQTSTEAYKFIEQKKKEAERVVKENNHIGVGAKILPRPNYLKGELDSNSLGLHKDITAPL

SGVKYPAAAIQDISRSLEEWDISGFAGAELLSESEINLCNEIRILPSHYFKMQNVLSSEI

SKGNISKKCDAHRLFKVEPSKVDRVYDMLIKKGIASSN

>KRH17662.1 hypothetical protein GLYMA_13G006200 [Glycine max]

MGRCRAASRPGDDDPKYRSKRKRAALNVENSETLPTGQGVTNSKVSPYHCNYCNKDISGK

IRIKCAVCQDFDLCIECFSVGAEVTPHKSNHPYRIMDNLSFPLICPDWNADEEMLLLEGI

EMYGFGNWNEVAEYIGTKSKSQCIDHYNAVYMNSPCFPLPDLSHVMGKSKEELFAMMKGH

EAKKEFSLTTELTLKEEPPFVDGINYEESKKEEINDQTMSRLTSACGKAYSSTVKKASSV

IQNNDGVKVEESHADRSIGEKKLKLSGEDRPSMTNLSGYSFKREEFDVEYDNDAEQVLAD

MEFKDTDTEAEYEMKLQVLHIYSKRLDERKRRKNFILERDLLYPDPFEKSLLPEELQICQ

RYKVFMRFHSKEEHQDLLKNIIEEHRLVKRIQDLQEARIAGCVTAADAYRFIEQKRTKEA

EPSACKESGQIGTSAKTLQRPNSLKGEVDSSPQGLQKGTAALFAGAKDSPPAIQVFTRSL

EEWDISGFAGAELLSESEKKLCDEIRILPSHYLNMLQTLSLEISKGSVTKKSDAHALFKV

EPSKVDRVYDMLVTKGVVQT

>XP_028198151.1 transcriptional adapter ADA2a-like isoform X5 [Glycine soja]

MGRCRAASRPGDDDPKYRSKRKRAALNVENSETLPTGQGVTNSKVSPYHCNYCNKDISGK

IRIKCAVCQDFDLCIECFSVGAEVTPHKSNHPYRIMDNLSFPLICPDWNADEEMLLLEGI

EMYGFGNWNEVAEYIGTKSKSQCIDHYNAVYMNSPCFPLPDLSHVMGKSKEELFAMMKGH

EAKKEFSLTTELTLKEEPPFVDGINYEESKKEEINDQTMSRLTSACGKAYSSTVKKASSV

IQNNDGVKVEVESHADRSIGEKKLKLSGEDRPSMTNLSGYSFKREEFDVEYDNDAEQVLA

DMEFKDTDTEAEYEMKLQVLHIYSKRLDERKRRKNFILERDLLYPDPFEKSLLPEELQIC

QRYKVFMRFHSKKEHQDLLKNIIEEHRLVKRIQDLQEARIAGCVTAADAYRFIEQKRTKE

AEPSACKESGQIGTSAKTLQRPNSLKGEVDSSPQGLQKGTAALFAGAKDSPPAIQVFTRS

LEEWDISGFAGAELLSESEKKLCDEIRILPSHYLNMLQTLSLEISKGSVTKKSDAHALFK

VEPSKVDRVYDMLVTKGVVQT

>XP_027343936.1 transcriptional adapter ADA2-like isoform X2 [Abrus precatorius]

MGRCRAASRPADDDPNHRSKRKRAALNVESPETLPTGHGLADSKVSLYHCNYCNKDISGK

IRIKCVVCQDFDLCIECFSVGAELTPHKSNHPYRIMDNLSFPLICLDWNADEEMLLLEGI

EMYGFGNWNEVAEYVGTKNKSQCIDHYNAVYMNSPCFPLPDLSHVMGKSREELLAMAKGH

EAKKEFPPTAELTLKEEPPFSDGINYEESKKAEITNQKVSRLTSACGKAYSSTTKKASNV

SQSNDGVKVEESQADRSIGEKKPKISGEDRPSMTELSGYSFKREEFEIEYDNDAEQVLAD

MEFKDTDTETEREMKLHVLHIYSKRLDERKRRKNFILERDLLYPDPFEKSLSSEELQICQ

RYKVFMRFHSKEEHRELLKNIIEEHRLAKRIQDLQDARIAGCVTAAEAYQYIEQKRTKEA

ESGTCKEIGHIGTSAKTVQRPSYFKGELDSSPQGLQKGTTALLNGAKDSSTVIQAITRSL

EEWDISGFAGAELLSESEKNLCNEIRILPSHYLNMLQTISLEISKGSVTKKSDAHTLFKV

DPSKVDRVYDMLVKKRVVKE

>TKY47885.1 Transcriptional adapter ADA2 [Spatholobus suberectus]

MGRCRTVSRPVADDDPDHNRSKRKRAALNVENSETLPTGQGITKSKVSLYHCNYCNKDIS

GKIRIKCVVCQDFDLCIECFSVGAEVTPHKSNHPYRIMDNLSFPLICPDWNADEEMLLLE

GIEMYGFGNWNEVAEYVGMKSKTQCIDHYNAVYMNSPCFPLPDLSHVMGKSREELMAKGH

EVRKEFPLTAELTLKEEPPFSNGINYEESKKAEINNQTISSLTSGSACGKAYSSTIKRAS

NVSQNNDGVKVEESQSDRSIGEKKLKLSGEDRPSMTELSGYSFKREEFDVEYDNDAEQVL

ADMEFKDTDTEAEHEMKLHVLHIYSKRLDERKRRKNFILERDLLYPDPFEKSLLPEELQI

CQRYKVFMRYHSKEEHQDLLKNIIEEHRLVKRIQDLQEARIAGCVTAADAYRFIEQKRTK

EAEPSACKESGQIGTSAKTLQRPNYLKGELDSSPRGLQKGTTALFAGAKDSPTAIQAITR

SIEEWDISGFAGAELLSESEKKLCNEIRLLPSHYLNMLQTMSLEISKGTVTKKSDAHTLF

KVEPSKVDRVYDMLAEKGVVQT

>XP_020237926.1 transcriptional adapter ADA2a [Cajanus cajan]

MGRCRAASRPADDDPNHRSKRKKAALTVENSETLPTGQGITNSKVSLYHCNYCNKDISGK

IRIKCVLCQDFDLCIECFSVGAEVTPHKSNHPYRTMDNLSFPLMCPDWNTDEEMLLLEGI

EMYGFGNWNEVTEYVGTKSKFQCIDHYNAVYMNSPCFPLPDLSHVMGKSREELFAMVKGR

EVMKEIPLTAELTLKEEIPFSDGISYEESKKAEIYNQTMSRLTSECGKAYSSTIKKASNV

SQNNDGVKMEESQADRSIGEKKPKLSGEDRPSMTELSGYSFKREEFDIEYDNDAEQVLAD

MEFKDTDTEAEHKMKLHVLHIYSKRLDERKRRKIFILERDLLYPDPFEKSLLPEELQICQ

RYKVFTRFHSKQEHLDLLKTIIEEHRLVKRIQDLQEARIAGCVTAADAYRFIEQKRTKEA

ESSACKESGQIGTSAKTLLRPNYLKGELDTSPRGLQKGTSALFAGAKDSPAAIQTITRSL

EEWDISGFAGAELLSESEKKLCNEIRVLPSHYLNMLQTMSSEISKGNVTNKSDARTLFKV

EPSKVDRVYDMLVNKGVVQT

>XP_025657096.1 transcriptional adapter ADA2a isoform X3 [Arachis hypogaea]

MGRCRAASRLADDDPNLRSKKKKAALNVENLDTSSPDVAAQGVTDGKGALYHCNYCNKDI

SRMIRIKCAVCQDFDLCIECFSVGAEVTPHKSNHPYMIMDNLSFPLITPDWNADEEMLLL

EGIEMYGFGNWNEVAEYVGTKSKSQCIDHYNAIYMNSPCFPLPDLSHVMGKNKEELLEMA

KGHEVKKECPATAKLTLKEEPPLSEGINSEESNRAEVTNPTSMLTSEYQSDRSIGEKKPK

LSGKVEPSVTELSGYSFKREEFEVEYDNDAEQVLADMEFKDIDTESEREMKLHVLRIYSR

RLDERKRRKNFILERNLLYPDPFEKSLSPEEVEICQRYKVFTRFHSKEEHMELLKNIIEE

HRLIKRIQDLQEAHTAGYQTSTEAYKFIEQKKKEAERVVKENNHIGVGAKILPRPNYLKG

ELDSNSLGLHKDITAPLSGVKYPAVAIQDISRSLEEWDISGFAGAELLSESEINLCNEIR

ILPSHYFKMQHVLSSEISKGNISKKCDAHRLFKVEPSKVDRVYDMLIKKGIAPSN

>XP_020959032.1 transcriptional adapter ADA2a [Arachis ipaensis]

MGRCRAASRLADDDPNLRSKKKKAALNVENLDTSSPDVAAQGVTDGKGALYHCNYCNKDI

SRMIRIKCAVCQDFDLCIECFSVGAEVTPHKSNHPYMIMDNLSFPLITPDWNADEEMLLL

EGIEMYGFGNWNEVAEYVGTKSKSQCIDHYNAIYMNSPCFPLPDLSHVMGKNKEELLEMA

KGHEVKKECPATAKLTLKEEPPLSEGINSEESNRAEVTNPTSMLTSVCGKTYSSTIKKAS

NVSQNADGVKMEEYQSDRSIGEKKPKLSGKVEPSVTELSGYSFKREEFEVEYDNDAEQVL

ADMEFKDIDTESEREMKLHVLRIYSRRLDERKRRKNFILERNLLYPDPFEKSLSPEEVEI

CQRYKVFTRFHSKEEHMELLKNIIEEHRLIKRIQDLQEAHTAGYQTSTEAYKFIEQKKKE

AERVVKENNHIGVGAKILPRPNYLKGELDSNSLGLHKDITAPLSGVKYPAVAIQDISRSL

EEWDISGFAGAELLSESEINLCNEIRILPSHYFKMQHVLSSEISKGNISKKCDAHRLFKV

EPSKVDRVYDMLIKKGIAPSN

>KEH35178.1 transcriptional adapter ADA2a [Medicago truncatula]

MVFLSKRKKVALNADDLEASYAGMGITDGKVSLYHCNYCKKNISGKIHIKCAVCQDFDLC

IECFFVGAELTPHKSNHPYRVMDSLSFPLTSPDWSAGEEKLLIEAIDMYGFGNWNGVAEN

VGTKSKSQCIDHYNSVYLNSPCFPLPDLSYSMGKNKEELLAMAKGHQLKKGLLLDDRNHN

FSEESKKAETTNQNMSRPTSARDEALPSITKKASNVNQNNDGVKVEESQAGWSAGEKKPK

LSGEYKPSMEVLRGYNSNRGEFEIEYDNDAEQVLAEMEFKDTDTEAEREMKLQVLRGYGK

KLNERKRRKEFILERNLLCPDPFEKFLSPEELQICEQYKVFMRFHSKEEHEELLQTAIRE

HRLAKRIKDLKEARIAGCVTSDEAYQFIEQKRTKEAEQGNCKESGQIGTSGKTESFPSTK

DAPPAIQAITKTLEEWDISDFEGAELLSESEIKLCNEIRMLPPIFLNITRIMQLEISKGR

VTKKSDAYPLFKFSPSKIDRIYDMLVEKGVVQA

>XP_018823893.1 PREDICTED: transcriptional adapter ADA2-like [Juglans regia]

MGRSRGNFHSADEDPTQRSRRKKNASSGENSESASAGQGTSEGKRALYHCNYCNKDITGK

IRIKCAVCPDFDLCIECFSVGAEVTPHKSNHPYRVMDNLSFPLICADWNADDEILLLEGI

EMYGLGNWAEIAEHVGTKSKEQCIEHYTNVYMSSPYFPLPDMSHVVGKNRKELLAMAKGH

SEDKKGFPMIGELNLKEESPFSPSRVKVEDSHKGGSAGRLLSSLTAEVESGVRSSGSSAA

STAANKKASNIAQVKDGNGVVKLEDPQADRSFGGKKPNSSGNKGPSLVELSGYNPKRHEF

DPEYDNDAEQLLAEMEFKDTDTEDERELKLRVLRIYSKRLDERKRRKDFILERSLLYPNS

FEKDLSPEERAICRRYDVFMRFHTKEEHEDLLQTVIAEHRTLKRIQELKEARAAGCRTSA

EANRYLELKRKRESDETARRTKESAQIGLSSQAGPNTFMASESVGKELNSRPVGQAASSS

VNDLDIGGSHGADLLSESEKRLCSEIRLPPPLYLKVQEVMSIEIINGNVTKKSDAHHLFK

LEPSKIDRVYDILVKKGIAQP

>KAF3975783.1 hypothetical protein CMV_000977 [Castanea mollissima]

MGRSRGNFHSPDEDPTQRSRRKKNANSGENSESAAAGQGTSEGKRALYHCNYCNKDITGK

IRIKCFNCPDFDLCIECFSVGAEVQPHKSNHPYRVMDNLSFPLICPDWNADDEILLLEGI

EMYGMGNWTEVAEHVGTKSKEQCIDHYTNKYKNSPYFPLPDMSHVVGKNRKELLAMAKGH

SEDKKGFPMMGELNLKEESPFSPPRVKVEESHKGGSSSRLLSSLNAEVESGVRSSSSSSA

ATAANKKASYMTQVKDGPGVIKMEDHQADRSFGGKRPHSSGSEGPSLVELSGYNHKRQEF

DPEYDNDAEQLLAEMEFKDTDSEDERELKLRVLRIYAKRLDERKRRKDFILERNLLYPNH

FEKDLSPEERAICRRYDVFMRFHSKEEHEDLLQTVISEHRTLKRIQELKEARAAGCRTAA

EADRYLEQKRKREAEESALRAKESAQVGPSSQAGPNPFMASESGGKESNSRPAVQATSSS

MNDLEITGLHGADLLSESEKRLCSEIRLPPALYLNMQEVMSIEIINGNVSKKADAHRLFK

LETSKIDRVYDMLVKKGIAQP

>XP_030972601.1 transcriptional adapter ADA2 [Quercus lobata]

MGRSRGNFHSPDEDPTQRSRRKKNANSGENSESAAAGQGTSEGKRALYHCNYCNKDITGK

IRIKCFNCPDFDLCIECFSVGAEVQPHKSNHPYRVMDNLSFPLICPDWNADDEILLLEGI

EMYGMGNWAEVAEHVGTKSKEQCIDHYTNKYKNSPYFPLPDMSHVVGKNRKELLAMAKGH

SEDKKGFPMMGELNLKEESPFSPSRVKVEESHKGGSSSRLLSSLNAEVESGVRSSSSSSA

ATAANKKASNMAQVKDGPGVIKVEDRQADRSFGGKRPHSSGSEGPSLVELSGYNHKRQEF

DPEYDNDAEQLLAEMEFKDTDTEDERELKLRVLRIYAKRLDERKRRKDFILERNLLYPNH

FEKDLSPEERAICRRYDVFMRFHAKEEHEDLLQTVISEHRTLKRIQELKEARAAGCRTAA

EADRYLEQKRKREAEESARRAKESAQVGPSNQAGPNPFMASESGGKESNSRPAVQATSSS

INDLEITGLHGADLLSESEKRLCSEIRLPPALYLNMQELMSIEIINGNVSKKADAHRLFK

LETSKIDRVYDMLVKKGIAQP

>XP_018836315.1 PREDICTED: transcriptional adapter ADA2-like isoform X2 [Juglans regia]

MGRSRAVLHPAEDDPNQRSKRKRTVSNVETMGITPSVEGISEGKVALYHCNYCNKDISGR

IRMKCVMCPDFDLCVECFSVGAEVTPHKSNHPYRVMDNLSFPLICPDWNTDEEILLLEGI

EMYGFGNWTEVAEHVGTKTKSQCIDHYNGIYMNSPCFPLPDMSHVMGKSRAELLALAKGP

GEVRKEIPKIGELTLKEESPFSGRLSCDESKKGLAWHSSSSLTSGTGLDSSSGNTFSGVI

KKASNKVQTKDGTSTEEFPADRSIGEKKPRVPGDEEPSMTELSGYNFKRQEFEIEYDNDA

EQVLADMEFRDTDGDAEHELKLRVLRIYSKRLDERKRRKDFILERNLLYPDPFEKSLSPE

EREICRRYRVFMRFHSKEEHDELLKNIIEQQRIVKRIQDLQKARAAGCRTAAEANKFLEQ

KRNMGVEESEQGVNESAQGAGGKVLQRQNHLKGEFDASTQGVPKESPVLHPSGKDTSSAM

QATSNSLQEWDITGFVGADLLSETEKRLCGEIRILPSHYLNMLQIISLEIMKGNVTKKSD

AHSLFKVEPSKVDVVYDMLVQKGIAQA

>KAE8008270.1 hypothetical protein FH972_004798 [Carpinus fangiana]

MGRSRALSHTVEEDPNQSRSKRKRAASNAEITVQGIFEGKVALYHCNYCHKDVSGRVRIK

CVMCPDFDLCIECFSIGAEVTPHKSNHPYRVMDNLCFPLMCPDWNVDEEILLLEGIEMYG

FGNWTEVAEHVGTKTKLQCIDHYNAVYISSPCFPLPDMSHVMGKSREELLAMAKGPGEVK

EEIPNIRELTLKEESPFPARDGYDESKKGQPCHSSSSFTSDVGTGLGSSSGDTFSNAVKK

ASNKAQIKDGTTVQELQSDRSIREKKPRVLGDEGPSMTELSGYNFKRQEFEIEYDNDAEL

VLADMEFKDTDSDTDHELKLRVLRIYSKRLDERKRRKDFILERNLLYPDPFEKSFTPEER

EICQRYRVFMRYHLKEEHDELLKNIIEEQRIVKRIQDLQEARAAGCCTAAEANRFIEQKR

KKEAEESEHGVKESAQGPNGKVPQKQNHLKGEFEASPKGFIKGSTVLQPSSSDSSSALQA

ISSCVDEWDINGFVGADLLSETEKGLCGEIRILPSHYLNMLEVMSVEILKGNVTKKSDAH

SLFKVEASKVDKVYDMLVEKGIAQAL

>XP_023873221.1 transcriptional adapter ADA2-like [Quercus suber]

MGRSRAVSHLSEEDPNQRSKRKRPTTITTTTTTSNTSNAASASNAETTETATPTGKLALY

HCNYCNKDISGKIRIKCVMCPDFDLCIECFSVGAEVTPHKSNHPYRVMDNLAFPLLCSDW

NVDEEMLLLEGIEMYGFGNWNEVSEHVGTKRRSQCIDHYNAVYMNSPCFPLPDMSHVMGK

SREELLAMSRGHGEVKKEIPNVGELTLKEESSFSARVRSDELKEGPAWQSSSSLTSETGK

GLGSSSGNTFSSVIKKASNNSQIKDGIKMEEFQSDRSIGEKKPRVPGDEGPSMTELSGYN

FKRQEFEIEYDNDAEQILADMEFKNNDSAADHELKLRVLRIYSKRLDERKRRKDFILERN

LLYPDPLEKSLLPKEREICQRYKVFMRFHSKEEHDELLKNIIEEQQIVKRIQDLQEARAA

GCRTAAEANRFLEQKRKMEAEESEHGIKESAQGPSGKVLQRPTQLKGEFDASPRGVVKGS

TGLELSDKDSSSAMQAISSDLDKWDITGFVGADLLSETEKQLCGEIRILPAHYLNMLQII

SVEILKGNVTNRSDAHGLFKVEPSKVDRVYDMVVKKGIVQA

>KAF3964090.1 hypothetical protein CMV_011594 [Castanea mollissima]

MGRSRAVSHLSEEDPNQRSKRKRPTTITTTTTTSNTSNAASASNAETTETATPTGKLALY

HCNYCNKDISGRIRIKCVICPDFDLCIECFSVGAEVTPHKSNHPYRVMDNLAFPLLCSDW

NVDEEMLLLEGIEMYGFGNWTEVSEHVGTKRRSQCIDHYNAVYMNSPCFPLPDMSHVMGK

SREELLAMSRAHGEVKKEIPNVGELTLKEDSSFSARVRSDESKEGPAWQSSSSLTSETGK

GLGSSSCNTFSSVIKKASNNSQIKDGIKTEEFQSDRSIGEKKPRVPGDEGPSMTELSGYN

FKRQEFEIEYDNDAEQILADMEFKDTDSAADRELKLRVLRIYSKRLDERKRRKDFILERN

LLYPDPLEKSLLPREREICQRYKVFMRFHSKEDHDELLKNIIEEQQIVKRIHDLQEARAA

GCRTAAEANRFLEEKRKMEAEESEHGIKEGAQGPSGKVLQRPTQLKGESDASPRGVVKGS

TGLELSGKDSSSAMQAISSDLDKWDITGFVGADLLSETEKQLCGEIRILPAHYLNMLQII

SVEILKGNVTNRSDAHGLFKVEPSKVDRVYDMVVKKGIVQA

>XP_030971490.1 transcriptional adapter ADA2-like [Quercus lobata]

MGRSRAVSHLSEEDPNQRSKRKRPTTITATTTTSNTSNAASASNAETTETATPTGKLALY

HCNYCNKDISGKIRIKCVMCPDFDLCIECFSVGAEVTPHKSNHLYRVMDNLAFPLLCSDW

NVDEEMLLLEGIEMYGFGNWTEVSEHVGTKRRSQCIDHYNAVYMNSPCFPLPDMSHVMGK

SREELLAMSRGHGEVKKEIPNVGELTLKEESSFSARVRSDESKEGPAWQSSSSLTSETGK

GLGSSSGNTFSSVIKKASNNSQIKDGIKMEEFQSDRSIGEKKPRVPGDEGPSMTELSGYN

FKRQEFEIEYDNDAEQILADMEFKNTDSAADRELKLRVLRIYSKRLDERKRRKDFILERN

LLYPDPLGKGLLPKEREICQRYKVFMRFHSKEEHDELLKNIIEEQQIVKRIQDLQEARAA

GCRTAAEANSFLEQKRKVEAEESEHGIKESAQGPSGKVLQRPTQLKGEFDASPRGVVKAS

TGLELSEKDSSSAMQAISSDLDKWDITGFVGADLLSETEKQLCGEIRILPAHYLNMLQII

SVEILKGNVTNRSDAHGLFKVEPSKVDRVYDMVVKKGIVQA

>KAB1221705.1 Transcriptional adapter ADA2b [Morella rubra]

MGRSRGNFHSADEDPTQRSRRKKNASSGENSESTSAGQGTSEGKRALYHCNYCNKDITGK

IRIKCYVCPDFDLCVECFSVGAEVQPHKSNHPYRVMDNLSFPLICPDWNADDEILLLEGI

EMYGFGNWAEVAEHVGTKSKEQCIEHYTNVYINSPHFPLPDMSHVVGKNRKELLAMAKGH

SEDKKGFPMIGELNLKEESAFSPSRVKVEDSHKGGSSGRLLSSLSTGEAEHILGSSYEVE

SGVGRSSGSSAAATAANKKASNMAQVKDGPGVIKVEGPQADRSFGGKKPSSSANEGPSLV

ELSGYNPKRQEFDPEYDNDAEQLLAEMEFKDTDTEEEREIKLRVLRIYSKRLDERKRRKD

FILERNLLHPNPFEKDLSPEERAICRRYDVFMRFHTKEEHDDLLQTVITEHRMLKRIQEL

KDARAAGCRTSTEADRYMELKRKREAEESARRARESTQAGPSSQAGPNAFMASESVGKEL

NSRPTGQATSSSVNDFDVMGSHGADLLSESEKRLCSEVRLPPPLYLKMQEVMSIEIINGN

LTKKSDAHHLFHLDPSKIDRVYDMLVKKGIAQP

>OAY41154.1 hypothetical protein MANES_09G078400 [Manihot esculenta]

MGRSRGNFHSADEDPTQRSRRKKNAASGENLESSSAGDGKRALYHCNYCNKDITGKIRIK

CVMCPDFDLCIECFSVGAEVTPHKSSHTYRVMDNLSFPLICPDWNADDEILLLEGIEMYG

LGNWAEVAEHVGTKSKEMCIEHYTNIYMNSPFFPLPDMSHVVGKNRKELLAMAKGHGEDK

KGTSMLGEHTLKEESPFSPSRVKVEEMHKGGPSGRLISNINADPQTDRSFKGKKLNSSGN

EGSLVEASGYNPKRQEFDPEYDNDAEQLLAEMEFKDTDTEDERELKLRVLRIYSKRLDER

KRRKEFILERNLLYPNHFEKDLSPEERALCRRYDVFMRFHSKEEHEDLLQTVISEHRTLK

RIQELKEARAAGCRTSAEADKYLEDKRKREAEENSQRAKESVQVGPSNQGGPNVFMASES

VCKDSNPRPAGQYINDLDALSFYETQLLSEAEKRLCHEIKLPPPLYLKMQEVMTKEIFSG

NVTKKSDAHPLFKLEASKIDRVYDVLVKKGIAQP

>EEF39499.1 transcriptional adaptor, putative [Ricinus communis]

MGRSRGKFHSADEDPIQRSRRKKNAASGENLESSSVGQGTSEGKRALYHCNYCNKDITGK

IRIKCAMCPDFDLCIECFSVGAEVTPHKSNHPYKVMDNLSFPLICPNWNADDETLLLEGI

EMYGLGNWTEVAEHVGTKSKEMCIEHYTNIYMNSPFFPLPDMSHVVGKNRKELLAMAQGH

GEDKKGSSMLGEHTLKEESPFSPSRVKYAFYVESGIRPNSLNAAATSAIKKASKIARVKD

GSNIVKVEEPQTDRSFKGKKPNSSGKNGSLIESSGYNAKRQEFDPEYDNDAEQLLAEMDF

KDTDTEDERELKLRVLRIYSKRLDERKRRKDFILERNLLYPNLFEKDLSPEEKALCRRYD

VFMRFHSKEEHEELLQTVISEHRTLKRIQELKEARAAGCHSSADADRYLEQKRKREAEES

SQRAKESGQVGPSNQGGPNVFIGSDSISKDSNSRPAGQSYVNDLERLGFSEAQLLTESEK

RLCQEIKLPPAVYLKMQEVMTKEIFIGNVTKKADAHPLFKLEASKVDRVYDVLVKKGIAQ

P

>XP_021676495.1 transcriptional adapter ADA2b isoform X2 [Hevea brasiliensis]

MGRSRGNFHSVDEDPTQRSRRKKNSASGENLESSSAGQGTGEGKRALYHCNYCNKDITGK

IRIKCAMCPDFDLCIECFSVGAEVTPHKSNHPYRVMDNLSFPLICPDWNADDEILLLEGI

EMYGLGNWAEVAEHVGTKSKELCIEHYTNIYMNSPFFPLPDMSHVVGKNRKELLAMAKGH

GEDKKGSSMLGEHTVKEESPFSPSRVKVEEMHKGGPSGRLLSNLNADVESGVHPNSLDAA

ATAAIKKASKMAHVKDGLSVVKVEDPQTDRSFKGKKLNSSGNDGSLVELSGYNPKRQEFD

TEYDNDAEQLLAEMEFKDTDTDDEHELKLRVLRIYSKRLDERKRRKDFILERNLLYPNRF

EKDLSPEERALCRRYDVFMRFHSKEEHEDLLQTVIAEHRTLKRIQELKEARAAGCRTSAE

ADKYLEEKRKREAEESSHRAKESVQVGPSNQGGPSVFMASESVGKDSTTRPAGHYVNDMD

ALGFYETQLLSEAEKRLCHEIKLPPPLYLKMQEVMTKEIFSGNVTKKSDAHPLFKLEASK

IDRVYDVLVKKGIAQP

>XP_012082182.1 transcriptional adapter ADA2b isoform X2 [Jatropha curcas]

MGRSRGNFHSADEDPTQRSRRKKNAASGENLESSSAGQGTGEGKRALYHCNYCNKDITGK

IRIKCAMCPDFDLCIECFSVGAEVTPHKSNHPYRVMDNLSFPLICPDWNADDEILLLEGI

EMYGLGNWAEVAEHVGTKSKEMCIEHYTNIYMNSPFFPLPDMSHVVGKNRKELLAMAKGH

GEDKKGSSMLGEHTLKEESPFSPSSRVKVEDMHKGGASGRLLSTLNGDPQTEIGFKGKKS

NSSANEGSLIESSGYNPKRQEFDPEYDNDAEQLLAEMEFKDTDTEDERELKLRVLRIYAK

RLDERKRRKDFILERNLLYPNVFEKDLSPEERALCRRYDVFMRFHSKEEHEDLLQTVIAE

HRTLKRIQELKEARAAGCRTSAEADRYREEKRKREAEESSQRAKESSQVGPSNQGGPNVF

MASESLGKDSNARPGGQGTGYETLDALGFYETQLLSEAEKRLCHEIKLPPPLYLKMQEVM

TKEIFSGNVTKKSDAHPLFKLESSKVDRVYDVLVKKGIAQP

>XP_034898193.1 transcriptional adapter ADA2b-like [Populus alba]

MGRSRGNFHSTDEDPTQRSRRKKNAASGENSESSSAGQGGSDGKRALYHCNYCNKDITGK

TRIKCAVCPDFDLCLECFSVGAEVTPHKSNHPYRVMDNLSFPLICPDWNADEEILLLEGI

EMYGLGNWAEVAEHVGTKNKETCIKHYNSVYMQSQFFPLPDMSHVVGKNRKELLAMAKGH

SEDKKGTSMLGERTLKEESPFSPSRVKVEEMHKVGSFGRLSTLNSEVETASRPNSANSAA

TAANKKASSMAQINDGPGVKVEDPQVDRNFKGKKPSSSGSEGPSLMELSGYNPKRQDFDP

EYDNDAEQLLAEMEFKDNDTEEEHELKLRVLRIYSRRLDERKRRKDFILERNLLHPSPFE

KDLTPEERALCWRFDPFMRFHSKEEHEELLQAVVKEHRMMKRIEELKDAQVAGCRTAAEA

DRYLEHKRKIEAEETSRRLKDNAQIGPSSQGAPSAFMSPDSVGKDSSTRPAGQGSSSYAN

NLDIMGFYETQLLSETEKRLCCEIHLPPPVYLKMQEVMTKEIFSGNITKKSDAHPLFKIE

ASKVDGVYDMLVKKGIAQP

>XP_024443401.1 transcriptional adapter ADA2b isoform X1 [Populus trichocarpa]

MGRSRGNFHSTDEDPTQRSRRKKNAASGENSESSSAGQGSSDGKRALYHCNYCNKDITGK

TRIKCAVCPDFDLCLECFSVGAEVTPHKSNHPYRVMDNLSFPLICPDWNADEEILLLEGI

EMYGLGNWAEVAEHVGTKNKETCIKHYNSVYLQSQFFPLPDMSHVVGKNRKELLAMAKGH

SEDKKGTSMLGEHTLKEESPFSPSRVKVEEMHKVGSSGRLSTLNSELETASRPNSANSAA

TAANKKASSMARINDGPGVKVEDPQVDRNFKGKKPSSSGSEGPSLMELSGYNPKRQEFDP

EYDNDAEQLLAEMEFKDNDTEEERELKLRVLRIYSRRLDERKRRKDFILERNLLHPSPFE

KDLTPEERALCRRFDPFMRFHSKEEHEELLRAVVKEHWMLKRVEELKDAQVAGCRTAVEA

DRYLEHKRKIEAEETSRRLKDNAQIGPSSQGAPNAFMSPDSVGKDSSTRPAGQGSSSYAN

DLDIMGFYETQLLSETEKRLCCEIHLPPPVYLKMQEVMTKEIFSGNITKKSDAHPLFKIE

ASKVDGVYDMLVKKGIAQP

>XP_011040180.1 PREDICTED: transcriptional adapter ADA2b-like [Populus euphratica]

MGRSRGNFHSTDEDPTQRSRRKKNAASGENSESSSAGQGSSDGKRALYHCNYCNKDITGK

TRIKCAVCPDFDLCVECFSVGAEVTPHKSNHPYRVMDNLSFPLICPDWNADEEILLLEGI

EMYGLGNWAEVAEHVGTKNKETCIEHYDSVYTQSQFFPLPDMSHVVGKNRKELLAMAKGH

SEDKKGTSMLGEHTLKEESPFSPSRVKVEEMHKVGSSGRLSTLNSEVETVSRPNSANSAA

TAANKKASSTARIKDGPGVKVEDPQEDRNFKGKIPSSSGSEGPSLMELSGYNPKRQEFDP

EYDNDAEQLLAEMEFKDNDTEEERELKLRVLRIYSRRLDERKRRKDFILERNLLHPSPFE

KDLTPEERALCRRFDPFMRFHSKEEHEELLRAVVKEHRMLKRIKELKEAQAAGCRTAAEA

YRYLEHKRKIEAEETSRRLKENSQIGPSSHGAPNAFMTPDSVGKDSSTRPAGQGSSGYVN

DLDIMGFYETQLLSETEKQLCCEIHLPPPVYLKMQEVMTKEIFSGNITKKSDAHPLFKIE

ASKVDGVYDMLVKKGIAQP

>KAB5551556.1 hypothetical protein DKX38_008867 [Salix brachista]

MGRSRGNFHSNDEDPTQRSRRKKNAASGDNSESSLAGQGSGDGKKALYHCNYCNKDITGK

TRIKCAMCPDFDLCLECFSVGAEVTPHKSNHPYRVMDNLSFPLICPDWNADEEILLLEGI

EMYGLGNWAEIAEHVGTKSKETCIEHYSSVYMQSQYFPLPDMSLVVGKNRKELLAMAKGY

SEDKKGAAMLGELTLKEESPFSPSRVKVEEMHKGGSSGRLSTLNSEVESAGRPTTTNSAA

TAANKKASSTARVKDGASVVKVEDPQVDRNAKGKKPNSSGSEGPSLMELSGYNSKRQEFD

PEYDNDAEQLLAEMEFKDTETEEERELKLRVLRIYSKRLDERKRRKDFILERNLLQPSPF

EKDLTPEERALCQRYDPFMRFHSKEEHEELLQVVVEEHRMLKRMEELKEAQAAGCRTAAE

ADRYLEQKRKKEAEENSRRLKDNAQVGPSNHGAPNAFMPSESVGKDSSTRPAGQGSASYA

NGLDITGFYETQLLSETEKRLCREIHLPPPVYLKMQEVMTKEIFSGNIANKLDAHPLFKI

EESKVDRVYDMLVKKGIAQP

>XP_015576574.1 transcriptional adapter ADA2 [Ricinus communis]

MGRSRAISRPADEDASQRSKRKKTVSNAGSAQTTSSPACQETSEGKAALYHCNYCKKDIS

GFIRIKCAVCPDFDLCVECFSVGAEVTPHKSSHPYRVMDNLSFPLICPDWNADEEILLLE

GIEMYGFGNWAEVAEHVGTKSKSKCIDHYNAIYMNSPCFPLPDMSHVMGKSREELVAMAK

GQCEIKKEFPAVGDLVLNEESPLSSRIKSESWKKEDVACKSSSSIKAEFGSCMSSSSCTD

AVKKASNTNQIKDGIKVEESLADWSIGEKKLRISGEEQPSMTELSGYNSKRHEFEIEYDN

DAEQILADMEFKASDTDAERELKLRVLRIYSKRLDERKRRKDFILERNLLYPDPFEVNLS

QEERAIYDRYKVFMRFHSKEEHEELMKSVIEEYRIVKRIQDLQDARAAGCQTAAELNRFL

EEKRKKESDESAQRVKESPSGKVLQRTSSLKVEADGSPRGVVTGSTGLHNSGKDSSLTIT

KQISSSLDHWDISGFLGAGLLSECEKHLCGEIRILPSHYLNMLQTMAVEIMKGTITKKSD

AHRLFKVEPSKVDKVYDMLVKKGMAQA

>XP_021658095.1 transcriptional adapter ADA2 isoform X3 [Hevea brasiliensis]

MGRSRAVSHSVLEDVNQRSKRKKTASSLESAQTASTVCQEMSEGKAALYHCNYCNKDISG

MVRIKCAVCPDFDLCIECFSVGAEVTPHKSSHPYRVMDNLSFPLICPDWNADEEILLLEG

IEMYGFGNWTEVAEHVGTKSKLQCIDHYNAVYMNSPCFPLPDMSHVMGKSREELLAMAKE

NCIIKKEFPALGELTPKDESLLSARIKSEEQNKDVACQLSSTLKAEFGSFMSSSSGNTST

GAVKKASNMAQIKDGIKVEDSLADRSIGEKKLRISGEEGPSMTELSGYNFKRQEFEIEYD

NDAEQLLADMEFKDTDTDAERELKLRVLHIYSKRLDERKRRKDFILERNLLYPDAFENTL

SREEREIYQRYKVFMRFHSKEEHEELMKCVIEEHRIVKRIQDLQEARAAGCQTAAEVNRF

IEQKQQKESDEGAQRVKESTIAGPGAKVLQRPSNVKGDVDGSPCGVVRGSAYISSPLDDW

DISGFLGADLLSESEKRLCGELRILPSHYLSMLQKMSLEIMKGTVSKKSDAHILFKVEPS

KVDKVYDMLVKKGIAQA

>XP_012071741.1 transcriptional adapter ADA2a isoform X2 [Jatropha curcas]

MGRSRAVSRSIDDDANHRSKRKKTASNAESGQTASTVSQETSEGKAALYHCNYCKKDISG

MIHIKCAVCPDFDLCLECFSVGAEVTPHKSSHPYRVMDNLSFPLICPDWNADEEILLLEG

IEMYGFGNWTEVAEHVGTKNRSQCIDHYNAVYMNSPCFPLPDMSQVMGKSREELLAMAKG

YGVIKKEFPALGELTLKEESPLSARVKSEAQKKKDVACQSSPSLKSEFGASMSSNSANTF

TGAIKKASNITQMKDGIKVEDLQADRSIGEKKLRAPGEEGPSMTELSGYNFKRQEFEIEY

DNDAEQLLADMEFKDTDTDAERELKLRVLHIYSKRLDERKRRKDFILERNLLYPDPFENN

LAPEEREIYQRYKVFMRFHSKEEHEELMKSVIEEHRIVKRIQDLQEARAAGCRTAAEVNR

FIEQKQKKEADHSIHRVKESTVAGPSGKVLQRPTNLKGEVDGSPRGVVKGSPGLHAGDKD

SFSTIAKQISSSLDDWDISGFLGADLLSESEKRLCGEMRILPSHYLNMLQTMSVEIMKGT

ITKKSDAYGLFKMAPNKVDQVYDMLVKKGIA

>XP_021612255.1 transcriptional adapter ADA2a isoform X1 [Manihot esculenta]

MGRSRAVARSIDEDAHHRSKRKKTGSGLESAETASTVCQEMVEGKAALYHCNYCNKDITG

MIRIKCAVCPDFDLCIECFSVGAEVTPHKSSHPYRVMDNLSFPLICPDWNADEEILLLEG

IEMYGFGNWTEVAEHVGTKSKLQCIDHYNAVYMNSPCFPLPDMSHVMGKSREELLAMAKG

HVVIKKEFPALGGLTLEEESPSSTIIKSEAQQKDVVCQLSSSLKAEFGPSMSSSGDNTFS

GAVKKASNMAQIKDSIKVEDSQSDRSVGEKKLRISGEEGPSMTELSGYNFKRQEFEIEYD

NDAEQLLADMEFKDTDTNAERELKLQVLRIYSKRLDERKRRKDFILERNLLHPDAFENTL

SPEEREIYQRYKVFMRFHSKEEHEELMERVIEEHRIVKRIQVLQEARAAGCRTAAEVNRF

IEQKKKKEADESAKRVKESAVAALSGKVLQRPSNLKGEVDGSPRGVVRGSTGLHIGGIDS

PSTIANKISSSLDDWDISGFLGADLLSESEKHLCGELRILPSQYLSMLQKMSEEIMKGTV

SKKSDAHSLFKVDPSKVDKVYDMLLKRGLVKHD

>XP_034912098.1 transcriptional adapter ADA2a isoform X2 [Populus alba]

MGRSRGRPPSSGTSTAAAASDDPNNRSSKRKKTTSNVGSIETAFPAVYQETSQGKLALYH

CNYCHKDISGMVRIKCAMCPDFDLCIECFSVGAEVTPHKGNHPYRVMDNLSFPLFHPDWN

TDEEILLLEGIEMYGFGNWTEVSEHVGTKSKSQCIDHYNAVYMDSPCFPLPDMSHVMGKT

REELLAMARGNVEMKKELSAFEELTLNQESPFSAKIKSEASKKEDLASHSSSIVNAEVSS

HKGSSSGNTFSDAVKKATNEAQVKDKIKVEEPLSDRSIREKKPRICGEEGPSMTELSGYN

FKRQEFEIEYDNDAEQLLADMEFKDTDTDAELDMKLQVLRIYSKRLDERKRRKDFILERN

LFYPDAFEKNLSPEEKEIYQRYKVFMRFHTKEEHEELMKTVIEDHQIMKRIQDLQEARAA

GCQTAGEAQGFIEQKRKKEAEESAQRAKESMQAGPAGKLLPKPNHLDSSPRGAVKCSTVF

HPGGNDSSSMIAKQAISSTLDEWDIAGFLGADLLSESDKRLCCELRILPAHYLNMLHIMS

TEITKGTVTNKTDAHSLFKVESSKVDRVYDMLVKKGIAQA

>RQP00258.1 hypothetical protein POPTR_014G166300 [Populus trichocarpa]

MGRSRGRPPSSGTSTAAAASDDPNNRSSKRKKTTSNVGSIETAFPAVYQEKGQGKLALYH

CNYCHKDISGMVRIKCAVCPDFDLCVECFSVGAEVTPHKSNHPYRVMDNLSFPLFHPDWN

TDEEILLLEGIEMYGFGNWTEVSEHAGTKSKSQCIDHYNAVYMDSPCFPLPDMSHVMGKT

REELLAMARGNVEMKKELSAFEELTLNQESPFSVKINEASKKEDLASHSSSIVNAEVSSH

MGSSSGNTFSDAVKKASNEAQIKDKIKVEEPLSDRSIREKKPRICGEEGPSMTELSGYNF

KRQEFEIEYDNDAEQLLADMEFKDTDTDAELDMKLQVLRIYSKRLDERKRRKDFILERNL

FYPDAFEKNISPEEKEIYQRYKVFMRFHTKEEHEELMKTVIEDHQIMKRIQDLQEARAAG

CQTAGEAQGFIEQKRKKEAEESAQRAKESMQAGPAGKLLPKPNHLDSSPRGAVKCSTVFH

PGGNDSSSMIAKQAISSTLDEWDIAGFLGADLLSESDKRLCCELRILPAHYLNMLHIMSI

EITKGTVTNKTDAHSLFKVESSKVDRVYDMLVKKGIALA

>XP_011010467.1 PREDICTED: transcriptional adapter ADA2a isoform X2 [Populus euphratica]

MGRSRGRPPSSGTSSAAAASDDPNNRSSKRKKTTSNVGSIETAFPAVYQETSQGKLALYH

CNYCYKDISGMVRIKCAVCPDFDLCLGCFSVGAEVTPHKSNHPYRVMDNLSFPLFHPDWN

TDEEILLLEGIEMYGFGNWTEVSEHVGTKSKSQCIDHYNAVYMDSPCFPLPDMSHVMGKT

REELLAMARGNVEMKKELSAFEELTLNQESPFSVKIKSEALKKEDLASHSSSIVNAEVSS

HMGSSSGNTFSDAVKKASNEAQIKDKIKVEEPLSDRSIREKKPRICGEEGPSMTELSGYN

FKRQEFEIEYDNDAEQLLADMEFKDTDTHAELDMKLQVLRIYSKRLDERKRRKDFILERN

LFYPDAFEKNLSPEEKEIYQRYKVFMRFHTKEEHEELMKTVIEDRQIMKRIQDLQEARAA

GCRTAGEAQGFIEQKRKKEAEENAQRAKESMQAGPAGKLLPKPNHLDSSPHGAVKCSTIF

HPGGNDSSPMIAKQAISSTLDEWDIAGFQGADLLSESDKRLCCELRILPAHYLNMLHIMS

IEITKGTVANKTDAHSLFKVESSKVDRVYDMLVKKGIAQA

>GAV59866.1 Myb_DNA-binding domain-containing protein/ZZ domain-containing protein/SWIRM domain-containing protein [Cephalotus follicularis]

MGRSRGNFHSDEDPTQRSRRKKNTSSGDNLESASAGQGTGEGKRALYHCNYCNKDISGKI

RIKCATCPDFDLCIECFSVGADVTPHRSSHPYRVMDNLSFPLICPDWNADDEILLLEGIE

MYGLGNWAEVAEHVGTKNKEQCIEHYTNVYMNSPYFPLPDMSHVVGKNRKELLAMAKGHG

EDKKGSSLLGDLTLKEETPFSPSRVKVEEMHKGGPSGRLLSSSNAEVESGVHSNATNIAA

TSAVKKASNMAQIKDGPNNIKVEDPQTERSYGVKKPHSSANEGPSLIELSGFNHKRQEFD

PEYDNDAEQLLAEMEFKDTDTEDERELKLRVLRIYSKRLDERKRRKDFILERNLLYPNSF

EKDLSPEEKALCRRYDIFMRFHSKEEHEELLQTVISEHRTLKRIHELKEARAAGCRTSAE

ADRYMEQKKKREAEESSRRVKESAQVGPSNQGGQNVFMASESIGKDSSSRLPGQASSSHV

NDLDIMGFYETQLLSEAEKRLCCEIRLPPPLYLKMQEVMSVEIINGNVSTKSDAHHLFKI

ESSKIDRVYDMLVKKGLAQP

>GAV86755.1 Myb_DNA-binding domain-containing protein/ZZ domain-containing protein/SWIRM domain-containing protein [Cephalotus follicularis]

MGRSRAVPRSNDDDLNQSRSKRKRTASSVENLETTSTVQGLIDGKVALYHCNYCNKDISG

VVRIKCVVCPDFDLCIECFSVGAEVTPHKSNHPYRVMDNLSFPLICPEWNADEEILLLEG

IEMYGFGNWAEVAEHVGTKSKLHCIDHYNAIYMNSPCFPLPDMSHVMGKCREELLAMAKG

HGEGKKETPTLDELIVKEESPVSASVRYEVPRKEDQPYQFSSSLTAEAGPRMSLSSDNAL

SGAHKKAFNVVQIKDGIKEEETRAEVPSDRSIGEKKPRVSGDKGPSMTELSGYNFKRQEF

EIEYDNDAEQLLADMEFKDTDTDAERDLKLQVLRIYSKRLDERKRRKDFILERNLLYSDP

IEKNLSPDEREIYKRYKVFMRFHSKEEHEELLKSVIEEHRIVKRIQDLQEARAAGCLTAA

EANRFLEEKRKKEFEESGQRVRESSQVGPSGKVLQKPNQLKGEMDDSPRGVFRGFSGLQP

GGMQSSLTVTGQVIPGSLDDWDITGFVGSDLLAETEKQLCGEIRILPSHYLNMLQTLSVE

VLKGNISTKSDAHNLFKVDPSKVDRVYDMLVKKGIAHA

>PON90270.1 Transcriptional adaptor [Trema orientale]

MGRSRGNFHSPDEDATQRSRRKKNASSGENLESATAGQGTSEGKALYHCNYCNKDITGKI

RIKCCTCADFDLCIECFSVGAEVTPHKSNHPYRVMDNLSFPLICPDWNADDEILLLEGIE

MYGLGNWLDIAEHVGTKSKEQCINHYSNVYMNSPFFPLPDMTHVVGKNRKELLAMAKGHS

EEKKGLPMIAELNLKEESPFSPSRIKIEDSHKVGSSSRLLSSLNTDSGPRSGGINVVATA

PNKKPNVTQVKDGPGIVKVEDPQADRNFIGKKPISGNEGSSLVELSGYNPKRQEFDPEYD

NDAEQLLADMEFKEADTEEERDLKLRVLRIYTKRLDERKRRKDFILDRNLLYPNHFEKDL

SPEERAICRHYDVFMRFHSKEEHEELLQTVISEHRTLKRIQELKEARGAGCRTSVEAERF

LEQKRKREAEENARGTKESAHVGPSSQGGPNAYMASELTAKDTNSRPAGRVTLNPIGDMD

VMGFNGADLLSEAEKRLCSETKLPPPVYLKMQEVMSVQIFSGNITKKSDAHHLFKIEPSR

IDRVYDMLVKKGIAQP

>PON38112.1 Transcriptional adaptor [Parasponia andersonii]

MGRSRGKFHSPDEDPTQRSRRKKNASSGENLESATAGQGTSEGKALYHCNYCNKDITGKI

RIKCCTCADFDLCIECFSVGAEVTPHKSNHPYRVMDNLSFPLICPDWNADDEILLLEGIE

MYGLGNWLDIAEHVGTKSKEQCINHYTNVYMNSPFFPLPDMTHVVGKNRKELLAMAKGHS

EEKKGLPMIAELNLKEDSPFSPSRIKIEDSHKGGSSSRLLSSLNADSGPRSGGINVVANA

PNKKPNVTQVKDCPGIVKVEDPQADRNFIGKKPISGNEGSSLVELSGYNPKRQEFDPEYD

NDAEQLLADMEFKEADTEEERELKLRVLRIYSKRLDERKRRKDFILDRNLLYPNHFEKDL

LPVERTICRRYDVFMRFHSKEEHEELLQTVISEHRTLKRIQELKEARGAGCRTSVEAERF

LEQKRKREAEENACRTKESAHVGPSSQGGPNAYMASELTAKDTNSRPARQVTLNPIGDMD

VMGFNGADLLSEAEKRLCSETKLPPPIYLKMQEVMSVEIFSGNINKKSDAHHLFKIEPSR

IDMVYDMLVKKGIAQP

>XP_030491066.1 transcriptional adapter ADA2b [Cannabis sativa]

MGRSRGNFHSPDEDPTQRSRRKKNASSGENLESTTAGQGTSEGKALYHCNYCNKDITGKI

RIKCCTCADFDLCIECFSVGAEVQPHKSNHPYRVMDNLSFPLICPDWNADDEILLLEGIE

MYGLGNWTEIAEHVGTKSKEQCINHYTDVYMNSPLFPLPDMSHVVGKNRKELLAMAKGHS

EEKKGLPMISELDIKEESPFSPSRIKLEDSHKAGSSSRLLSSLNTDSGPRSSGANVGAAN

KKPNATHVKDGPIVKVEDPHTDRGFKGKRPVLGNEGSLVEMSGYNSKRLEFDPEYDNDAE

QLLADMEFKDADTEEEREIKLRVLRIYAKRLDERKRRKDFILERNLLYPNNFENDLLPEE

KGICRRYDVFMRFHSKEEHEELLRTVISEHRTLKRIQELKEARAAGCRTSVEANRYLEQK

RKREVEDVGGRTKEGAHVGPSSQGGGFMASIGDMDVMGFNGADLLSEAEKRLCSETRLTP

PIYLKMQEVMSVEIFSGNITKKSDAHQLFKIDPSRIDKVYDMLVKKGIAQP

>PON55049.1 Transcriptional adaptor [Trema orientale]

MGRSRAVSKIADNDPNQSKSSKRKRIASNSESAETTAYNTVLGQGNGDRKAPLYHCNYCN

KDISGKIRIKCVVCPDFDLCVECFSVGAEVTPHKSNHPYRVMDNLYFPLICPDWNADEET

LLLEGIGMYGFGNWNEVAEHVGTKSRQQCIDHYNAIYMNSPCFPLPDLSHVVGKSKDELL

AMAKGPSEVKIENPMVGELPPNEESPISARVKNEEYKKDPGPQSSTHPTAEDGLDSVKMA

TNESQIKDEIKVEESQVDRSIGEKKPRISGDEGPSMTELSGYNFKRQEFEVEYDNDAEQL

LADMEFKDTDTDAERELKLRVLRIYSKKLDERKRRKDFILERNLLYPDPFEKNLSPEEKE

IYQRFKVFMRFHSKEEHEEFLRIIIEEHRIMKRIQDLKEARAAGCQTAAEANRFIAQKRR

KSEESALRIRESSQAGPSGKVLQKPNHLKGELDGSPRGVVRGSMDLHPGTKDSSLAMKSI

ASSLDDWDITGFAGADLLSETEKQLCCEIRILPSHYLNMLQIMSVEVLNGKVTKKSDAYN

LFKVEPNKVDRVYDMLVKKGIAQP

>PON46048.1 Transcriptional adaptor [Parasponia andersonii]

MGRSRAVSKIADNDPNQSKSSKRKRIASNSESAETTAYNTVLGQGNGDRKAPLYHCNYCN

KDISGKIRIKCVVCPDFDLCVECFSVGAEVTPHKSNHPYRVMDNLYFPLICPDWNADEET

LLLEGIGMYGFGNWNEVAEHVGTKSRQQCIDHYNAIYMNSPCFPLPDLSHVVGKSKDELL

AMAKRPGEVKIENPMVGELPPNEESPVSVRVKNEEYKKDPGPQSSTHPTAEDGLDSVKMA

PNESQIKDEIKVEESQVDRSIGEKKPRISGDEGPSMTELSGYNFKRQEFEVEYDNDAEQL

LADMEFKDTDTDAERELKLRVLRIYSKKLDERKRRKDFILERNLLYPDPFEKNLSPEEKE

IYQRCKVFMRFHSKEEHEEFLRIIIEEHRIMKRIQDLKEARAAGCQTAAEANRFIAQKRR

KSEESALRIRESSLAGPSGKVMQKPNHLKGELDGSPRGVVRGSMDLHPSSKDSSLAMQSI

ASSLDDWDITGFAGADLLSETEKRLCCEIRILPSHYLNMLQIMSVEVLNGKVTKKSDAYN

LFKVEPNKVDRVYDMLVKKGIAQP

>XP_030483919.1 transcriptional adapter ADA2a [Cannabis sativa]

MGRSRAVSKIADSDLNQSKSSKRKRIALNSDSAENAAYNVVLGQVNGDRKAPLYHCNYCN

KDISGKIRIKCVVCSDFDLCIECFSVGAEVTPHKSNHPYRVMDNLYFPLICPDWNADEET

LLLEGIGMYGFGNWNEVAEHVGTKSKAQCIDHYDAIYLKSPCFPLPDLSHVMGKSKDELL

AMAKVPGEVKTENPMIIELPPSEEPPLSAIVKYEETKKDPSSTYSSHPTTEVGGDQVKPP

NESMIKDEIKAEESQVDRSIGEKKPRLSGDEGPTLTELSGYNIKRHEFEIEYDNDAEQLL

ADMEFKDTDTEAERELKLRVLRIYSKKLDERKRRKDFILERNLLHPNPFEKNLSPDEKEI

YQRFKVFMRFHSKEEHEEFLRIIIEEHRIIKRIQDLKEARAVGCQTAAEANKFIQQKRSN

EESALRIRESFQAGPSGKVLHKPGHIKEELDGSPLGLVRGSIDLHSSTKDPSLAMQSVAN

SLDHWDITGLAGADLLSETEKQLCSEIRILPSHYLNMLQIMSVEVLNGKVTKKSDAYNLF

KVDPNKVDRVYDMLVKKGIAQA

>XP_010087637.1 transcriptional adapter ADA2b [Morus notabilis]

MGRSRGNFHSADEDPHQRSRRKKHSSSGENSESASAGQGTSEGKALYHCNYCDKDITGKI

RIKCFVCPDFDLCIECFSMGAEVTPHKSNHPYRVMDNLSFPLICPDWNADDEILLLEGIE

MYGLGNSAEVAEHVGTKSKEQCINHYTNVYMNSPFFPLPDMSHVGKNRKELLAKAKGQSE

DKKGFPTISELNLKEESPFSSARIKVEDSHKGGSSRLLSGFNADTESGSRSSGANASAAA

PNKKPSKGTQVKDAPSIVKVEDPPADRGMGKKPVSVNEGPLVELSGYNPKRQEFDPEYDN

DAEQLLADMEFKDADTEEERELKLRVLRIYSKRLDERKRRKDLILERNLLYPKPFEKDLS

PEERAICRRYDIFMRFHSKEEHEELLQTVISEHRTKKRIQELKEARAAGCRTSVEADTYL

EQKRKREAEENACRVKESPRVGTSSQGGPNAFMASDSKDTNSRPTGQAASSSVVDMDIMG

FNGADLLSEAEKRLCGEMRLAPPIYLKMIEIMSIQIFSGNMTKKSDAHHLFKIEPSKIDR

VYDMLVKKGIAQP

>XP_024026327.1 transcriptional adapter ADA2a isoform X2 [Morus notabilis]

MGRSRAVSKIADDDPNQRSSKRKRIASNAEPAETTPYNAVLGQGNGERKAPLYHCNYCNK

DISGKIRIKCVICPDFDLCIECFSVGAEVTPHKSNHPYRVMDNLAFPLICPDWNADEETL

LLEGIGMYGFGNWAEVAEHVGTKSKAQCIDHYNSIYMNSPCFPLPDLSHVMGKSKDELLA

MAKGPGVDVKIEVPLVGELQPKDESPISARIKYEESRKGPGPQSSTHPTEVGVALVSGSV

EMAPNKPQIKDETKVEEPHVDRSIGEKKPRISGDEGPSITELSGYNFKRQEFEIEYDNDA

EQLLADMEFKDSDTDAERELKLRVLRIYSKRLDERKRRKDFILERNLLYPDPFEKNLSPE

EREIYQGFKVFMRFHSKEEHEALLRNIIEEHHIVKRIQDLKEARAAGCRTADEAKTFIEQ

KRKEAEETALRIKEGSQAGPSVKVLQKPNHLKGELDGSPRGLVRGATDLNPSNKDSSLAM

RSIASSLDDWDITGFAGADLLSETEKRLCREIRILPAHYLNMLQTMSVEVLNGKVTKKAD

AYNLFNVDRNKVDRVYDMLVKKGIART

>XP_024930807.1 transcriptional adapter ADA2b isoform X4 [Ziziphus jujuba]

MGRSRGNFHSADDDPTQRSRRKKNASSGENLESSGAGQGSIDGKRALYHCNYCNKDITGK

IRIKCCVCPDFDLCIECFSVGAEVTPHKNNHPYRVMDNLSFPLICPDWNADDEILLLEGI

EMYGLGNWTEVAEHVGTKTKEQCIEHYTTVYKNSPLFPLPDLSHVVGKNRKELLAMAKGH

SEDRKGFPTLGELNLKEESPFSPSRIKVEDQHKDAEPGVRSGGTKSAAKAANKKPSKMAQ

VKDGPSIVKVEDAQADRGFKGKKPNMGNDGPLVELSGYNPKRQEFDPEYDNDAEQLLAEM

EFKDADTDEERELKLRVLRIYSKRLDERKRRKDFILERNLLHPNPFEKDLSPEERAICRR

YDVFMRFHSKEEHQELLETIISEHRTLKRIQELKEAQAAGCRTSAEADRYLEHKRKREAE

ESARRAKENAQVGPSSQGVPNMFMASESIGKDSNSRPAGHAASSSVNDMDIMGLNGADLL

SEAEKRLCSEVRLQPTVYLKMQEVMTIEFCSGNVSKKSDAYNLFKIEPSKIDRVYDMLVK

KGIVHP

>KAF3451863.1 hypothetical protein FNV43_RR07959 [Rhamnella rubrinervis]

MGRSRGNFHSGDDDPTQRSRRKKNASSGENLESSGAGQGATEGKRALYHCNYCNKDITGK

IRIKCVMCPDFDLCIDCFSVGAEVTPHKNNHPYRVMDNLSFPLICPDWNADDEILLLEGI

EMYGLGNWAEVAEHVGTKTKEQCIEHYTTVYMNSPFFPLPDMTHVVGKNRKELLAMAKGH

SEDKKGLFFFPWELNLKEESSFSPSRIKYTQDMHKGGPSSSLLSSLNTDAEPGVRSSGAN

SAATAANKKPSKMTQVKDSPTIVKVEDAQADRSFKGKKPNLGNDGSLVELSGYNPKRQEF

DPEYDNDAEQLLADMEFKDNDTEEEREIKLRVLRIYSKRLDERKRRKEFILERNLLYPNY

FEKNLSPEERAICRRYDVFMRFHSKEEHEELLETIVAEHRTLKRIQELKEAQSAGCRTSV

EADRYLENKRKREAEDSACRAKETAQVGPSSQGVPNAFMASQSIGKDSNSRPVGQATSSS

VNDMDIMGFNGADLLSEAEKRLCSEIRLHPHIYLKMQEVITVEICSGNVSKKSDAHNLFK

IEPGKIDRVYDMLVKKGIALP

>XP_015900772.1 transcriptional adapter ADA2-like isoform X1 [Ziziphus jujuba]

MGRSRAVSRAADDDPNQRSKRKRPASGAEPIEITSYNTVLGQGNSEPKGALYHCNYCNKD

ISGKIRIKCVICPNFDLCIECFSVGAEVTPHNSKHPYRVMDNLAFPLICPDWNADEESLL

LEGIEMYGYGNWSEVAEHVGTKSKAQCIDHYNAIYMNSPFFPLPDLSHVMGKTREELLTM

AKEIKKAEAPMLGEVPVEEESLRYNESKKGPIPQSSARSTSEVGTGPVFGAVKKTSNRTQ

INHEAKAEESQVETQVDRSIGEKKPRSLGDERPSMMELSGYNFKRQEFEIEYDNDAEQLL

ADMDFKDSDTSAERELKLRVLHIYSKRLDERKRRKDFILERNLLYPDPIEKNLSPEEKEI

YQRYKVFMRFHSKKEHEEFIKNIIEEHRIVKRIQDLQQARAAGCRTANEANRFIEQKRKR

ETEESALRIKESSQAGPSGKGLQKPNHVKGEADCNSPRGLARGSTSLHLGGKESTLTTQP

IIASSLDNWDISGLRGSELISDTEKQLCNEIRILPSHYLNMLQIISTEMWKGNVTKKSDA

YSLFKVEPSKVDKVYDMVMKKGITTSS

>KAF3443260.1 hypothetical protein FNV43_RR12942 [Rhamnella rubrinervis]

MGRSRAVSRPVDDDPNQRAKRKRPASGAEPVDTTSYNAVLSQGHNELKRALYHCNYCDKD

ISGRIRIKCVVCPDFDLCIECFSVGAEVNPHKSNHPYRVMDNLSFPLICPEWNADEETLL

LEGIEMYGFGNWSEVAEHVGTKSKAQCISHYNAIYMNSPCFPLPDMSHVKGKNKEELLAM

AKEVKKEVPMVGEVPLKEEPPTSARKGPAPQSSIPSVSEVDTVSGLDAVKKTSNLSHINI

ATKEEESQVDRSIGEKKPRLLGDKKPSLTELSGYNFKRQEFEIEYDNDAEQMLADMEFKD

SDTNIERELKLRVLRIYSKRLDERKRRKNFILERNLLYPDTLEKTLSPEEKEIYERFKVF

MRFHSKEQHEEFIKNIIEEHHIVQRIQDLQQARAAGCRTAEEAYRFIEEKGKKEAEESAL

RIKEGLQAGPSGKGLPKPSNVKGEVDASSPRGLTRVSTSLSAKDSTLTAGPIVSSLDDWD

IAGLTGSELLSDTEKQLCREIRILPSHYLKMLQTIATEVWNGNVTKKTDAHRLFMVDPSK

VDKVYDMVVEKGITSA

>XP_028954756.1 transcriptional adapter ADA2b [Malus domestica]

MGRSRGNFHSDEDPTQRSRRKKNASIGENLESSAAGQGTSEGKRDYHCNYCNKDITGKVR

IKCCMCPDFDLCIECFSVGAEVTSHKSNHSYRVMDNLSFPLICPDWNADDEILLLEASEM

YGLGNWAEVAEHVGTKSKEQCIEHYTNVYLNSQYFPLPDMSHVVGKNRKELLAMAKGHGE

DKKGFPTLGDHNLKEESPFSPSRTKVEDTHKGGPSGRLMSSMNSDVESGLRSSGANVAAA

AAAGNKKPSNMAQVNDAPGVIKLEDPHAERKGKKPSSLGSKGPSLVEMSGYNAKRQEFDP

EYDNDSEQLLADMEFKDTDTDEERELKLKVLRIYAKRLDERKRRKDFILERNLLYPNPFE

KDLSPEERAICRRYDVFMCFHSKEEHDELLHTVISEHRTLKRIQELKEARAAGCRTSVEA

DRYLLQKRRREAEENARRAKESGQVGPSSQGGPNLFISSESVGIGKDSNIRPAGQATSGS

ASEMDIMGFYGSDLLSEAEKRLCREIRLAPPVFLKIQEVISIEIFSGRVSKRSDVHHLFK

IEPNKIDRVYDMLVKKGVAQP

>KAB2637617.1 transcriptional adapter ADA2b [Pyrus ussuriensis x Pyrus communis]

MGRSRGNFHSDEDPTQRSRRKKNASIGENLESSAAGQATSEGKRDYHCNYCNKDITGKVR

IKCCMCPDFDLCIECFSVGAEVTSHKSNHSYRVMDNLSFPLICPDWNADDEILLLEATEM

YGLGNWAEVAEHVGTKSKEQCIEHYTNVYLNSQYFPLPDMSHVVGKNRKELLAMAKGHGE

DKKGFPTLGDHNLKEESPFSPSRTKVEDTHKGGPSGRLMSSMNSDVESGLRSSGANVAAA

AAGNKKPSSMAQVKDGPGVIKLEDPHAESGYNAKRQEFDPEYDNDSEQLLADMEFKDTDT

DEERELKLKVLRIYAKRLDERKRRKDFILERNLLYPNPFEEDLSPEERAICRRYDVFMCF

HSKEEHDELLHTVISEHRTLKRIQELKEARTAGCRTSAEADRYLVQKRRREAEESARRAK

ESGQVGPSSQGGPNLFMSSESVGIGKDSNTRPAGQATSGSASEMDIMGFYGSDLLSEAEK

RLCSEIRLAPPVFLKIQEVISIEIFSGRVSKRSDVHHLFKIEPNKIDRVYDMLVKKGVAQ

P

>XP_034213860.1 transcriptional adapter ADA2b [Prunus dulcis]

MGRSRGNFHSDEDPTQRSRRKKNASTGENLESSAAGAGQVTSEGKRAYHCNYCNKDITGK

IRIKCCMCPDFDLCIECFSVGAELTSHKSNHSYRVMDDLSFPLICPDWNADDEILLLEAT

EMYGLGNWAEVAEHVGTKSKEQCIEHYTNVYLNSQRFPIPDMSHVEGKNRKELLAMAKGH

GEDKKGFPMLGDHSLKEESPFSPSRTKAEDMHKGGHSSRLLSSINTDAESGLRSSGASVA

AAAGNKKPSNMAQVKDGHAVIKVEEPQADRKGKKPSSLGSAGPSLVELSGYNVKRQEFDP

EYDNDSEQLLADMEFKDTDTEDERDLKLRVLRIYSKRLDERKRRKDFILERNLLYPNPFE

KDLLPEERAICRRYDVFMCFHSKEEHEELLQTVIAEHRTMKRIQELKEARAAGCRTSAEA

DRYLEHKRKKDAEENARRAKESGQVGPSSQGGPNLFVSSESVDKDSNSRPAGQATSSSAS

DMDIMGFYGSDLLSEAEKRLCSEIRLPPPVFLKMQEVISIEIFSGNVSKKSDVHHLFKIE

PSKIDRVYDMLVKKGITQP

>XP_007211885.1 transcriptional adapter ADA2 isoform X1 [Prunus persica]

MGRSRGNFHSDEDPTQRSRRKKNASTGENLESSAAGAGQVTSEGKRAYHCNYCNKDITGK

IRIKCCMCPDFDLCIECFSVGAELTSHKSNHSYRVMDDLSFPLICPDWNADDEILLLEAT

EMYGLGNWAEVAEHVGTKSKEQCIEHYTNVYLNSQRFPIPDMSHVEGKNRKELLAMAKGH

GEDKKGFPMLGDHSLKEESPFSPSRTKAEDMHKGGHSSRLLSSINTDAESGLRSSGASVA

AAAGNKKPSNMAQVKDGPGVIKVEEPQADRKGKKPSSLGSAGPSLVELSGYNVKRQEFDP

EYDNDSEQLLADMEFKDTDTEDERDLKLRVLRIYSKRLDERKRRKDFILERNLLYPNPFE

KDLLPEERAICRRYDVFMCFHSKEEHEELLQTVIAEHRTMKRIQELKEARAAGCRTSAEA

DRYLEHKRKKDAEENARRAKESGQVGPSSQGGPNLFVSSESVDKDSNSRPAGQATSSSAS

DMDIMGFYGSDLLSEAEKRLCSEIRLPPPVFLKMQEVISIEIFSGNVSKKSDVHHLFKIE

PSKIDRVYDMLVKKGITQP

>XP_021831703.1 transcriptional adapter ADA2 [Prunus avium]

MGRSRGNFHSDEDPTQRSRRKKNASTGENLESSAAGAGQVTSEGKRAYHCNYCNKDITGK

IRIKCCMCPDFDLCIECFSVGAELTSHKSNHSYRVMDDLSFPLICPDWNADDEILLLEAT

EMYGLGNWAEVAEHVGTKSKEQCIEHYTNVYLNSQRFPVPDMSHVEGKNRKELLAMAKGH

GEDKKGFPMLGDHSLKEESPFSPSRTKAEDMHKGGHSSRLLSSINTDAESGLRSSGASVA

AAAGNKKPSNMAQVKDGPGVIKVEEPQADRKGKKPSSLGSAGPSLVELSGYNVKRQEFDP

EYDNDSEQLLADMEFKDTDTEDERDLKLRVLRIYSKRLDERKRRKDFILERNLLYPNPFE

KDLLPEERAICRRYDVFMCFHSKEEHEELLQTVIAEHRTMKRIQELKEARAAGCRTSAEA

DRYLEHKRKKDAEENARRAKESGQVGPSSQGGPNLFVSSESVDKDSNSRPAGQATSSSAS

DMDIMGFYGSDLLSEAEKRLCSEIRLPPPVFLKMQEVISIEIFSGNASKKSDVHHLFKIE

PSKIDRVYDMLVKKGITQP

>PQP96255.1 transcriptional adapter ADA2 isoform X1 [Prunus yedoensis var. nudiflora]

MGRSRGNFHSDEDPTQRSRRKKNASTGENLESSAAGAGQVTSEGKRAYHCNYCNKDITGK

IRIKCCMCPDFDLCIECFSVGAELTSHKSNHSYRVMDDLSFPLICPDWNADDEILLLEAT

EMYGLGNWAEVAEHVGTKSKEQCIEHYTNVYLNSQRFPVPDMSHVEGKNRKELLAMAKGH

GEDKKGFPMLGDHSLKEESPFSPSRTKAEDMHKGGHSSRLLSSINTDAESGLRSSGASVA

AAAGNKKPSNMAQVKDGPGVIKVEEPQADRKGKKPSSLGSAGPSLVELSGYNVKRQEFDP

EYDNDSEQLLADMEFKDTDTEDERDLKLRVLRIYSKRLDERKRRKDFILERNLLYPNPFE

KDLLPEERAICRRYDVFMCFHSKEEHEELLQTVIAEHRTMKRIQELKEARAAGCRTSAEA

DRYLEHKRKKDAEENARRAKESGQVGPSSQGGPNLFVSSESVDKDSNSRPAGQATSSSAS

EMDIMGFYGSDLLSEAEKRLCSEIRLPPPVFLKMQEVISIEIFSGNASKKSDVHHLFKIE

PSKIDRVYDMLVKKGITQP

>XP_009342203.1 PREDICTED: transcriptional adapter ADA2b [Pyrus x bretschneideri]

MGRSRGNFHSDEDPTQRSRRKKNASIGENLESSAAGQATSEGKRDYHCNYCNKDITGKVR

IKCCMCPDFDLCIECFSVGAEVTSHKSNHSYRVMDNLSFPLICPDWNADDEILLLEATEM

YGLGNWAEVAEHVGTKSKEQCIEHYTNVYLNSQYFPLPDMSHVVGKNRKELLAMAKGHGE

DKKGFPTLGDHNLKEESPFSPSRTKVEDTHKGGPSGRLMSSMNSDVESGLRSSGANVAAA

AAGNKKPSSMAQVKDGPGVIKLEDPHAERKGKKPSSLGSKGPSLVESGYNAKRQEFDPEY

DNDSEQLLADMEFKDTDTDEERELKLKVLRIYAKRLDERKRRKDFILERNLLYPNPFEED

LSPEERAICRRYDVFMCFHSKEEHDELLHTVISEHRTLKRIQELKEARTAGCRTSAEADR

YLVQKRRREAEESARRAKESGQVGPSSQGGPNLFMSSESVGIGKDSNTRPAGQATSGSAS

EMDIMGFYGPDLLSEAEKRLCSEIRLAPPVFLKIQEVISIEIFSGRVSKRSDVHHLFKIE

PNKIDRVYDMLVKKGVAQP

>PQQ04449.1 transcriptional adapter ADA2 isoform X1 [Prunus yedoensis var. nudiflora]

MGRSRGNFHSDEDPTQRSRRKKNASTGENLESSAAGAGQVTSEGKRAYHCNYCNKDITGK

IRIKCCMCPDFDLCIECFSVGAELTSHKSNHSYRVMDDLSFPLICPDWNADDEILLLEAT

EMYGLGNWAEVAEHVGTKSKEQCIEHYTNVYLNSQRFPVPDMSHVEGKNRKELLAMAKGH

GEDKKGFPMLGDHSLKEESPFSPSRTKAEDMHKGGHSSRLLSSINTDAESGLRSSGASVA

AAAGNKKPSNMAQVKDGPGVIKVEEPQADRKGKKPSSLGSAGPSLVELSGYNVKRQEFDP

EYDNDSEQLLADMEFKDTDTEDERDLKLRVLRIYSKRLDERKRRKDFILERNLLYPNPFE

KDLLPEERAICRRYDVFMCFHSKEEHEELLQTVIAEHRTMKRIQELKEARAAGCRTSAEA

DRYLEHKRKKDAEENARRAKESGQVGPSSQGGPNLFVSSESVDKDSNSRPAGQATSSSAS

EMDIMGFYGSDLLSEAEKRLCSEIRLPPPVFLKMQEVISIEIFSGNASKKSDVHHLFKIE

PSKIDRVYDMLMKKGITQP

>XP_028943516.1 transcriptional adapter ADA2-like [Malus domestica]

MGRSRGNFHSDEDPTQRSRRKKNPSSGENLESSAAGQGTSEGKRAYHCNYCNKDITGKVR

IKCCMCPDFDLCIECFSVGAEVTSHKSNHSYRVMDNLSFPLICPDWNADDEILLLEATEM

YGLGNWAEVAEHVGTKSKEQCIEHYTNVYLNSPYFPLPDMSHVVGKNRKELLAMAKGHGE

DKKGFPTLGDHNVKEESPFSPSRTKVEDTHKGGSSGHLMSSINSDVESGLRSSGANVAAA

AGNKKPSNMAQVKDGPSVIKLEDPQAERKGKNPSTLGSKDPSLVESSGYNAKRQEFDTEY

DNDSEQLLADMEFKDTDTNDERELKLKVLRIYAKRLDERKRRKDFILERNLLYPNPFEKD

LSPEEKAICRRYDVFMCFHSKEEHDELLQTVISEHRTLKRIQELKEARAAGCRTSADADR

YLLQKRRREAEENARRAKESGQVGPSSQGGPNLFMSLESVGIGKDSNSRPAGQATSGSAR

DLDTVGFYRSDLLSEAEKRLCSEIRLPPPVFLKMQEVISIEIFSGRVSKRSDVHQLFKIE

PNKIDRVYDMLVKKGIAQP

>XP_008227439.1 PREDICTED: transcriptional adapter ADA2 [Prunus mume]

MGRSRGNFHSDEDPTQRSRRKKNASTGENLESSVAGQGISEGKRAYHCNYCNKDITGKIR

IKCCMCPDFDLCIECFSVGAELTSHKSNHSYRVMDNLSFPLICPDWNADDEILLLEATEM

YGLGNWAEVAEHVGTKSKEQCIEHYTNVYLNSQRFPIPDMSHVEGKNRKELLAMAKGHGE

DKKGFPLLGDHSLKEESPFSPSRTKAEDMHKGGHSSRLLSSINTDAESGLRSSGASVAAA

AGNKKPSNMAQVKDGPGVIKVEEPQADRKGKKPSSLGSSGPSLVESSGYNVKRQEFDPEY

DNDSEQLLADMEFKDTDTEDERDLKLRVLRIYSKRLDERKRRKDFILGRNLLYPNPFEKD

LLPEERAICRRYDVFMCFHSKEEHEELLQTVIAEHRTMKRIQELKEARAAGCRTSAEADR

YLEHKRKKDAEENARRAKESGQVGPSSQGGPNLFMSSESVDKDSNSRPAGQATSSSASDM

DIMGFYGSDLLSEAEKRLCSEIRLPPPVFLKMQEVISIEIFSGNASKKSDVHHLFKIEPS

KIDRVYDMLVKKGITQP

>XP_020415167.1 transcriptional adapter ADA2a isoform X2 [Prunus persica]

MGRSRAASHPAVDNHNQRGKRKRTASGAEPAENSSTAQRAVAQETSEPKGAFYHCNYCNK

DISGKTRIKCVVCPDFDLCIECFSVGAELTPHKCNHPYRVMDNLSFPLLCPDWNADEEML

LLEGIEMYGFGNWTEVSEHVGTKSRQHCIDHFKAIYMNSPRFPLPDMSHVMGKSREELLA

LAKGSGEIKKEVPMLVEITLKEVSPFSTGVKCEELKKNPACQSSSHSTADDASGLVLGAV

KKASNKAQIKDETKVYKVEESQVDRSVGGKKLRSLGDEGPSITEFSGYNFKRQEFEIEYD

NDAEQILADMEFKDSDTNADRELKLRVLHVYSKRLDERKRRKDFILERNLLYPDPFEKNL

SPEEREIYQRFKVFMRFHSNEEHKELLKSIIEEQQIVKRILDLQEARTAGCRTAAEASRY

LEEKRKKENEESNLRIKESSQAGKGLQISPRGAFKGSTGLHPVSKDSFSTTQAISSSLDY

WDITGLVGADLLSETEQRLCSEMRILPSHYLNMLQIISTEIENGNVKKKSDAHSLFKVEP

SKVDRVYDMLVKKGMARE

>XP_008229912.1 PREDICTED: transcriptional adapter ADA2a-like isoform X2 [Prunus mume]

MGRSRAASHPAVDNHNQRGKRKRTASGTEPAENSSTAQKAVAQETSEPKGAFYHCNYCNK

DISGKTRIKCVVCPDFDLCIECFSVGGELTPHKCNHPYRVMDNLSFPLLCPDWNADEEML

LLEGIEMYGFGNWTEVSEHVGTKSRQHCVDHFKAIYMNSPRFPLPDMSHVMGKSREELLA

LAKGSGGIKKEVPMLVEITLKEVSPFSTGVKCEELKKNPACQSSSHSTADDASGLVLGAV

KKASNKAQIKDEIKVCKVEESQVDRSVGGKKLRSLGDEGPSITEFSGYNFKRQEFEIEYD

NDAEQILADMEFKDSDTNADRELKLRVLHVYSKRLDERKRRKDFILERNLLYPDPFEKNL

SPEEGEIYQRFKVFMRFHSNEEHKELLKSIIEEQQIVKRILDLQQARTAGCRTAAEASRY

LEEKRKKENEESSLRIKESSQAGKGLQVSPRGAFKGSTGLHPVSKDSFSTTQAISSSLDY

WDITGLVGADLLSETEQRLCSEMRILPSHYLNMLQIISTEIENGNVKKKSDAHSLFKVEP

SKVDRVYDMLVKKGMAQE

>KAB2631914.1 transcriptional adapter ADA2a-like [Pyrus ussuriensis x Pyrus communis]

MGRSRAVSQAAVDNHNQSRGKRKRTASGAEAAENSGTAQKSVAQEISEPKGAFYHCNYCN

KDISGKIRIKCVVCPDFDLCIECFSVGAELTPHKCNHPYRVMDNLAFPLICPDWNVDEEM

LLLEGIEMYGFGNWTEVSEHVGTKTRQQCIDHYKAIYMNSPFFPLPDMSHVMGKSREELL

ALAKGSDEIKKEVPMLVEITLKEVSPFSAEVKCEESKKNPASQSSSNSTADVTKGLVPGA

VKKASNKKQIKDETKVGKVEETQVDRSVGGKKLRSLGEEGPITEFSGYNFKREEFETEYD

NDAEQILADMEFKDTDTNADRELKLRVLHVYAKRLDERKRRKNFILERNLLYPDPFEKGL

TPEEREIYKRFKVFMRFHSSEDHKELLKNIIEEQQIVKRIQDLQEARTAGCRTASDASRY

LEEKRKKEAEESALRIKESSQAGKGLQISPRGSFKGSTVLHPFSKDSYLTTQAISSSLDY

WDVTGLAGADLLSEAERRLCSEIRILPSHYLNMLQTISIEIVNGNVKNQADARSLFKVEP

SKVDRVYDMLVQKGMAQA

>XP_021805857.1 transcriptional adapter ADA2a-like isoform X1 [Prunus avium]

MGRSRAASHPAADNHNQSRGKRKRTASGAEPAENSSTAQKAVAQETSEPKGAFYHCNYCN

KDISGKTRIKCVVCPDFDLCIECFSVGAELTPHKCNHPYRVMDNLSFPLLCPDWNADEEM

LLLEGIEMYGFGNWTEVSEHVGTKSRQHCIDHFKAIYMNSPRFPLPDMSHVMGKSREELL

ALAKGSGEIKKEVPMLVEITLKEVSPFSTGVKCEESKKNPACQSSSHSTADDASGLVLGA

VKKASNKAQIKDETKVCKVEESQVDRSVGGKKLRSLGDEGPSITEFSGYNFKRQEFEIEY

DNDAEQILADMEFKDSDTNADRELKLRVLHVYSKRLDERKRRKDFILERNLLYPDSFERN

LSPEEREIYHRFKVFMRFHSNEEHKELLKSIIEEQQIVKRILDLQEARTAGCRTAAEASR

YLEEKRTKETEESSLRIKESSQAGKGLQISPRGAFKGPHPVSKDSFLTTQAISSSLDYWD

ITGLVGADLLSETEQRLCSEMRILPSHYLNMLQIISTEIENGNVKKKSDAHSLFKVEPSK

VDRVYDMLVKKGMAQE

>XP_009343465.1 PREDICTED: transcriptional adapter ADA2a-like isoform X1 [Pyrus x bretschneideri]

MGRSRAVSQAAVDNHNQSRGKRKRTASGAEAAENSGTAQKSVAQEISEPKGAFYHCNYCN

KDISGKIRIKCVVCPDFDLCIECFSVGAELTPHTCNHPYRVMDNLAFPLICPDWNVDEEM

LLLEGIEMYGFGNWTEVSEHVGTKTRQQCIDHYKAIYMNSPFFPLPDMSHVMGKSREELL

ALAKGSDEIKKEVPMLVEITLKEVSPFSAEVKCEESKKNPASQSSSNSTADVAKGLVPGA

VKKASNKKQIKDETKVGKVEETQVDRSVGGKKLRSLGEEGPITEFSGYNFKREEFETEYD

NDAEQILADMEFKDTDTNADRELKLRVLHVYAKRLDERKRRKNFILERNLLYPEPFEKGL

TPEEREIYKRFKVFMRFHSSEDHKELLKNIIEEQQIVKRIQDLQEARTAGCRTASDASRY

LEEKRKKEAEESALRIKESSQAGKGLQISPRGSFKGSTVLHPFSKDSYLTTQAISSSLDY

WDVTGLAGADLLSEAERRLCSEIRILPSHYLNMLQTISIEIVNGNVKNQADARSLFKVEP

SKVDRVYDMLVQKGMAQA

>XP_008365226.2 LOW QUALITY PROTEIN: transcriptional adapter ADA2a-like [Malus domestica]

MGRSRAVSQAAVDNHNQSRGKRKRTASGAEAAENSGTAQKSVAQEISEPKGAFYHCNYCN

KDISGKIRIKCVVCPDFDLCIECFSVGAELTPHRCNHPYRVMDNLAFXLICPDWNVDEEM

LLLEGIEMYGFGNWTEVSEHVGTKTRQHCIDHYKAIYMNSPFFPLPDMSHVMGKSREELL

ALAKGSDEIKKEVPMLVEITLKEVSPFSAEVKCEESKNNPASQSSSNSTADVAKGLVPGA

VKKASNKKQIKDETKVSKVEETQVDRSVGEKKLRSLGEEGPITEFSGYNFKREEFETEYD

NDAEQILADMEFKDTDTNADRELKLRVLHVYAKRLDERKRRKKFILERNLLYPDPFEKGL

APEEREIYKRFKVFMRFHSSEDHKELLKNIIEEQQIVKRIQDLQEARTAGCRTASDASRY

LEEKRKKEAEESALRIKESSQAGKGLQISPRGSFKGSTVLHPFSKDSYLTTQAISSSLDY

WDVTGLAGADLLSEAERRLCSEIRILPSHYLNMLQTISIEIVNGNVKKQADARSLFKVEP

SKVDRVYDMLVQKGMAQA

>XP_024156694.1 transcriptional adapter ADA2b isoform X2 [Rosa chinensis]

MGRSRGNFHSDEDPTQRSRRKKNASSGENLESSAAGQGKSEGKRAYHCNYCNKDITGKIR

IKCNSCPDFDLCIECFSVGAELTSHKSYHPYQVLDNLSFPLICPDWNADDEILLLEATEM

YGLGNWAEVAEHVGTKSKEQCIEHYTNVYLNSPYFPLPDMSHVVGKNRKELLAMAKGHGE

DKKGFPMLGDHNVKEESPFSPSRIKLEDSHKGGPSSRLLSSINTDAESGVRSSGASVAAT

AGHKKPSNMTQIKDGPGVFKVEEPQVGRKGKKPSSLVNEGPSLVEMSGYNPKRQEFDPEY

DNDSEQLLADMEFKDNDTEDERELKLRVLRIYSKRLDERKRRKDFILERNLLYPNPFEKD

LSPEEKAICRRYDVFMCFHSKEEHTELLQTVIGEHRTLKRIQELKEARNAGCRTSSEADR

YLEQKRKREAEENARRARESGEVGPSQGNPNLSSASEMDILGVYGSDLLSEAEKRLCSEM

GLPPPFYLKMEEVISIEIFSGNVTKRSDAHHLFKIEPSKIDRVYDMLVKKGIAQS

>XP_004293386.1 PREDICTED: transcriptional adapter ADA2b isoform X2 [Fragaria vesca subsp. vesca]

MGRSRGNFHSDEDPTQRSRRKKNASSGENLESLAAGQGKSEGKRAYHCNYCNKDITGKIR

IKCNYCPDFDLCIECFSVGAELTSHKSNHPYQVLDNLSFPLICPDWNADDEILLLEATEM

YGLGNWAEVAEHVGTKSKEQCIEHYTNVYLNSPYFPLPDMSHVVGKNRKELLAMAKGHGE

DKKGFPMLGDHNVKEESPFSPSRIKVEDSHKGGPSSRLLSSINTDAESGVRSSGVAAIAG

HKKPSNMTQVKDGPGVFKVEEPQVGRKGKRPSSLVNEGPSLVESGYNPKRQEFDPEYDND

SEQLLADMEFKDNDTEDERELKLRVLRIYAKRLDERKRRKDFILERNLLYPNPFEKDLNP

EEIAICRRYEVFMCFHSKEEHAELLQTVIAEHRTRKRIQELKEARNAGCRTSAEADRYLE

QKRKREADENARRAKESGEVGPSGQAGPNLSSASELDILGVYGSDLLSEAEKRLCSEMGL

PPPVYLKMEEVMSIEIFSGNVTKRSDAHHLFKIEPSKIDRVYDMLVKKGIAQS

>XP_011467297.1 PREDICTED: transcriptional adapter ADA2 isoform X2 [Fragaria vesca subsp. vesca]

MGRSRAVAQSAPGDSNQRGKRKRTASGAEVAENSSAAQKGLAQELSEPKGAFYHCNYCQK

DISGKIRIKCGVCPDFDLCIECFSVGVEVTPHKCNHPYRVMDNLSFPLICPDWNADEEML

LLEGIEMYGFGNWTEVSEHVGTRRRQQCIDHYNAIYMNSPCFPLPDMSRVMGKSREDLLA

LSEGSGEIKKELPMFMEITLKEESPFSPRVKSEEPKKTAASKASSHSTADGASGAGKKAS

NKLQIKDEPKVTKVEESQRTIGGKKLRSLVEEGPSVTELSGYNYKRQEFEIEYDNDAEQI

LADMEFHDTDTDGDRELKLRVLHVYGKRLDERKRRKDFVLERNLLHPDPLEKNLSSEVRE

IHQRFKVFMRFHSNEDHKELLTSIIEEQQIVKRILDLQEARAAGCRTATEASNYLEQKRK

NESEESLLKIKENAQAGASVKILQQISPRGAARGSAILHPGPKDLSLITQPISNSLDDWD

ISGLMGADLLSETEKRLCCELRILPSHYLHMLQTISTETLNGNVKKKSDAHSLFKVDPDK

VDKVYDMVIQKGLTLA

>XP_024185528.1 transcriptional adapter ADA2-like isoform X3 [Rosa chinensis]

MGRSRAVAQSAVGDSNQRGKRKRTASGAELGENSSTAQKGLAQELSEPKGAFYHCNYCQK

DISGKIRIKCGVCPDFDLCIECFSVGVEVTPHKCNHPYRVMDNLSFPLICPDWNADEEML

LLEGIEMYGFGNWTEVSEHVGTRRRQQCIDHYNAIYLNSPCFPLPDMSHVMGKSREDLLA

LAKGSGQIKKELPMFMEITLKEVSPFSAGAKCEEPKKTAASKSSSHSTADGASGAGKKAS

NKLQIKDEPKVTKVEAESQRSIGEKKLRSLVEEGPSVTELSGYNYKRQEFEIEYDNDAEQ

ILADMEFLDTDTDRDRELKLRVLHVYGKRLDERKRRKDFVLERNLLHPDPLEKNLSPEVG

EIYQRFKVFMQFHSNEDHKELLKSIIEEQQIVKRILDLQEARAAGCRTATEATKYLEQKR

KNESEESLQKIKENSQAGASGKVLQQISPRGAVRGSALLHPGIKDLPLTTQPIPNPLDDW

DISGLAGANLLSETDKRLCCELRIIPSHYLHMLQTISTEISSGNVKKKSDAHSLFKAEPS

KVDRVFDMVIRKGLTLA

>XP_034698673.1 LOW QUALITY PROTEIN: transcriptional adapter ADA2b [Vitis riparia]

MGRSRGVFSPLEDPTQRSRRKKNASSGENLDSAAAGQGSSEGKKALYHCNYCNKDITGKI

RIKCAVCPDFDLCIECFSVGAEVTSHKSNHPYRVMDNLSFPLICPDWNADDEILLLEGIE

MYGLGNWTEVAEHVGTKTKEPCIEHYANVYMNSPYFPLPDLSHVVGKNRKELLAMAKGHS

DDKKGFSLLGELTLKEESPFSPSRVKVEDTHKGGPSGRLLSVLNADVDSTVRSSGTNVAA

TATVKKASNMAQVKDGPNVVKVEDPQIDRNFGGKKPNSGAEGSSLVELSGYNSKRHEFDP

EYDNDAEQLLAEMEFKEPDTEDERELKLRVLRIYAKRLDERKRRKDFILERNLLHPNPFE

KDLSPEEKELCQRYDIFMRFHSKEEHEDLLKTIISEHRTLKRIQELKEARAAGCRTSAEA

DRYLEQKRRREAEEHARRVKESAQGGTSGQGAQNVFMASESVGKDANSRTAGQATSSSVN

DFDVMGCPEAELLSETEKRLCSEIRLAPAHYLKMQETLSVEIFQGNVTKKSDAHRLFKIE

PSKVDRVYDMLAKKGIAQL

>RVW80464.1 Transcriptional adapter ADA2b [Vitis vinifera]

MGRSRGVFQSPDEDPTQRNAGLRELDVGLCFPADAERSRRKKNASSGENLDSAAAGQGSS

EGKKALYHCNYCNKDITGKIRIKCAVCPDFDLCIECFSVGAEVTSHKSNHPYRVMDNLSF

PLICPDWNADDEILLLEGIEMYGLGNWTEVAEHVGTKTKEPCIEHYANVYMNSPYFPLPD

LSHVVGKNRKELLAMAKGHSDDKKGFSLLGELTLKEESPFSPSRVKVEDTHKGGPSGRLL

SVLNADVDSTVRSSGTNVAATATVKKASNMAQVKDGPNVVKVEDPQIDRNFGGKKPNSGA

EGSSLVELSGYNSKRHEFDPEYDNDAEQLLAEMEFKEPDTEDERELKLRVLRIYAKRLDE

RKRRKDFILERNLLHPNPFEKDLSPEEKELCQRYDVFMRFHSKEEHEDLLKTIISEHRTL

KRIQELKEARAAGCRTSAEADRYLEQKRRREAEEHARRVKESAQGGTSGQGAQNVFMASE

SVGKDANSRTAGQATSSSVNDFDVMGCPEAELLSETEKRLCCEIRLAPAHYLKMQETLSV

EIFQGNVTKKSDAHRLFKIEPSKVDRVYDMLAKKGIAQL

>XP_034702310.1 transcriptional adapter ADA2-like [Vitis riparia]

MGRSRAVLHSTDDDQGSHRSKRRKTASTADNLEGATAGQGMSEGKRASYHCNYCSKDISG

KIRTKCVVCPDFDLCIECFSIGAEVTPHVCFHPYRVMDNLSFPLICPDWNADEEMLLLEG

IEMYGLGNWSEVSEHVGTKRKSECIDHYVATYMNSPCFPLPDMSHVLGKTRAELLAMARG

EDEVKKGSPTHGELTLKVESPLSARVKVEESRKEEIQSDMVSSSTKTSAGAVKRASNMAQ

VKDGRDNIKVEETQTDRSVGEKKPRTSGDEGPSVTELSGYNFKRQEFDVEYDNDAEQLLA

DMEFKDTDTDAEHELKLQVLHIYSKRLDERKRRKDFILERNLLYPDPFEKNLSPEERDVN

QRFKVFMRFHSKEEHEELLRVVLEEHWIQKRIQDLQEARAAGCRTSVEAERYLEEKGKKE

AEESAQRAKESAEAGPSGGKVLQRVNTAKGESDGSPRGGGRDSAGLEPGIKDSSSTTAGH

AILRSLDVWDITGFPGEDLLSETEKQLCSEIRILPSHYLNMLHTMLTETLNGNITRKSDA

HGLFKVEPSKVDKVYDMFVKKGIVKS

>XP_010657463.1 PREDICTED: transcriptional adapter ADA2 [Vitis vinifera]

MGRSRAVLHSTDDDQGSHRSKRRKTASTADNLEGATAGQGMSEGKRASYHCNYCSKDISG

KIRTKCVVCPDFDLCIECFSIGAEVTPHVCFHPYRVMDNLSFPLICPDWNADEEMLLLEG

IEMYGLGNWSEVSEHVGTKRKSECIDHYVAIYMNSPCFPLPDMSHVLGKTRAELLAMARG

EDEVKKGSPTHGELTLKVESPLSARVKVEESRKEEIQSDMVSSSTKTSAGAVKRASNMAQ

VKDGRDNIKVEETQTDRSVGEKKPRTSGDEGPSVTELSGYNFKRQEFDVEYDNDAEQLLA

DMEFKDTDTDAEHELKLQVLHIYSKRLDERKRRKDFILERNLLYPDPFEKNLSPEERDVN

QRFKVFMRFHSKEEHEELLRVVLEEHWIQKRIQDLQDARAAGCRTSAEAERYLEEKGKKE

AEESAQQAKESAEAGPSGGKVLQRVNTAKGESDGSPRGGGRGSAGLEPGIKDTSSTTAGH

AILRSLDVWDITGFPGEDLLSETEKQLCSEIRILPSHYLNMLHTMLTETLNGNITRKSDA

HGLFKVEPSKVDKVYDMFVKKGIVKS

>NP_567495.1 ADA2 2B [Arabidopsis thaliana]

MGRSRGNFQNFEDPTQRTRKKKNAANVENFESTSLVPGAEGGGKYNCDYCQKDITGKIRI

KCAVCPDFDLCIECMSVGAEITPHKCDHPYRVMGNLTFPLICPDWSADDEMLLLEGLEIY

GLGNWAEVAEHVGTKSKEQCLEHYRNIYLNSPFFPLPDMSHVAGKNRKELQAMAKGRIDD

KKAEQNMKEEYPFSPPKVKVEDTQKESFVDRSFGGKKPVSTSVNNSLVELSNYNQKREEF

DPEYDNDAEQLLAEMEFKENDTPEEHELKLRVLRIYSKRLDERKRRKEFIIERNLLYPNP

FEKDLSQEEKVQCRRLDVFMRFHSKEEHDELLRNVVSEYRMVKRLKDLKEAQVAGCRSTA

EAERYLGRKRKRENEEGMNRGKESGQFGQIAGEMGSRPPVQASSSYVNDLDLIGFTESQL

LSESEKRLCSEVKLVPPVYLQMQQVMSHEIFKGNVTKKSDAYSLFKIDPTKVDRVYDMLV

KKGIAQL

>XP_020873971.1 transcriptional adapter ADA2b isoform X1 [Arabidopsis lyrata subsp. lyrata]

MGRSRGNFQNFEDPTQRTRKKKNAANVENFESTSMVPGTEGGGKYNCDYCQKDITGKIRI

KCAVCPDFDLCVECMSVGAEITPHKCDHPYRVMGNLTFPLICPDWSADDEMLLLEGLEIY

GLGNWAEVAEHVGTKSKEQCLEHYRNIYLNSPFFPLPDMSHVAGKNRKELQAMAKGRIDD

KKAEQNMKEEYPFSPPKVKVEDTQKESFIDRSFGGKKPVTTSVNNSLVELSNYNQKREEF

DPEYDNDAEQLLAEMEFKENDTPEEHELKLRVLRIYSKRLDERKRRKEFIIERNLLYPNP

FEKDLSQEEKVQCRRLDVFMRFHSKEEHDELLRSVVSEYRMVKRLKDLKEAQVAGCRSTA

EAERYLGRKRKRENEEGMNRGKESGQFGQIAGEMGSRPPVQASSSYVNDLDLIGFTESQL

LSESEKRLCREVKLVPPVYLQMQQVMSHEIFKGNVTKKSDAYSLFKIDPTKVDRVYDMLV

KKGIAQL

>XP_010449793.1 PREDICTED: transcriptional adapter ADA2b isoform X1 [Camelina sativa]

MGRSRGNFHNYEDPTQRTRKKKNAANVENFESTSTVPGTEGGGKYNCDYCQKDITGKIRI

KCAVCPDFDLCVECMSVGAEITPHKCDHPYRVMGNLTFPLICPDWSADDEMLLLEGLEIY

GLGNWAEVAEHVGTKSKEQCLEHYRNIYLNSPFFPLPDMSHVAGKNRKELQAMAKGRIDD

KKAEQNMKEEYPFSPPKVKVEDTQKESHIDRSFGGKKPVATPVNNSLVELSNYNQKREEF

DPEYDNDAEQLLAEMEFKDNDTPEEHELKLRVLRIYSKRLDERKRRKEFIIERNLLYPNP

FEKDLSQEEKVQCRRLDVFMRFHSKEEHDELLRSVVSEYRMVKRLKDLKEAQVAGCRSTA

EAERYLGRKRKRENEEGMNRGKESGQFGQIVGEIGSRPPVQASSSYVNDLDLIGFTESQL

LSESEKRLCNEVKLVPPVYLQMQQVMSHEIFKGNVTKKSDAYSLFKIEPTKVDRVYDMLV

KKGIAQL

>XP_006285376.1 transcriptional adapter ADA2b isoform X1 [Capsella rubella]

MGRSRGNFHNYEDPTQRTRKKKNAANVENFESTSMVPGTEGGGKYNCDYCQKDITGKIRI

KCAVCPDFDLCVECMSVGAEITPHKCDHPYRVMGNLTFPLICPDWSADDEMLLLEGLEIY

GLGNWAEVAEHVGTKSKEQCLEHYRNIYLNSPFFPLPDMSHVAGKNRKELQAMAKGRIDE

KKAEQNMKEEYPFSPPKVKVEDTQKESHTDRSFGGKKPVAPAVNNTLVEMSNYNQKREEF

DPEYDNDAEQLLAEMEFKETDTPEEHELKLRVLRIYSKRLDERKRRKEFIIERNLLYPNP

FEKDLSQEEKVQCRRLDVFMRFHSKEEHEELLRSVVSEYRMVKRLKDLKEAQVAGCRSTA

EAERYLGRKRKRENEEGMNRGKESGQFGQIVGEMGSRPPVQASSSYVNDLDLIGFTESQL

LSESEKRLCSEVKLVPPVYLQMQQVMSHEIFKGNVTKKSDAYSLFKIEPTKVDRVYDMLV

KKGIAQL

>XP_006414353.1 transcriptional adapter ADA2b isoform X1 [Eutrema salsugineum]

MGRSRGNFHNFEDPTQRTRKKKNAANVENFESTSMVPGTEGGGKYNCDYCQKDITGKIRI

KCAVCPDFDLCVECMSVGAEITPHKRDHAYRVMGNLTFPLICPDWSADDEMLLLEGLEIY

GLGNWAEVAEHVGTKSKEQCLEHYKNIYLNSPFFPLPDMSHVAGKNKKELQAMAKGRVED

KKAEQIMKEEYPFSPPKVKVEDTQKESHTDRSFGGKKPVVAPGNNSLVELSNYNLKRQEF

DPEYDNDAEQLLAEMEFKENDTPEEHELKLRVLRIYSKRLDERKRRKEFILERNLLYPNP

FEKDLSQEEKVQCRRLDVFMRFHSKEEHEELLRSVVSEYRMVKRLKDLKEAQGAGCRSTA

EAERYLGRKRKRENEEGMNRGKESGQFGQLAGEMGSRPPVQASSSYVNDLDLIGFTESQL

LSESEKRLCSEAKLVPPIYLQMQQVMSHEIFKGNVTKKSDAYSLFKIDPTKVDRVYDMLV

KKGIAQL

>CAA7046308.1 unnamed protein product [Microthlaspi erraticum]

MGRSRGNFHNFEDPTQRTRKKKNAANVENFESTSMVPGTEGGGKYNCDYCQKDITGKIRI

KCAVCPDFDLCVECMSVGAEITPHKCDHAYRVMGNLTFPLICPDWSADDEMLLLEGLEIY

GLGNWAEVAEHVGTKSKEQCLEHYKNIYLNSPFFPLPDMSHVAGKNKKELQAMAKGRVED

KKEQNMKEEYPFSPPKVKVEDTQKESHTDRSFGGKKPVAPGNNTLVELSNYNHKRQEFDP

EYDNDAEQLLAEMEFKENDTPEEHELKLRVLRIYSKRLDERKRRKEFILERNLLYPNPFE

KDLSQEEKVQCRRLDVFMRFHSKEEHDELLRSVVNEYRMMKRLKDLKEAQMAGCRSTAEA

ERYLGRKRKRENEEGMNRGKESGQFGQIAGEMGSRPPVQASSSYVNDLDLIGFSESQLLS

ESEKRLCSEVKLVPPVYLQMQQVMSHEIFKGNVTKKSDAYSLFKMDPTKVDRVYDMLVKK

GIAQL

>BAJ34320.1 unnamed protein product [Eutrema halophilum]

MGRSRGNFHNFEDPTQRTRKKKNAANVENFESTSMVPGTEGGGKYNCDYCQKDITGKIRI

KCAVCPDFDLCVECMSVGAEITPHKRDHAYRVMGNLTFPLICPDWSADDEMLLLEGLEIY

GLGNWAEVAEHVGTKSKEQCLEHYKNIYLNSPFFPLPDMSHVAGKNKKELQAMAKGRVED

KKAEQIMKEEYPFSPPKVKVEDTQKESHTDRSFGGKKPVVAPGNNSLVELSNYNLKRQEF

DPEYDNDAEQLLAEMEFKENDTPEEHELKLRVLRIYSKRLDERKRRKEFILERNLLYPNP

FEKDLSQEEKVLCRRLDVFMRFHSKEEHEELLRSVVSEYRMVKRLKDLKEAQGAGCRSTA

EAERYLGRKRKRENEEGMNRGKESGQFGQLAGEMGSRPPVQASSSYVNDLDLIGFTESQL

LSESEKRLCSEAKLVPPIYLQMQQVMSHEIFKGNVTKKSDAYSLFKIDPTKVDRVYDMLV

KKGIAQL

>KFK26542.1 hypothetical protein AALP_AA8G262500 [Arabis alpina]

MGRSRGNFNNFEDPTQRTRKKKNAANVENFESTSMVTGAEGGGKYNCDYCQKDITGKIRI

KCAVCPDFDLCVECMSVGAEITPHKSDHPYRVMGNLTFPLICPDWSADDEMLLLEGLEIY

GLGNWAEVAEHVGTKTKEQCLDHYRNIYLNSPFFPLPDMSHVAGKNRKELQAMAKGRTEE

KKADQNMKEEYPFSPPKVKVEDTQKESHTDRTFGVKKPVAPGNNSLVELSSYNHKRQEFD

PEYDNDAEQLLAEMEFKENDTPEEHELKLRVLRIYSKRLDERKRRKEFILERNLLYPNPY

EKDLSQEEKVQCRRLDVFMRFHSKEEHDELLRSVVSEYRMVKRLKDLKEAQVAGCRSTAE

AERYLGRKRKRENEEGINRGKESGQFGQISGEMGSRPPVQASSSYVNDLDLIGFTESQLL

SESEKRLCSEAKLVPPVYLQMQQVMSHEIFKGNVTKKSDAYSLFKIDPIKVDRVYDMLVK

KGIAQL

>VVA93431.1 unnamed protein product [Arabis nemorensis]

MGRSRGNFNNFEDPTQRTRKKKNAANVENFESTSMVTGAEGGGKYNCDYCQKDITGKIRI

KCAVCPDFDLCVECMSVGAEITPHKSDHPYRVMGNLTFPLICPDWSADDEMLLLEGLEIY

GLGNWAEVAEHVGTKSKEQCLDHYRNIYLNSPFFPLPDMSHVAGKNRKELQAMAKGRIEE

KKAEQNMKEEYPFSPPKVKVEDTQKESHTDRSFGVKKPVAPGNNSLVELSNYNHKRQEFD

PEYDNDAEQLLAEMEFKENDTPEEHELKLRVLRIYSKRLDERKRRKEFILERNLLYPNPF

EKDLSQEEKVQCRRLDVFMRFHSKEEHDELLRSVVSEYRMVKRLKDLKEAQVAGCRSTAE

AERFLGXXXXXXXXXXXXXXXXSGQFGQIAGEMGSRPPVQASSSYVNDLDLIGFTESQLL

SESEKRLCSEAKLVPPVYLQMQQVMSHEIFKGNVTKKSDAYSLFKIDPTKVDRVYDMLVK

KGIAQL

>CDY69435.1 BnaA08g30200D [Brassica napus]

MGRSRGNFHNFEDPTQRTRKKKNAANVENFESSSMVTGTEGGGKYNCDYCQKDITGKIRI

KCDVCPDFDLCVECMSVGAEITPHKCDHAYRVMGNLTFPLICPDWSADDEMLLLEGLEIY

GMGNWAEVAEHVGTKSKEQCLEHYRNIYLNSPFFPLPDMSHVAGKNKKELQAMAKGRIEE

KKAEQNMKEEYPFSPPKVKVEDTQKESHTDRSFGGKKPVVAPGNNSLVELSNYNHKREEF

DPEYDNDAEQLLAEMEFKDNDTPEEKDLKLRVLRIYSKRLDERKRRKDFILDRNLLYPNP

FEKELSQEEKMQCRRLDVFMRFHSKEEHAELLRSVVSEYRMVKRLKDLKEAQMAGCRSTA

EAERYLARKRKRENEEGMMNRGKESGQFGAGEMGTRPPVQASSSYVNDLDLIGFTESQLL

SESEKRLCSEAKLVPPVYLHMQQVMSHEIFKGNVTKKSDAYSLFKIDPTKVDRVYDMLVK

KGIAQL

>XP_013604354.1 PREDICTED: transcriptional adapter ADA2b isoform X1 [Brassica oleracea var. oleracea]

MGRSRGNFHNFEDPTQRTRKKKNAANVENFESSSMVTGTEGGGKYNCDYCQKDITGKIRI

KCDVCPDFDLCVECMSVGAEITPHKCDHAYRVMGNLTFPLICPDWSADDEMLLLEGLEIY

GMGNWAEVAEHVGTKSKEQCLEHYRNIYLNSPFFPLPDMSHVAGKNKKELQAMAKGRIEE

KKAEQNMKEEYPFSPPKVKVEDTQKESHTDRSFGGKKPVVAPGNNSLVELSNYNHKREEF

DPEYDNDAEQLLAEMEFKDNDTPEEKDLKLRVLRIYSKRLDERKRRKEFILDRNLLYPNP

FEKELSQEEKMQCRRLDVFMRFHSKEEHEELLRSVVSEYRMVKRLKDLKEAQMAGCRSTA

EAERYLARKRKRENEEGMMNRGKESGQFGAGELGTRPPVQASSSYVNDLDLIGFTESQLL

SESEKRLCSEAKLVPPVYLHMQQVMSHEIFKGNVTKKSDAYSLFKIDPTKVDRVYDMLVK

KGIAQL

>XP_009124520.1 transcriptional adapter ADA2b isoform X1 [Brassica rapa]

MGRSRGNFHNFEDPTQRTRKKKNAANVENFESSSMVTGTEGGGKYNCDYCQKDITGKIRI

KCDVCPDFDLCVECMSVGAEITPHKCDHAYRVMGNLTFPLICPDWSADDEMLLLEGLEIY

GMGNWAEVAEHVGTKSKEQCLEHYRNIYLNSPFFPLPDMSHVAGKNKKELQAMAKGRIEE

KKAEQNMKEEYPFSPLKVKVEDTQKESHTDRSFGGKKPVVAPGNNSLVELSNYNHKREEF

DPEYDNDAEQLLAEMEFKDNDTPEEKDLKLRVLRIYSKRLDERKRRKEFILDRNLLYPNP

FEKELSQEEKMQCRRLDVFMRFHSKEEHAELLRSVVSEYRMVKRLKDLKEAQMAGCRSTA

EAERYLARKRKRENEEGMMNRGKESGQFGAGEMGTRPPVQASSSYVNDLDLIGFTESQLL

SESEKRLCSEAKLVPPVYLHMQQVMSHEIFKGNVTKKSDAYSLFKIDPTKVDRVYDMLVK

KGIAQL

>XP_018451532.1 PREDICTED: transcriptional adapter ADA2b isoform X1 [Raphanus sativus]

MGRSRGNFQNFEDPTQRTRKKKNAANVENFESSSTVTGTEGGRKYNCDYCQKDITGKIRI

KCAVCPDFDLCVECMSIGAQITPHKCEHTYSVMGDLNFELTCPGWSADDEMLLLEGLEIY

GMGNWAEVAEHVGTKSKEQCLQHYTNYYLNSPFFPLPDMSHVAGKNMKELQAMAKGRVVE

EKKAEQKMKEEYPFSPPKVKVEDTQKESHTDRSFGGKKPVAPPGNNSLVELSNYNHKREE

FDPEYDNDAEQLLAEMEFKDNDTPEEKDLKLRVLRIYSKRLDERKRRKEFILERNLLYPN

PFEKDLSQEEKMQCRRLDVFMRFHSKEEHEELLRSVVSEYRMVKRLKELKEAQMAGCRST

AEAERYLARKRKRENEEGMNRGKESGGEMGTRPPVQASSSYVNDLDLIGFTESQLLSESE

KRLCSEAKLVPPVYLHMQQVMSHEIFKGNVTKKSDAYSLFKIDPTKVDRVYDMLVKKGIA

QL

>XP_010540843.1 PREDICTED: transcriptional adapter ADA2b isoform X2 [Tarenaya hassleriana]

MGRSRGNFHNYEDPTQRTRKKKNAANVENFESTSTVQGTEGGGKYNCDYCQKDITGRIRI

KCAVCPDFDLCVECMSVGAEVTPHKSDHPYRVMGNLTFPLICPDWSADDEMLLLEGLEIY

GLGNWAEVAEHVGTKSKELCLEHYRNIYLNSPFFPLPDMSHVAGKNRKELQAMAKGRIED

KKVEPNMKEEYPFSPSKIKVEETPKESQADRSFGGKKPIASGNNSLVELSNYNQKRQEFD

PEYDNDAEQLLAEMEFKETDTPEEHELKLRVLRIYSKRLDERKRRKEFILERNLLYQNPF

EKDLSPEEKALCRRFDVFMRFHSKEEHDEMLRTIVNEYRMLKRLKDLKDAQAAGCRSTAE

AERYLERKRKREIEERAKESGQFGGNLNDTGSRPCVQASSSYVNDFDLIGFSESQLLSES

EKRLCCETKLVPPVYLHMLQIMSQEIFKGNVTKKSDAYSLFKIDSSKVDRVYDMLVKKGI

AQP

>XP_021902078.1 transcriptional adapter ADA2b isoform X1 [Carica papaya]

MGRSRGNFHSDEDPTQRSRRKKNAPSGENSDSAFAGQGSSEGKRALYHCNYCNKDITGKI

RVKCAVCPDFDLCVECFSVGAEVTPHKCNHPYKVMDNLSFPLICPDWNADEEMLLLEGIE

MYGLGNWAEVAEHVGTKNKESCIEHYTSVYLNSPYFPLPDMSHVVGKNRKELLAMAKGHS

EDKKGALMLGELNIKEERSFSPSTVKVEEMHRTGPSNRLLSGGKAELDSGTHSGSKSMEA

AADMAQVKDDSSIIKMEDPQTDRNFGGKKPNSLGNDNLLELSGYNPKRQEFDPEYDNDAE

QLLAEMEFKDTDTNEERELKLRVLHIYSRRLDERKRRKDFILERNLLYTNPFEKDLSPEE

RAICRSYDAFMRFHSKEEHEELLRTAIQEHRTLKRLQELKEARAAGCRTSAEADRYLEQR

RKRQSEESSRRAKESTQVGTSIQGGPNAFMASEPVSKDSNCRPSGQTSSSSLNDLDILGF

SETQLLSESEKRLCREIRLPPPLYLRMQQVISEEIFRGNVKKKSDAHSLFKMEHSIVDRV

YDILVKKGIAQP

>XP_010551961.1 PREDICTED: transcriptional adapter ADA2a isoform X4 [Tarenaya hassleriana]

MGRSRIVSRPADEDANPGKSKRKKTSPGPENAGTAISTVLNERKAGLYHCNYCNKDLSGL

IRFKCAVCTDFDLCVECFSVGAELNPHKSNHPYRVMDNLSFPFVTPDWNADEELLLLEAI

STYGFGNWQEIADNVGSKTKSECSDHYHSAYMNSPCFPFPDLSHAIGKSKEELIALGKEL

TIKKEVPVLMEPSPKEELPMPAEIKDEASGKEDVIDQSLPNLAGIKKKVNGLQAKESIKS

EVADQQPDRSVGEKKPRLAGDQALSVTESYGYNFKRQEFDVEYDNDAEQLLADMDFKESD

TDAEHDLKLQVLHVYSKRLDERKRRKNFILERNLLYPDTFEMSLSPEERQIYKRYKVFAR

FHSKEEHEELLKTIIEEHRILRRIQDLQEARTAGCRTASEANRYIEEKRKKEAEETGQRL

MQGVPGSVAGKALKSPRGLPKSLQPFGAETLSKVTGPIISSSLDNWDVNGLLGADLLSET

EKKMCNEIRILPAHYLKMLQTLSMEIKNGHIKQKSDAYTLFKVDPYKVDKVYDMLVQKGI

AQS

>KFK38232.1 hypothetical protein AALP_AA3G086100 [Arabis alpina]

MGRSKLAARPAEEDANPGKSKRKKLSLGPENAVASVSIGGEVGNERKPGLYCCNYCDKDL

SGLVRLKCAVCLDFDLCVECFSVGVELNRHKSSHPYRVMDNLSFPLVTSDWNADEEILLL

EAIATYGFGNWKEVADHVGSKTKAECIDHFNSAYMQSHCFPLPDLSHTLGKSKEELFAMC

KEHAVKTEIPAIVKLSPKEELPMSAEIKHKTSGKDDAIDQPLPGLAGVKKKVNVPQAKDN

IKLEAAKQQSDRSVGEKKPRLPGEKVPLVTELYGYNLKREEFEIEHDNDAEQLLADMEFL

ESDTDAEREQKLQVLHIYSKRLDDRKRKKEFVLERNLLYPDEFEKSLSAEERKLYNSCKV

FARFHSKEEHKELVKNVIEEHQILRRIQDLEEARTAGCRTTSEANRFIEDKRKKEAEESM

LRLNQGAPGSIAGKSLKSPRGLARNLQPFGSTSLSKVTLPMPSSLDNWDVSGLLGADLLS

ETEKKMCNEIRILPAHYFKMLETLTSEIKKGQIKKKSDAYSFFKVEPSKVDKVYDLLIQK

GIGEST

>XP_006297371.1 transcriptional adapter ADA2a [Capsella rubella]

MGRSKLASRPADADLNPGKSKRKKISSGSDNAGASVPTGGEAGNERKAGLYCCNYCDKDL

SGLVRFKCAVCMDFDLCVECFSVGVELNRHKNSHPYRVMDNLSFPLVTSDWNADEEILLL

EAIATYGFGNWKEVADHVGSKTKTECIDHFNSAYMQSPCFPLPDLSRTFGKSKEELLAMS

KEHAVKTEIPALVRLSPKEELPMPAEIRHKDLGKEDAIDQPLSALAGVKKKGNVPQAKDI

TKLEAAKQQSDRSVGEKKLRLPGEKVPLVTELYGYNLKREEFEIEHDNDAEQLLADMEFK

DSDTDAEREQKLQVLHIYSKRLDERKRRKEFVLERNLLYPDQYEMSLSAEEKKIYKSCKV

FARFHSKEEHEELIKKVIEEHQILRRIQDLQEAKTAGCKTSSEANRFIEEKRKKEAEDSI

VLRLNHGAPGSIAGKTLKSPRGLPRNLQPFGPDPLSKVTPPTIYSGLDNWDVNGLLGADL

LSETEIKMCNETRILPVHYLKMLEILTSEIKKGHINKKSDAYSFFKVEPSKVDRVYDMLI

QKGIGESS

>CAA7050967.1 unnamed protein product [Microthlaspi erraticum]

MGRSKLAARPAEDDANPGKSKRKKLSSGSENAVASISTGGEAGNERKAGLYCCNYCDKDL

SGLVRFKCAVCLDFDLCVECFSVGVELSRHKSSHPYRVMDNLSFPLVTSDWNADEEILLL

EAIATYGFGNWKEVADHVGSKTMTECIDHFNSAYMQSPCFPLPDLSHTIGKSKDELLAMS

EEHAVKTEIPASVKLSPKEESPMSAEIKHEASGKDNAIDQPLPGLAGVKKKVVAPQATDS

IKLEAAKQQSDRSVGEKKPRLPGEKVPLATELYGYNLKRQEFEIEHDNDAEQLLADMEFK

DTDTDAEREQKLQVLHIYSKRLDERKRRKEFVLDRNLLYPDQFELSLSAEERQIYNKCKV

FARFHSKEEHKELIEKVIEEHRILKRIQDLQEAKAGGCRTTSEATRFIEEKRKKEAEESL

LRQNQGAPGSIASKTLKSPRGSARSLQPFGSDSLSKVTLPVISSSLDNWDVSGLLGADLL

SETEKKMCNEIRLLPAHYLKMLETLTREMKKGQIKKKSDAYSFFKVEPSKVDKVYDLLIQ

KGIGVSPHDS

>XP_002884665.1 transcriptional adapter ADA2a isoform X2 [Arabidopsis lyrata subsp. lyrata]

MGRSKLASRPAEEDLNTGKSKRKKISSGPENAGASVSTGSEAGNERKAGFYCCNYCDKDL

SGLVRFKCAVCMDFDLCVECFSVGVELNRHKNSHPYRVMDNLSFPLVTSDWNADEEILLL

EAIATYGFGNWKEVADHVGSKTNTECIDHFNSAYMQSPCFPLPDLSHTIGKSKEELLAMS

KESAVRTELPALVRLSPKEELPMSAEIKHEASGKDNAIDPPLPALAGVKKKVNVPQAKDI

KLEAAKQQSDRSVGEKKLRLPGEKVPLVTELYGYNLKREEFEIEHDNDAEQLLADMEFKD

SDTDAEREQKLQVLHIYSKRLDERKRRKEFVLERNLLYPDQYEMSLSAEERKIYKSCKVF

ARFHSKEEHKELIKKVIEEHQILRRIQDLQEARTAGCRTTSEANRFIEEKRKKEAEESVL

LRLNHGAPGSIAGKTLKSPRGLPRNLQPFGSDSLPKVTPPIIYSGLDTWDVDGLLGADLL

SETEKKMCNETRILPVHYLKMLDILTSEIMKGQIKKKSDAYSFFKVEPSKVDRVYDMLVQ

KEIGEST

>XP_033148224.1 transcriptional adapter ADA2a isoform X1 [Brassica rapa]

MGRSKLASRPATEDANPGKAKRKKLSSGTENAPGPSISIGGEAGNERKPGLYCCNYCDKD

LSGLVRLKCAVCADFDLCVECFYVGVELNRHKSSHPYRVMDNLSFPLVSSDWNADEEILL

LEAIATYGFGNWKEVANYVGSKTQTECVDHFNSAYMQSPCFPLPDLSHTNGKSKDELLAM

SKEHAVKKGLLDESPALVNLSPKEELPMSVEIKEEASGKEDSVDQPLPILAGVKKKANAP

PQTQDTIKLEAAKQLSERSVGEKKPRLPGEKVPFVTELYGYNLKRQEFEIEHDNDAEQLL

SDMEFKDCDTDAEREQKLQVLHIYSKRLDERKRRKEFVLERNLLYPDQFELSLSAEEKQI

YNKCKVFARFHSKEEHKELIQKVIEEHRILKRIQDLQEARAAGCTTTTEANRFIEEKRKK

EAEENLVRLNHGVPGSVAGKALKSPRGLQRNLQPFGSESLSKATLPIICSSLDNWDVSGL

LGADLLSETEKEMCNEMRILPAQYFKMLETLTSEIKKGTVKKKSDAYSFFRVEPSKIDRV

YDLLIQKGIGESS

>XP_006407803.2 transcriptional adapter ADA2a [Eutrema salsugineum]

MGRSKLASRPADEDANPGKSKRKKLSLDPENAGASVSTGGEARIERKPGLYCCNYCDKDL

SGFVRFKCAVCMDFDLCVECFSVGAELNRHKSSHPYRVMDNLSFPLVTSDWNADEEILLL

EAISTYGFGNWKEVADHVGSKTKTECIDHFNSAYMQSPCFPLPDLSHTNGKSKEELLAMS

KEHAVKNEIPALVKLSPKEELQMSAVIIHEDSGKDDAIDQPLPVLAGVKKKVNGPQATDS

IKLEAAKQPSDRSVGEKKPRLPGEKVPLVTELYGYNLKRQEFEIEHDNDAEQLLADMEFK

DSDTDAEREQKLQVLHIYSKRLDERKRRKEFVLERNLLYPDQFEMSLSAEERKLYSSCKV

FARFHSKEEHKELMKKVIEEHRILRRIQDLQDARAAGCRTSTDANRFIEEKRKKEAEESW

LRQNHGAPGSIASKTLKSPRGLPRNLQPFGSVSLSKVTLPIISSSLDDWDVSGLLGADLL

SETEKSMCNEMRILPAHYFKMLETLTSEIKKGNIKKKSDAYSFFKVEPSKVDKVYDLLIQ

KGIGEST

>XP_010457040.1 PREDICTED: transcriptional adapter ADA2a-like [Camelina sativa]

MGRSKLASRPAADADLNPGKSKRKKISTGSDNAGASVPTGGEAGNERKAGLYCCNYCDKD

LSGLVRFKCAVCMDFDLCVECFSVGVELSRHKNSHPYRVMDNLSFPLVTSDWNADEEILL

LEAIATYGFGNWKEVADHVGSKTKIECRDHFNSAYMQSPFFPLPDLSHTIGKGKEELLAM

SKEHDVKTEIPALARLSPKEELPMLAEIKHKASGKEDAIDQPLTALAGVKKKVNVPQAKD

ITKLEAAKQQSDRSVGEKKLRLPGEKVPLVTELYGYNLKREEFEIEHDNDAEQLLADMEF

KDSDTDAEREQKLQVLHIYSKRLDERKRRKEFVLERNLLHPDQYEMGLSAEEKKIYKSCK

VFARFHSKEEHKELIKEVIEEHQILRRIQDLQEARTAGCKTASEAKRFIEEKRKKEAEES

LLLRLNHGAPGSIAGKTLKSPRGLPRNLQPFGSDSLSKITPPIIYSGLDNWDVDGLLGAD

LLSEIEKKMCNETRILPVHYLKMLEILTSEIKKGQIKKKSDAYSFFKVEPSKVDRVYDML

IQKGIGGEST

>NP_566317.1 ADA2 2A [Arabidopsis thaliana]

MGRSKLASRPAEEDLNPGKSKRKKISLGPENAAASISTGIEAGNERKPGLYCCNYCDKDL

SGLVRFKCAVCMDFDLCVECFSVGVELNRHKNSHPYRVMDNLSFSLVTSDWNADEEILLL

EAIATYGFGNWKEVADHVGSKTTTECIKHFNSAYMQSPCFPLPDLSHTIGKSKDELLAMS

KDSAVKTEIPAFVRLSPKEELPVSAEIKHEASGKVNEIDPPLSALAGVKKKGNVPQAKDI

IKLEAAKQQSDRSVGEKKLRLPGEKVPLVTELYGYNLKREEFEIEHDNDAEQLLADMEFK

DSDTDAEREQKLQVLRIYSKRLDERKRRKEFVLERNLLYPDQYEMSLSAEERKIYKSCKV

FARFQSKEEHKELIKKVIEEHQILRRIEDLQEARTAGCRTTSDANRFIEEKRKKEAEESM

LLRLNHGAPGSIAGKTLKSPRGLPRNLHPFGSDSLPKVTPPRIYSGLDTWDVDGLLGADL

LSETEKKMCNETRILPVHYLKMLDILTREIKKGQIKKKSDAYSFFKVEPSKVDRVYDMLV

HKGIGDST

>XP_013697995.1 transcriptional adapter ADA2a isoform X1 [Brassica napus]

MGRSKLASRPAPEDANPGKSKRKKLSSGTENAPGPSVSIGGEAGNERKPGLYCCNYCDKD

LSGLVRLKCAVCVDFDLCVECFYVGVELNRHKSSHPYRVMDNLSFPLVSSDWNADEEILL

LEAIATYGFGNWKEVANYVGSKTLTECVDHFNSAYMQSPCFPLPDLSHTNGKSKDELLAM

SKDHAVKKGLLDETPPLVNLSPKEELPMSVEINEEASGKEDSVDQPLPILAGVKKKANAP

QTQDTIKLEAAKQLSERSVGEKKPRLPGKKVPFVTELYAYNLKRQEFEIEHDNDAEQLLS

DMEFKDCDTDAEREQKLQVLHIYSKRLDERKRRKEFVLERNLLYPDQFELSLSAEERQIY

NKCKVFARFHSKEEHKELIQKVIEEHRILKRIQDLQEARAAGCTTTTEANRFIEEKRKKE

AEENLVRLNHGGPGSVAGKGLKSPRGLQRNLQPFGSESLSKATLPIICSSLDNWDVSGLL

GADLLSETEKEMCNEMRILPAHYFKMLETLASEIKKGTVKKKSDAYNFFRVEPSKVDRVY

DLLIQKGIGESS

>KAF3605343.1 hypothetical protein DY000_02051189 [Brassica cretica]

MGRSKLASRPAPEDANPGKSKRKKLSSGTENAPGPSVSIGGEAGNERKPGLYCCNYCDKD

LSGLVRLKCAVCVDFDLCVECFYVGVELNRHKSSHPYRVMDNLSFPLVSSDWNADEEILL

LEAIATYGFGNWKEVANYVGSKTQTECVDHFNSAYMQSPCFPLPDLSHTNGKSKDELLAM

SKEHAVKKETPPPLVNLSPKEELPISVEIKEEASGKDDSVDQPLPVLAGVKKKANAPQTQ

DTIKLEAAKQLSERSVGEKKPRLPGEKVPFVTELYGYNLKRQEFEIEHDNDAEQLLSDME

FKDCDTDAEREQKLQVLHIYSKRLDERKRRKEFVLERNLLYPDQFELSLSAEEKQIYNKC

KVFARFHSKEEHKELIQKVIEEQRILKRIQDLQEARAAGCTTTTEANRFIEEKRKKEAEE

NLVRVNHGGPGSVAGKALKSPRGLQRNLQPFGSESLSKATLPIICSSLDNWDVSGLLGAD

LLSETEKEMCNVMRILPAHYFKMLETLTREIKKGTVKKKSDAYSFFRVEPSKVDRVYDLL

IQKGIGESS

>XP_013585313.1 PREDICTED: transcriptional adapter ADA2a [Brassica oleracea var. oleracea]

MGRSKLASRPAPEDANPGKSKRKKLSSGTENAPGPSVSIGGEAGNERKPGLYCCNYCDKD

LSGLVRLKCAVCVDFDLCVECFYVGVELNRHKSSHPYRVMDNLSFPLVSSDWNADEEILL

LEAIATYGFGNWKEVANYVGSKTLTECVDHFNSAYMQSPCFPLPDLSHTNGKSKDELLAM

SKDHAVKKETPPLVNLSPKEELPMSVEINEEASGKEDSVDQPLPILAGVKKKANAPQTQD

TIKLEAAKQLSERSVGEKKPRLPGKKVPFVTELYAYNLKRQEFEIEHDNDAEQLLSDMEF

KDCDTDAEREQKLQVLHIYSKRLDERKRRKEFVLERNLLYPDQFELSLSAEERQIYNKCK

VFARFHSKEEHKELIQKVIEEHRILKRIQDLQEARAAGCTTTTEANRFIEEKRKKEAEEN

LVRLNHGGPGSVAGKGLKSPRGLQRNLQPFGSESLSKATLPIICSSLDNWDVSGLLGADL

LSETEKEMCNEMRILPAHYFKMLETLASEIKKGTVKKKSDAYNFFRVEPSKVDRVYDLLI

QKGIGESS

>VVA96852.1 unnamed protein product [Arabis nemorensis]

MGRSKLASRPAEEDANPGKSKRKKLSLGPENAVASISIGGEVGNERKPGLYCCNYCDKDL

SGLVRFKCAVCLDFDLCVECFSVGVELNRHKSSHPYRVMDNLSFPLVSSDWNADEEILLL

EAIATYGFGNWKEVSDHVGSKTKAECIDHFNSAYMQSPCFPLPDLSQTIGKSKEELFAMS

KEHAVKTESPALVKLSPKEELLMSAEIKHEASGKDDTIDQPLPGLAGVKKKVNVPQAKDN

IKLEAAKQQSDRSVGEKKPRLPGEKVPLVTELYGYNLKRQEFEIEHDNDAEQLLADMEFL

DSDTDAEREQKLQVLHIYSKRLDERKLRKEFVLERNLLYPDQFEMSLSAEERKIYNECKV

FARFHSKEEHKELIEKVIEEHRILRRIQDLQEARTAGCRTTSEAKRFXXXXXXXXXXXXM

LRLNHGAPGSTAGKSLKSPRGLARNLQPFGSASLSKVTLPISSSLANWDVSGLLGADLLS

ETEKKMCNEIRILPVHYFKMLETLTSEIKKGQIKKKSDAYSFFKVEPSKVDKVYDLLIQK

GIGEST

>XP_018492581.1 PREDICTED: transcriptional adapter ADA2a-like [Raphanus sativus]

MGRSKLASRPATEDANPGKSKRKKLSSGTENAPGPSVSIGGEGGNERKPGLYCCNYCDKD

LSGLVRLKCAVCVDFDLCVECFSVGVELSRHKSSHPYRVMDNLSFPLVSSDWNADEEILL

LEAIATYGFGNWKEVANYVGSKTQSECIDHFNSAYMQSPCFPLPDLSRTNGKSKDELLAM

SKEHVVKKESPALVMNLSPKEELPMSAEIKDEASGKDDPIDQPLPVLAGVKKKANAPQTQ

DIVKLEAAKQPSERSVGEKKPRLPGEKVPFVTELYGYNLKRQEFEIEHDNDAEQLLSDME

FKDCDTDAEREQKLQVLHIYSKRLDERKRRKEFVLERNLLYPDQFELSLSAEERQIYNKC

KVFARFHSKEEHKELIQKVIEERRILRRIQDLQEARAAGCTTTTEANRFIEEKRKKEAEE

NLVRLNHGGPGSTVAGKALKSPRGLHRNLQPFGSDSLSKTTLPIICSSLDNWDVSGLLGA

DLLSETEKKMCNEMRILPAHYFKMLETLTSEMKKGKIKKKSDAYSFFRVEPSKVDRVYDL

LIQKGVGESS

>XP_030514080.1 transcriptional adapter ADA2b isoform X1 [Rhodamnia argentea]

MGRSRGNFHSNDEDPTQRSRRKKNATSGENLDSSSAGQGSSDGKKALYHCNYCNKDITGR

IRIKCAMCPDFDLCIECFSVGAEVVPHKSSHQYRVMDNLSFPLICPDWNADDEILLLEGI

EMYGLGNWTEVAEHVGTKSKEQCIDHYREVYMNSPVFPLPDMSHVVGKNRKELLAMAKGH

GEDKKGPSVGDLSLKEESPFSPPRAKVEDPHKGGSSGRLLSGANADMDTGSCSSSNNAAS

GAGIKAYNMAQAKNGVSNIKVEDAQFNSNFNGMTPSTLGDDGPSLIESSGYNPKRQEFDT

EYDNDAEQLLAEMEFKETDTEEENELKLRVLRIYSKRLDERKRRKDFILERNLLFQSTFE

KSLTPEERALCRRYDVFMRFHSKEEHEDLLQTIVAEHRTLKRIKELQEARAAGCRTSAEA

DRYFENKRRREAEESSRRGKDSSQLGASGQGPSIHASSGLANDLDVMGFNGSDLLSDHEK

LLCSELRLPPPVYLKMQQVMSMEIFSGNVTKKSDAHHLFQMEPGKVDRVYDMLVRKGLAP

P

>KCW75135.1 hypothetical protein EUGRSUZ_E03886 [Eucalyptus grandis]

MGPSRGNFHSNDEDPTQRSRRKKNASTGENLDSSSAGQGSSDGKKAAYHCNYCNKDITGR

IRIKCAMCPDFDLCIECFSVGAEVVPHKSGHPYRVMDNLSFPLICPDWNADDEILLLEGI

EMYGLGNWAEVAEHVGTKSKELCIDHYYEVYMNSPVFPLPDMSHVEGKNRKELLAMAKGH

GEDKKGPSVGDLSLKEESPFSPPRVKLEDPHKGGSSGRLLSGANADMDAGSCSSSNNAAS

GAGIKGSNVAQAKNGVSNIKAEDAQFNGNFNGMTPSTLGDDGPSLIESSGYNPKRQEFDT

EYDNDAEQLLAEMEFKETDTEEEHELKLRVLRIYSKRLDERKRRKDFILERNLLFQSPFE

KSLMPEERSLCRRYDVFMRFHSKEEHEDLLQTIVAEHRTLKRIKELQEARAAGCRTSAEA

DRYFENKRRREAEESTRRGKDSSQLGATGQGPPSIHASSGLASDLDVMGFNGSDLLSDTE

KRLCSELRLPAPVYLKMQQVMSVEIFSGNVTKKSDAHNLFQMEPSKVDRVYDMLVRKGLA

PP

>XP_030471165.1 transcriptional adapter ADA2b isoform X1 [Syzygium oleosum]

MGRSRGNFHSNDEDPTQRSRRKKNASSGENLDSSSAGQGSSDGKKALYHCNYCNKDITGR

IRIKCAMCPDFDLCIECFSVGAEMAPHKSSHPYRVMDNLSFPLICPDWNADDEILLLEGI

EMYGLGNWAEVAEHVGTKSKEQCIDHYNEVYMNSPVFPLPDMSHVVGKNRKELLAMAKGH

GEDKKGPSVGDLSLKEESPFSPPRAKVEDPHKGGSSGRLLSGTNADMDTGSCSSSNNAAS

GAGIKASNKTQAKNGVSNIKVEDAQFNSNFNGMTPSTLGDDGPSLIESSGYNPKRQEFDT

EYDNDAEQLLAEMEFKDTDTEEEHELKKRVLRIYSKRLDERKRRKEFILERNLLYQSPFE

KSLTPEERALCRRYDVFMRFHSKEEHEDLLQTIVAEHRTLKRIKELQEARAAGCCTSAEA

DRYFENKRRREAEESSRRGKDSSQLGAGGQGPSSMHASSGLPNDLDVMGFNGSELLSDNE

KRLCSELRLPAPVYLKMQQVMSMEIFSGNVTKKSDAHHLFQMEPSKVDRVYDMLVRKGLA

PP

>XP_031393500.1 transcriptional adapter ADA2b [Punica granatum]

MGRSRGNFHSADEDPTQRSRRKKNPSSGENSESASIGQGSSEGKRALYHCNYCNKDITGR

IRIKCAVCPDFDLCVECFSVGAEVTPHRSNHAYRVMDNLSFPLICPDWNADDEILLLEGI

EMYGLGNWMEVAEHVGTKNKEQCIDHYTEVYMNSPFFPLPDMTHVVGKNRSELLAMAKGQ

GEDKKGVSSLGDLTLKEESPFSPSRVKVEDPHKGGPSGRFVSGIGADMDSRPCSSSTNSA

APMKKASNMGQVKDGMVKMEDAQGERSFKGKSPSTAGNNGPSLLEMSSYNPKRQEFDIEY

DNDAEQLLAEMEFKDTDTEDERELKLRILRIYAKRLDERKRRKDFILERNLLYPNPFEKD

LTPEERAICRHYDVFMRFHTKEEHNDLLETVISEHRILKRIQLLQDARTAGCRTPAEADA

YFEHRRREAEESAHRAKDGSQLGPSSHGAFMGSSDPRAAGQGSSSSANIVETMGYSGADL

LSENEKRLCTEIRVPPQVYLSMQQVMSVEIFKGKVTKKMDAYHLFQMDPSKIDRVYDMLV

RKGIAAP

>XP_010045771.1 PREDICTED: transcriptional adapter ADA2a [Eucalyptus grandis]

MGRSRAASRPADEDPNQRSKKKKIASGPENVDSASAVIGQGNEEKGASYHCNYCNKDISG

KIRIRCATCPDFDLCIECFSVGAEINPHKSNHPYRVMDNLSFPLLCPGWNADEEMLLLEG

IEMYGFGNWAEVADHIGSKSKSQCIAHYNAMYMNSPCWPLPDLSHIMGKNREELLAIAKG

QEELKEESSSVGEPLKEESASSTKFKSMESLKEIPVHAPSSGSIAGKRGCNISHAKEEIK

DEESQINRSIGEKKPRISGDEGPSATEFSGYNFKRQEFEVEYDNDAEQLLADMEFRDTDT

DAERELKLQVLRIYSKRLDERKRRKDFILERNLLYPDPFEKNLSAEEREIYKHFKVFTRF

HSKEEHEEFVKSIIEEHRIVKRIHELQDARAAGCRTAAEANKFIERKREKEAEGAQRMKE

ISQAGPSGKIFPKSSHVKVEPESSPQGLVRSSSSLQSSGKDATLTSLDTLHSSLDDWDIS

DFPGADLLSETEKRLCGEIRILPAHYLKMLHVMSIEMLRGNLTKKSDAHGLFRVEPSKVD

RVYDMLLSKGIAQA

>XP_030539748.1 transcriptional adapter ADA2 [Rhodamnia argentea]

MGRSRAASRSADEDPNQRSKRKKIGSGPENVESAYAVVGQGNEEKGASYHCNYCNKDMSG

KIRMKCATCPDFDLCIECFSVGAESTPHKSNHPYRVMDNLSFPLLCPGWNADEEMLLLEG

IEMYGFGNWAEVAEHVGSKSKSQCITHYNAMYMNSPCWPLPDLSHVMGKNREELVAMAKG

QEELKEESSSIGEPLKEESASSTKFKSMESLKEIPVHVLSSGSIAGKRGCNISHAKEEIK

DEESQIDRSIGEKKPRISGDEGPTATEFSGYNFKRQEFEVEYDNDAEQLLADMEFRDTDT

HAERELKLQVLHIYSKRLDERKHRKDFILERNLLYPDPFEKNLSPEEREIYKRFKVFTRF

HSKEEHEEFVKSIIEEHRIVKRIHELQDARAAGCRTAAEANKFIERKREKEAEGAQRMKE

ISQAGPSGKIFPKPSHVKVEQESSPRGLVRSSSSLQSSGKDTTLTSLDTLHSSLDDWDIS

DFPGVELLSETEKRLCGEIRILPAHYLKMLHVMSIEMLRGNLTKKSDAHGLFRVEPSKVD

RVYDMLLSKGIAQA

>XP_030460415.1 transcriptional adapter ADA2a-like isoform X1 [Syzygium oleosum]

MGRSRAASRPADEDPNQRSKRKKIGSGPENAESASTAIGQGNEEKGASYHCNCCDKDISG

KIRIKCAACPDFDLCIECFSVGAEIIPHKSNHPYRVMDNLCFPLLCPGWNADEEMLLLEG

IEMYGFGNWAEVAEHVGSKSKSQCITHYSAMYMNSPCWPLPDLSHVMGKNREELLAMAKG

QEELKEESSSIGEPLKEESASSTKFKSMESLKEIPVHVLSSGSIAGKRGCNISHTKEEIK

DEESQIDRSIGEKKPRISGDEGPSATEFSGYNFKRQEFEVEYDNDAEQLLADMEFRDTDT

DAERELKLQVLRIYSKRLNERKRRKDFILERNLLYPDPFEKNLSPEEKEIYKRFKVFTRF

HSKEEHEEFVKSIIEEHRIVKRIHELQDARAAGCRTAAEANKFIERKREKEAEGARRMKE

ISQAGPSGKIFPKPSHVKVEQESSPQGLVRSFSSLQSSGKDTTLTSLDTLHSSLDGWDIS

DFPGADLLSEAEKRLCGEIRILPAHYLKMLHVMSIEMLRGNLTKNTDAHGLFRVEPSKVD

RVYDMFLSKGIAQA

>PKI53764.1 hypothetical protein CRG98_025845 [Punica granatum]

MGRSRTAFRTGDDDPNQRAKKKKVASGPENAEAEFTGINEGTAGLYHCNYCNKDISGKVR

IKCAACPDFDLCIECFSVGAEITPHKSNHPYRVMDNLSFPLICPDWNVDEEMLLLEAIEM

YGFGNWAEVADYVGSKSKSQCIDHYNAVYMNSPHFPLPDLTHVMGKNKEELIAMAKELAE

VKEDSSSSGELPPKEEPVFSTRAKSVESEKEVPVQQLSSSSTAGKRTSHMLLIKDEIKEE

GTLNGRIGEKKPRLTGDEGPSMSDLGGYNFKRQEFEIEYDNDAEQLLADMEFRDTDTDAE

RELKLQVLRIYSRKLDERKRRKDFILERNLLYPDHFEKNLPPEERGIYKRLKVFTRYHCK

EDHEELLKSVIQEHQILRRIQQLQEARAAGCRTAAEANKFIDDKREREAEESSQRAKESA

QAGPSSKVLPRLNYSKSEVDGIPMELLDWDISDFPGAELLSETEKRLCREIRLLPAHYLK

MLHVLSMEIKEGKLTKKSDAYSLFKVDPTKTDRVYDMLVSKRNCSS

>ESR63149.1 hypothetical protein CICLE_v10014921mg [Citrus clementina]

MGRSRGNFHANDEDPTQRSRRKKNVSSDNSDTAAPGQGAGEGKRALYHCNYCNKDITGKI

RIKCAVCPDFDLCIECFSVGVEVHPHKSNHPYRVMDNLSFPLICPDWNADDEILLLEGIE

MYGLGNWAEIAEHVGTKTKELCIEHYTNVYMNSPFFPLPDMSHVVGKNRKELLAMAKGHI

DDKKGPSKPGEATVKEESPFSPSRVKIEEMHKVGPSGRGLNAADPQTERSSKGKKPVTSG

NDGPSLVELSGYNSKRQEFDPEYDNDAEQLLAEMEFKDADSEEERDIKLRVLRIYSKRLD

ERKRRKDFILERNLLYPNPFEKDLSPEEREHCRRYDVFMRFHSKEDHEDLLQTVISEHRT

LKRIQDLKEARAAGCRTSAEADRYLELKRGREAEEASRRAKEGGHAGASSQGGANVFMAS

ESLGKDSNSRPSGQASSSHVNDLYIMGFNETQLLSEAEKRLCCEIRLAPPLYLRMQEVMS

REIFSGNVNNKADAHHLFKIEPSKIDRVYDMLVKKGLAPP

>GAY35394.1 hypothetical protein CUMW_016040 [Citrus unshiu]

MGRSRGNFHANDEDPTQRSRRKKNVSSDNSDTAAPGQGAGEGKRALYHCNYCNKDITGKI

RIKCAVCPDFDLCIECFSVGVEVHPHKSNHPYRVMDNLSFPLICPDWNADDEILLLEGIE

MYGLGNWAEIAEHVGTKTKELCIEHYTNVYMNSPFFPLPDMSHVVGKNRKELLAMAKGHI

DDKKGPSKPGEATVKEESPFSPSRVKIEEMHKVGPSGRGLNADPQTERSSKGKKPVTSGN

DGPSLVELSGYNSKRQEFDPEYDNDAEQLLAEMEFKDADSEEERDIKLRVLRIYSKRLDE

RKRRKDFILERNLLYPNPFEKDLSPEEREHCRRYDVFMRFHSKEDHEDLLQTVISEHRTL

KRIQDLKEARAAGCRTSAEADRYLELKRGREAEEASRRAKEGGHAGASSQGGANVFMASE

SLGKDSISRPSGQASSSHVNDLYIMGFNETQLLSEAEKRLCCEIRLAPPLYLRMQEVMSR

EIFSGNVNNKADAHHLFKIEPSKIDRVYDMLVKKGLAPP

>XP_006467294.1 transcriptional adapter ADA2b isoform X2 [Citrus sinensis]

MGRSRGNFHANDEDPTQRSRRKKNVSSDNSDTAAPGQGAGEGKRALYHCNYCNKDITGKI

RIKCAVCPDFDLCIECFSVGVEVHPHKSNHPYRVMDNLSFPLICPDWNADDEILLLEGIE

MYGLGNWAEIAEHVGTKTKELCIEHYTNVYMNSPFFPLPDMSHVVGKNRKELLAMAKGHI

DDKKGPSKPGEATVKEESPFSPSRVKIEEMHKVGPSGRGLNADPQTERSSKGKKPVTSGN

DGPSLVELSGYNSKRQEFDPEYDNDAEQLLAEMEFKDADSEEERDIKLRVLRIYSKRLDE

RKRRKDFILERNLLYPNPFEKDLSPEERELCRRYDVFMRFHSKEDHEDLLQTVISEHRTL

KRIQDLKEARAAGCRTSAEADRYLELKRGREAEEASRRAKEGGHAGASSQGGANVFMASE

SLGKDSNSNSRPSGQASSSHVNDLYIMGFNETQLLSEAEKRLCCEIRLAPPLYLRMQEVM

SREIFSGNVNNKADAHHLFKIEPSKIDRVYDMLVKKGLAPP

>XP_031273471.1 transcriptional adapter ADA2 [Pistacia vera]

MGRSRGNFHSNDEDPTQRSRRKKNVSSENSESSAAGQGAGEGKRALYHCNYCNKDITGKI

RIKCAVCPDFDLCIECFSVGAEVTPHKCHHPYRVMDNLSFPLICPDWNADDEILLLEGIE

MYGLGNWAEVAEHVGTKTKEHCIEHYNSVYMNSPFFPLPDMSHVVGKNRKELLAMAKGHT

EDKKGSTMLGELTLKEESPFSPSRVKLEEPHKGGPSGRLISGSNAEVESGACTNRTNLAA

KAAVKKASNMTQVKDGPSIIKVEEPQTERSFKGKKPMSSANDGPSLVELSGYNPKRHEFD

PEYDNDAEQLLAEMEFKDTDTEEERELKLRVLRIYSKRLDERKRRKDFILERNLLYPNPF

EKDLSPEERAICRRYDVFMRFHSKEEHEELLQTVIAEHRTLKRIQELKEARAAGCRTSAE

ADRYLELKRKREAEEASRRSKDSAQVGPSSQGGANAFMASESGGKDSNSRPAGQASVSHV

NDFDILGFTETQLLSEAEKRLCCEIRLPPPLYLRMQEIISREVFSGNVTKKADVHHLFKI

EANKVDRVYDMLVKKGLAPP

>XP_031264776.1 transcriptional adapter ADA2-like isoform X2 [Pistacia vera]

MGRSRAVSHYADDDHNQKSKRKRTISSLENIETASTGQPFSEGKGALYHCNYCNKDISGF

VRIKCALCSDFDLCIECFSVGAEITPHKSNHPYRVMDNLSFPLICPDWNADEEILLLEGI

EMYGFGNWAEVAEHVGTKSKSQCIDHYNAIYLSSPCFPLPDLSHVMGKSREELLAMAKGH

GEVKKELTTVGEVTMNEESSFSPRVKSETLKKEDPAHQSSSSLTIEAGSHMHSSSSNTFS

STVKKALNIPQIKDSIKAEEPLSDRSIGEKKPRVAGDEKPSMTELSGYNFKRQEFEVEYD

NDAEQLLADMEFKDTDTDAERELKLRVLRIYSKRLDERKRRKDFIVERNLLYLDPFEKDL

SPEEKEIYQRFKVFMRFHSKEDHEELLKSIIEEHRIVKRIQDLQEALAAGFHTSAEANKF

LEQKRRKEAEENAQRVKSSSQAGPSGKVLQRPNSLKGEVDVSPCGVVRGSMALQPFGNVS

PSTMASSLDDWDISGFVGASLLSETEKQLCGEIRILPSHYLKMLEILSVEVMKGNISKKS

DAHDLFKVEASKVDRVYDMLVKKGIAKA

>XP_006469487.1 transcriptional adapter ADA2a isoform X5 [Citrus sinensis]

MGRYRAVSHVADEDHSQKSKRKRTISSLENVETASTGQPLSEGKGALYHCNYCNKDLSGM

VRIKCAMCSDFDLCVECFSVGAQIYPHESNHPYRVMDNLSFPLICPDWNADEEILLLEGI

EMYGFGNWGEVSEHVGTKSKSQCIDHYNAIYMNSPCFPLPDLSHVMGKNREELLAMAKEH

QQVKKELPTVAELALKEDAPFSTRMKPETRKEDTTRQSSSGLTTVEVNSIDPSNGNAFSF

KKASNMTQVKESVKVEEPQSDRSIGEKKLRTSGDERPSMKELSGYNFKRQEFEIEYDNDA

EHLLADMEFNKNDTDAERELKLRVLRIYGKRLDERKRRKDFILERNLLFPDPFERNLSPE

EREIYQQYKVFMRFHSKEDHEELLKSVIEEHRIVKRIQELQEAQAAGCRTSSEAHRFLEQ

KRKKEAEENGQRVKESGQAGPSGKVLQRPNSLKEVEVSPRGVVRGSTSLQPFGNDSYSTI

ASSLEDWDISGFVGADLLSETEKRLCGEIKILPAHYLKMLEILSVEIYKGNVSKKSDAHN

LFKVEPNKVDRVYDMLVRKGIAQA

>XP_024047094.1 transcriptional adapter ADA2a isoform X2 [Citrus clementina]

MGRYRAVSHVADEDHSQKSKRKRTISSLENVETASTGQPLSEGKGALYHCNYCNKDLSGM

VRIKCAMCSDFDLCVECFSVGAQIYPHESNHPYRVMDNLSFPLICPDWNADEEILLLEGI

EMYGFGNWGEVSEHVGTKSKSQCIDHYNAIYMNSPCFPLPDLSHVMGKNREELLAMAKEH

QQVKKELPTVAELAQKEDAPFSTRMKPETRKEDTTRQSSSGLTTVEVNSIDPSNGNAFSF

KKASNMTQVKESVKVEVLAEPQSDRSIGEKKLRTSGDERPSMKELSGYNFKRQEFEIEYD

NDAEHLLADMEFNKNDTDAERELKLRVLRIYGKRLDERKRRKDFILERNLLFPDPFERNL

SPEEREIYQQYKVFMRFHSKEDHEELLKSVIEEHRIVKRIQELQEAQAAGCRTSSEAHRF

LEQKRKKEAEENGQRVKESGQAGPSGKVLQRPNSLKEVEVSPRGVVRGSTSLQPFGNDSY

STIASSLEDWDISGFVGADLLSETEKRLCGEIKILPAHYLKMLEILSVEIYKGNVSKKSD

AHNLFKVEPNKVDRVYDMLVRKGIAQA

>EOY28942.1 Histone acetyltransferase complex component isoform 1 [Theobroma cacao]

MGRSRGNFHSADEDPTQRSRRKKNASAGENLESSSSGQGTNDGKRALYHCNYCNKDITGK

IRIKCAVCPDFDLCIECFSVGAEVTPHKSNHPYRVMDNLSFPLICPDWNADDEILLLEGI

EMYGLGNWAEVAEHVGTKTKEKCIEHYDNVYMKSPFFPLPDMSHVVGKNRKELLAMAKGH

GEDKKGSSMLGELTVKEESPFSPSRVKVEGGPSGRLLSGLNADVESGVRSSSSSTTPAAV

NKASNMAQVKDGNVKMEDPQMDRKGKKPNSLGNDGLVELSGYNPKRQEFDPEYDNDAEQL

LAEMEFKDTDTEEEHEIKLRVLRIYSKRLDERKRRKDFILERNLLYPNPFEKDLTPEERA

LCRRYDVFMRFLSKEEHEDLLQTIVSEHRTLKRIEELKEARAAGCRTSAEADRYLELKRK

REAEESSHRAKDGVHVNPSGQGGPNSFMASESVAKDSNSRPTAQASSSFATDLDIMGFSE

TQLLSEAEKRLCSEIRLPPPLYLRMLQIISEEIFSGNVTKKSDAHRLFKLDPSKTDRVYD

MLVKKGIAPP

>XP_021293995.1 transcriptional adapter ADA2b [Herrania umbratica]

MGRSRGNFHSADEDPTQRSRRKKNASAGENLESSSSGQGTHDGKRALYHCNYCNKDITGK

IRIKCAVCPDFDLCIECFSVGAEVTPHKSNHPYRVMDNLSFPLICPDWNADDEILLLEGI

EMYGLGNWAEVAEHVGTKTKEKCIEHYDNVYMKSPFFPLPDMSHVVGKNRKELLAMAKGH

GEDKKGSSMLGELTVKEESPFSPSRVKVEGGPSGRLLSGLNADVESGVRSSSSSTAPAAV

SKASNMAQVKDGNVKMEDPQMDRKGKKPNSLGNDGLVELSGYNPKRQEFDPEYDNDAEQL

LADMEFKDTDTEEEREIKLRVLRIYSKRLDERKRRKDFILERNLLYPNPFEKDLTPEEIA

LCRRYDVFMRFLSKEEHEDLLQTIVAEHRTLKRIEELKEARAAGCRTSAEADRYLELKRK

REAEESSHRAKDGVQVNPSGQGGPNSFMASESVAKDSNSRPTAQASSSFATDLDIMGFSE

TQLLSEAEKRLCGEIRLPPPLYLRMLQIISEEIFNGNVTKKSDAHRLFKLDPSKTDRVYD

MLVKKGIAPP

>XP_021293189.1 transcriptional adapter ADA2a isoform X1 [Herrania umbratica]

MGRSRTVSRSTEDDLNQSRSKRKRTVYGGVENLELPPSGVGHVANEAKGPALYHCNYCNK

DISGMVRIKCAVCPDFDLCVECFSVGAEVTPHKGNHPYRVMDNLSFPLICPDWNADEEIL

LLEGIEMYGFGNWTEVAEHVGTKSKSQCIDHYNAIYMNSPCFPLPDLSHVMGKSREELLA

MAKGNGQVRKEFTAHGEHTLKEESPVAAKVKYDAPRKDDPAYQSSSSLTGEVGSHIDSSS

GNSFQGSGKKTNLAQSKDGIKLEEPQADRSIGEKKLRVSTDEEPSMTELSGYNFKRQEFE

IEYDNDAEQLLADMEFKDTDTKPEHELKLRVLRIYSKRLDERKRRKDFILERNLLYPDPF

ERNLSPEEKEIYQRYKVFMRFHSKEEHEELLKSVIEEHRIVKRIQDLQEARAAGCRTAAE

ANKFIEQKRKKEAEENAQRLRESVQAGPSGKVLLHGSPQGVMRGSTSLQPISKEASTVIG

GATTLDDWDITGFIGADLLSDTEKKLCSEIRILPSHYLSMLQTLSVELMKGNIGKKSDAH

NLFKVEPSKVDRVYDMLVKKGIAQA

>XP_007045538.1 PREDICTED: transcriptional adapter ADA2a isoform X2 [Theobroma cacao]

MGRSRTVSRSTEDDLNQRSKRKRTAYGGVENLELPPSGVGQVANEAKGPALYHCNYCNKD

ISGMVRIKCAVCPDFDLCVECFSVGAEVTPHKGNHPYRVMDNLSFPLICPDWNADEEILL

LEGIEMYGFGNWTEVAEHVGTKSKSQCIDHYNAIYMNSPCFPLPDLSHVMGKSREELVAM

AKGNGQVRKEFTAHGEHTLKEESSVAAKVKYDAPRKDDPAYQSSSSLTGEAHIDSSSGNS

FQGSGKKTNLAQSKDGIKLEEPQADRSIGEKKLRVSTDEEPSMTELSGYNFKRQEFEIEY

DNDAEQLLADMEFKDTDTKPEHELKLRVLHIYSTRLDERKRRKDFILERNLLYPDPFERN

LSPEEKEIYQRYKVFMRFHSKEEHEELLKSVIEEHRIVKRIQDLQEARAAGCRTAAEANK

FIEQKRKKEAEENAQRLRESVQAGPSGKVLLHGSPQGVMRGSTSLQTISKESSTVIGGAT

TLDDWDITGFIGADLLSDTEKKLCSEIRILPSHYLSMLQTLSVEIMKGNIGKKSDAHNLF

KVEPSKVDRVYDMLVKKGIAQA

>EOY01368.1 ADA2 2A isoform 2 [Theobroma cacao]

MGRSRTVSRSTEDDLNQSRSKRKRTAYGGVENLELPPSGVGQVANEAKGPALYHCNYCNK

DISGMVRIKCAVCPDFDLCVECFSVGAEVTPHKGNHPYRVMDNLSFPLICPDWNADEEIL

LLEGIEMYGFGNWTEVAEHVGTKSKSQCIDHYNAIYMNSPCFPLPDLSHVMGKSREELVA

MAKGNGQVRKEFTAHGEHTLKEESSVAAKVKYDAPRKDDPAYQSSSSLTGEAHIDSSSGN

SFQGSGKKTNLAQSKDGIKLEEPQADRSIGEKKLRVSTDEEPSMTELSGYNFKRQEFEIE

YDNDAEQLLADMEFKDTDTKPEHELKLRVLHIYSTRLDERKRRKDFILERNLLYPDPFER

NLSPEEKEIYQRYKVFMRFHSKEEHEELLKSVIEEHRIVKRIQDLQEARAAGCRTAAEAN

KFIEQKRKKEAEENAQRLRESVQAGPSGKVLLHGSPQGVMRGSTSLQTISKESSTVIGGA

TTLDDWDITGFIGADLLSDTKKLCSEIRILPSHYLSMLQTLSVEIMKGNIGKKSDAHNLF

KVEPSKVDRVYDMLVKKGIAQA

>OMP07958.1 Zinc finger, ZZ-type [Corchorus olitorius]

MGRSRGNFHSADEDPTQRSRRKKNASAGENLESSTSGQGTNEGKKALYHCNYCNKDLTGK

IRIKCAFCPDFDLCIECFSVGAEVTPHKSNHPYRVMDNLSFPLICPDWNADDEMLLLEGI

EMYGLGNWAEVAEHVGTKSKEKCIEHYENVYMKSPVFPLPDMSHVVGKNRKELLAMAKGH

NEDKKGSSMFGELTVKEESPFSPSRVKVEGGPSGRLLSGLNADVESGIRSSSGSTKSAAV

KKASNMAQVKDGNVKMEDPQMDRSFKGKKPDSSGNDGPSLLELSGYNPKRQEFDPEYDND

AEQLLADMEFKDTDTEEERELKLRVLRIYSKRLDERKRRKDFILERNLLYPNPFEKDLTP

EERAICRRYDVFMRFHSKEEHEELLQTVISEHRSLKRIEELKDAQIAGCRTSAEADRYLE

QKRKREAEETSQRGKDGVQVNLSGPGGPNSFMASESVKDSNSRPTPQASSSYANDLDIMG

FSETQLLSEAEKRLCSEIRLPPPLYLRMLQIISEEVFNGNVTKKSDAHRLFKIEPTKTDR

VYDMLVKKGIAPP

>OMO65317.1 Zinc finger, ZZ-type [Corchorus capsularis]

MGRSRGNFHSADEDPTQRSRRKKNASAGENLESSTSGQGTNEGKKALYHCNYCNKDLTGK

IRIKCAFCPDFDLCIECFSVGAEVTPHKSNHPYRVMDNLSFPLICPDWNADDEMLLLEGI

EMYGLGNWAEVAEHVGTKSKEKCIEHYENVYMKSPVFPLPDMSHVVGKNRKELLAMAKGH

NEDKKGSSMFGELTVKEESPFSPSRVKVEGGPSGRLLSGLNADVESGIRSSSGSTKSAAV

KKASNMAQVKDGNVKMEDSQMDRSFKGKKSNPSGNDGPSLLELSGYNPKRQEFDPEYDND

AEQLLADMEFKDTDTEEERELKLRVLRIYSKRLDERKRRKDFILERNLLYPNPFEKDLTP

EERAICRRYDVFMRFHSKEEHEELLQTVISEHRSLKRIEELKEAQIAGCRTSAEADRYLE

QKRKREAEETSQRGKDGVQVNLSGPGGPNSFMASESVKDSNSRRTPQASSSYANDLDIMG

FSETQLLSEAEKRLCSEIRLPPPLYLRMLQIISEEVFNGNVTKKSDAHRLFKIEPTKTDR

VYDMLVKKGIAPP

>OMO98986.1 Zinc finger, ZZ-type [Corchorus capsularis]

MGRSRTVNRSNEEDLNQSSKSKRKRTASGVVENVELPSTGVGQVANEAKGPALYHCNYCN

KDISGMVRIKCAVCPDFDLCVECFSVGAEVTPHKANHPYRVMDNLSFPLICPDWNADEEI

LLLEGIEMYGFGNWTEVSEHVGTKSRSQCIDHYNAIYMNSPCFPFPDLSHVMGKSREELL

AMAKENGQVKKEFTTHDEVTLKHESTVAAKIKYEAPRKEDPAPQSSSSLTGEGRSHLDSS

SGNTFQGAGKITSNVAPIKDCIKVEEPQADRSIGEKKLRVSTDEEPSMTELSGYNFKRQE

FEIEYDNDAEQLLADMEFKDTDTKAEHELKLRVLHIYSKRLDERKRRKDFILERNLLYPD

PFERNLSPEEREIYQRYKVFMRFHSKEEHEELLKSVIEEHRIVKRIQDLQEARASGCRTA

AEANKFIEQKRKKEAEENAQRLKESAQAGTSGKGLFHSSPRGIIRGSTGLQPFGKESPTV

IGQATLSLSNLDEWDITGFIGADLLSDTEKQLCCEIRLLPSHYLSMLQTLSVEIMKGNIS

KKSDAHNLFKVEPSKVDRVYDMLVKKGIAQA

>XP_022720722.1 transcriptional adapter ADA2b-like isoform X2 [Durio zibethinus]

MGRSRGNFHSADEDPTQRSRRKKNASAGENLESSTSVQGTSEGKRALFHCNYCNKDITGK

IRIKCAICPDFDLCIECFSVGAEVTPHKSNHSYRVMDNLCYPLICPDWNADDEMLLLEGI

EMYGLGNWAEVAEHVGTKSKEKCIDHYSNVYMKSPFFPLPDMSHVVGKNRKELLAMAKGH

AEDKKGSSMLGELPVKEESPFSPSRVKVEGGSSGRLLSALNTDVESGVRSSSSSTASAAV

KKVSNMAQVKDGNVKMEDPQIDRNFGGKKPNSLGNDGPSLVELSGYNPKRQEFDPEYDND

AEQLLAEMEFKDTDTEEERELKLRVLRIYSKRLDERKRRKDFILERNLLYPNPFEKNLSP

EERALCRRYDVFMRFHSKEEHDELLETVIAEHRTLKRIEELKEAQVAGCRTSAEADRYLE

QKRKREAEESSQRAKDGVQGGPSGHGGPNSFMASESVGKESNSRPIAQASSSNANDLDIM

GFSETQLLSETEKRLCGEIRLPPPLYLRMLQIISEEIFNGNVTKKADARGLFKMEASKID

RVYDMLVKKGIAPA

>XP_022733546.1 transcriptional adapter ADA2a-like isoform X2 [Durio zibethinus]

MGRSRTVSRSTEEDLNQRSKRKRTGPGVVENVELPSTIVGQVPSEAKGPALYHCNYCNKD

LSGMVRIKCAVCPDFDLCVECFSVGAEVSPHKSNHPYRVMDNLSFPLICPEWNADEEILL

LEGIEMYGFGNWTEVAEHVGTKSKSQCIDHYNAIYMNSPCFPLPDLSHVMGKSREELLAM

AKGNSQVKKEFTAHGELTLKDESPTGATVKYEAPRKEDPAYQSSSSLTAEPQADRSIGEK

KPRVSTDEEPSMTELSGYNFKRQEFEIEYDNDAELLLADMEFKDTDTKAEHELKLRVLHI

YSKRLDERKRRKDFILERNLLYPDPFKRNLSPEEREIYQRCKVFMRFHSKEEHEELLKSV

IEEHRTVKRIQELQEARAAGCRTAAEANKYIEQKRKKEAEENAQRLRESVQAGPSGKVLL

HGSPRGIVRGSTGLQPFSKESSTVIGQDTLSTLDDWDITGFIGADLLSDTEKQLCNEIRI

LPTHYLSMLQTLSMEIMKGNISKKSDAYNLFKVEPSKVDRVYDMLVKKGIAQA

>TYJ17763.1 hypothetical protein E1A91_A09G075000v1 [Gossypium mustelinum]

MGRSRGNFHSTDEDPTQRSRRKKNASAGENLESSSSGQGTSEGKGALYHCNYCNKDITGK

IRIKCAICPDFDLCIECFSVGAEVTPHKSCHLYRVMDNLSFPLICPDWNADDEILLLEGI

EMYGLGNWAEVAEHVGTKSKEKCIDHYTNVYMKSPFFPLPDMSHVVGKNRKELLAMAKGH

TEDKKGTMLGELSVKEESPFSPSRVKVEGSNCGRIMSGLNADVESGARSSSNSTASATVV

KVSNMNQVKDGNVKMEDHQIDRNFGGKKPNSSGNEGPSLVELSGYNAKRQEFDPEYDNDA

EQLLAEMEFKDTDTEEERELKLRVLRIYSKRLDERKRRKDFILERNLLYPNPIEKDLSPE

ERALCRRYDVFMRFHSKEEHDELLQTVIAEHRTLRRIEELKEARAAGCRTSAEADRYLEQ

KRRREAEESSHRAKDGVQGGPGGQAAPNSFMASESVGKDSNSRPTAQASSSYAKDLDIMG

FAETQLLSETEKRLCSEIRLPPPLYLMMLQIISEEIFNGNVSKKADAHRLFKIEPSKIDR

VYDMLVKKGIAAP

>XP_017612085.1 PREDICTED: transcriptional adapter ADA2b [Gossypium arboreum]

MGRSRGNFHSADEDPTQRSRRKKNASAGENLESSSSGQGTSEGKGALYHCNYCNKDITGK

IRIKCSICPDFDLCIECFSVGAEVTPHKSSHLYRVMDNLSFPLICPDWNADDEILLLEGI

EMYGLGNWAEVAEHVGTKSKEKCIDHYTNVYMKSPFFPLPDMSHVVGKNRKELLAMAKGH

TEDKKGTMLGELSVKEESPFSPSRVKVEGSNCGRIMSGLNADVESGARSSSNSTASATVV

KVSNMNQVKDGNVKMEDHQIDRNFGGKKPNSSGNEGPSLVELSGYNAKRQEFDPEYDNDA

EQLLAEMEFKDTDTEEERELKLRVLRIYSKRLDERKRRKDFILERNLLYPNPIEKDLSPE

ERALCRRYDVFMRFHSKEEHDELLQTVIAEHRTLRRIEELKEARAAGCRTSAEADRYLEQ

KRKREAEESSHRAKDGVQGGPGGQAAPNSFMASESVGKDSNSRPTAQASSSYAKDLDIMG

FAETQLLSETEKRLCSEIRLPPPLYLRMLQIISEEIFNGNVSKKADAHRLFKIEPSKIDR

VYDMLVKKGIAAP

>KAB2012275.1 hypothetical protein ES319_D09G078100v1 [Hibiscus syriacus]

MGRSRGNFHSADEDPTQRSRRKKNASAGENLESSSSGQGTSEGKRALYHCNYCNKDITGK

IRIKCAICPDFDLCIECFSVGAEVTPHKSCHLYRVMDNLSFPLICPDWNADDEILLLEGI

EMYGLGNWAEVAEHVGTKSKEKCIDHYTNVYMKSPFFPLPDMSHVVGKNRKELLAMAKGH

TEDKKGTMLGELSVKEESPFSPSRVKVEGSNCGRIMSGLNADVESGARSSSNSTASATVV

KVSNMNQVKDGNVKMEDHQIDRNYGGKKPNSSGNEGPSLVELSGYNAKRQEFDPEYDNDA

EQLLAEMEFKDTDTEEERELKLRVLRIYSKRLDERKRRKDFILERNLLYPNPIEKDLSPE

ERALCRRYDVFMRFHSKEEHDELLQTVIAEHRTLKRIEELKEARAAGCRTSAEADRYLEQ

KRKREAEESSHRAKDGVQGGPGGQAAPNSFMASESVGKDSNARPTAQASSSYAKDLDIMG

FAETQLLSETEKRLCSEIRLPPPLYLRMLQIISEEIFNGNVSKKADAHRLFKIEPSKIDR

VYDMLVKKGIAAP

>KAE8731015.1 Transcriptional adapter ADA2 [Hibiscus syriacus]

MGRSRGNFHSADEDPTQRSRRKKNASAGENLDSSSSGQGTSEGKRALYHCNYCNKDITGK

IRIKCANCPDFDLCIECFSVGAEVTPHKSCHPYRVMDNLSFPLICPDWNADDEILLLEGI

EMYGLGNWTEVAEHVGTKSKEKCIDHYTNVYMKSPFFPLPDMSHVVGKNRKELLAMAKGH

TEDKKGPMLGELSVKEESLFSPSRVKVEGGSCGRLLSALNADVESGVRSNSNSTPLGGVM

KVSNMSQVKDGNVKMEDHQVDRNFGGKKPNSSGNDGPSLVELSGYNAKRQEFDPEYDNDA

EQLLAEMEFKDTDTEEERELKLRVLRIYSKRLDERKRRKDFILERNLLYSNPFEKDLSLE

ERALCRRYDVFMRFHSKEEHDELLQTVIAEHRTLKRIEELKEARAAGCRTSAEADRYLEQ

KRKREAEESSHRAKDGSQGGPGGQTAPNSFMASESVGKDSNSRPTAQASSSYAKDLDIMG

FAETQLLSETEKRLCSEIRLPPPLYLRMLQIISEEIFNGNISKKADAHRLFKIEPSKIDR

VYDMLVKKGIAAP

>XP_012484629.1 PREDICTED: transcriptional adapter ADA2b [Gossypium raimondii]

MGRSRGNFHSADEDPTQRSRRKKNASAGENLESSSSGQGTSEGKRALYHCNYCNKDITGK

IRIKCAICPDFDLCIECFSVGAEVSPHKSCHLYRVMDNLSFPLICPDWNADDEILLLEGI

EMYGLGNWAEVAEHVGTKSKEKCIDHYTNVYMKSPFFPLPDMSHVVGKNRKELLAMAKGH

TEDKKGTMLGELSVKEESPFSPSRVKVEGSNCGRIMSGLNADVESGARSSSNSTASATVV

KVSNMNQVKDGNVKMEDHQIDRNYGGKKPNSSGNEGPSLVELSGYNAKRQEFDPEYDNDA

EQLLAEMEFKDTDTEEERELKLRVLRIYSKRLDERKRRKDFILERNLLYPNPIEKDLSPE

ERALCRRYDVFMRFHSKEEHDELLQTVIAEHRTLKRIEELKEARAAGCRTSAEADRYLEQ

KRKREAEESSHRAKDGVQGGPGGQAAPNSFMASESVGKDSNARPTAQASSSYAKDLDIMG

FAETQLLSETEKRLCSEIRLPPPLYLRMLQIISEEIFNGNVSKKADAHRLFKIEPSKIDR

VYDMLVKKGIAAP

>TYH53236.1 hypothetical protein ES332_D09G085000v1 [Gossypium tomentosum]

MGRSRGNFHSADEDPTQRSRRKKNASAGENLESSSSGQGTSEGKRALYHCNYCNKDITGK

IRIKCAICPDFDLCIECFSVGAEVTPHKSCHLYRVMDNLSFPLICPDWNADDEILLLEGI

EMYGLGNWAEVAEHVGTKSKEKCIDHYTNVYMKSPFFPLPDMSHVVGKNRKELLAMAKGH

TEDKKGTMLGELSVKEESPFSPSRVKVEGSNCGRIMSGLNADVESGARSSSNITASATVV

KVSNMNQVKDGNVKMEDHQIDRNYGGKKPNSSGNEGPSLVELSGYNAKRQEFDPEYDNDA

EQLLAEMEFKDTDTEEERELKLQVLRIYSKRLDERKRRKDFILERNLLYPNPIEKDLSPE

ERALCRRYDVFMRFHSKQEHDELLQTVIAEHRTLKRIEELKEARAAGCRTSAEADQYLEQ

KKKREAEESSHRAKDGVQGGPGGQAAPNSFMASESVGKDSNARPTAQASSSYAKDLDIMG

FAETQLLSETEKRLCSEIRLPPPLYLRMLQIISEEIFNGNVSKKADAHRLFKIEPSKIDR

VYDMLVKKGIAAP

>KAB2084269.1 hypothetical protein ES319_A05G323800v1 [Gossypium barbadense]

MGRSRTVSRPTEEDLNQRSKRKKTASGSVENVELPSAGLGQVPSEAKGPALYHCNYCNKD

LSGMVRIKCAVCPDFDLCVECFSVGAEITPHKCNHPYRVMDNLSFPLICPDWNADEEILL

LEAIEMYGFGNWAEVAEHVGTKSKSQCIDHYNAIYMNSPCFPLPDLSHVMGKSREELLAM

AKGNGLVKKEFTTHGELTLKQESPAGAKVKYEAPRKEDPAHQSSCSLTGGSHIDSRSGGN

TFLVAGKKTSNMAQIKDGIKVEEPQADRSIGEKKPRVFTEEEPSMTVLSGYNFKRQEFEI

EYDNDAEQLLADMEFKDTDTKAERELKLRVLHIYSKRLDERKRRKDFILERNLLYPDPFE

RNLSPEEKEIYQRYKVFMRFHSKEEHRELLKSVIEEHRIVKRIQELQEARAAGCRTAAEA

NKFIEQKRKKEAEENAQRLKESVQAGPSGKVLLHGSPRGIVRGSTGLQPFSKESSTVLGQ

TTLSTLDDWDITGFIGADLLSDSEKQLCSEIRILPSHYLSMLQTLSMEIMKGNISKKSDA

HNLFKVEPGKVDRVYDMLVKKGIVQV

>TYH19294.1 hypothetical protein ES288_A05G341100v1 [Gossypium darwinii]

MGRSRTVSRPTEEDLNQRSKRKKTASGSVENVELPSAGLGQVPSEAKGPALYHCNYCNKD

LSGMVRIKCAVCPDFDLCVECFSVGAEITPHKCNHPYRVMDNLSFPLICPDWNADEEILL

LEAIEMYGFGNWAEVAEHVGTKSKSQCIDHYNAIYMNSPCFPLPDLSHVMGKSREELLAM

AKGNGLVKKEFTTHGELTLKQESPAGAKVKYEAPRKEDPAHQSSCSLTGEIGSHIDSRSG

GNTFLVAGKKTSNMAQIKDGIKVEEPQADRSIGEKKPRVFTEEEPSMTVLSGYNFKRQEF

EIEYDNDAEQLLADMEFKDTDTKAERELKLRVLHIYSKRLDERKRRKDFILERNLLYPDP

FERNLSPEEKEIYQRYKVFMRFHSKEEHRELLKSVIEEHRIVKRIQELQEARAAGCRTAA

EANKFIEQKRKKEAEENAQRLKESVQAGPSGQTTLSTLDDWDITGFIGADLLSDSEKQLC

SEIRILPSHYLSMLQTLSMEIMKGNISKKSDAHNLFKVEPGKVDRVYDMLVKKGIVQV

>KAE8691950.1 Transcriptional adapter ADA2 [Hibiscus syriacus]

MGRSRAVSRPTEEDLNQRSKRKKTGSSVVDNVELPSTGVGQVPSEAKGPALYHCNYCNKD

LSGMVRIKCAVCPDFDLCLECFSVGAEISPHKGNHPFRVMDNLSFPLICPDWNADEEILL

LEGIEMYGFGNWAEVAEHVGTKSKSQCIDHYNAIYMNSPCFPLADLSHVMGKSREELLAM

AKGNGQVKKEFTTHGELSLKQQSSSGAKVKYEAPRKEDPAHQSSSSLTGEIGSRIDSSSG

GNTFPGAGKKTSNMVQNKEGIKVEEPQSDRSIGEKKPRVFTEEEPSITELSGYNSKRQEF

EIEYDNDAEQLLADMEFKDTDTKAERELKLRVLHIYSKRLDERKRRKDFILERNLLYPDP

FERNLSPEEREIYQRYKVFMRFHSKEEHQELLKSVIEEHRIVKRIQELQEARAAGCRTTA

EANKFIEQKRKKETEKNAQRLKESVQAGPSGKVLLHGSPRGVVRGSTGLQPSGKESSTFM

GQTTLSTLDDWDITGFIGSDLLSDTEKQLCSEIRILPSHYLSMLQTLSMEIMKGNISKKS

DAHNLFKVEASKVDRVYDMLVKKGILQNAAPPVL
